# Paired box 6 gene delivery preserves beta cells and improves islet transplantation efficacy

**DOI:** 10.15252/emmm.202317928

**Published:** 2023-11-07

**Authors:** Wing Yan So, Yilie Liao, Wai Nam Liu, Guy A Rutter, Weiping Han

**Affiliations:** ^1^ Institute of Molecular and Cell Biology (IMCB), Agency for Science, Technology and Research (A*STAR) Singapore Singapore; ^2^ Zhongshan Institute for Drug Discovery Shanghai Institute of Materia Medica Chinese Academy of Sciences Zhongshan Guangdong 528400 China; ^3^ Center for Neurometabolism and Regenerative Medicine Bioland Laboratories Guangzhou Guangdong 510530 China; ^4^ Centre de Recherche du CHUM, Faculté de Médicine Université de Montréal Montréal QC Canada; ^5^ Section of Cell Biology and Functional Genomics, Division of Diabetes, Endocrinology and Metabolism, Department of Metabolism, Digestion and Reproduction, Faculty of Medicine Imperial College London London UK; ^6^ Lee Kong Chian Imperial Medical School Nanyang Technological University Singapore Singapore

**Keywords:** AAV, beta cells, diabetes, islet transplant, PAX6, Genetics, Gene Therapy & Genetic Disease, Metabolism

## Abstract

Loss of pancreatic beta cells is the central feature of all forms of diabetes. Current therapies fail to halt the declined beta cell mass. Thus, strategies to preserve beta cells are imperatively needed. In this study, we identified paired box 6 (PAX6) as a critical regulator of beta cell survival. Under diabetic conditions, the human beta cell line EndoC‐βH1, *db/db* mouse and human islets displayed dampened insulin and incretin signalings and reduced beta cell survival, which were alleviated by PAX6 overexpression. Adeno‐associated virus (AAV)‐mediated PAX6 overexpression in beta cells of streptozotocin‐induced diabetic mice and *db/db* mice led to a sustained maintenance of glucose homeostasis. AAV‐PAX6 transduction in human islets reduced islet graft loss and improved glycemic control after transplantation into immunodeficient diabetic mice. Our study highlights a previously unappreciated role for PAX6 in beta cell survival and raises the possibility that *ex vivo PAX6* gene transfer into islets prior to transplantation might enhance islet graft function and transplantation outcome.

## Graphical abstract

PAX6 was identified as a critical regulator of beta cell function, survival, and identity. Furthermore, PAX6 gene delivery in beta cells promoted beta cell potency, preserved beta cells, and enhanced the capability of glycemic control.

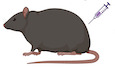

**The paper explained**

**Problem**

Loss of pancreatic beta cells is the central feature of all forms of diabetes. Current therapies fail to halt the declining beta cell mass. Islet transplantation is a beta cell replacement therapy adopted in clinical practice. However, post‐transplant islet graft loss and insufficient supply of cadaveric islets limit the success and utility of this modality of treatment.

**Results**

This work identified PAX6 as a critical regulator of beta cell function and survival. PAX6 expression was reduced in beta cells or islets under diabetic conditions, associated with loss of functional beta cells. Replenishment of PAX6 preserved beta cells and led to a sustained maintenance of glucose homeostasis in diabetic mice. Moreover, PAX6 overexpression in human islets prior to transplantation promoted islet potency, preserved post‐transplant islet graft and enhanced glycemic control in diabetic mice.

**Impact**

Through identification of PAX6 as a critical regulator of beta cell function, survival, and identity, this work demonstrates that *PAX6* gene transfer might overcome two of the major barriers of islet transplantation: post‐transplant islet graft loss and scarcity of islet donors. These findings thus provide grounds to support future clinical translation of PAX6 gene delivery to overcome beta cell failure in diabetes.

## Introduction

Diabetes is a global public health problem. There are currently 529 million people suffering from diabetes, estimated to exceed 1.31 billion by 2050 (GBD 2021 Diabetes Collaborators, 2023). Type 2 diabetes (T2D), which constitutes the majority of total diabetes cases, develops when beta cells fail to secrete sufficient insulin to cope with increased insulin demand. Reduced insulin‐secreting capability (Campbell & Newgard, 2021), beta cell death (Butler *et al*, 2003), and beta cell dedifferentiation (Efrat, 2019) are all likely to contribute to beta cell failure and progression to T2D. T2D patients are often treated with anti‐hyperglycemic medications such as metformin, sodium‐glucose transporter‐2 (SGLT2) inhibitors, thiazolidinediones (TZD), glucagon‐like peptide‐1 (GLP‐1) receptor agonists, and dipeptidyl peptidase‐4 (DPP‐4) inhibitors. However, none of the above approaches reliably halts the loss of functional beta cells, and hence T2D is usually considered to be an incurable chronic disease (Kahn *et al*, 2014; Taylor *et al*, 2021).

Autoimmune destruction of beta cells is the fundamental cause of type 1 diabetes (T1D) (Atkinson *et al*, 2014). Islet transplantation offers a minimally invasive treatment option for T1D and late‐stage T2D patients and, when successful, gives excellent metabolic control without triggering hypoglycemia (Shapiro *et al*, 2000; Lablanche *et al*, 2015; Rickels & Robertson, 2019). However, several factors including the scarcity of donor organs, islet graft loss, and the adverse effects of systemic immunosuppression remain substantial obstacles to the success of islet transplantation which limit its widespread application. Generally, multiple islet donors are required in a single transplantation to achieve insulin independence (Shapiro *et al*, 2000; Markmann *et al*, 2003), primarily because a substantial portion of the transplanted islets undergo apoptosis due to the exposure to a toxic environment of hyperglycemia, hypoxia, and immunosuppressant soon after transplantation (Shapiro *et al*, 2017; Gamble *et al*, 2018). In particular, clinical studies suggest that primary islet graft function has an overwhelming influence on the long‐term transplantation outcome (Vantyghem *et al*, 2009; Lam *et al*, 2022). These observations highlight the urgent need for the development of islet replacement strategies with improved islet graft survival after transplantation. Apart from improving the transplantation outcome, this would also enable the reduction in the number of transplantations, as well as the amount of islets required to attain euglycemia which in turn increases the number of patients that can receive islet transplantation with the limited islet supply.

Given that the loss of functional beta cells is a central event in both T1D and T2D, strategies that protect and restore functional beta cell mass are key and fundamental to prevent or delay diabetes onset and even to treat diabetes (Kahn *et al*, 2014; Vetere *et al*, 2014; Holman *et al*, 2020; Weir *et al*, 2020). Maintenance of optimal expression and activity of key transcription factors that control beta cell function and survival is crucial to the preservation of beta cells. Defining the roles of these transcription factors is of paramount importance to understand the pathophysiology of diabetes and paves the way to develop effective therapies against diabetes. In this regard, the paired box (PAX) proteins, specifically PAX4 and PAX6, have been shown to regulate pancreas development or function. PAX4 is predominantly expressed in pancreatic beta and delta cells with its maximal expression detected in the pancreas when beta cell differentiation is initiated (Sosa‐Pineda *et al*, 1997). Coinciding with the decline in beta cell proliferation after birth, PAX4 expression is low in adult beta cells (Brun & Gauthier, 2008). Acute overexpression of PAX4 has been found to protect beta cells against stress‐induced apoptosis and stimulates proliferation. Intriguingly, sustained PAX4 overexpression in mice results in beta cell dedifferentiation, blunted glucose‐stimulated insulin secretion (GSIS), and glucose intolerance (Hu He *et al*, 2011). Besides, increased PAX4 expression in human T2D islets (Brun *et al*, 2008) also implies that PAX4 up‐regulation may trigger beta cell dedifferentiation in human T2D. These observations reduce the enthusiasm for PAX4 as a therapeutic target for beta cell regeneration given the dedifferentiative feature of this gene. On the other hand, PAX6 also critically regulates pancreas development. Mice with PAX6 deficiency display severely reduced numbers of islet cells, suggesting that PAX6 is necessary for the differentiation of multiple islet endocrine cell types (Sander *et al*, 1997; Hart *et al*, 2013). PAX6 also maintains mature mouse beta cell function and identity (Gosmain *et al*, 2012; Swisa *et al*, 2017); while selective PAX6 deletion in adult mouse beta cells impairs insulin secretion and causes progressive hyperglycemia (Mitchell *et al*, 2017; Swisa *et al*, 2017). Our recent work (So *et al*, 2021) has further delineated the mechanism by which PAX6 regulates GSIS in beta cells through modulation of both proximal and distal signaling, while highlighting the pathophysiological role of PAX6 in T2D‐associated beta cell dysfunction. Furthermore, transcriptomic analysis of PAX6‐deficient beta cells revealed PAX6 as a potential regulator of cell proliferation in mouse (Swisa *et al*, 2017). Of note, several clinical studies find that *PAX6* gene mutations are associated with diminished insulin secretion and glycemic perturbations in humans (Yasuda *et al*, 2002; Nishi *et al*, 2005; Motoda *et al*, 2018), while PAX6 is found to be down‐regulated in human T2D islets (Taneera *et al*, 2012; So *et al*, 2021). These observations suggest a positive correlation between PAX6 activity/levels and human islet function. Since most of the mechanistic studies are mainly done in mouse beta cells, it is recognized that species differences do exist between human and rodent islets in terms of metabolism, cytoarchitecture, functional implications and the capability of beta cell replication (Cabrera *et al*, 2006; MacDonald *et al*, 2011; Kulkarni *et al*, 2012). However, evidence regarding the role of PAX6 in human beta cell survival, the key determinant of beta cell mass, is still missing. More importantly, whether PAX6 can serve as a therapeutic target for beta cell preservation remains ambiguous. In the present study, we provide evidence showing PAX6 as an essential regulator to conserve mouse and human beta cells. Replenishment of PAX6 under diabetic conditions prevents beta cell loss and ameliorates glucose homeostasis. Notably, this crucial role may potentially be applied in the protection of human islet graft to enhance the efficacy of islet transplantation.

## Results

### PAX6 is essential to preserve human pancreatic beta cells

**Figure 1:**
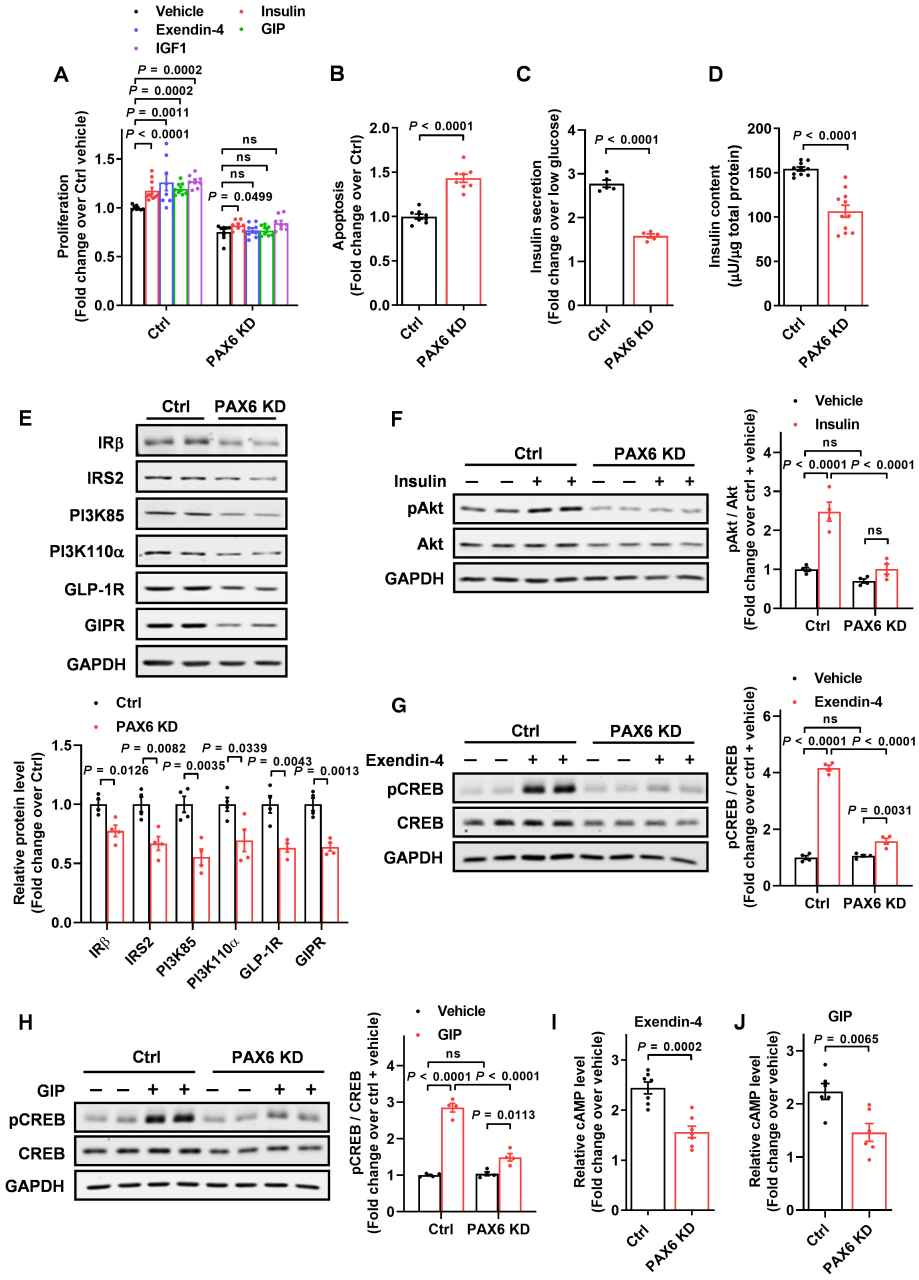
PAX6 is essential to preserve human pancreatic beta cells AEndoC‐βH1 cells with PAX6 knockdown were subjected to cell proliferation measurement indexed by BrdU labeling after 72‐h treatment with insulin (100 nM), Exendin‐4 (10 nM), GIP (10 nM), or IGF1 (50 ng/ml) (*n* = 8–10).B–D(B) Cell apoptosis (*n* = 8), (C) GSIS (*n* = 5), and (D) insulin content (*n* = 10–11) were measured in control and PAX6 knockdown cells.EProtein expression of insulin and incretin signaling components in cells with PAX6 knockdown (*n* = 4).FPhosphorylated and total Akt abundance in cells with PAX6 knockdown were measured after 15‐min insulin (100 nM) stimulation (*n* = 4).G, HPhosphorylated and total CREB abundance in cells with PAX6 knockdown were measured after 15‐min (G) Exendin‐4 (10 nM) or (H) GIP (10 nM) stimulation (*n* = 4).I, JcAMP concentration was measured in cells with PAX6 knockdown after 15‐min (I) Exendin‐4 (10 nM) or (J) GIP (10 nM) stimulation (*n* = 6–7). Data information: Each *n* represents an independent biological replicate (A–J). Mann–Whitney test (A). Unpaired Student's *t*‐test (B–E, I, J). Two‐way ANOVA (F–H). Data are means ± SEM. ns, nonsignificant. Source data are available online for this figure.

To elucidate the role of PAX6 in beta cell survival, PAX6 expression was silenced in the human beta line EndoC‐βH1 (Appendix Fig S1A) and cell survival was assessed. Hormones or factors that are well‐known to stimulate beta cell proliferation including insulin, exendin‐4 (GLP‐1 analog), glucose‐dependent insulinotropic polypeptide (GIP), and insulin‐like growth factor 1 (IGF1) were used to stimulate cell proliferation. Results showed that lentivirus‐mediated PAX6 knockdown blocked cell proliferation stimulated by insulin, exendin‐4, GIP, and IGF1 (Fig 1A). In addition, PAX6 knockdown augmented cell apoptosis (Fig 1B), decreased GSIS (Fig 1C), and insulin content (Fig 1D). As both insulin and incretin signalings are crucial cellular events for beta cell survival (Drucker, 2006; Leibiger *et al*, 2008), we next examined the interaction between PAX6 and these signaling pathways in the EndoC‐βH1 cells. PAX6 knockdown suppressed the protein expression of the signaling components, including insulin receptor β (IRβ), insulin receptor substrate 2 (IRS2), phosphatidylinositol 3‐kinase 85‐kDa (PI3K85) and 110‐kDa (PI3K110α) subunits, GLP‐1 receptor (GLP‐1R), and GIP receptor (GIPR) (Fig 1E). Consistently, PAX6 knockdown attenuated both insulin and incretin signaling responses as shown by diminished insulin‐induced Akt phosphorylation and exendin‐4‐ or GIP‐induced cAMP response element‐binding protein (CREB) phosphorylation (Fig 1F–H). Exendin‐4‐ or GIP‐induced cAMP generation was also suppressed by PAX6 knockdown (Fig 1I and J). Loss of beta cell identity is an emerging mechanism causing beta cell deficit, especially in T2D (Talchai *et al*, 2012; Spijker *et al*, 2015), and PAX6 is required to maintain adult mouse beta cell identity (Mitchell *et al*, 2017; Swisa *et al*, 2017). In line with the previous mouse studies, PAX6 knockdown in EndoC‐βH1 cells led to decreased gene expression of several key beta cell transcription factors including *MAFA*, *NKX6‐1*, *PDX1*, *PAX4*, and *NEUROD1* (Appendix Fig S1B), suggesting a similar role of PAX6 in maintaining human beta cell identity.

### PAX6 down‐regulation reduces beta cell survival under diabetic condition

**Figure 2:**
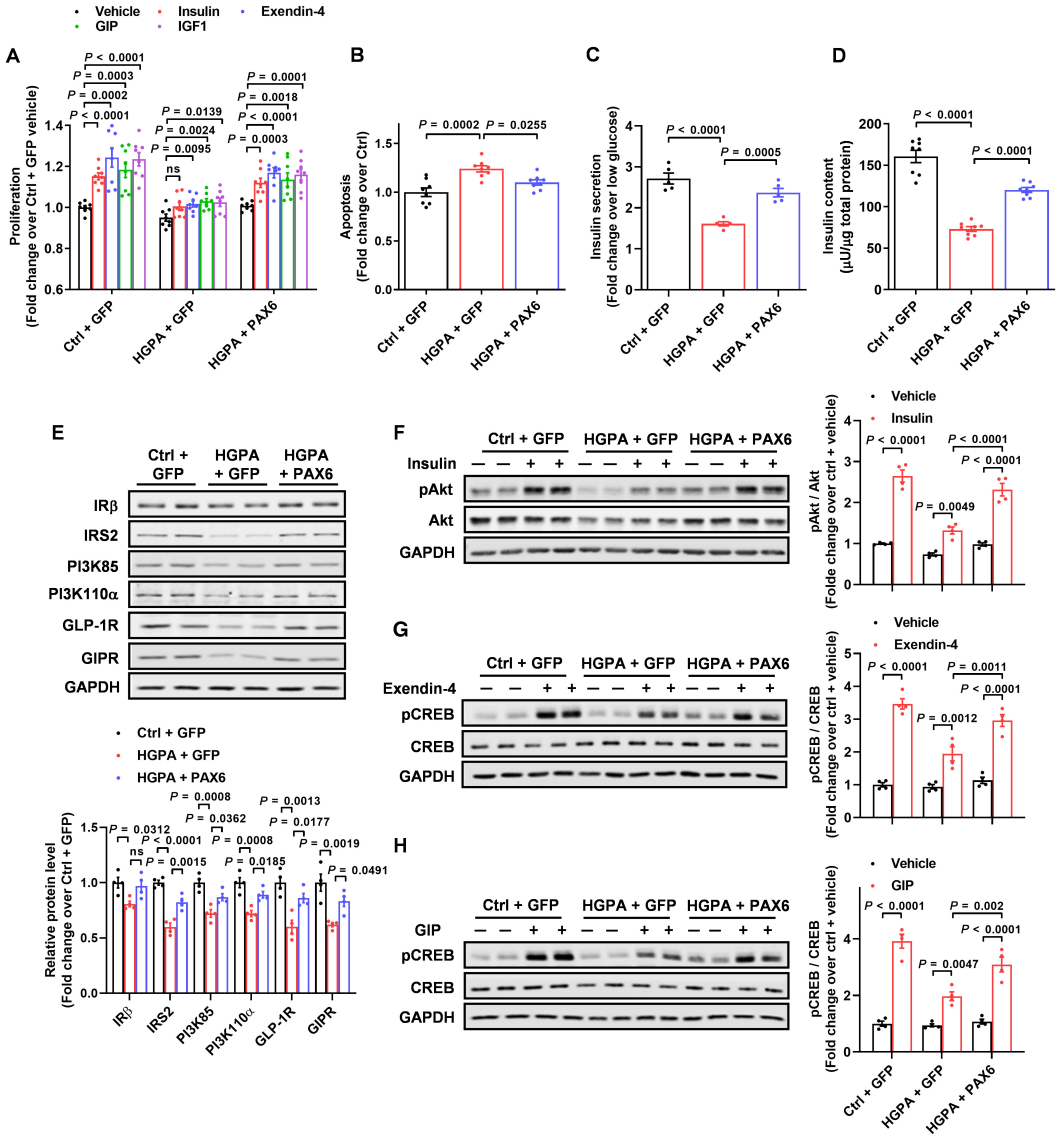
PAX6 down‐regulation reduces beta cell survival under diabetic condition AEndoC‐βH1 cells with PAX6 overexpression were subjected to cell proliferation measurement indexed by BrdU labeling after 72‐h exposure to normal or HGPA condition with insulin (100 nM), Exendin‐4 (10 nM), GIP (10 nM), or IGF1 (50 ng/ml) (*n* = 8).B–EAfter 72‐h exposure to normal or HGPA condition, (B) cell apoptosis (*n* = 8), (C) GSIS (*n* = 5), (D) insulin content (*n* = 8), and (E) protein expression of insulin and incretin signaling components (*n* = 4) were measured in cells with PAX6 overexpression.FPhosphorylated and total Akt abundance were measured in cells with PAX6 overexpression after 15‐min insulin (100 nM) stimulation (*n* = 4).G, HPhosphorylated and total CREB abundance were measured in cells with PAX6 overexpression after 15‐min (G) Exendin‐4 (10 nM) or (H) GIP (10 nM) stimulation (*n* = 4). Data information: Each *n* represents an independent biological replicate (A–H). Unpaired Student's *t*‐test (A). One‐way ANOVA (B–E). Two‐way ANOVA (F–H). Data are means ± SEM. ns, nonsignificant. Source data are available online for this figure.

As beta cell loss is a hallmark of both T1D and T2D, we examined the potential participation of PAX6 in diabetes‐associated beta cell loss by treating EndoC‐βH1 cells with high glucose and palmitic acid (HGPA) to mimic the diabetic condition. Extended (72 h) HGPA treatment repressed PAX6 expression (Appendix Fig S1C) and cell proliferation stimulated by insulin, exendin‐4, GIP and IGF1, which were reversed by PAX6 overexpression (Appendix Fig S1C and Fig 2A). Moreover, HGPA treatment modestly increased cell apoptosis but markedly decreased GSIS and insulin content, which were counteracted by PAX6 overexpression (Fig 2B–D). Mechanistically, HGPA treatment attenuated protein expression of IRβ, IRS2, PI3K85 PI3K110α, GLP‐1R, and GIPR, which was alleviated by PAX6 restoration in cells (Fig 2E). Concurrently, HGPA treatment prominently inhibited insulin and incretin signalings as evidenced by the diminution of Akt and CREB phosphorylation upon insulin and exendin‐4/GIP stimulation, whereas PAX6 replenishment mitigated HGPA's effects (Fig 2F–H). Effects of PAX6 overexpression under normal condition were also examined. Results showed that PAX6 overexpression (Appendix Fig S1D) increased cell proliferation stimulated by insulin, exendin‐4, GIP and IGF1 (Appendix Fig S1E). PAX6 overexpression inhibited cell apoptosis (Appendix Fig S1F), increased GSIS (Appendix Fig S1G) and insulin content (Appendix Fig S1H). On top of that, PAX6 overexpression also enhanced insulin and incretin signalings (Appendix Fig S1I–L). Overall, these results show that PAX6 overexpression promotes beta cell function and survival under both normal and diabetic conditions.

### Beta cell‐specific PAX6 overexpression promotes beta cell expansion and alleviates glycemic disturbance in streptozotocin (STZ)‐induced diabetic mice

**Figure 3:**
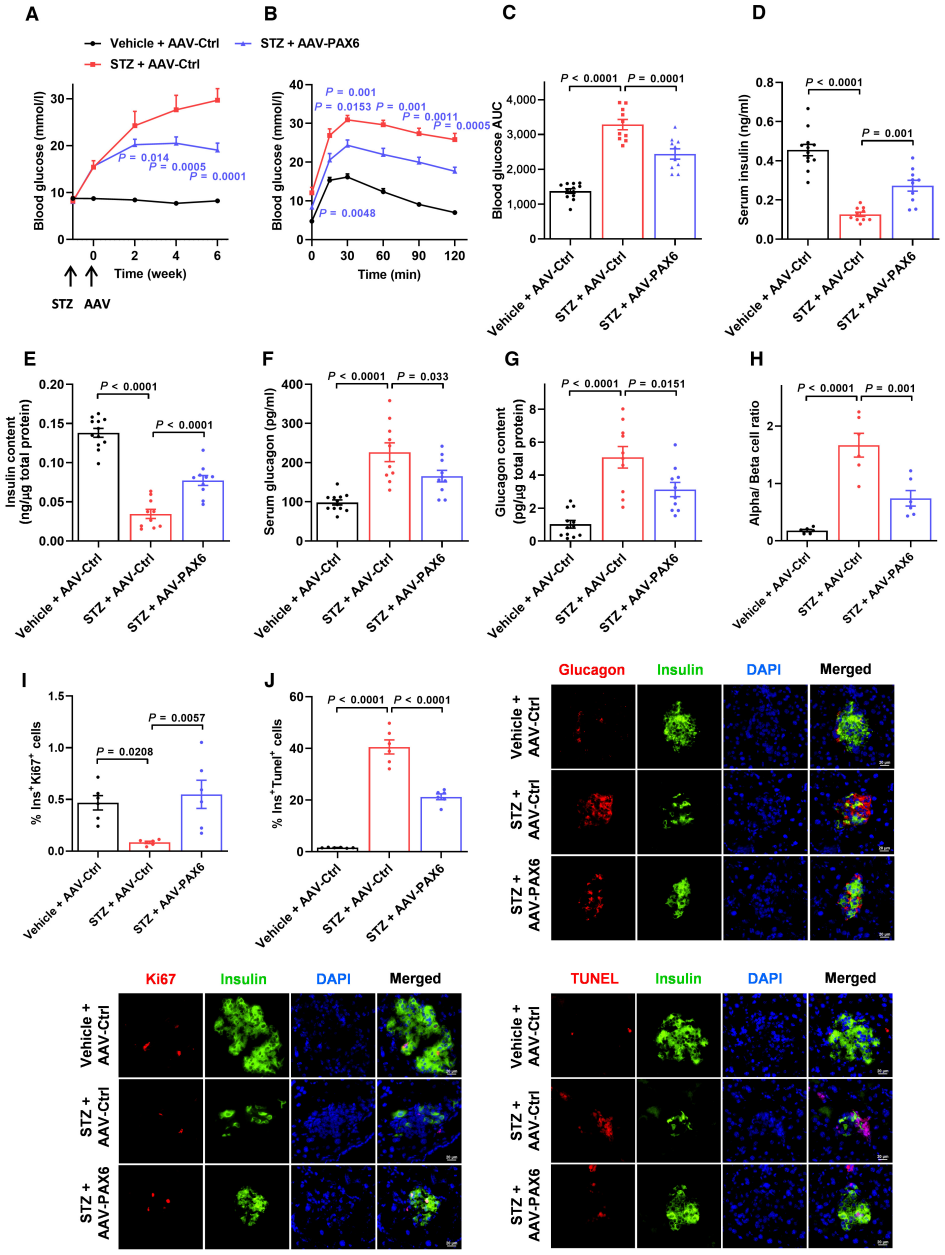
Beta cell‐specific PAX6 overexpression promotes beta cell expansion and alleviates glycemic disturbance in STZ‐induced diabetic mice A, B(A) Fasting blood glucose and (B) glucose tolerance of control or STZ‐treated mice with AAV injection (*n* = 10–12).CGlucose profiles calculated as AUC (*n* = 10–12).D–G(D) Serum insulin, (E) pancreatic insulin content, (F) serum glucagon, and (G) pancreatic glucagon content of control or STZ‐treated mice with AAV injection (*n* = 10–12).H–JRepresentative immunostaining and quantification showing (H) alpha‐to‐beta cell ratio, (I) insulin/Ki67 and (J) insulin/TUNEL signals in pancreatic islets of control or STZ‐treated mice with AAV injection (*n* = 6). Scale bar = 20 μm. Data information: Each *n* represents the measurement of a sample from distinct mice (A–J). Unpaired Student's *t*‐test and Mann–Whitney test (A, B). STZ + AAV‐PAX6 versus STZ + AAV‐Ctrl. One‐way ANOVA (C–J). Data are means ± SEM. AUC, area under the curve. Source data are available online for this figure.

The above *in vitro* data illustrated the evident modulatory role of PAX6 in human beta cell survival. We then substantiated this role in a physiological diabetic situation using STZ‐induced diabetic mice, which exhibit severe beta cell loss. STZ administration led to the development of overt diabetes (Fig 3A) and a significant diminution of PAX6 protein expression in pancreas (Appendix Fig S2A). We then adopted an adeno‐associated virus (AAV) approach to overexpress PAX6 in beta cells in order to investigate the therapeutic potential of PAX6 in beta cell survival and diabetes management. Immunofluorescence staining showed that most of the exogenous PAX6 was expressed in beta cells (Appendix Fig S2B) with minimal expression in other internal organs (Appendix Fig S2C). Strikingly, AAV‐PAX6 progressively relieved hyperglycemia in STZ‐treated mice 2 weeks after injection and the effect was sustained for at least 6 weeks (Fig 3A). Glucose tolerance was also markedly improved (Fig 3B and C). Concomitantly, serum insulin level and pancreatic insulin content were decreased drastically by STZ administration, while AAV‐PAX6 partially but significantly increased both of them (Fig 3D and E). On the contrary, STZ treatment enhanced both serum and pancreatic glucagon levels but these were reduced by AAV‐PAX6 (Fig 3F and G). Consistent with the alterations in circulating and pancreatic insulin and glucagon levels, immunofluorescence staining showed that STZ caused a severe beta cell loss and prominently increased the alpha‐to‐beta cell ratio in islets, which was reversed by PAX6 overexpression (Fig 3H). As the determining factors of cell survival, beta cell proliferation and death were evaluated. Proliferating beta cells labeled by both Ki67 and insulin were normally observed in control mouse islets but were barely detected in STZ‐treated mice; whereas PAX6 overexpression in beta cells stimulated cell proliferation (Fig 3I). Besides, STZ substantially elevated the amount of TUNEL‐positive apoptotic cells, which was partially rescued by AAV‐PAX6 (Fig 3J). In line with these findings, the significant reduction of beta cell mass in STZ‐treated mice was reversed by AAV‐PAX6 (Appendix Fig S2D). On the other hand, it has been reported that diminished intra‐islet insulin and γ‐aminobutyric acid (GABA) content as a result of STZ‐induced beta cell ablation would lead to alpha cell expansion (Feng *et al*, 2017). Our results showed that pancreatic GABA content was decreased (Appendix Fig S2E) while proliferating alpha cells (positively labeled for both Ki67 and glucagon) and alpha cell mass (Appendix Fig S2F and G) were increased in STZ‐treated mice. Notably, these observations were reversed by PAX6 replenishment and were consistent with the corresponding changes in serum and pancreatic glucagon levels. Effects of AAV‐PAX6 in normal mice were also investigated. Unlike the marked effects in STZ‐treated mice, PAX6 overexpression in beta cells of normal mice (Appendix Fig S3A and B) resulted in a mild reduction in blood glucose (Appendix Fig S3C) and slight improvement in glucose tolerance (Appendix Fig S3D and E). Serum insulin level and pancreatic insulin content were increased by AAV‐PAX6 administration (Appendix Fig S3F and G) while serum and pancreatic glucagon levels were not significantly altered (Appendix Fig S3H and I). Immunofluorescence staining showed that the islet alpha‐to‐beta cell ratio was not altered by PAX6 overexpression (Appendix Fig S3J). Moreover, the anti‐apoptotic effect of AAV‐PAX6 was not evident in normal mouse islets as both AAV‐Ctrl‐ and AAV‐PAX6‐treated mice displayed a low amount of TUNEL‐positive apoptotic cells in islets (Appendix Fig S3K). Notably, similar to the effects in STZ‐treated mice, AAV‐PAX6 increased beta cell proliferation (Appendix Fig S3L) and beta cell mass (Appendix Fig S3M) in normal mice, whereas alpha cell mass was not significantly altered (Appendix Fig S3M). Taken together, AAV‐PAX6 stimulates beta cell proliferation but mildly improves glucose homeostasis in normal mice; whereas in STZ‐induced diabetic mice, *PAX6* gene delivery promotes beta cell expansion but restraining alpha cell expansion, which in turn modulates the serum levels of insulin and glucagon and alleviates glycemic disturbance.

### Beta cell‐specific PAX6 replenishment preserves beta cells and ameliorates glucose homeostasis in *db/db* mice

**Figure 4:**
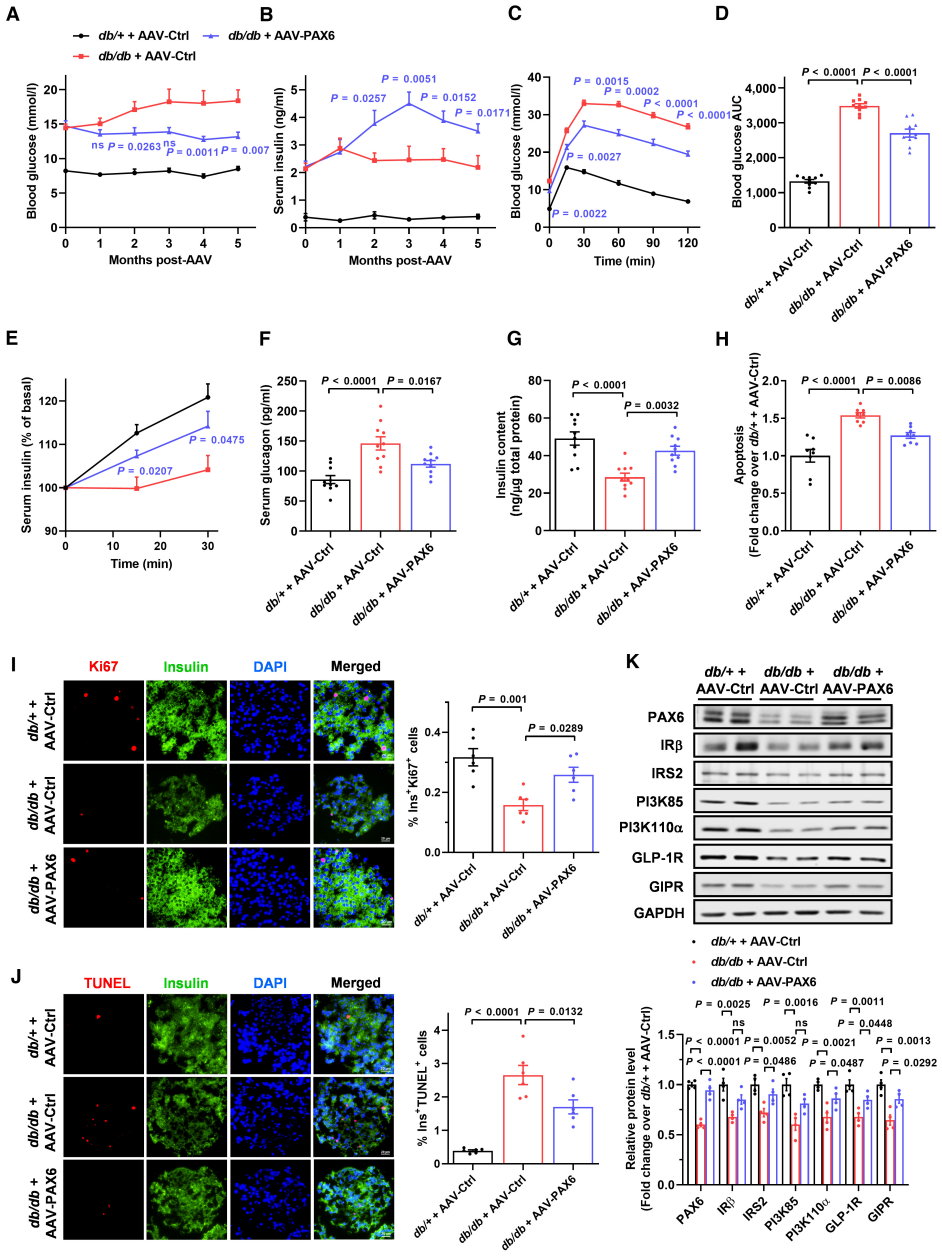
Beta cell‐specific PAX6 replenishment preserves beta cells and ameliorates glucose homeostasis in *db/db* mice A, B(A) Fasting blood glucose and (B) serum insulin of *db*/+ or *db/db* mice at the indicated time points after AAV injection (*n* = 10).CIPGTT of *db*/+ or *db/db* mice with AAV injection (*n* = 10).DGlucose profiles calculated as AUC (*n* = 10).ESerum insulin during IPGTT expressed as percent of basal (*n* = 10).F–H(F) Serum glucagon, (G) islet insulin content, and (H) islet apoptosis of *db*/+ or *db/db* mice with AAV injection (*n* = 8–10).I, JRepresentative immunostaining and quantification showing (I) insulin/Ki67 and (J) insulin/TUNEL signals in pancreatic islets of *db*/+ or *db/db* mice with AAV injection (*n* = 6). Scale bar = 20 μm.KProtein expression of insulin and incretin signaling components in pancreatic islets of *db*/+ or *db/db* mice with AAV injection (*n* = 4). Data information: Each *n* represents the measurement of a sample from distinct mice (A–K). Unpaired Student's *t*‐test and Mann–Whitney test (A–C). *db/db* + AAV‐PAX6 versus *db/db* + AAV‐Ctrl. Unpaired Student's *t*‐test (E). *db/db* + AAV‐PAX6 versus *db/db* + AAV‐Ctrl. One‐way ANOVA (D, F–K). Data are means ± SEM. AUC, area under the curve; ns, nonsignificant. Source data are available online for this figure.

The role of PAX6 in beta cell preservation was further assessed using a T2D model. *db/db* mice represent a model of T2D with obesity that develops beta cell failure, characterized by severe hyperglycemia, beta cell dysfunction, loss of beta cell identity, and PAX6 down‐regulation in islets (Dalboge *et al*, 2013; Swisa *et al*, 2017). *db/db* mice with PAX6 replenishment displayed a long‐term improvement in hyperglycemia and elevated serum insulin level with an overall trend up to 5 months compared to *db/db* mice receiving AAV‐Ctrl (Fig 4A and B). AAV‐PAX6 also resulted in improved glucose tolerance and enhanced insulin secretion (Fig 4C–E). Hyperglucagonemia, a key driver of T2D progression, was also mitigated by AAV‐PAX6 (Fig 4F). For beta cell survival, *db/db* mouse islets exhibited reduced insulin content and increased apoptosis, which were ameliorated by PAX6 replenishment (Fig 4G and H). In corroboration, immunofluorescence staining revealed a diminution of Ki67‐positive and an increase in TUNEL‐positive beta cells in *db/db* mouse islets, which were reversed by AAV‐PAX6, suggesting that PAX6 overexpression stimulated beta cell proliferation while averting cell apoptosis (Fig 4I and J). Moreover, consistent with the changes in insulin and glucagon levels, PAX6 replenishment increased beta cell mass but decreased alpha cell mass in *db/db* mice (Appendix Fig S4A and B). Mechanistically, suppressed protein expression of IRβ, IRS2, PI3K85 PI3K110α, GLP‐1R, and GIPR was observed in *db/db* mouse islets (Fig 4K), rendering a repression of insulin‐induced Akt phosphorylation and exendin‐4‐ or GIP‐induced CREB phosphorylation (Appendix Fig S4C–E). Noticeably, PAX6 replenishment restored the protein expression of most of the components as well as the insulin and incretin signaling pathways (Fig 4K and Appendix Fig S4C–E). Change in beta cell identity was also detected. Expression of MAFA, NKX6.1, and PDX1 was reduced in *db/db* mouse islets, which was increased by AAV‐PAX6 (Appendix Fig S5A and B). Aldehyde dehydrogenase 1 isoform A3 (ALDH1A3), a progenitor cell marker (Marcato *et al*, 2011) enriched in dedifferentiated beta cells (Kim‐Muller *et al*, 2016), was profoundly up‐regulated in *db/db* mouse islets but was partially reversed by PAX6 overexpression (Appendix Fig S5C). Furthermore, a significant presence of insulin and glucagon double‐positive cells was detected in islets of *db/db* mice compared to the lean control, further supporting beta cell dedifferentiation and upregulation of the alpha cell program in diabetes. Importantly, AAV‐PAX6 notably rescued these phenotypes (Appendix Fig S5D). Collectively, these results indicate that PAX6 replenishment preserves beta cells through promotion of beta cell survival and maintenance of beta cell identity, thereby improving glucose homeostasis.

### Beta cell‐specific PAX6 replenishment enhances beta cell survival and maintains beta cell identity in human T2D islets

**Figure 5:**
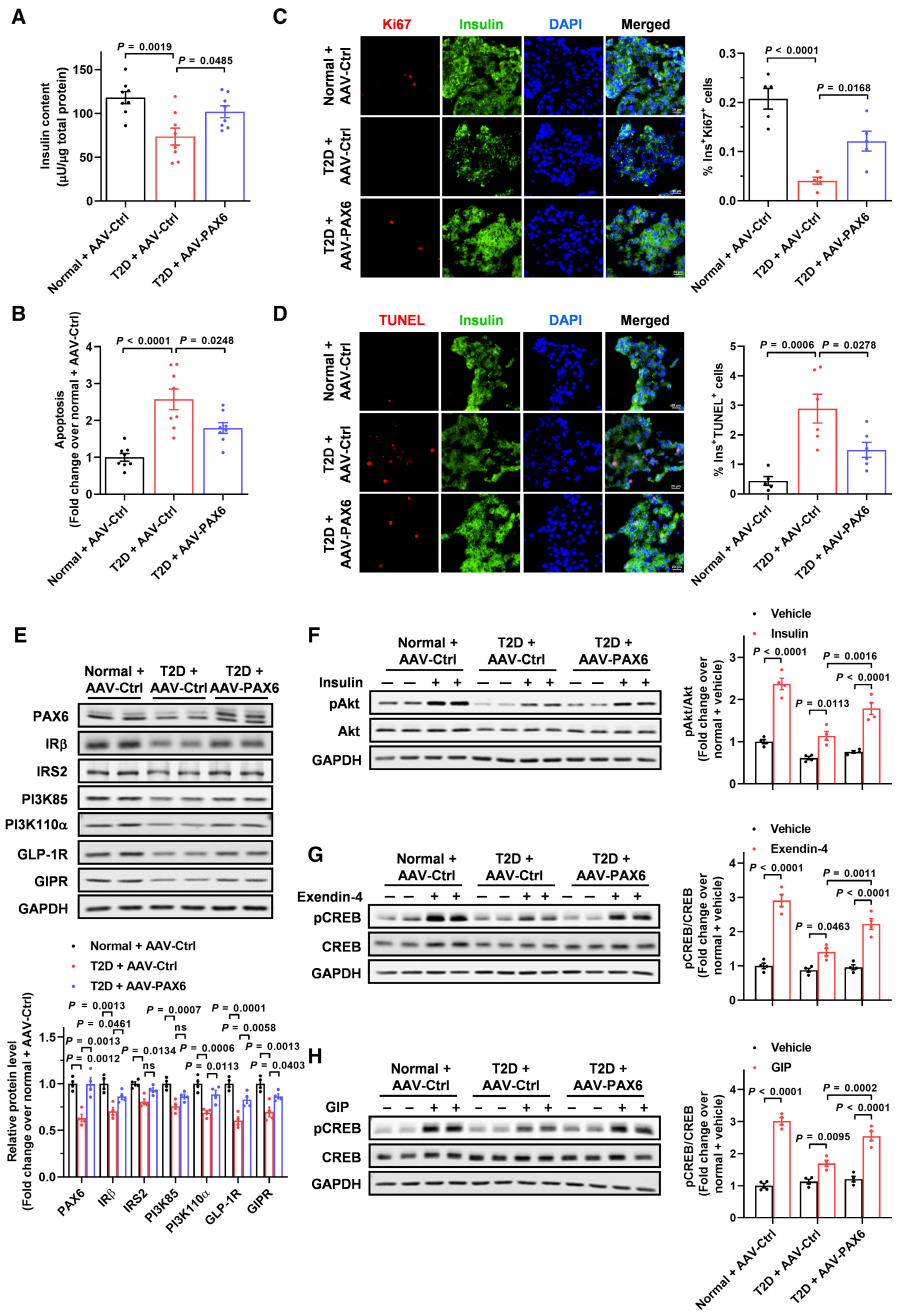
Beta cell‐specific PAX6 replenishment enhances beta cell survival and maintains beta cell identity in human T2D islets A, B(A) Islet insulin content and (B) islet apoptosis of normal or T2D human islets with AAV transduction (*n* = 8).C, DRepresentative immunostaining and quantification showing (C) insulin/Ki67 and (D) insulin/TUNEL signals in normal or T2D human islets with AAV transduction (*n* = 5–6). Scale bar = 20 μm.EProtein expression of insulin and incretin signaling components in normal or T2D human islets with AAV transduction (*n* = 4).FPhosphorylated and total Akt in human islets after 15‐min insulin (100 nM) stimulation (*n* = 4).G, HPhosphorylated and total CREB in human islets after 15‐min (G) Exendin‐4 (10 nM) or (H) GIP (10 nM) stimulation (*n* = 4). Data information: Each *n* represents an independent biological replicate (A–H). One‐way ANOVA (A–D). One‐way ANOVA and Kruskal–Wallis test (E). (F–H) Two‐way ANOVA. Data are means ± SEM. ns, nonsignificant. Source data are available online for this figure.

To validate our findings for clinical relevance, we examined primary pancreatic islets from healthy donors or T2D patients. Concurrent with the results observed in *db/db* mice, T2D islets displayed suppressed GSIS, lower insulin content, and enhanced apoptosis, all of which were rescued by AAV‐PAX6 (Appendix Fig S6A, Fig 5A and B). Immunofluorescence staining revealed that cell proliferation occurred in beta cells of normal islets as shown by the insulin and Ki67 double‐positive signal, although at a modest level; while the Ki67 signal was barely detected in T2D islets. Strikingly, PAX6 replenishment significantly enhanced the amount of Ki67 signal in T2D islets, indicating a stimulation of beta cell proliferation (Fig 5C). Besides, T2D islets showed an elevated number of TUNEL‐positive apoptotic beta cells that was rescued by AAV‐PAX6 (Fig 5D). These results further substantiated the protective role of PAX6 in human beta cell survival. Insulin and incretin signalings were also evaluated. Diminished protein expression of IRβ, IRS2, PI3K85 PI3K110α, GLP‐1R, and GIPR was observed in T2D islets (Fig 5E), which was concomitant with the attenuation of insulin‐induced Akt phosphorylation and exendin‐4‐ or GIP‐induced CREB phosphorylation (Fig 5F–H). Of note, PAX6 replenishment increased the protein expression of those signaling components as well as the insulin‐ and incretin‐induced signaling pathways (Fig 5E–H). Beta cell identity was also assessed as beta cell dedifferentiation is implicated in the pathogenesis of human T2D (Spijker *et al*, 2015). T2D islets display a reduction in mRNA and protein expression of MAFA, NKX6.1, and PDX1 while PAX6 replenishment enhanced their expression levels (Appendix Fig S6B and C). ALDH1A3 expression was also elevated in T2D islets but reduced by AAV‐PAX6 (Appendix Fig S6D). Furthermore, a higher portion of insulin and glucagon double positive cells was observed in T2D islets compared to normal islets, which was reversed by AAV‐PAX6 (Appendix Fig S6E). Our results demonstrate that PAX6 replenishment preserves beta cells through enhancing cell survival and maintaining beta cell identity in human T2D islets.

### Beta cell‐specific PAX6 overexpression enhances glycemic control by human T2D islets after transplantation into immunodeficient diabetic mice

**Figure 6:**
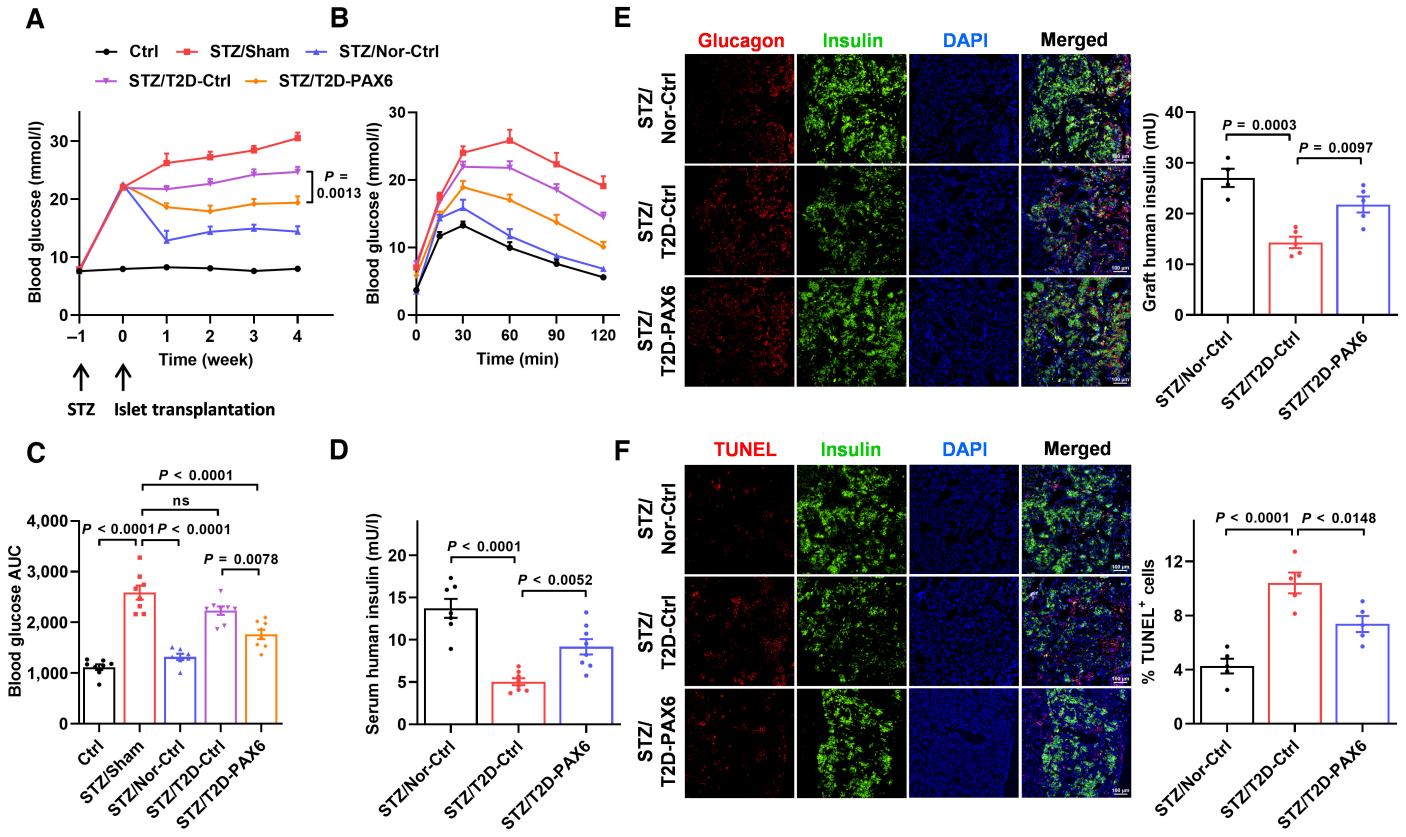
Beta cell‐specific PAX6 overexpression enhances glycemic control by human T2D islets after transplantation into immunodeficient diabetic mice A–C(A) Fasting blood glucose, (B) glucose tolerance, and (C) glucose profiles calculated as AUC of control or STZ‐treated mice transplanted with normal or T2D human islets (*n* = 7–8).DSerum human insulin (*n* = 7–8).ERepresentative immunostaining showing insulin/glucagon signal and ELISA measurement of human insulin content in human islet grafts (*n* = 4–5). Scale bar = 100 μm.FRepresentative immunostaining and quantification showing insulin/TUNEL signal in human islet grafts (*n* = 5). Scale bar = 100 μm. Data information: Each *n* represents the measurement of a sample from distinct mice (A–F). One‐way ANOVA (A–F). Data are means ± SEM. AUC, area under the curve; ns, nonsignificant. Source data are available online for this figure.

To assess the protective role of PAX6 in human islet survival and function in terms of *in vivo* glycemic control, normal or T2D islets transduced with AAV‐Ctrl or AAV‐PAX6 were transplanted into STZ‐induced diabetic NOD scid gamma (NSG) mice with glucose homeostasis monitored. As shown in Fig 6A, STZ administration (STZ/Sham) caused a drastic increase in blood glucose level while transplantation of 1,500 IEQ of normal islets (STZ/Nor‐Ctrl) largely reversed STZ‐induced hyperglycemia 1 week after transplantation and the effect was sustained for at least 4 weeks. On the other hand, transplantation of T2D islets transduced with AAV‐Ctrl (STZ/T2D‐Ctrl) modestly decreased the blood glucose level. Noticeably, transduction of T2D islets with AAV‐PAX6 (STZ/T2D‐PAX6) prior to transplantation further lowered the blood glucose level, indicating enhanced glycemic control by the transplanted islets *in vivo*. Besides, normal islet transplant remarkably ameliorated glucose intolerance compared to the sham mice. T2D‐Ctrl islet grafts tended to improve glucose tolerance slightly whereas T2D‐PAX6 islet grafts displayed enhanced efficacy (Fig 6B and C). Human insulin was detected in the circulation of mice with human islet transplant. Mice with T2D‐Ctrl islet grafts displayed a much lower circulating human insulin level compared to those with normal islet grafts, which was augmented by AAV‐PAX6 pre‐treatment (Fig 6D). The islet grafts were harvested for the assessment of graft survival. Immunofluorescence staining showed that the T2D‐Ctrl islet grafts exhibited a dampened insulin signal. ELISA measurement confirmed the lower insulin content while the T2D‐PAX6 islet grafts had increased insulin content (Fig 6E). Consistent with the findings in *ex vivo* isolated islets (Fig 5C), Ki67 signal was barely detected in T2D islet grafts but was still present in T2D islet graft with prior AAV‐PAX6 transduction (Appendix Fig S7A). On the other hand, the TUNEL assay showed that more apoptotic cells were detected in T2D‐Ctrl islet grafts, which could be reduced by AAV‐PAX6 transduction (Fig 6F). Previous studies have found that loss of transplanted islets can be detected in the first few days after transplantation (Biarnes *et al*, 2002; Eich *et al*, 2007), while the early graft function is closely associated with long‐term transplantation outcome (Vantyghem *et al*, 2009; Lam *et al*, 2022). We, therefore, harvested the graft‐bearing kidneys 1 week post‐transplant and found that PAX6 replenishment enhanced the early graft survival by reducing cell apoptosis (Appendix Fig S7B). These findings indicate that PAX6 replenishment in T2D islets promotes islet survival and results in better glycemic control after transplantation.

### Beta cell‐specific PAX6 overexpression enhances the potency of normal human islets and their glycemic control after transplantation into immunodeficient diabetic mice

**Figure 7:**
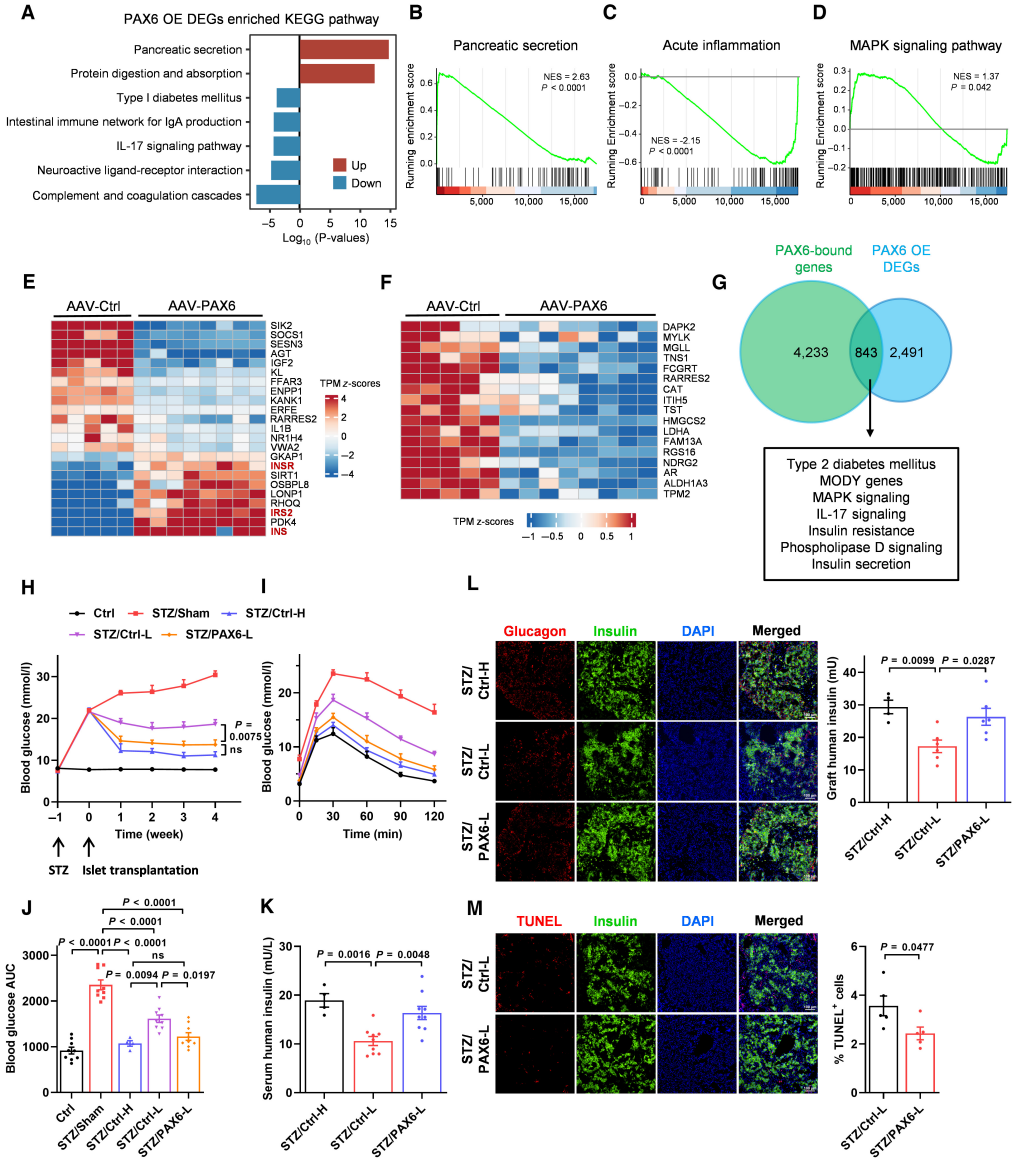
Beta cell‐specific PAX6 overexpression enhances the potency of normal human islets and their glycemic control after transplantation into immunodeficient diabetic mice ATop KEGG pathways enriched by DEGs in human islets with PAX6 overexpression compared with the control group. Up‐ or down‐regulated genes enriched pathways were represented in red and blue colors, respectively.B–DGSEA enrichment graphs of pancreatic secretion pathway, acute inflammatory pathway, and MAPK signaling pathway.E, FHeatmaps of DEGs showing (E) the increased expression of INS, INSR, and IRS2 genes and (F) the decreased expression of disallowed genes in human islets with PAX6 overexpression.GVenn diagram showing the top overlapping KEGG pathways between the PAX6‐bound genes (GSE87530) (Swisa *et al*, 2017) and the DEG list of our PAX6‐overexpressing islets (adjusted *P*‐value < 0.05; cutoff log_2_FC > 0.5).H–J(H) Fasting blood glucose, (I) glucose tolerance, and (J) glucose profiles calculated as AUC of control or STZ‐treated mice with normal human islet transplantation (*n* = 4–9).KSerum human insulin (*n* = 4–9).LRepresentative immunostaining showing insulin/glucagon signal and ELISA measurement of human insulin content in human islet grafts (*n* = 4–6).MRepresentative immunostaining and quantification showing insulin/TUNEL signal in human islet grafts (*n* = 5). Scale bar = 100 μm. Data information: Each *n* represents the measurement of a sample from distinct mice (H–M). Hypergeometric test was applied for the pathway enrichment analysis and the *P*‐values were adjusted by Benjamini–Hochberg correction method (A). Gene list ranked by their log_2_ fold changes were used for GSEA based on the Kolmogorov–Smirnov test. False discovery rate < 0.05 was set as significance threshold (B–D). One‐way ANOVA (H–L). Unpaired Student's *t*‐test (M). Data are means ± SEM. DEGs, differentially expressed genes; AUC, area under the curve; ns, nonsignificant. Source data are available online for this figure.

Loss of islet grafts and insufficient islet supply are the main obstacles impeding the success of islet transplantation and limiting its widespread application. To evaluate if *PAX6* gene transfer in islets isolated from healthy donors, which are normally used in clinical islet transplantation, would enhance islet potency and preserve islet graft post‐transplant, AAV‐PAX6 transduction was performed in normal human islets. Results showed that PAX6 overexpression in normal islets stimulated beta cell proliferation, increased GSIS and insulin content *ex vivo* (Appendix Fig S8A–C). RNA sequencing was performed to compare the transcriptomic profiles of PAX6‐overexpressing islets (AAV‐PAX6) with the control islets (AAV‐Ctrl). Principal component analysis indicated marked differences in the gene expression profile (Appendix Fig S8D). Up‐regulated genes were enriched in pancreatic secretion and protein metabolism pathways while down‐regulated genes were enriched in pathways involved in T1D and inflammation (Fig 7A–C). Gene set enrichment analysis (GSEA) also revealed that the mitogen‐activated protein kinase (MAPK) signaling, a critical signaling pathway involved in beta cell proliferation and survival, had significant enrichment in PAX6‐overexpressing islets (Fig 7D). Specifically, PAX6 overexpression induced the expression of key insulin signaling components (*INS*, *INSR*, and *IRS2*) (Fig 7E) and a number of genes involving in the regulation of DNA replication and cell proliferation (Appendix Fig S8E), which was corroborated with the increased beta cell proliferation, insulin content as well as the potentiation of insulin signaling in PAX6‐overexpressing islets. Consistent with the role of PAX6 in maintaining beta cell identity, a list of disallowed genes was found to be down‐regulated in PAX6‐overexpressing islets, which may also contribute to the enhanced potency of islets (Fig 7F). Moreover, by overlapping the differentially expressed genes (DEGs) in our dataset with PAX6‐bound genes identified in previously published chromatin immunoprecipitation (ChIP)‐sequencing data in EndoC‐βH2 cells (GSE87530) (Swisa *et al*, 2017), we found that the shared genes were highly enriched in several aspects or pathways including T2D, maturity‐onset diabetes of the young (MODY), MAPK signaling, IL‐17 signaling, insulin secretion and resistance, and phospholipase D signaling (Fig 7G), which are all closely related to beta cell function and diabetes. There were 4,233 genes bound by PAX6 with their expression unaltered by PAX6 overexpression. It is recognized that ChIP reveals the binding of transcription factors to thousands of genomic locations in the vicinity of both active and inactive regions. A number of weakly bound sites or the sites bound at low occupancy detected this way actually fail to drive gene transcription (Li *et al*, 2008; Fisher *et al*, 2012; Spivakov, 2014). On the other hand, there were 2,491 genes altered by PAX6 overexpression without PAX6 binding. This can be explained by the regulation of the expression or activity of other transcription factors or master regulators such as MAFA, PDX1, NKX6.1, CREB, and Akt by PAX6. These factors in turn regulate the expression of a bundle of effector genes. Nevertheless, these findings suggest that PAX6 functions as either a direct or indirect activator or repressor of genes in human beta cells that determines beta cell function and diabetes development. To evaluate if enhanced islet potency enables a reduction in transplanted islet mass, 2 doses of normal islets (1,500 and 1,000 IEQ) transduced with AAV‐Ctrl or AAV‐PAX6 were transplanted into STZ‐induced diabetic mice. Transplantation of a lower islet mass (STZ/Ctrl‐L) resulted in a modest improvement in blood glucose levels and glucose tolerance in the STZ‐treated mice compared to the transplantation of a higher islet mass (STZ/Ctrl‐H) (Fig 7H–J). Importantly, AAV‐PAX6 transduction (STZ/PAX6‐L) in the lower islet mass enhanced the graft function by exhibiting higher efficacy in controlling glycemia and glucose tolerance compared to AAV‐Ctrl transduction (STZ/Ctrl‐L), and matching the efficacy of the STZ/Ctrl‐H group (Fig 7H–J). Serum human insulin levels were also increased by AAV‐PAX6 pre‐treatment (Fig 7K). Consistently, islet grafts with prior AAV‐PAX6 transduction showed increased insulin content (Fig 7L) with more Ki67 signal detected (Appendix Fig S8F). Besides, TUNEL assay further revealed that PAX6 overexpression reduced the number of apoptotic cells in islet grafts 1 week (Appendix Fig S8G) and 4 weeks (Fig 7M) post‐transplant. Overall, these results demonstrate that PAX6 overexpression in human islets enhances islet potency, provides long‐term protection to islet grafts, and allows effective glycemic control even with reduced number of transplanted islets.

## Discussion

Both T1D and T2D manifest with loss of functional pancreatic beta cells. It has been reported that beta cell mass is reduced by 40–60% in T2D (Butler *et al*, 2003; Sun & Han, 2020) and by 70–97% in T1D (Campbell‐Thompson *et al*, 2016; Oram *et al*, 2019). Preventing beta cell loss or promoting beta cell regeneration is an attractive curative approach for diabetes. However, it is generally recognized that the capability of adult human beta cell replication or regeneration is very limited when compared to rodent beta cells (Kulkarni *et al*, 2012). Despite substantial advances in the development of anti‐diabetic therapies, there is still no clinical therapy proven to stimulate beta cell proliferation or preserve beta cell mass once beta cell failure has ensued (DeFronzo *et al*, 2015). Deciphering the network of beta cell intracellular signaling pathways that regulate cell survival can provide new insights to address this critical issue. PAX6 is a transcription factor recently found to regulate adult beta cell function (Swisa *et al*, 2017; So *et al*, 2021). However, whether PAX6 determines human beta cell survival as well as its therapeutic potential in beta cell preservation remains ambiguous. In the present study, we made several notable observations concerning the role of PAX6 in beta cell preservation and diabetes management. First, we provided *in vitro* and *ex vivo* evidence unraveling the essential role of PAX6 in maintaining beta cell function and survival through regulation of insulin and incretin signalings. Second, replenishment of PAX6 in beta cells under diabetic conditions preserves beta cells and alleviates glycemic perturbation *in vivo*. Third, PAX6 overexpression in human islets prior to transplantation promotes islet potency, preserves post‐transplant islet graft and enhances the capability of glycemic control in diabetic mice.

We investigated the effects of *PAX6* gene delivery in beta cell preservation using mouse models and human islets. There are several approaches to conduct gene delivery including clustered regularly interspaced short palindromic repeats (CRISPR), nanoparticle, and AAV. The CRISPR system is usually applied in stem cell‐derived beta cells while there is only one study demonstrating the use of CRISPR in human islets, which involves extensive manipulations including islet dispersion and reaggregation into pseudoislets (Bevacqua *et al*, 2021). There are also limited number of studies using nanoparticles (Wang & Moore, 2016; Melamed *et al*, 2023) to deliver genes into islets. Further investigation is warranted to confirm the efficiency and effects on islet physiology of these approaches. In contrast, AAV is the leading platform for gene delivery for the treatment of various human diseases as it provides long‐term gene modification without causing substantial adverse effects (Wang *et al*, 2019). In particular, AAV8 has been demonstrated to effectively transduce beta cells or islets *in vivo* and *in vitro* (Rehman *et al*, 2005; Wang *et al*, 2006; Xiao *et al*, 2018), and thus was adopted to overexpress PAX6 in beta cells. We first demonstrated the *in vivo* role of PAX6 in beta cell preservation using the STZ‐induced diabetic mouse model which is widely applied to mimic the severe beta cell loss in T1D. Our results show that PAX6 overexpression in beta cells stimulated beta cell proliferation and inhibited cell apoptosis. Elevated pancreatic and serum insulin levels in turn relieved glycemic perturbation. In addition, alterations in alpha cells were also observed. Alpha cell expansion led by STZ‐induced severe beta cell loss caused a dramatic increase in the alpha‐to‐beta cell ratio and the concomitant increases in pancreatic and serum glucagon levels. It has been reported that intra‐islet insulin and GABA released from beta cells act together to restrain alpha cell proliferation; while local insulin and GABA deficit caused by STZ‐mediated beta cell loss triggers alpha cell expansion (Feng *et al*, 2017). Importantly, PAX6 overexpression in beta cells stimulated beta cell proliferation, increased pancreatic insulin and GABA contents while inhibiting the expansion of alpha cells and the rise in glucagon levels. The simultaneous conservation of beta cells and restraint on alpha cell expansion preserved the islet architecture, maintained balanced insulin and glucagon levels, and thereby mediated the anti‐diabetic effect of AAV‐PAX6.

The role of PAX6 in beta cell preservation was further delineated in T2D using *db/db* mouse islets and primary human T2D islets, in which both displayed a diminished PAX6 expression and reduced beta cell survival but were reversed by PAX6 replenishment. Previous ChIP‐sequencing data (Swisa *et al*, 2017) reveal that PAX6 binds to genes related to the insulin signaling while PAX6 also controls the GLP‐1R and GIPR expression levels in rodent beta cells (Gosmain *et al*, 2012; Mitchell *et al*, 2017; Swisa *et al*, 2017). Both insulin and incretin signalings are known to critically regulate beta cell function and survival (Drucker, 2006; Leibiger *et al*, 2008) but whether PAX6 modulates these signaling events in human beta cells is yet to be validated. In this respect, we provide compelling evidence demonstrating the regulation of both insulin and incretin signalings by PAX6 in association with beta cell function and survival. PAX6 knockdown in EndoC‐βH1 cells resulted in a diminution of both insulin and incretin signaling transduction which was associated with suppressed cell survival. Notably, those phenomena were observed in EndoC‐βH1 cells under HGPA condition, *db/db* mouse and human T2D islets, while these changes could be rescued by PAX6 overexpression, suggesting that down‐regulation of PAX6 and the related survival signaling events under diabetic condition contribute to beta cell loss.

Our results may have important clinical implications. Incretin therapy is currently adopted in clinical practice to potentiate insulin secretion in diabetic patients. However, clinical studies have reported that diabetic patients are less responsive to the current incretin‐based treatments (Krarup *et al*, 1987; Kjems *et al*, 2003). Defects in insulin biosynthesis or exocytotic machinery limit the responsiveness to incretin administration. On top of that, suppressed GLP‐1R and GIPR expression in human T2D islets as revealed by the current and other studies (Lyssenko *et al*, 2011; Taneera *et al*, 2012; Guo *et al*, 2013) is expected to lead directly to incretin resistance and lower the efficacy of treatments. Our results suggested that PAX6 is essential to sensitize beta cells to incretins; while the maintenance of insulin content and insulin exocytotic machinery by *PAX6* gene transfer (So *et al*, 2021) might orchestrate to enhance the overall efficacy of incretin treatments when applied in combination therapy.

Beta cell dedifferentiation has recently been considered as an early feature of T2D where dedifferentiated beta cells revert to progenitor‐like cells or transdifferentiate into other pancreatic endocrine cell types, leading to the loss of beta cell mass (Spijker *et al*, 2015). MAFA, NKX6.1, and PDX1 are the key beta cell transcription factors regulating beta cell function, identity and proliferation (Gao *et al*, 2014; Aigha & Abdelalim, 2020; Nishimura *et al*, 2022) while repression of these transcription factors mediates the loss of beta cell identity and progression of islet failure in T2D (Guo *et al*, 2013). We confirmed the loss of beta cell identity in islets under diabetic conditions by showing the significantly reduced expression levels of MAFA, NKX6.1, and PDX1 in both *db/db* mouse and human T2D islets. Besides, the increased frequency of insulin and glucagon coexpressing cells was another piece of evidence indicating the loss of beta cell identity and adoption of the alpha cell fate, which likely contributes to hyperglucagonemia as observed in both *db/db* mice and T2D subjects. PAX6 overexpression in beta cells helped maintain beta cell identity by restoring the expression of those key transcription factors and decreasing the amount of insulin and glucagon coexpressing cells under diabetic conditions. Apart from the maintenance of beta cell identity, MAFA, PDX1, and NKX6.1 have been revealed to regulate cell proliferation (Gao *et al*, 2014; Aigha & Abdelalim, 2020; Nishimura *et al*, 2022). While some of the existing therapies such as insulin, SGLT inhibitor, and TZD lower blood glucose levels in *db/db* mice, beta cell dedifferentiation is not rescued (Ishida *et al*, 2017). Our results show that PAX6 replenishment in diabetic islets not only improves hyperglycemia but also conserves beta cells by enhancing survival and maintaining identity through interactions with other key beta cell transcription factors. This highlights PAX6 as a potential therapeutic target in beta cell regenerative medicine.

Islet transplantation is a beta cell replacement therapy adopted in clinical practice. It is a promising measure for insulin‐dependent diabetes which provides long‐term glycemic control for type 1 and late‐stage type 2 diabetics with minimal risk of hypoglycemic unawareness. However, despite tremendous progress in the procedures of islet isolation and transplantation, the success and utility of this modality of treatment is limited due to islet cell exhaustion and cell death following transplantation, limited supply of cadaveric islets, and the need for lifelong administration of immunosuppressive drugs (Shapiro *et al*, 2017; Gamble *et al*, 2018). In general, large numbers of islets from multiple donors are required in a single transplantation to compensate for the significant islet loss after transplantation. This profound loss of islet graft was observed in both immunodeficient and syngeneic transplant models (Carlsson *et al*, 2001, 2002). On the other hand, islet encapsulation, a strategy showing potential to overcome immune rejection, however, does not support long‐term graft survival in clinical trials (Tuch *et al*, 2009; Kuwabara *et al*, 2022). These observations indicate that nonimmune‐mediated physiological stress, including the exposure of engrafted islets to a toxic milieu of hyperglycemia, cytokines, and immunosuppressants as well as the prolonged hypoxia during the revascularization process, is a major factor that negatively influences islet graft survival (Narang & Mahato, 2006). For all these reasons, a substantial proportion of the islet graft becomes damaged and is lost due to apoptosis. Of note, clinical studies suggest that islet graft function plays a predominant role to determine the long‐term transplantation outcome (Vantyghem *et al*, 2009; Lam *et al*, 2022). Therefore, significant progress remains necessary to enhance islet potency and to reduce the early loss of islets after transplantation, which could have a major impact on the long‐term success of islet transplantation and the number of diabetic patients that could benefit from the procedure. In this regard, our transplant study using T2D islets demonstrated that PAX6 replenishment potentiated the functionally compromised islets and provided long‐lasting protective effect on graft survival, which in turn enhanced the capability of glycemic control. The use of induced pluripotent stem cells (iPSCs)‐derived beta cells in transplantation is under active investigation as they serve as a potentially unlimited source of beta cells. However, they are not yet suitable for large‐scale production of patient‐specific iPSCs‐beta cells for transplantation since individuals require a large number of individual pancreatic islets. In addition, unlike whole pancreas or islet transplantation, iPSC‐derived beta cells do not replace the lost function of alpha cells. Deficiency in glucagon production precipitates hypoglycemia unawareness. Until these challenges are addressed, transplantation of islets isolated from healthy donors remains the preferred approach in clinical practice. However, the procedures of islet isolation and *ex vivo* culture *per se* induce islet cell inflammation and lead to cell dedifferentiation, reduced function, and survival (Negi *et al*, 2012; Teo *et al*, 2018), which lower the transplant efficacy and increase the requirement of transplanted islet mass. Notably, we showed that AAV‐PAX6 transduction in normal human islets stimulated beta cell proliferation, maintained beta cell identity, suppressed inflammation and enhanced GSIS, in which the up‐regulation of MAPK (Stewart *et al*, 2015) and insulin signaling (Leibiger *et al*, 2008; Darden *et al*, 2022) might be involved. By adopting a suboptimal transplant model, we demonstrated that AAV‐PAX6 transduction prior to transplantation enhanced post‐transplant islet graft survival which compensated for the reduced mass of engrafted islets and therefore displayed a comparable efficacy with the transplantation of a higher islet mass. In other words, the results suggest that our *PAX6* gene delivery approach enhances islet potency, provides long‐term graft preservation, and enables the reduction of the mass of islets required to reverse hyperglycemia in each transplantation, which therefore potentially enables more diabetic patients to receive islet transplantation.

In summary, our study provides evidence from multiple, complementary human systems that advances the understanding of PAX6 as a critical regulator of beta cell function, survival, and identity. Augmentation of PAX6 expression or activity thus represents a promising approach to reverse beta cell loss in diabetes. In particular, we demonstrate that *PAX6* gene transfer might overcome two of the major barriers of islet transplantation: post‐transplant islet graft loss and scarcity of islet donors. Given the prevalence of diabetes worldwide, our findings provide grounds to support the future clinical translation of *PAX6* gene delivery to overcome beta cell failure in diabetes.

## Materials and Methods

### Cell culture

Human EndoC‐βH1 cell line (Univercell‐Biosolutions, Toulouse, France) was cultured as previously described (Ravassard *et al*, 2011). Cells were maintained under normal condition (5.6 mmol/l glucose) or treated with HGPA (25 mmol/l D‐glucose and 0.3 mmol/l palmitic acid) at 37°C in a humidified 5% CO_2_ condition. The cell line was regularly tested to ensure it was negative for mycoplasma contamination.

### Lentivirus‐mediated gene overexpression and knockdown

PAX6‐overexpressing construct was generated by subcloning the coding sequence expressing human *PAX6* (OriGene, Rockville, MD, USA) into the lentiviral vector (Addgene, Cambridge, MA, USA). PAX6 knockdown construct was generated by subcloning a short hairpin RNA (shRNA) sequence targeting human *PAX6* (Appendix Table S1) into the lentiviral pLKO.1 vector (Addgene). A non‐targeting shRNA sequence was used as control. All constructs were validated by DNA sequencing. Viral particles were generated by transient transfection of HEK‐293FT cells (American Type Culture Collection, Manassas, VA, USA) with the lentiviral plasmids and helper plasmids (pRSV‐Rev, pCMV‐VSVG, and pMDLg/pRRE; Addgene) using lipofectamine 3000 reagent (Thermo Fisher Scientific, Rockford, IL, USA). The cell supernatant was collected, concentrated, and titrated at 48 h after transfection. EndoC‐βH1 cells were infected with viral particles and subjected to antibiotic selection to generate stable cell lines.

### Animal models

Ten to twelve‐week‐old male genetically diabetic C57BL/KsJ‐db (*db/db*) and their lean heterozygote littermates (*db*/+); male C57BL/6J and immunodeficient NSG mice were obtained from the Jackson Laboratory (Bar Harbor, ME, USA). A single high dose of STZ (Sigma‐Aldrich, St. Louis, MO, USA) was intraperitoneally injected into C57BL/6J (150 mg/kg) or NSG (120 mg/kg) mice to render hyperglycemia. Mice were fed *ad libitum* and maintained with constant ambient temperature and a 12‐h light/dark cycle. All experimental procedures involving animals were approved by the Institutional Animal Care and Use Committee at the Agency for Science, Technology and Research (A*STAR; reference no. 191479).

### Administration of AAV

Normal or STZ‐induced diabetic C57BL/6J mice with blood glucose levels exceeding 14 mmol/l for 2 consecutive times, as well as *db/db* and their lean heterozygote littermates *db*/+ mice were injected with AAV. The AAV8 carrying PAX6‐flag (AAV‐PAX6) or GFP (AAV‐Ctrl) driven by the rat insulin promoter was purchased from VectorBuilder. AAV‐PAX6 or AAV‐Ctrl was injected intraperitoneally into mice at the dose of 1 × 10^11^ viral genomes (vg) per mouse. Mice were observed for the indicated periods of time after AAV injection.

### *In vivo* glucose homeostasis

Glucose tolerance was assessed by intraperitoneal glucose tolerance test (IPGTT). After 16 h fasting, mice were given 1 g/kg body weight of glucose (Sigma‐Aldrich) by intraperitoneal injection. Blood glucose levels were measured at the indicated time points. Serum insulin (Mercodia AB, Uppsala, Sweden) and glucagon (R&D Systems, Minneapolis, MN) levels were measured by enzyme‐linked immunosorbent assay (ELISA) kits.

### Isolation and culture of mouse pancreatic islets

Intact pancreatic islets were isolated from mice as previously described (So *et al*, 2021). Briefly, mouse pancreas was given collagenase P (Roche, Mannheim, Germany) in Hanks' balanced salt solution (Gibco, Grand Island, NY, USA) through intra‐ductal injection. The pancreas was digested in 37°C. After washing and gradient centrifugation, the islets were handpicked under a stereomicroscope. Isolated islets were further dispersed into single cells by trypsin for cAMP measurement. Whole islets or dispersed cells were cultured in RPMI‐1640 medium (Thermo Fisher Scientific) supplemented with 10% (vol/vol) fetal bovine serum (FBS; Gibco) and 1% (vol/vol) penicillin and streptomycin (Thermo Fisher Scientific).

### Human islet culture and transduction

Primary human islets from seven normal and five T2D donors (Appendix Table S2) were obtained from Prodo Laboratories, Inc. (Aliso Viejo, CA, USA). Human islets were cultured in PIM(S) complete media (Prodo Labs) and transduced with AAV‐Ctrl or AAV‐PAX6 at a dose of 20,000 vg per islet cell. All procedures involving human tissues were approved by A*STAR Institutional Review Board (reference no. 2019‐021). Informed consent was obtained from each donor or the donor's next of kin and that the experiments conformed to the principles set out in the WMA Declaration of Helsinki and the Department of Health and Human Services Belmont Report.

### RNA sequencing and analysis

Human islets with AAV‐Ctrl or AAV‐PAX6 transduction were harvested with total RNA extracted using TRIzol Reagent (Thermo Fisher Scientific). RNA samples were submitted to Omics Drive (Singapore) for RNA sequencing analysis. Briefly, 1 μg total RNA from each sample with a RNA integrity number value above 7 was used for library preparations. The poly(A) mRNA isolation, fragmentation and priming were performed using the Poly(A) mRNA Magnetic Isolation Module kit. First‐ and second‐strand cDNA was synthesized using ProtoScript II Reverse Transcriptase and Second Strand Synthesis Enzyme Mix, respectively. The double‐stranded cDNA was purified using beads followed by treatment with End Prep Enzyme Mix. Size selection of adaptor‐ligated DNA was performed using beads with fragments of ~400 bp (with an approximate insert size of 300 bp) were recovered. Each sample was amplified by polymerase chain reaction (PCR) using P5 and P7 primers. Subsequently, PCR products were cleaned up using beads, validated using a Qsep100 (Bioptic, Taiwan), and quantified using a Qubit 3.0 Fluorometer (Invitrogen, Carlsbad, CA, USA). Libraries with different indices were multiplexed and loaded on an HiSeq/NovaSeq instrument (Illumina, San Diego, CA, USA) according to the manufacturer's instructions. Sequencing was performed using a 2 × 150‐bp paired‐end configuration. Image analysis and base calling were conducted by HiSeq/NovaSeq Control Software + OLB + GAPipeline‐1.6 (Illumina). After sequencing, the reads were analyzed on Partek Flow software (Partek Inc., St. Louis, MO, USA). In essence, the reads were mapped to the hg38 reference genome using STAR aligner. The aligned reads were quantified and annotated based on RefSeq transcripts (version 99). Differential gene expression analysis was performed using DESeq2 algorithm, genes with Benjamini–Hochberg (BH) adjusted *P*‐value < 0.05 and absolute log_2_ fold changes (FC) ≥ 1 were identified as DEGs. Overrepresentation enrichment analysis and GSEA was performed using clusterProfiler, based on Kyoto Encyclopedia of Genes and Genomes Pathways (*Homo sapiens*). 3,334 DEGs based on adjusted *P*‐value cutoff were used for overlapping with PAX6‐bound genes, to ensure the comparable gene numbers.

### Islet transplantation

NOD scid gamma mice with blood glucose levels exceeding 20 mmol/l for 2 consecutive times were used as recipients of islet transplantation. Under anesthesia, the left kidney of the recipient mouse was exposed through an incision. Around 1,000 or 1,500 IEQ human islets or saline were injected under the kidney capsule. The capsulotomy was cauterized and peritoneum was sutured back.

### Quantitative real‐time PCR

Total RNA from EndoC‐βH1 cells or pancreatic islets was extracted using TRIzol reagent and subjected to reverse transcription using High‐Capacity cDNA Reverse Transcription Kits (Applied Biosystems, Foster City, CA, USA). Gene expression was quantified by real‐time PCR using SYBR™ Green PCR Master Mix (Applied Biosystems) in an i‐Cycler Thermal Cycler (Applied Biosystems). Relative gene expression was normalized relative to beta actin and analyzed using the 2(^−ΔΔCt^) method. The sequences of primers used are listed in Appendix Table S1.

### Western blotting

Proteins from cell or tissue samples were extracted using the RIPA Lysis and Extraction Buffer (Thermo Fisher Scientific). Proteins were separated by SDS–PAGE and transferred to nitrocellulose membrane by iBlot™ 2 Gel Transfer Device (Thermo Fisher Scientific). After blocking, the membrane was incubated with antibodies against the following proteins: GAPDH (1/1,000, sc‐32233, Santa Cruz Biotechnology Inc., Santa Cruz, CA, USA), PAX6 (1/1,000, ab197768, Abcam), IRβ (1/1,000, 3025, Cell Signaling Technology, Danvers, MA, USA), IRS2 (1/1,000, 3089, Cell Signaling Technology), PI3K85 (1/1,000, 4292, Cell Signaling Technology), PI3K110α (1/1,000, 4255, Cell Signaling Technology), GLP1R (1/1,000, NLS1205, Novus Biologicals, Littleton, CO, USA), GIPR (1/1,000, ab198694 and 1/1,000, ab136266, Abcam), phospho‐Akt (1/1,000, 4060, Cell Signaling Technology), Akt (1/1,000, 4685, Cell Signaling Technology), phospho‐CREB (1/1,000, 9198, Cell Signaling Technology), CREB (1/1,000, 9197, Cell Signaling Technology), ALDH1A3 (1/1,000, NBP2‐15339, Novus Biologicals), Flag (1/1,000, F1804, Sigma‐Aldrich). After washing, the membrane was incubated with appropriate IR‐Dye labeled secondary antibodies (1/10,000, LI‐COR Biosciences, Lincoln, NE, USA). Labeled protein bands were visualized and quantified by Odyssey® CLx Imager (LI‐COR Biosciences).

### Insulin secretion assay

Cells or size‐matched islets were pre‐incubated in Krebs–Ringer bicarbonate buffer (KRBB; supplemented with 10 mmol/l HEPES and 2 mg/ml BSA) with 2.5 mmol/l glucose for 1 h followed by an additional 1 h incubation in KRBB containing 2.5 mmol/l or 16.7 mmol/l glucose. Buffer was collected to measure insulin release by ELISA kits (Mercodia AB).

### Determination of cell proliferation and apoptosis

Cell proliferation was determined by measuring bromodeoxyuridine (BrdU) incorporation. Cells were labeled with BrdU for 2 h and DNA synthesis was measured by a cell proliferation assay kit (Roche). Cell apoptosis was measured with a cell death detection ELISA plus kit (Roche). All procedures were performed according to the manufacturer's instructions.

### Measurement of intracellular cAMP level

EndoC‐βH1 cells were stimulated with 10 nmol/l exendin‐4 or GIP for 15 min. The intracellular cAMP levels were measured using the cAMP immunoassay kit (Sigma‐Aldrich) according to manufacturers' instructions.

### Immunofluorescence staining

Isolated islets, whole pancreas or graft‐bearing kidney were embedded and frozen. Fixed cryostat sections were incubated with antibodies against the following proteins: flag (1/500, F1804, Sigma‐Aldrich), insulin (1/1,000, ab181547, Abcam), glucagon (1/1,000, ab92517, Abcam), Ki67 (1/1,000, ab15580, Abcam), MAFA (1/500, ab26405, Abcam), PDX1 (1/500, ab134150, Abcam), or NKX6.1 (1/200, F55A12, Developmental Studies Hybridoma Bank, the University of Iowa, IA USA). For terminal deoxynucleotidyl transferase‐mediated dUTP nick‐end labeling (TUNEL) assay, an *in situ* cell death detection kit (Roche) was used before incubation with anti‐insulin antibody. After washing, slides were probed with Alexa Fluor dye‐conjugated secondary antibodies (1/2,000, Thermo Fisher Scientific) and were mounted with antifade mountant with DAPI (Thermo Fisher Scientific). Images were acquired by a Ti‐E fluorescence microscope (Nikon, Tokyo, Japan) and quantified by Nikon NIS‐Elements (Nikon). For normal and STZ‐treated mouse pancreas sections, more than 10 pancreas sections containing over 50 islets were quantified for each mouse and 5–6 mice were included for each group. For *db/db* mouse and human islet sections, more than 5 sections containing over 50 islets were quantified for each batch of islets and 5–6 batches of islets were included for each group. More than 4,000 beta cells per mouse or batch of islets were counted. For islet graft‐bearing kidney sections, more than 5 sections were quantified for each mouse and 4–6 mice were included for each group. For beta and alpha cell mass measurement, at least 10 evenly 200 μm apart sections throughout the whole pancreas were stained with insulin or glucagon and DAPI with total pancreatic and insulin or glucagon positive areas of each section measured. Beta or alpha cell mass was calculated by multiplying the ratio of total insulin or glucagon positive area to total pancreatic area with the pancreas weight.

### Statistics

Animals were randomized into different treatment groups. No mice were excluded from all the experiments. No blinding was assessed. Statistical analyses were performed using GraphPad Prism version 8. All data sets were tested for normality of distribution using the Shapiro–Wilk test. Comparisons between two groups were analyzed using unpaired Student's *t*‐test or Mann–Whitney test. Multiple groups were compared using one‐way or two‐way analysis of variance (ANOVA) followed by Tukey's *post hoc* test or Kruskal–Wallis test with Dunn's *post hoc* test. Data were presented as means ± standard errors (SEMs). *P*‐values less than 0.05 were considered statistically significant.

## Author contributions

**Wing Yan So:** Conceptualization; data curation; formal analysis; supervision; funding acquisition; validation; investigation; methodology; writing – original draft; writing – review and editing. **Yilie Liao:** Formal analysis; writing – review and editing. **Wai Nam Liu:** Formal analysis; writing – review and editing. **Guy A Rutter:** Conceptualization; writing – review and editing. **Weiping Han:** Conceptualization; resources; supervision; funding acquisition; validation; investigation; project administration; writing – review and editing.

## Disclosure and competing interests statement

The authors declare that they have no conflict of interest.

## Supporting information



Appendix

Source Data for Figure 1

Source Data for Figure 2

Source Data for Figure 3

Source Data for Figure 4

Source Data for Figure 5

Source Data for Figure 6

Source Data for Figure 7

## Data Availability

The gene expression profile reported in this paper has been deposited at the Gene Expression Omnibus (GEO) database (GSE242338 (http://www.ncbi.nlm.nih.gov/geo/query/acc.cgi?acc=GSE242338)).
