# Supplementary material for: Paired box 6 gene delivery preserves beta cells and improves islet transplantation efficacy
Source: EMBO Mol Med. 2023 Nov 7;15(12):e17928. doi: 10.15252/emmm.202317928 (PMC10701606; doi:10.15252/emmm.202317928)
Supplement: Supplementary file 1 — Appendix [file EMMM-15-e17928-s005.pdf]

Table of content

Appendix Figure S1……………………………………………………………………2

Appendix Figure S2……………………………………………………………………4

Appendix Figure S3……………………………………………………………………6

Appendix Figure S4……………………………………………………………………8

Appendix Figure S5…………………………………………………………………..10

Appendix Figure S6…………………………………………………………………..12

Appendix Figure S7…………………………………………………………………..14

Appendix Figure S8…………………………………………………………………..15

Appendix Table S1……………………………………………………………………..17

Appendix Table S2……………………………………………………………………..18

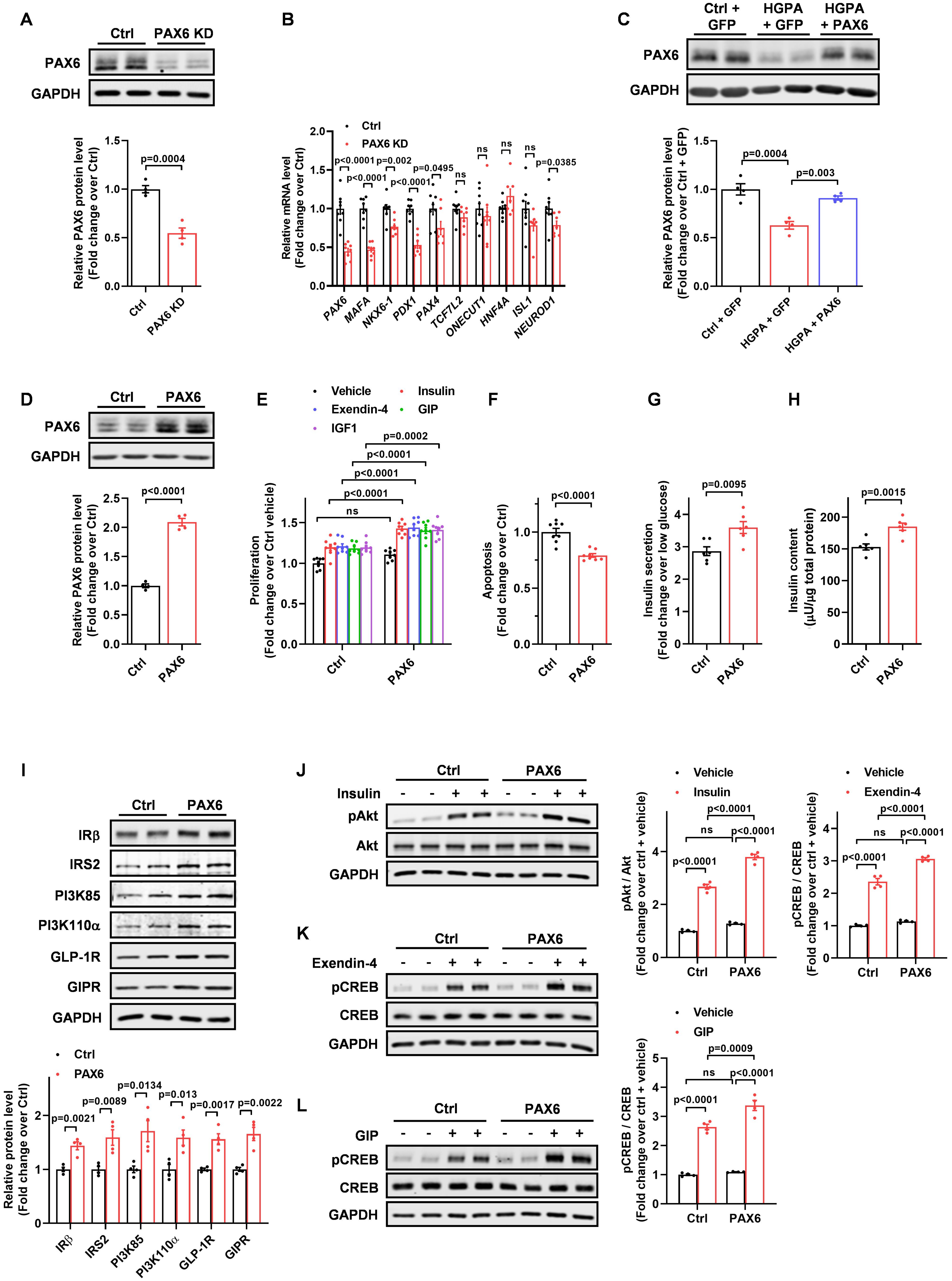
A
Ctrl
PAX6 KD
PAX6
GAPDH
Relative PAX6 protein level (Fold change over Ctrl)
p=0.0004
B
Ctrl
PAX6 KD
Relative mRNA level (Fold change over Ctrl)
p<0.0001
p<0.0001
p=0.002
p<0.0001
p=0.0495
ns
ns
ns
ns
p=0.0385
PAX6
MAFA
NKX6-1
PDX1
PAX4
TCF7L2
ONECUT1
HNF4A
ISL1
NEUROD1
C
Ctrl + GFP
HGPA + GFP
HGPA + PAX6
Relative PAX6 protein level (Fold change over Ctrl + GFP)
p=0.0004
p=0.003
D
Ctrl
PAX6
Relative PAX6 protein level (Fold change over Ctrl)
p<0.0001
E
Vehicle
Insulin
Exendin-4
GIP
IGF1
Proliferation (Fold change over Ctrl vehicle)
p=0.0002
p<0.0001
p<0.0001
p<0.0001
ns
F
Apoptosis (Fold change over Ctrl)
p<0.0001
G
Insulin secretion (Fold change over low glucose)
p=0.0095
H
Insulin content (μU/μg total protein)
p=0.0015
I
IRβ
IRS2
PI3K85
PI3K110α
GLP-1R
GIPR
GAPDH
Relative protein level (Fold change over Ctrl)
p=0.0021
p=0.0089
p=0.0134
p=0.013
p=0.0017
p=0.0022
J
Insulin
pAkt
Akt
pAkt / Akt (Fold change over ctrl + vehicle)
p<0.0001
ns
p<0.0001
p<0.0001
K
Exendin-4
pCREB
CREB
pCREB / CREB (Fold change over ctrl + vehicle)
p<0.0001
ns
p<0.0001
p<0.0001
L
GIP
p=0.0009
ns
p<0.0001
p<0.0001

Appendix figure S1. Effects of PAX6 knockdown and overexpression on EndoC-βH1 cells.

A. Protein expression of PAX6 in EndoC-βH1 cells with PAX6 knockdown (*n* = 4).

B. *PAX6*, *MAFA*, *NKX6.1*, *PDX1*, *PAX4*, *TCF7L2*, *ONECUT1*, *HNF4A*, *ISL1* and *NEUROD1* mRNA expression in EndoC-βH1 cells (*n* = 7 to 9).

C. Protein expression of PAX6 in EndoC-βH1 cells with PAX6 overexpression under normal and HGPA conditions (*n* = 4).

D. Protein expression of PAX6 in EndoC-βH1 cells with PAX6 overexpression under normal condition (*n* = 4).

E. EndoC-βH1 cells with PAX6 overexpression were subjected to cell proliferation measurement indexed by BrdU labelling after 72-hour treatment with insulin (100 nM), Exendin-4 (10 nM), GIP (10 nM) or IGF1 (50 ng/ml) (*n* = 8).

F-H. (F) Cell apoptosis (n = 8), (G) GSIS (*n* = 6) and (H) insulin content (*n* = 6) were measured in control and PAX6-overexpressing cells.

I. Protein expression of insulin and incretin signaling components in cells with PAX6 overexpression (*n* = 4).

J. Phosphorylated and total Akt abundance in cells with PAX6 overexpression were measured after 15-min insulin (100 nM) stimulation (*n* = 4).

K, L. Phosphorylated and total CREB abundance in cells with PAX6 overexpression were measured after 15-min (K) Exendin-4 (10 nM) or (L) GIP (10 nM) stimulation (*n* = 4).

Data information: Each *n* represents an independent biological replicate (A-L). Unpaired Student's *t* test (A, D, F-I). Unpaired Student's *t* test and Mann-Whitney test (B). One-way ANOVA (C). Two-way ANOVA (E, J-L). Data are means ± SEM. ns, nonsignificant.

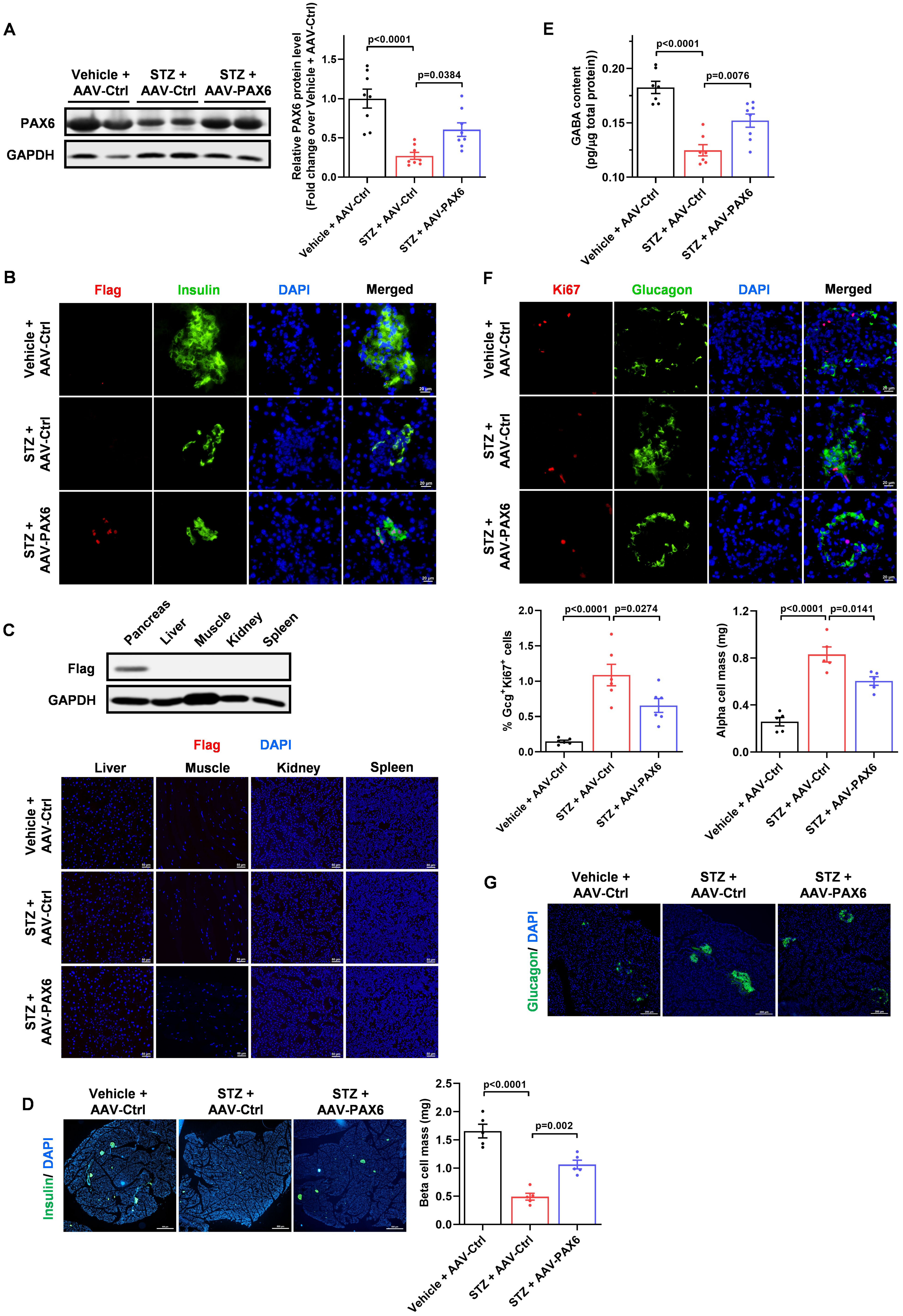
A
Vehicle + AAV-Ctrl
STZ + AAV-Ctrl
STZ + AAV-PAX6
PAX6
GAPDH
Relative PAX6 protein level (Fold change over Vehicle + AAV-Ctrl)
p<0.0001
p=0.0384
E
GABA content (pg/µg total protein)
p<0.0001
p=0.0076
B
Flag
Insulin
DAPI
Merged
F
Ki67
Glucagon
DAPI
Merged
C
Pancreas
Liver
Muscle
Kidney
Spleen
Flag
GAPDH
Flag
DAPI
Liver
Muscle
Kidney
Spleen
% Gcg+Ki67+ cells
p<0.0001
p=0.0274
Alpha cell mass (mg)
p<0.0001
p=0.0141
G
Glucagon/ DAPI
D
Insulin/ DAPI
Beta cell mass (mg)
p<0.0001
p=0.002

Appendix figure S2. Effects of PAX6 overexpression on pancreas of STZ-induced diabetic mice.

A. Protein expression of PAX6 in pancreas of STZ-induced diabetic mice with AAV injection (n = 8).

B. Representative immunostaining of mouse pancreas labelled for flag (red), insulin (green) and DAPI (blue) with AAV injection. Scale bar = 20 μm.

C. Western blotting showing the absence of flag tag in other mouse organs and representative immunostaining of mouse liver, muscle, kidney and spleen labelled for flag (red) and DAPI (blue). Scale bar = 50 μm.

D. Representative immunostaining and beta cell mass measurement of mouse pancreas labelled for insulin (green) and DAPI (blue). Scale bar = 500 μm.

E. GABA content was expressed as absolute amount (pg/μg total protein) (n = 7 to 8).

F. Representative immunostaining and quantification of pancreas from control or STZ-treated mice with AAV injection labelled for Ki67 (red), glucagon (green) and DAPI (blue) (n = 6). Scale bar = 20 μm.

G. Representative immunostaining and alpha cell mass measurement of mouse pancreas labelled for glucagon (green) and DAPI (blue). Scale bar = 200 μm.

Data information: Each n represents the measurement of a sample from distinct mice (A-G). One-way ANOVA (A, D-G). Data are means ± SEM.

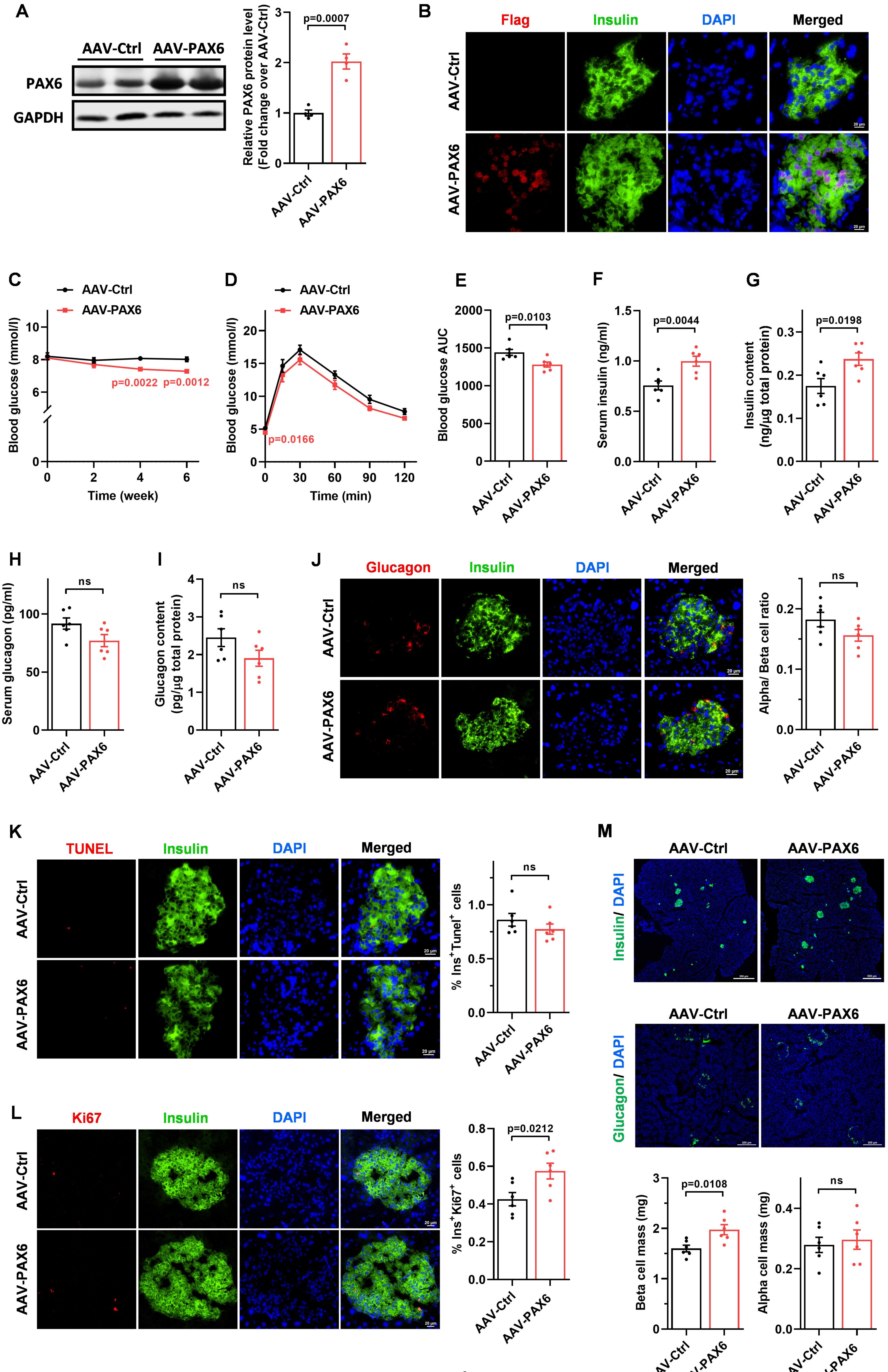
A
AAV-Ctrl
AAV-PAX6
PAX6
GAPDH
Relative PAX6 protein level
(Fold change over AAV-Ctrl)
p=0.0007
B
Flag
Insulin
DAPI
Merged
C
Blood glucose (mmol/l)
Time (week)
p=0.0022
p=0.0012
D
Time (min)
p=0.0166
E
Blood glucose AUC
p=0.0103
F
Serum insulin (ng/ml)
p=0.0044
G
Insulin content
(ng/μg total protein)
p=0.0198
H
Serum glucagon (pg/ml)
ns
I
Glucagon content
(pg/μg total protein)
J
Glucagon
Alpha/ Beta cell ratio
K
TUNEL
% Ins+Tunel+ cells
L
Ki67
% Ins+Ki67+ cells
p=0.0212
M
Insulin/ DAPI
Glucagon/ DAPI
Beta cell mass (mg)
p=0.0108
Alpha cell mass (mg)

Appendix figure S3. Effects of PAX6 overexpression on pancreas of normal C57BL/6J mice.

A. Protein expression of PAX6 in pancreas of normal mice with AAV injection (*n* = 4).

B. Representative immunostaining of mouse pancreas labelled for flag (red), insulin (green) and DAPI (blue) with AAV injection. Scale bar = 20 μm.

C, D. (C) Fasting blood glucose and (D) glucose tolerance of mice with AAV injection (*n* = 6).

E. Glucose profiles calculated as AUC (*n* = 6).

F-I. (F) Serum insulin, (G) pancreatic insulin content, (H) serum glucagon and (I) pancreatic glucagon content of mice with AAV injection (*n* = 6).

J-L. Representative immunostaining and quantification showing (J) alpha-to-beta cell ratio, (K) insulin/ TUNEL signal, (L) insulin/ Ki67 signal in pancreatic islets of mice with AAV injection (*n* = 6). Scale bar = 20 μm.

M. Representative immunostaining and beta and alpha cell mass measurement of mouse pancreas labelled for insulin or glucagon (green) and DAPI (blue). Scale bar = 500 μm or 200 μm.

Data information: Each *n* represents the measurement of a sample from distinct mice (A-M). Unpaired Student's *t* test (A, E-M). Unpaired Student's *t* test and Mann-Whitney test (C-D). Data are means ± SEM. AUC, area under the curve; ns, nonsignificant.

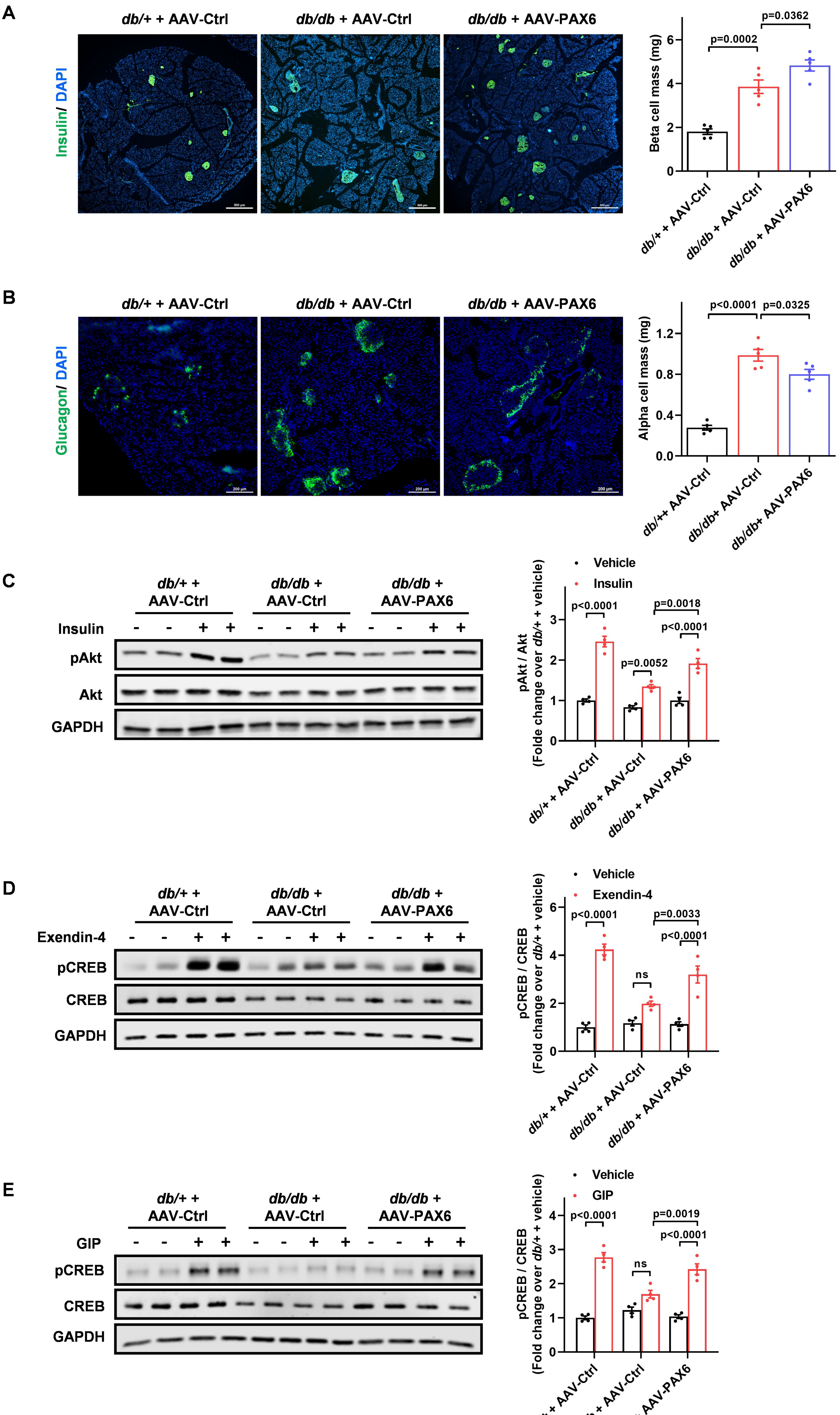
A
db/+ + AAV-Ctrl
db/db + AAV-Ctrl
db/db + AAV-PAX6
Insulin/ DAPI
Beta cell mass (mg)
p=0.0002
p=0.0362
B
db/+ + AAV-Ctrl
db/db + AAV-Ctrl
db/db + AAV-PAX6
Glucagon/ DAPI
Alpha cell mass (mg)
p<0.0001
p=0.0325
db/++ AAV-Ctrl
db/db+ AAV-Ctrl
db/db+ AAV-PAX6
C
db/+ + AAV-Ctrl
db/db + AAV-Ctrl
db/db + AAV-PAX6
Insulin
pAkt
Akt
GAPDH
Vehicle
Insulin
pAkt / Akt (Folde change over db/+ + vehicle)
p<0.0001
p=0.0052
p=0.0018
p<0.0001
D
db/+ + AAV-Ctrl
db/db + AAV-Ctrl
db/db + AAV-PAX6
Exendin-4
pCREB
CREB
GAPDH
Vehicle
Exendin-4
pCREB / CREB (Fold change over db/+ + vehicle)
p<0.0001
ns
p=0.0033
p<0.0001
E
db/+ + AAV-Ctrl
db/db + AAV-Ctrl
db/db + AAV-PAX6
GIP
pCREB
CREB
GAPDH
Vehicle
GIP
pCREB / CREB (Fold change over db/+ + vehicle)
p<0.0001
ns
p=0.0019
p<0.0001

Appendix figure S4. Effects of PAX6 replenishment on *db/db* mouse islets.

A. Representative immunostaining and beta cell mass measurement of mouse pancreas labelled for insulin (green) and DAPI (blue) ($n = 5$). Scale bar = 500 μm.

B. Representative immunostaining and alpha cell mass measurement of mouse pancreas labelled for glucagon (green) and DAPI (blue) ($n = 5$). Scale bar = 200 μm.

C. Phosphorylated and total Akt in isolated islets of *db/+* and *db/db* mice after 15-min insulin (100 nM) stimulation ($n = 4$).

D, E. Phosphorylated and total CREB in isolated islets of *db/+* and *db/db* mice after 15-min (D) Exendin-4 (10 nM) or (E) GIP (10 nM) stimulation ($n = 4$).

Data information: Each *n* represents the measurement of a sample from distinct mice (A-E). One-way ANOVA (A, B). Two-way ANOVA (C-E). Data are means ± SEM. ns, nonsignificant.

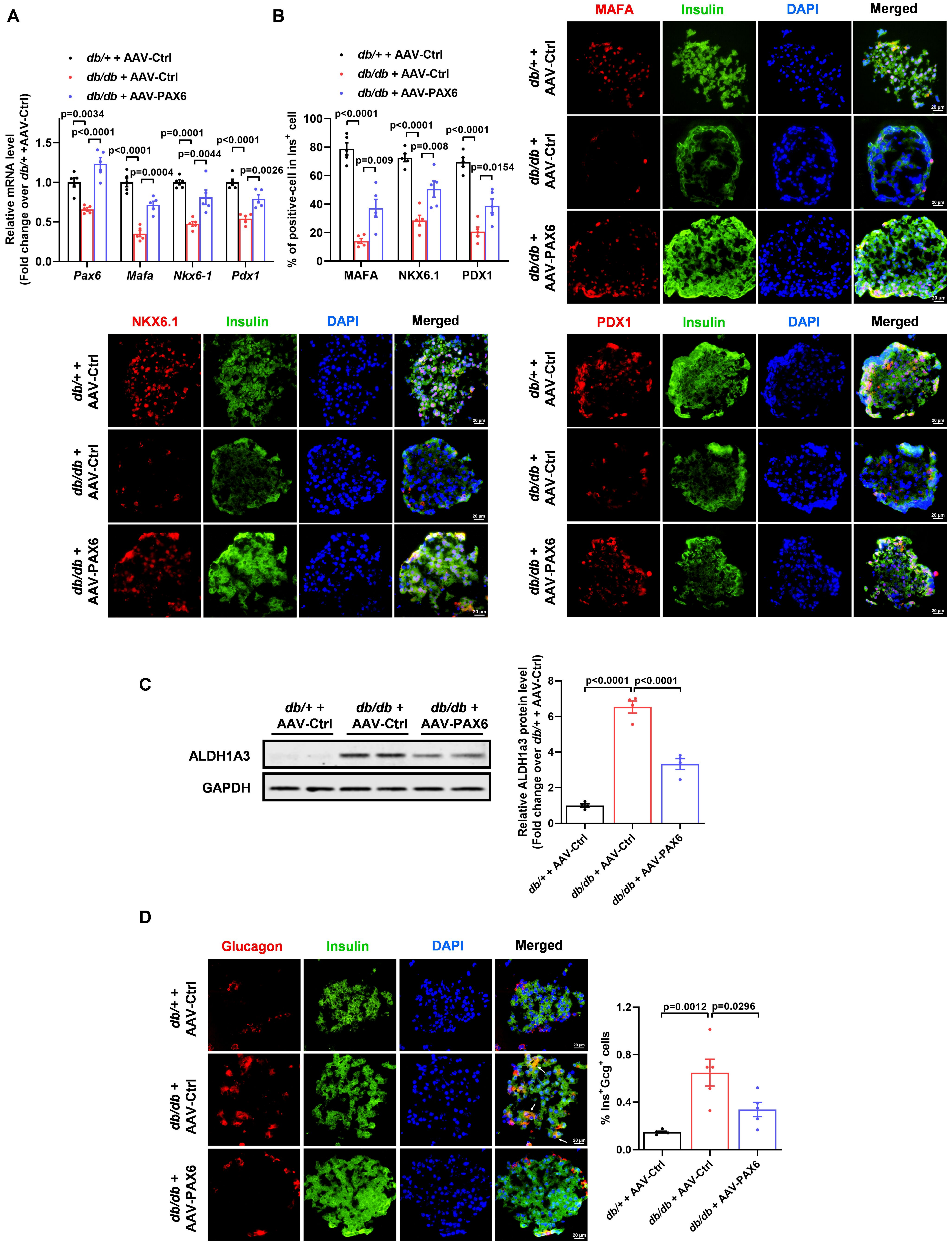
A
B
C
D
db/+ + AAV-Ctrl
db/db + AAV-Ctrl
db/db + AAV-PAX6
Relative mRNA level
(Fold change over db/+ +AAV-Ctrl)
Pax6
Mafa
Nkx6-1
Pdx1
p=0.0034
p<0.0001
p<0.0001
p=0.0004
p=0.0001
p=0.0044
p<0.0001
p=0.0026
% of positive-cell in Ins+ cell
MAFA
NKX6.1
PDX1
p<0.0001
p=0.009
p<0.0001
p=0.008
p<0.0001
p=0.0154
MAFA
Insulin
DAPI
Merged
NKX6.1
PDX1
ALDH1A3
GAPDH
Relative ALDH1a3 protein level
(Fold change over db/+ + AAV-Ctrl)
p<0.0001
p<0.0001
Glucagon
% Ins+Gcg+ cells
p=0.0012
p=0.0296
20 μm

Appendix figure S5. Effects of PAX6 replenishment on beta cell identity in *db/db* mice.

A. mRNA expression of *Pax6*, *Mafa*, *Nkx6.1* and *Pdx1* in isolated islets of *db/+* and *db/db* mice with AAV injection (n = 5).

B. Representative immunostaining and quantification of isolated islets from *db/+* and *db/db* mice labelled for MAFA/ NKX6.1/ PDX1 (red), insulin (green) and DAPI (blue) (n = 5). Scale bar = 20 μm.

C. Protein expression of ALDH1A3 in isolated islets of *db/+* and *db/db* mice (n = 4).

D. Representative immunostaining and quantification of islets from *db/+* and *db/db* mice labelled for glucagon (red), insulin (green) and DAPI (blue) (n = 5). Scale bar = 20 μm.

Data information: Each n represents the measurement of a sample from distinct mice (A-D). One-way ANOVA (A-D). Data are means ± SEM.

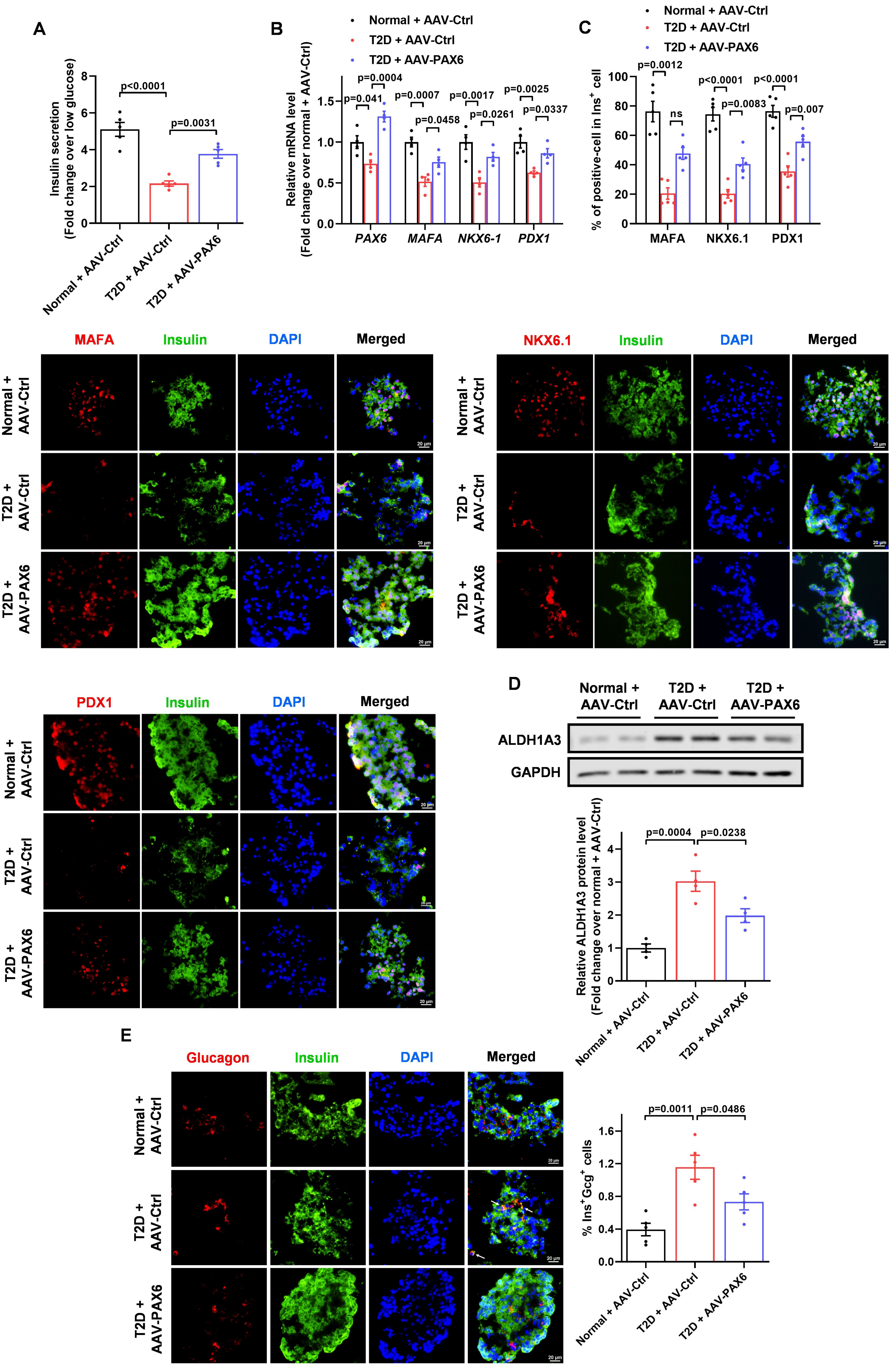
A
Insulin secretion (Fold change over low glucose)
p<0.0001
p=0.0031
Normal + AAV-Ctrl
T2D + AAV-Ctrl
T2D + AAV-PAX6
B
Normal + AAV-Ctrl
T2D + AAV-Ctrl
T2D + AAV-PAX6
Relative mRNA level (Fold change over normal + AAV-Ctrl)
p=0.041
p=0.0004
p=0.0007
p=0.0458
p=0.0017
p=0.0261
p=0.0025
p=0.0337
PAX6
MAFA
NKX6-1
PDX1
C
Normal + AAV-Ctrl
T2D + AAV-Ctrl
T2D + AAV-PAX6
% of positive-cell in Ins+ cell
p=0.0012
ns
p<0.0001
p=0.0083
p<0.0001
p=0.007
MAFA
NKX6.1
PDX1
MAFA
Insulin
DAPI
Merged
NKX6.1
Insulin
DAPI
Merged
Normal + AAV-Ctrl
T2D + AAV-Ctrl
T2D + AAV-PAX6
20 μm
PDX1
Insulin
DAPI
Merged
D
Normal + AAV-Ctrl
T2D + AAV-Ctrl
T2D + AAV-PAX6
ALDH1A3
GAPDH
Relative ALDH1A3 protein level (Fold change over normal + AAV-Ctrl)
p=0.0004
p=0.0238
E
Glucagon
Insulin
DAPI
Merged
% Ins+Gcg+ cells
p=0.0011
p=0.0486

Appendix figure S6. Effects of PAX6 replenishment on islet function and beta cell identity in human T2D islets.

A. GSIS of normal or T2D human islets with AAV transduction (n = 5).

B. mRNA expression of *PAX6*, *MAFA*, *NKX6.1* and *PDX1* in normal and T2D human islets with AAV transduction (n = 4).

C. Representative immunostaining and quantification of normal and T2D human islets labelled for MAFA/ NKX6.1/ PDX1 (red), insulin (green) and DAPI (blue) (n = 5). Scale bar = 20 μm.

D. Protein expression of ALDH1A3 in normal and T2D human islets (n = 4).

E. Representative immunostaining and quantification of normal and T2D human islets labelled for glucagon (red), insulin (green) and DAPI (blue) (n = 5). Scale bar = 20 μm.

Data information: Each n represents an independent biological replicate (A-E). One-way ANOVA (A, B, D, E). One-way ANOVA and Kruskal-Wallis test (C). Data are means ± SEM. ns, nonsignificant.

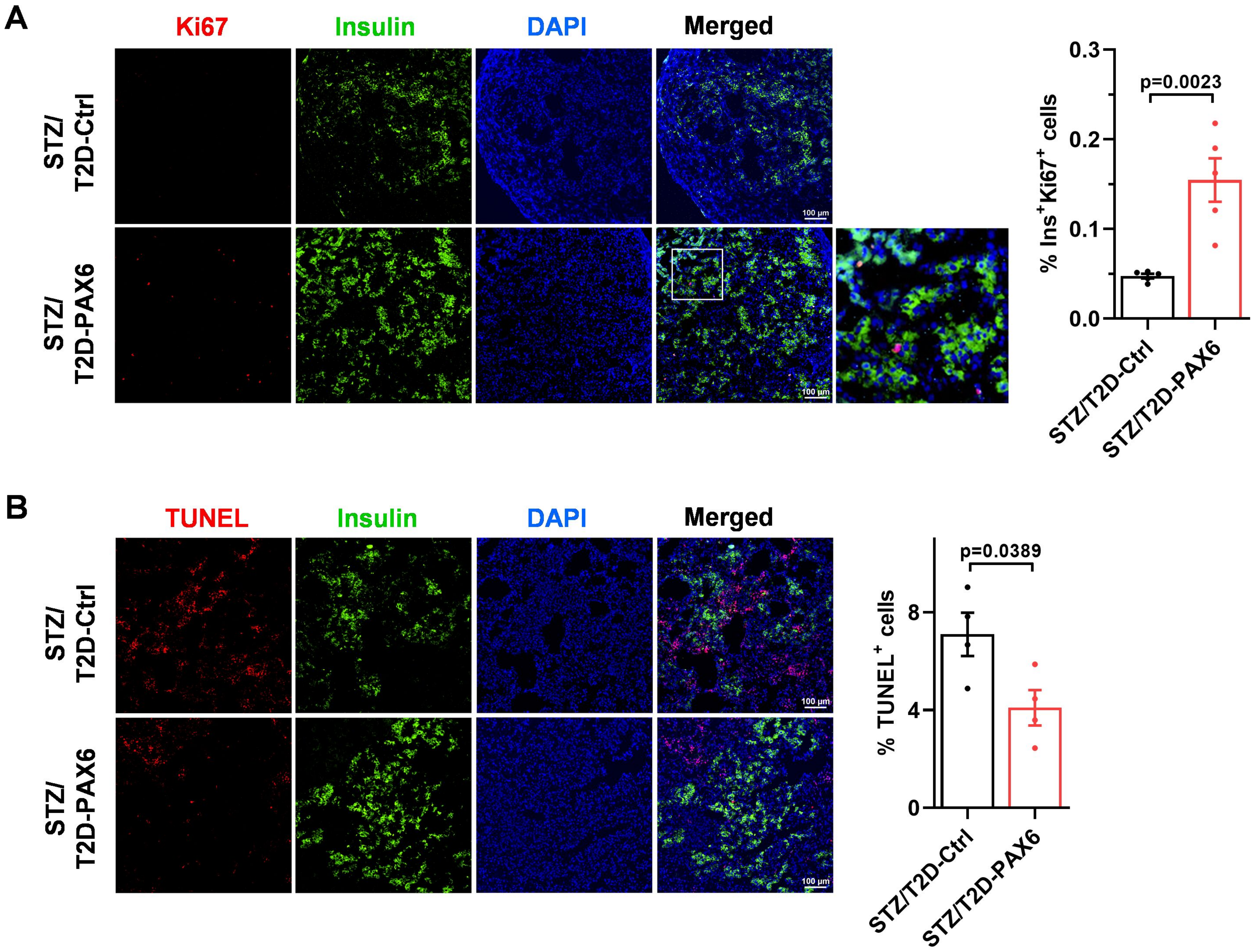

Appendix figure S7. Effects of PAX6 overexpression on human T2D islet graft survival after transplantation into immunodeficient diabetic mice.

A, B. Representative immunostaining and quantification of human islet grafts labelled for (A) Ki67 (red)/ insulin (green)/ DAPI (blue) (n = 5) and (B) TUNEL (red)/ insulin (green)/ DAPI (blue) (n = 4). Scale bar = 100 μm.

Data information: Each n represents the measurement of a sample from distinct mice (A, B). Unpaired Student's t test (A, B). Data are means ± SEM.

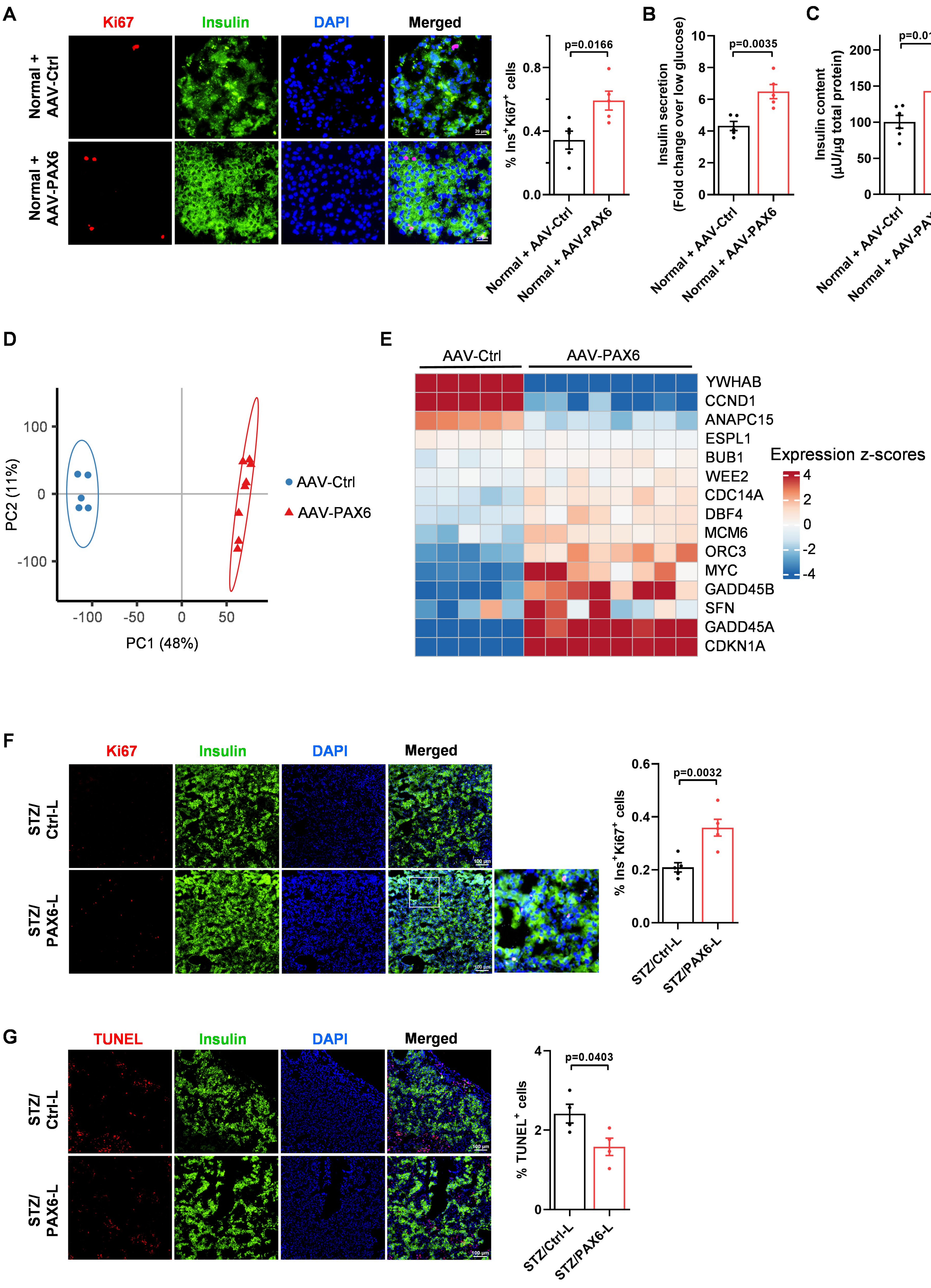
A
Ki67
Insulin
DAPI
Merged
Normal + AAV-Ctrl
Normal + AAV-PAX6
% Ins+Ki67+ cells
p=0.0166
Normal + AAV-Ctrl
Normal + AAV-PAX6
B
Insulin secretion (Fold change over low glucose)
p=0.0035
C
Insulin content (µU/µg total protein)
p=0.014
D
PC2 (11%)
PC1 (48%)
AAV-Ctrl
AAV-PAX6
E
AAV-Ctrl
AAV-PAX6
YWHAB
CCND1
ANAPC15
ESPL1
BUB1
WEE2
CDC14A
DBF4
MCM6
ORC3
MYC
GADD45B
SFN
GADD45A
CDKN1A
Expression z-scores
F
Ki67
Insulin
DAPI
Merged
STZ/ Ctrl-L
STZ/ PAX6-L
% Ins+Ki67+ cells
p=0.0032
STZ/Ctrl-L
STZ/PAX6-L
G
TUNEL
Insulin
DAPI
Merged
STZ/ Ctrl-L
STZ/ PAX6-L
% TUNEL+ cells
p=0.0403
STZ/Ctrl-L
STZ/PAX6-L

Appendix figure. S8. Effects of PAX6 overexpression on normal human islets and islet graft survival after transplantation into immunodeficient diabetic mice.

A. Representative immunostaining and quantification of human islets with AAV transduction labelled for Ki67 (red)/ insulin (green)/ DAPI (blue) (n = 5). Scale bar = 20 μm.

B, C. Measurement of (B) GSIS (n = 5) and (C) islet insulin content (n = 6) of normal human islets with AAV transduction.

D. Principle component analysis comparing the transcriptomes of human islets with AAV-Ctrl or AAV-PAX6 transduction.

E. A heatmap of DEGs showing the expression of genes involving in the regulation of DNA replication and cell proliferation in human islets with AAV-Ctrl or AAV-PAX6 transduction.

F, G. Representative immunostaining and quantification of human islet grafts labelled for (F) Ki67 (red)/ insulin (green)/ DAPI (blue) (n = 5) and (G) TUNEL (red)/ insulin (green)/ DAPI (blue) (n = 4). Scale bar = 100 μm.

Data information: Each n represents an independent biological replicate (A-E). Each n represents the measurement of a sample from distinct mice (F, G). Unpaired Student's t test (A-C, F, G). Data are means ± SEM.

Genes for knockdown	shRNA sequence
PAX6	AATTCTGGGCAGGTATTACGA
Non-targeting control	CAACAAGATGAAGAGCACCAA

Genes for qPCR	Forward primer (5'-3')	Reverse primer (5'-3')
Mouse *b-actin*	ACTGTCGAGTCGCGTCCA	ATCCATGGCGAACTGGTGG
Mouse *Pax6*	TAGCCCAGTATAAACGGGAGTG	CCAGGTTGCGAAGAACTCTG
Mouse *Mafa*	CAAGGAGGAGGTCATCCGAC	TCTCCAGAATGTGCCGCTG
Mouse *Nkx6.1*	CCCGGAGTGATGCAGAGTC	AGAACGTGGGTCTGGTGTGT
Mouse *Pdx1*	ATTCTTGAGGGCACGAGAGC	CTGGTCCGTATTGGAACGCT
Human *b-actin*	ACAGAGCCTCGCCTTTGCC	GATATCATCATCCATGGTGAGCTGG
Human *PAX6*	AACGATAACATACCAAGCGTGT	GGTCTGCCCGTTCAACATC
Human *MAFA*	TCGTCGCTCTTTGGACTAGC	AAACTTTCAACGAGTCGGCG
Human *NKX6.1*	GCCTGTACCCCTCATCAAGGA	TGCTGTCCGGAAAAAGTGGG
Human *PDX1*	GCGTCCTGGAGGAGCCCAAC	GTGCGCGTCCGCTTGTTCTC
Human *PAX4*	GGCAGTATCCTGATTCAGTGGC	GCTTCTCTTGCCGACGCCATTT
Human *ONECUT1*	GAGGATGTGGAAGTGGCTGCAG	CTGTGAAGACCAACCTGGGCTT
Human *HNF4A*	GGTGTCCATACGCATCCTTGAC	AGCCGCTTGATCTTCCCTGGAT
Human *NEUROD1*	GGTGCCTTGCTATTCTAAGACGC	GCAAAGCGTCTGAACGAAGGAG
Human *TCF7L2*	GAATCGTCCCAGAGTGATGTCG	TGCACTCAGCTACGACCTTTGC
Human *ISL1*	GCAGAGTGACATAGATCAGCCTG	GCCTCAATAGGACTGGCTACCA

Appendix Table S1. Oligonucleotide sequences used in shRNA vectors construction and quantitative real-time PCR.

Donor ID	Age	Gender	Weight (Ibs)	BMI	HbA1c (%)
Normal 1	23	F	157	24.5	4.9
Normal 2	41	M	218	35.12	5.8
Normal 3	51	M	195	31.45	5.2
Normal 4	55	M	160	23.63	4.7
Normal 5	31	M	202	26.45	5.2
Normal 6	26	M	197	26.37	5.4
Normal 7	62	F	123	25.66	5.7
T2D 1	55	F	164	29.9	7.4
T2D 2	63	M	234	31.69	6.9
T2D 3	52	M	158	24.75	7.6
T2D 4	59	M	168	25.54	7.3
T2D 5	62	M	250	34.61	6.8

Appendix Table S2. Human islet donor information.
