# Supplementary figures and images for: Paired box 6 gene delivery preserves beta cells and improves islet transplantation efficacy (part 1 of 2)
Source: EMBO Mol Med. 2023 Nov 7;15(12):e17928. doi: 10.15252/emmm.202317928 (PMC10701606; doi:10.15252/emmm.202317928)

E

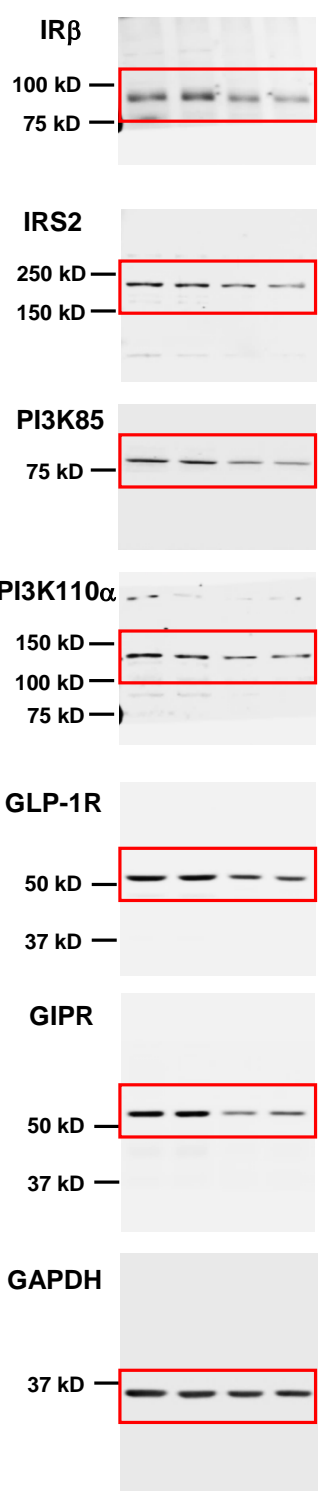

F

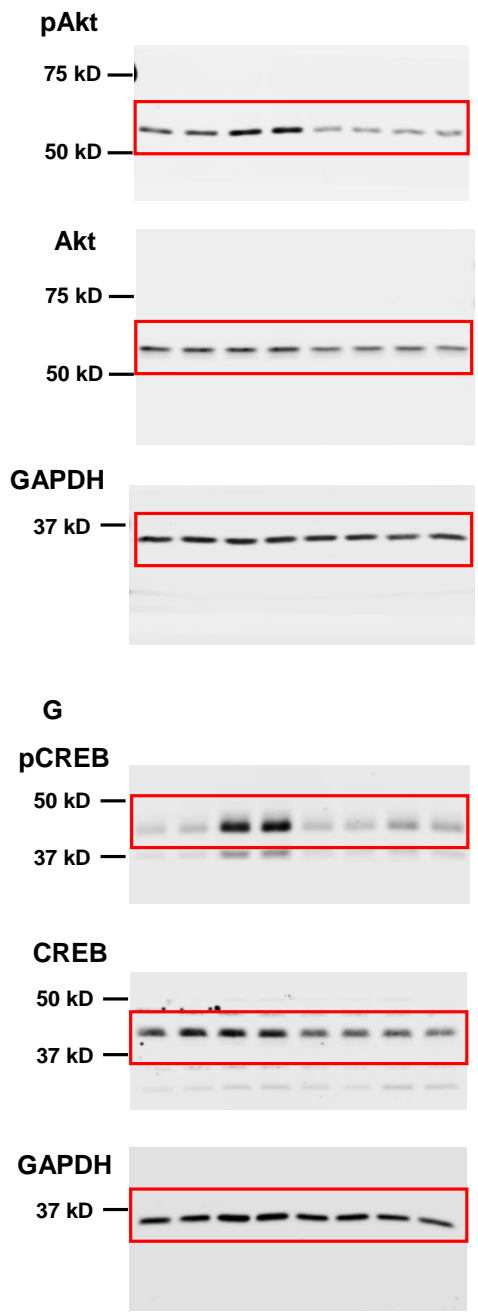

H

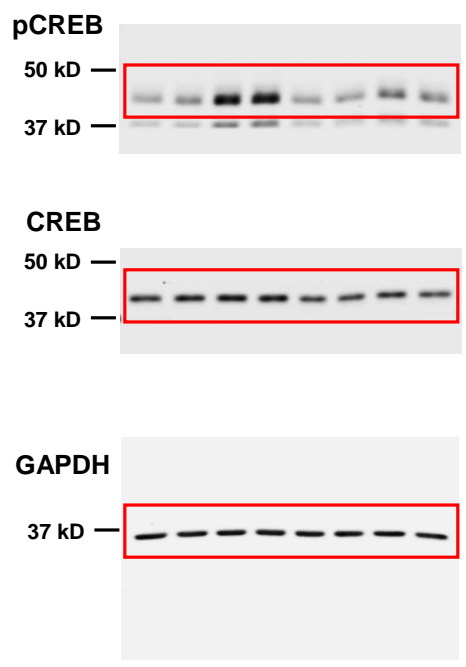

G

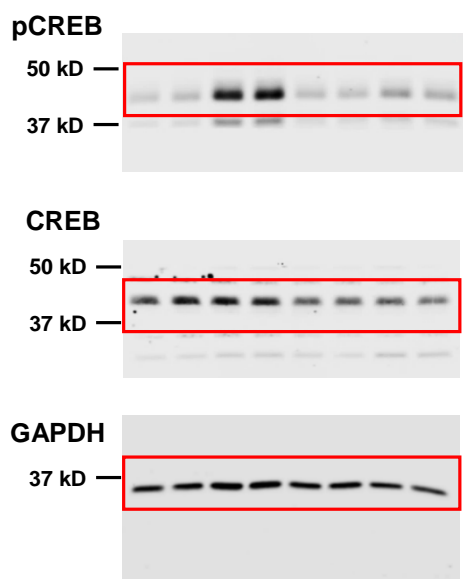

Supplement: Supplementary file 2 — Source Data for Figure 1 [file EMMM-15-e17928-s002.zip › Figure_1/Figure_1_western.pdf]

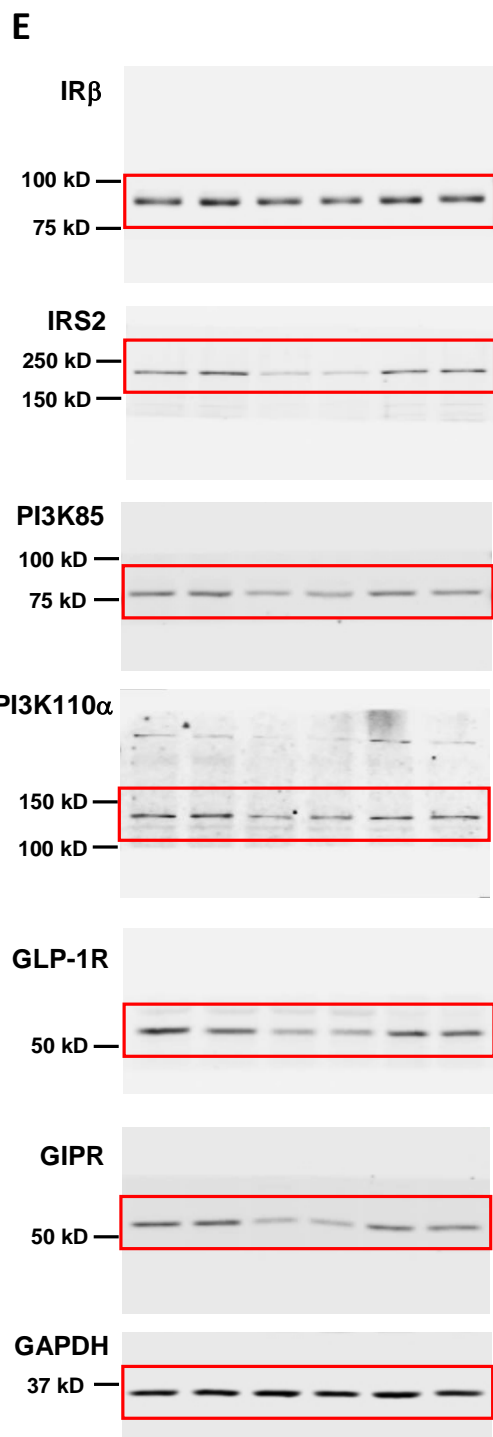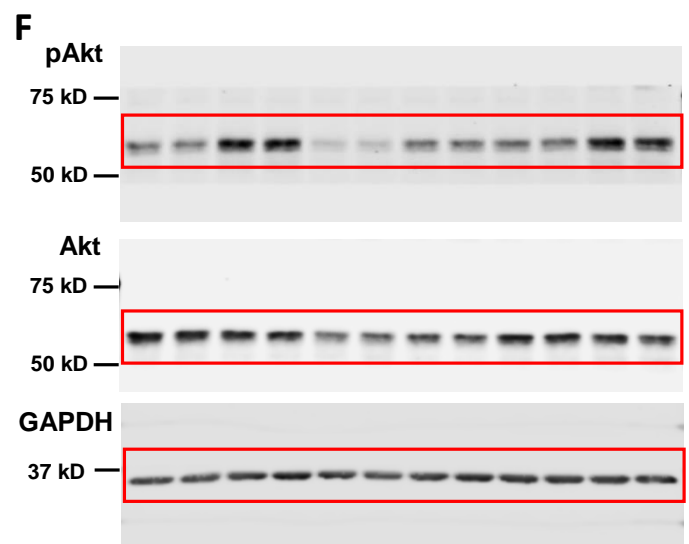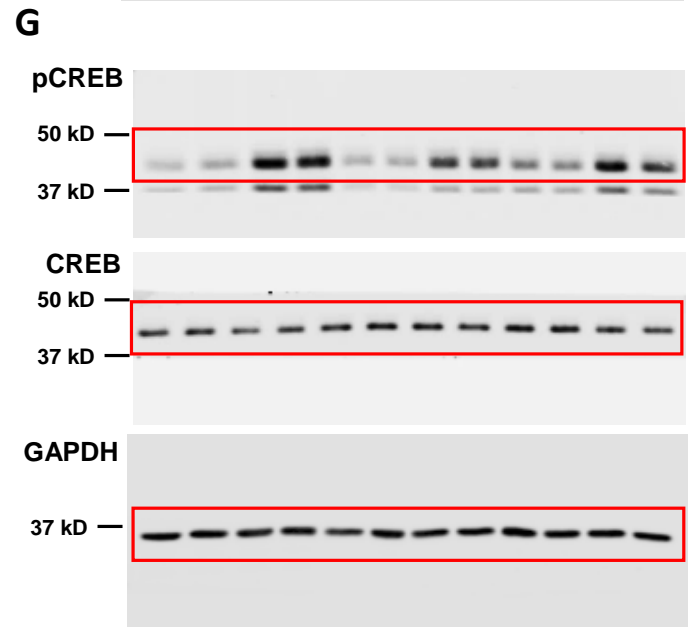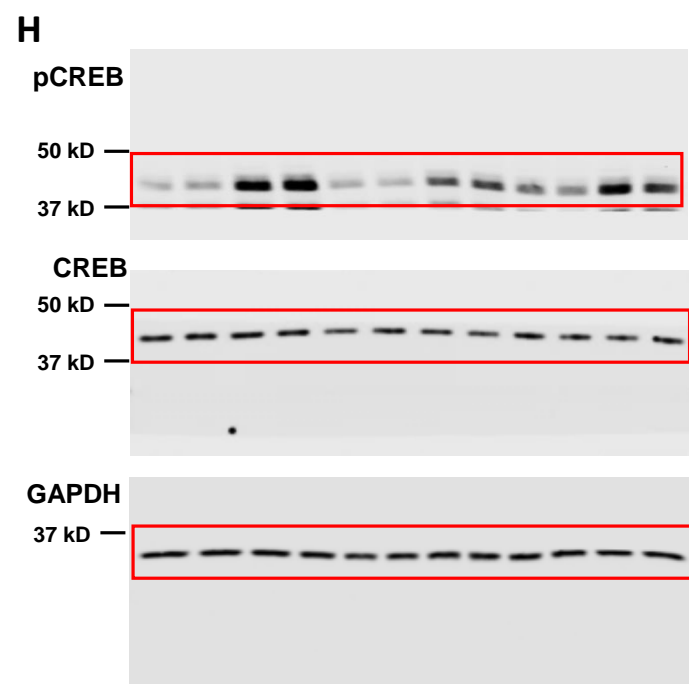

Supplement: Supplementary file 3 — Source Data for Figure 2 [file EMMM-15-e17928-s006.zip › Figure_2/Figure_2_western.pdf]

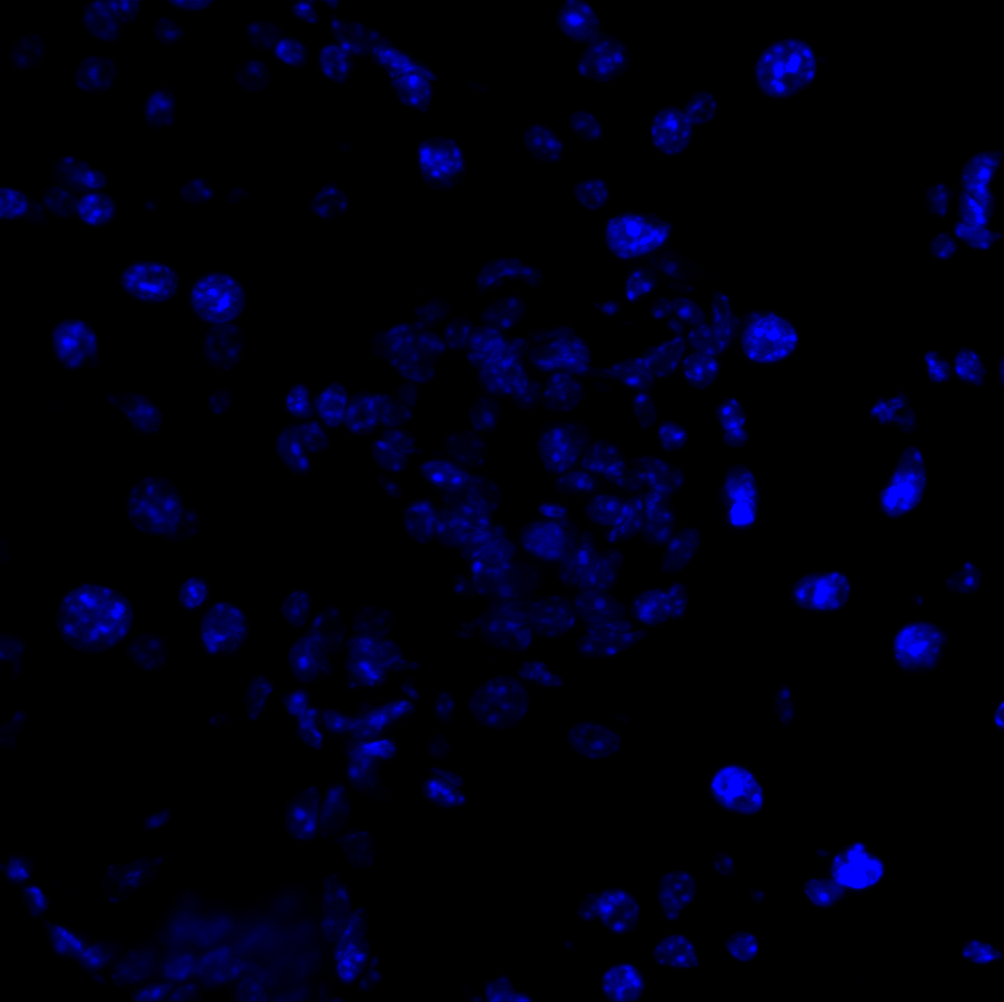

Supplement: Supplementary file 4 — Source Data for Figure 3 [file EMMM-15-e17928-s008.zip › Figure_3/3H_STZ+AAV-Ctrl_DAPI.tif]

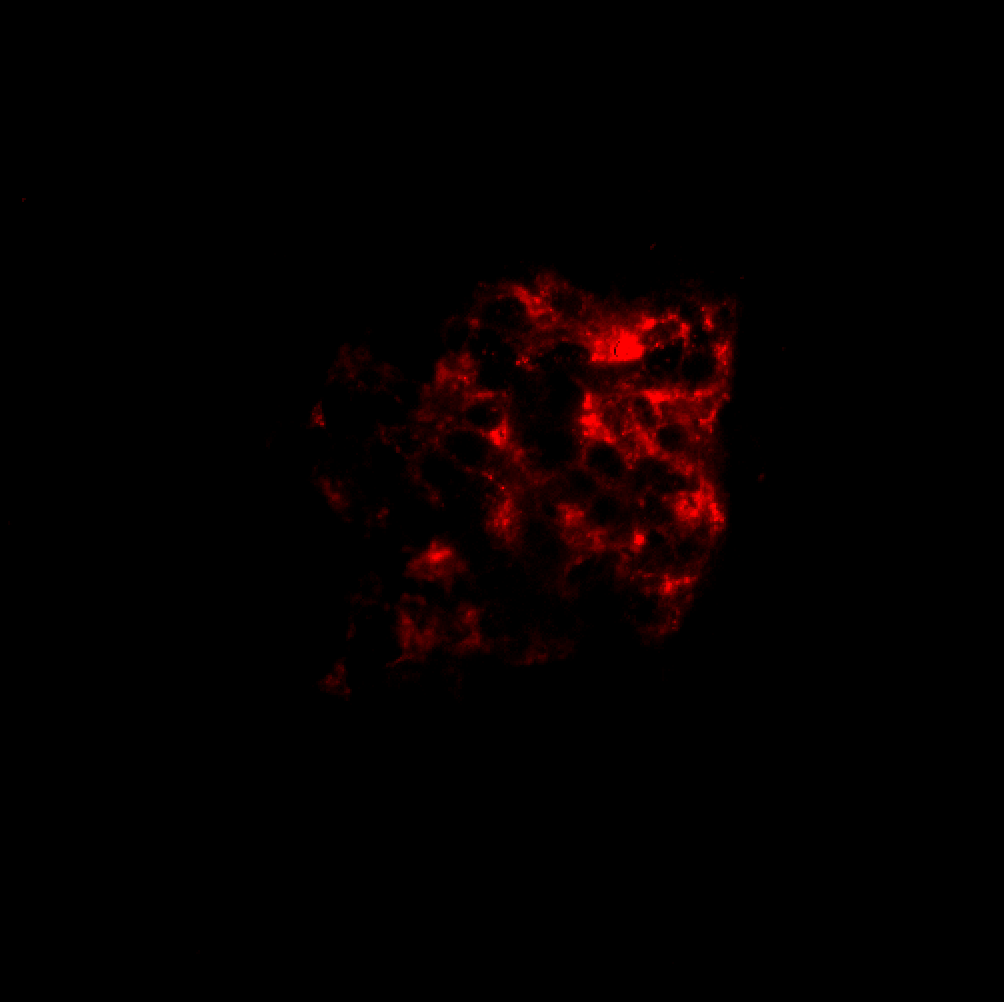

Supplement: Supplementary file 4 — Source Data for Figure 3 [file EMMM-15-e17928-s008.zip › Figure_3/3H_STZ+AAV-Ctrl_Glucagon.tif]

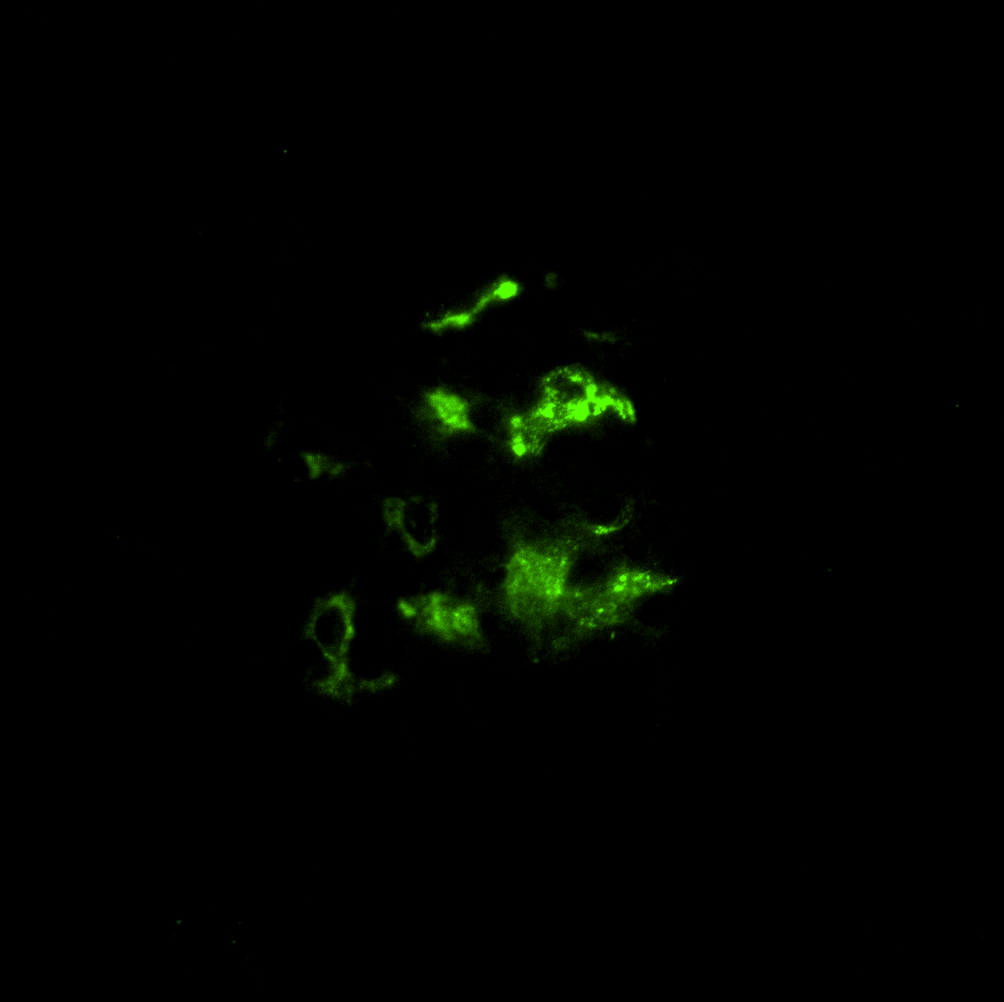

Supplement: Supplementary file 4 — Source Data for Figure 3 [file EMMM-15-e17928-s008.zip › Figure_3/3H_STZ+AAV-Ctrl_Insulin.tif]

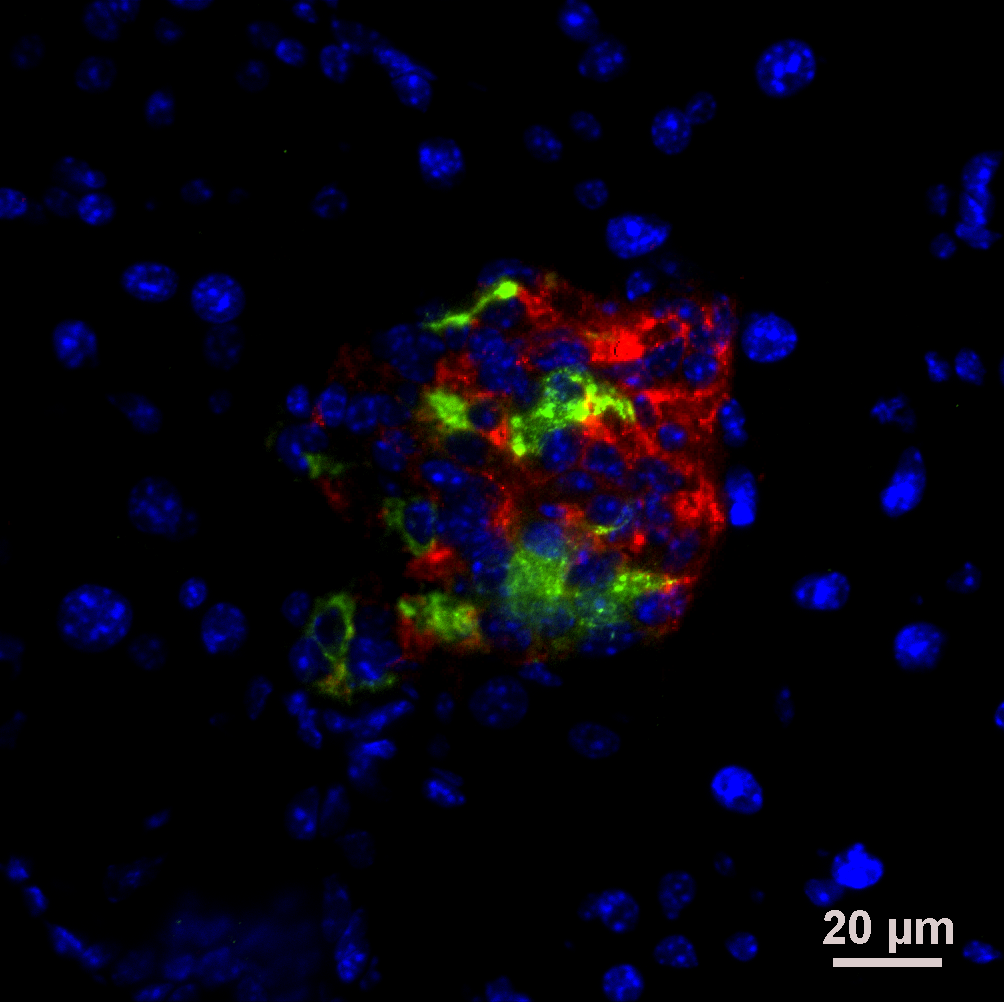

Supplement: Supplementary file 4 — Source Data for Figure 3 [file EMMM-15-e17928-s008.zip › Figure_3/3H_STZ+AAV-Ctrl_Merged.tif]

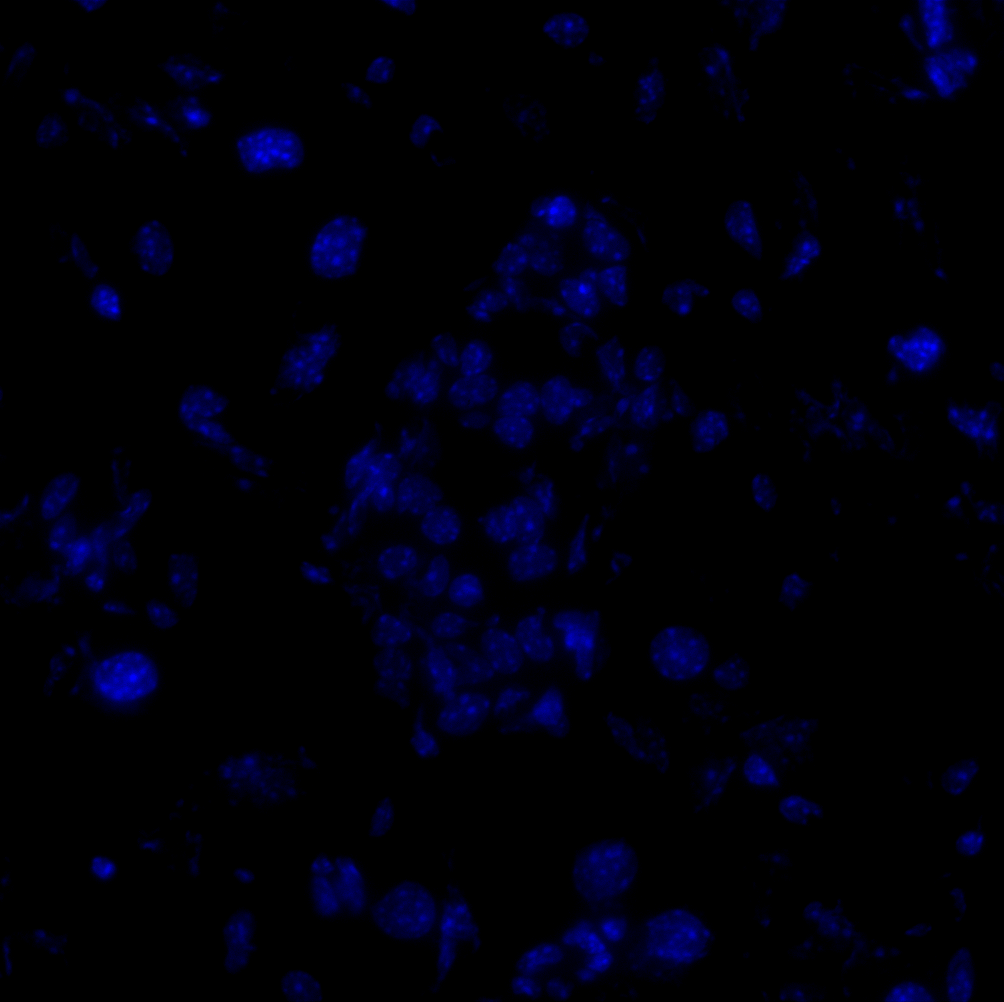

Supplement: Supplementary file 4 — Source Data for Figure 3 [file EMMM-15-e17928-s008.zip › Figure_3/3H_STZ+AAV-PAX6_DAPI.tif]

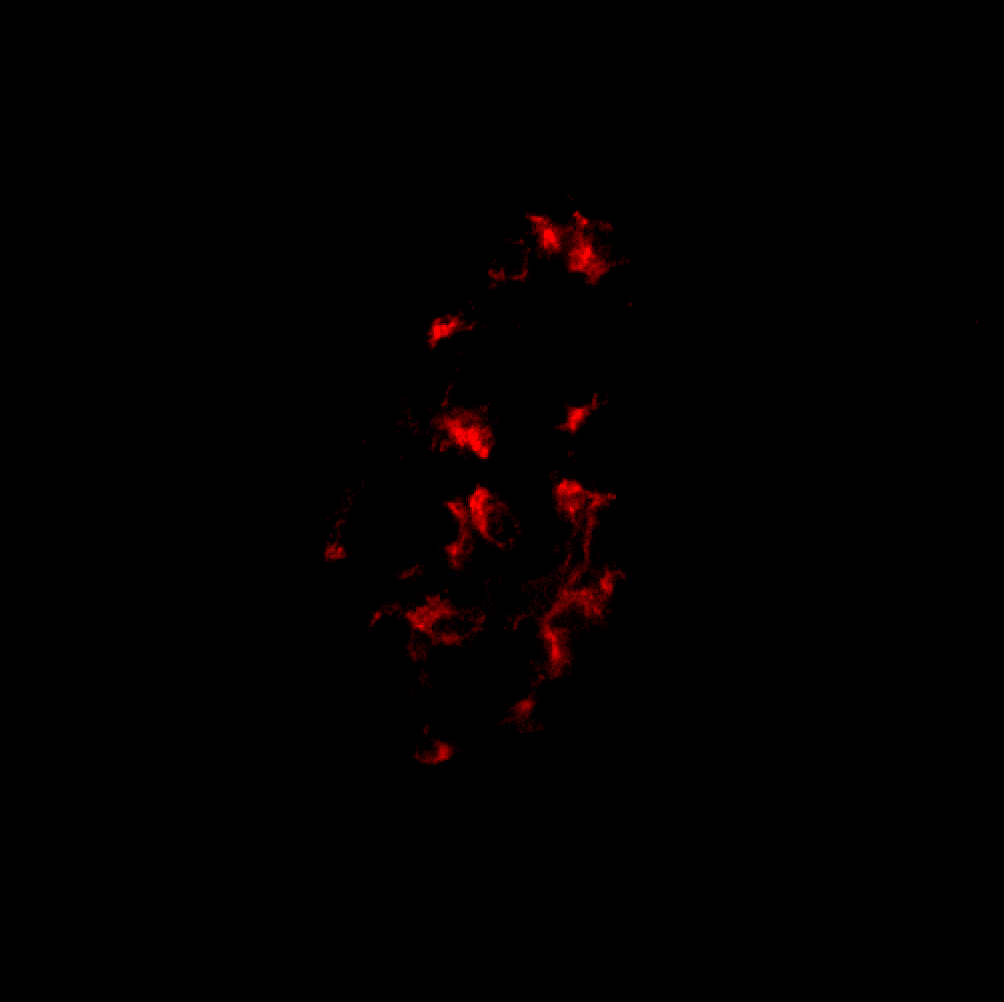

Supplement: Supplementary file 4 — Source Data for Figure 3 [file EMMM-15-e17928-s008.zip › Figure_3/3H_STZ+AAV-PAX6_Glucagon.tif]

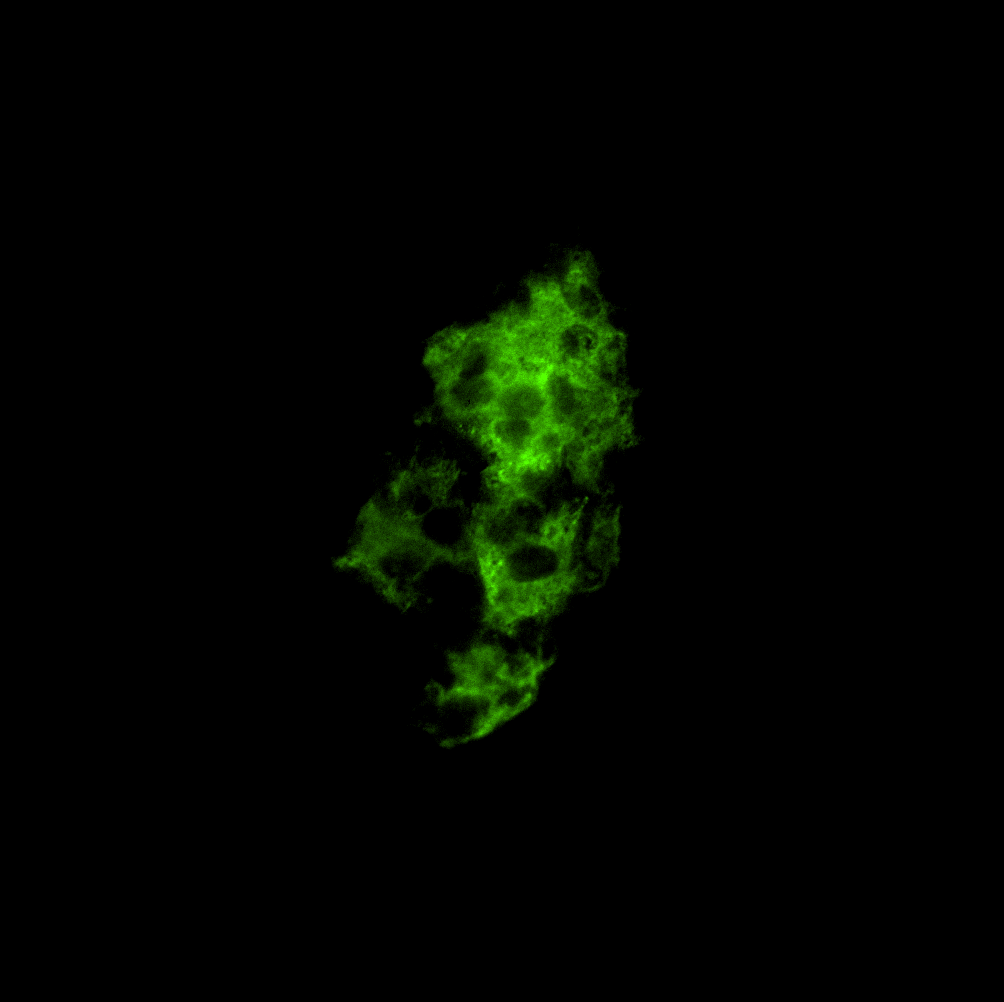

Supplement: Supplementary file 4 — Source Data for Figure 3 [file EMMM-15-e17928-s008.zip › Figure_3/3H_STZ+AAV-PAX6_Insulin.tif]

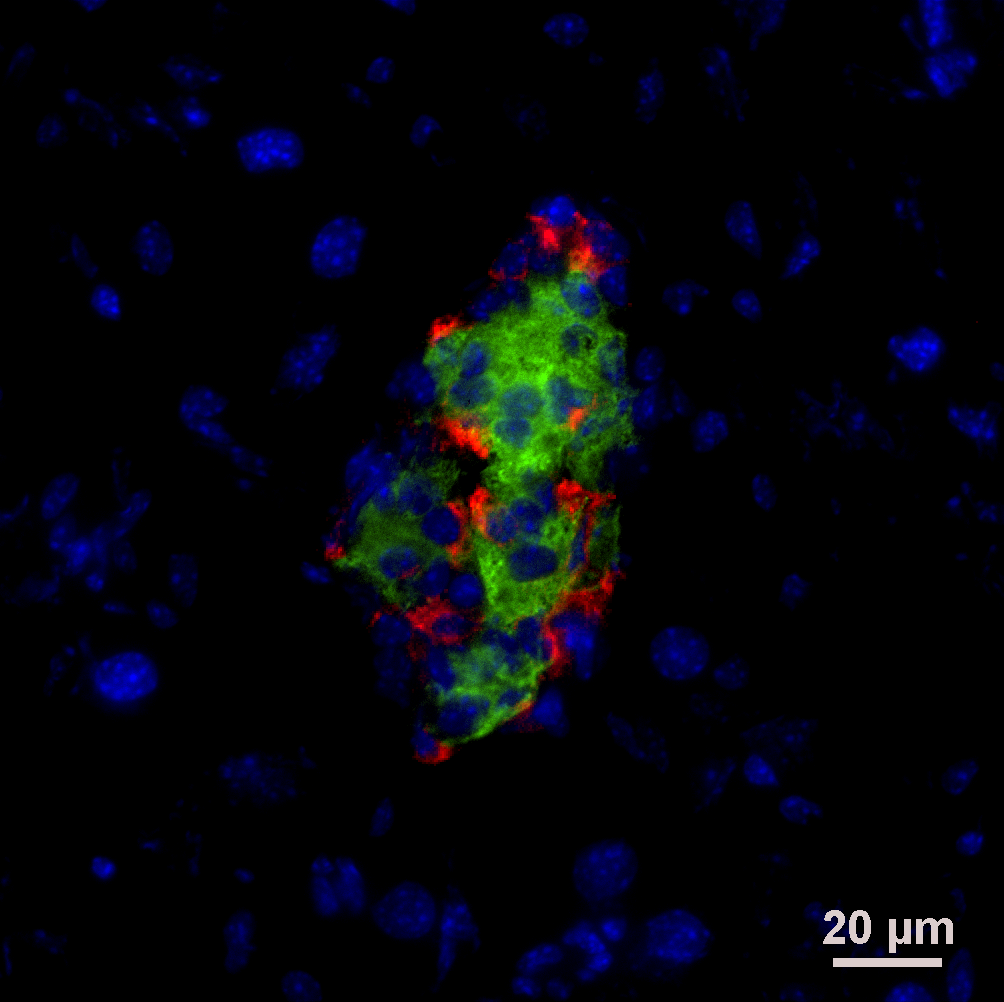

Supplement: Supplementary file 4 — Source Data for Figure 3 [file EMMM-15-e17928-s008.zip › Figure_3/3H_STZ+AAV-PAX6_Merged.tif]

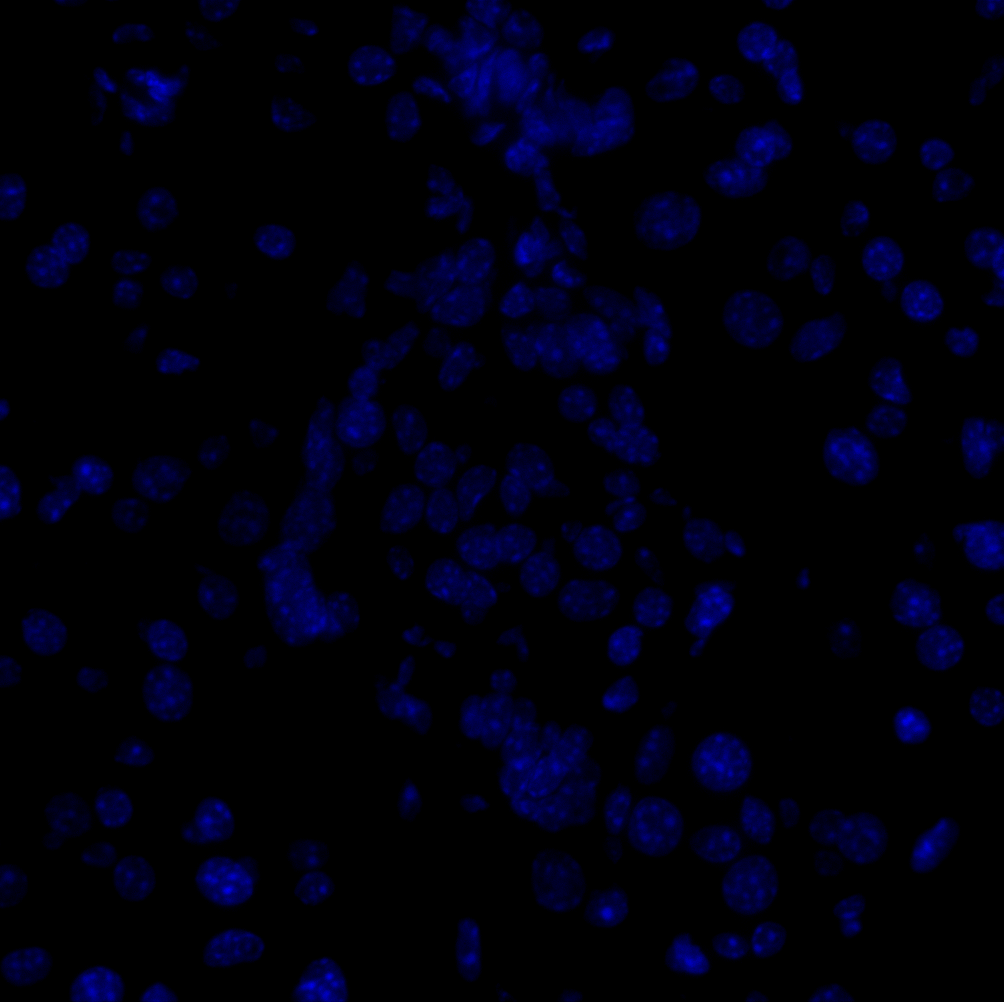

Supplement: Supplementary file 4 — Source Data for Figure 3 [file EMMM-15-e17928-s008.zip › Figure_3/3H_vehicle+AAV-Ctrl_DAPI.tif]

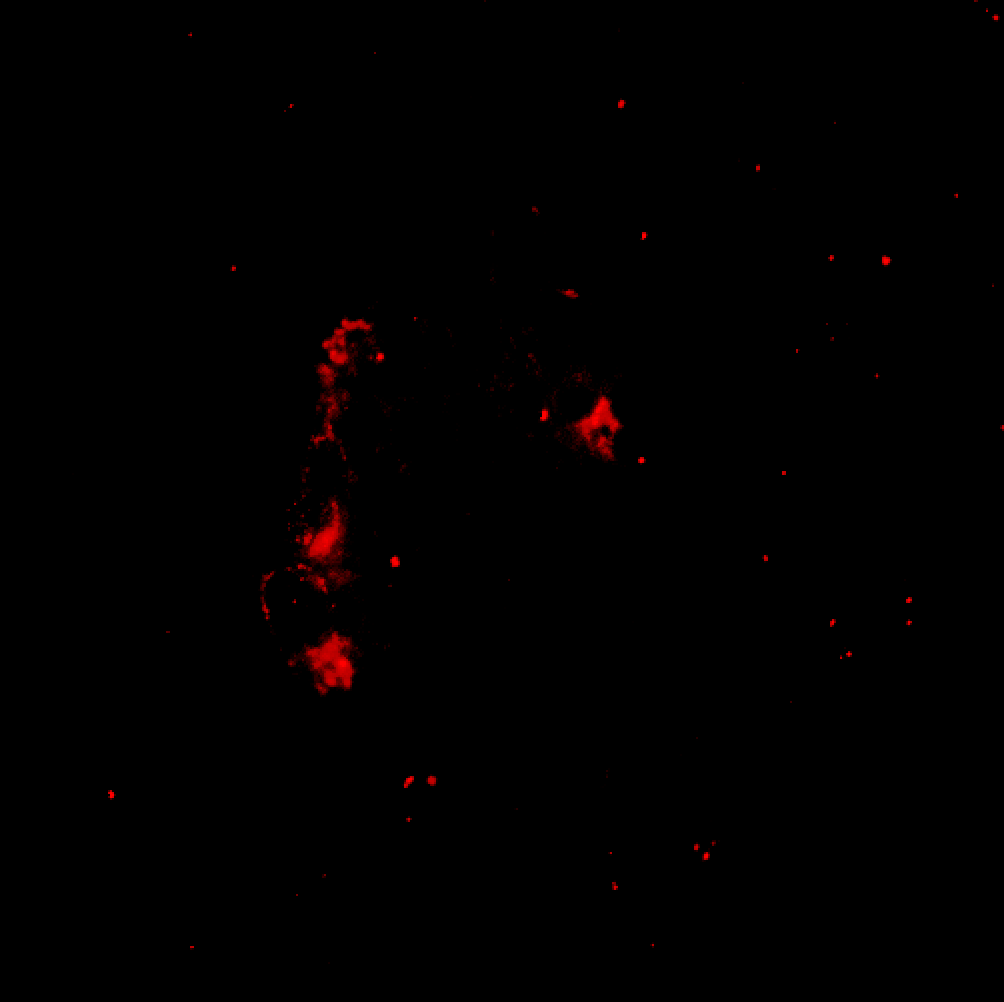

Supplement: Supplementary file 4 — Source Data for Figure 3 [file EMMM-15-e17928-s008.zip › Figure_3/3H_vehicle+AAV-Ctrl_Glugacon.tif]

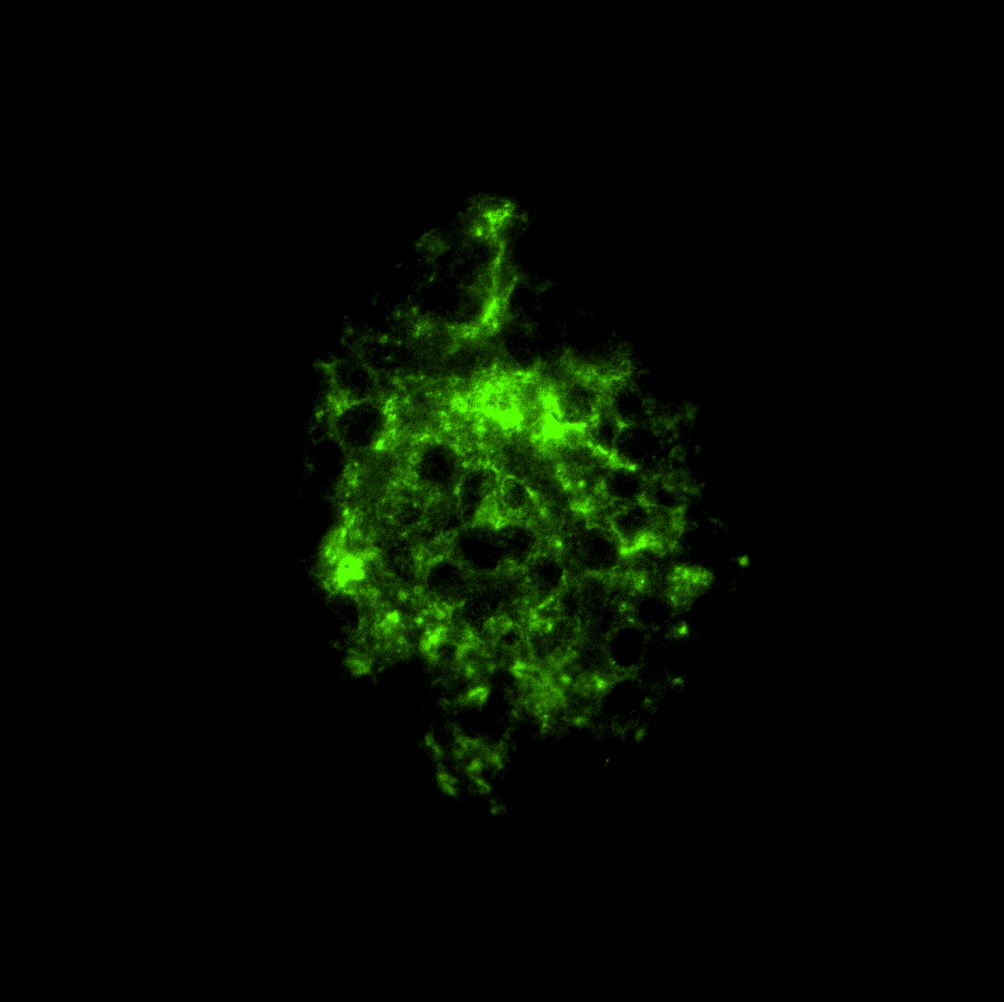

Supplement: Supplementary file 4 — Source Data for Figure 3 [file EMMM-15-e17928-s008.zip › Figure_3/3H_vehicle+AAV-Ctrl_Insulin.tif]

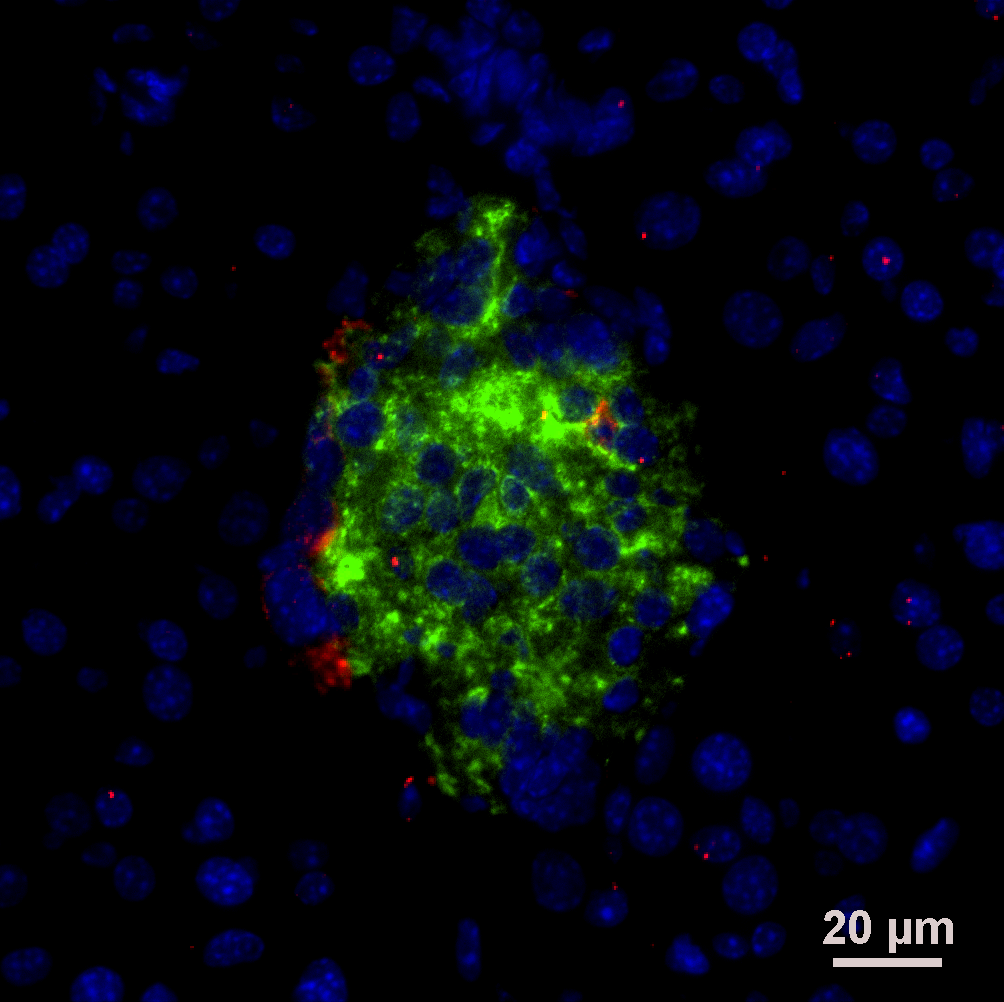

Supplement: Supplementary file 4 — Source Data for Figure 3 [file EMMM-15-e17928-s008.zip › Figure_3/3H_vehicle+AAV-Ctrl_Merged.tif]

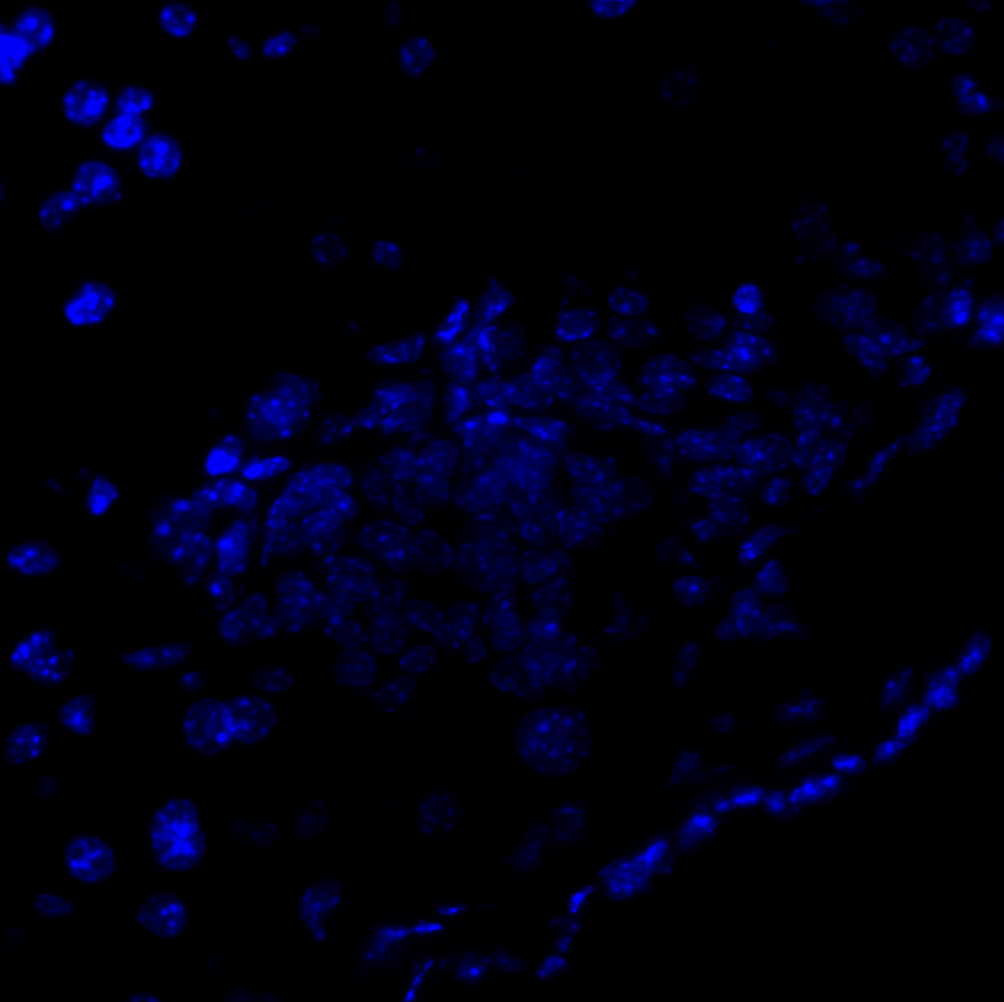

Supplement: Supplementary file 4 — Source Data for Figure 3 [file EMMM-15-e17928-s008.zip › Figure_3/3I_STZ+AAV-Ctrl_DAPI.tif]

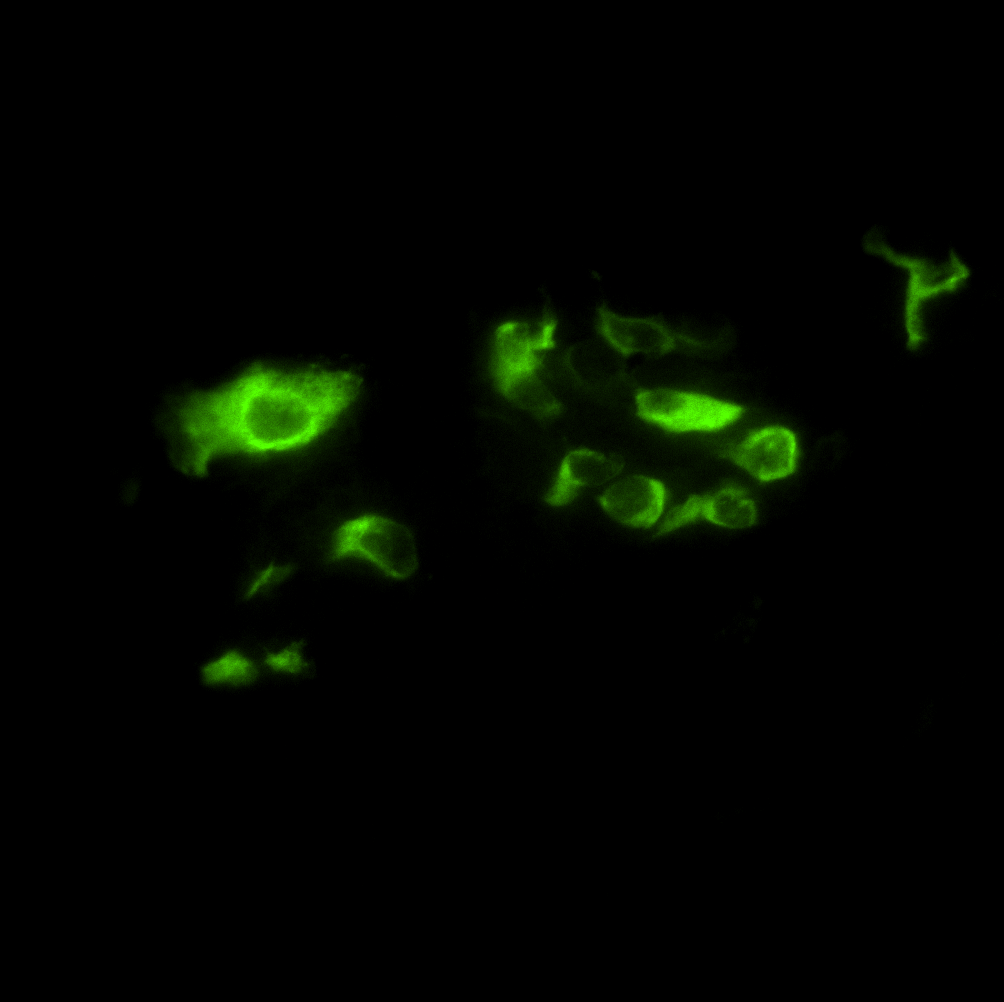

Supplement: Supplementary file 4 — Source Data for Figure 3 [file EMMM-15-e17928-s008.zip › Figure_3/3I_STZ+AAV-Ctrl_Insulin.tif]

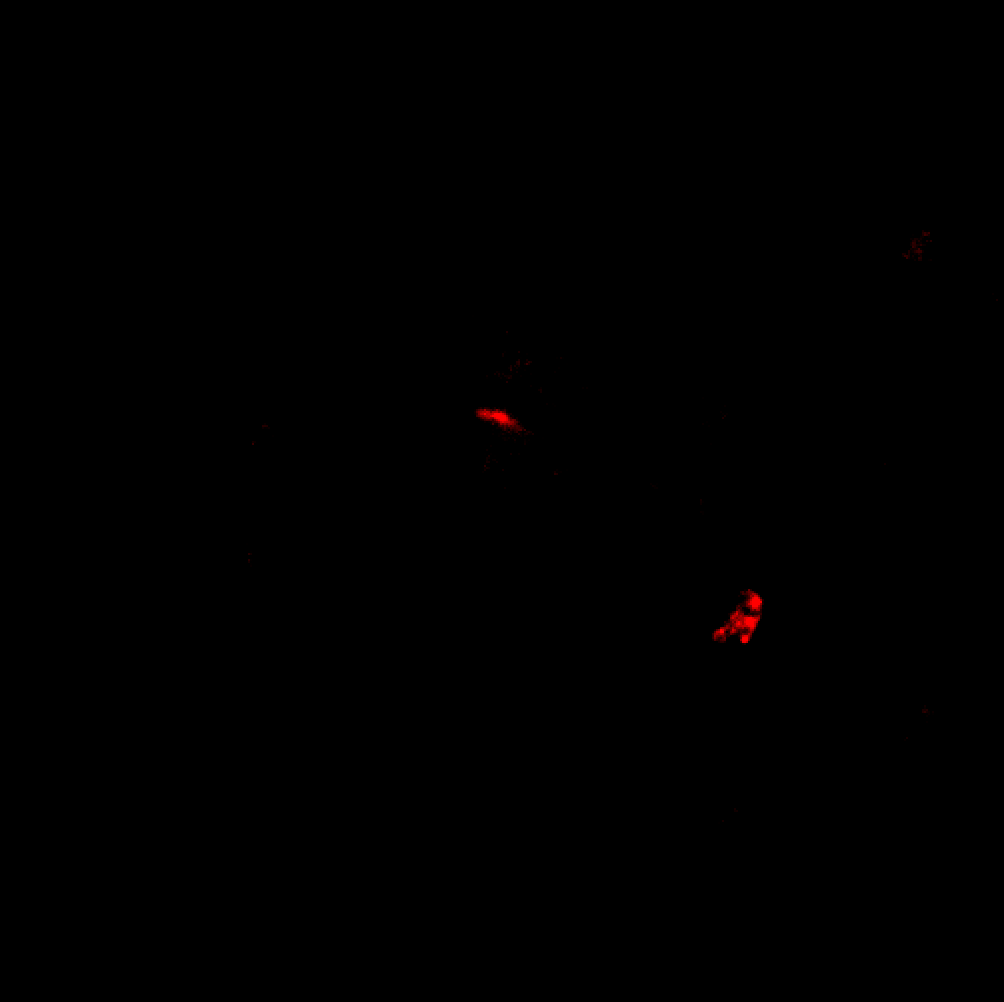

Supplement: Supplementary file 4 — Source Data for Figure 3 [file EMMM-15-e17928-s008.zip › Figure_3/3I_STZ+AAV-Ctrl_Ki67.tif]

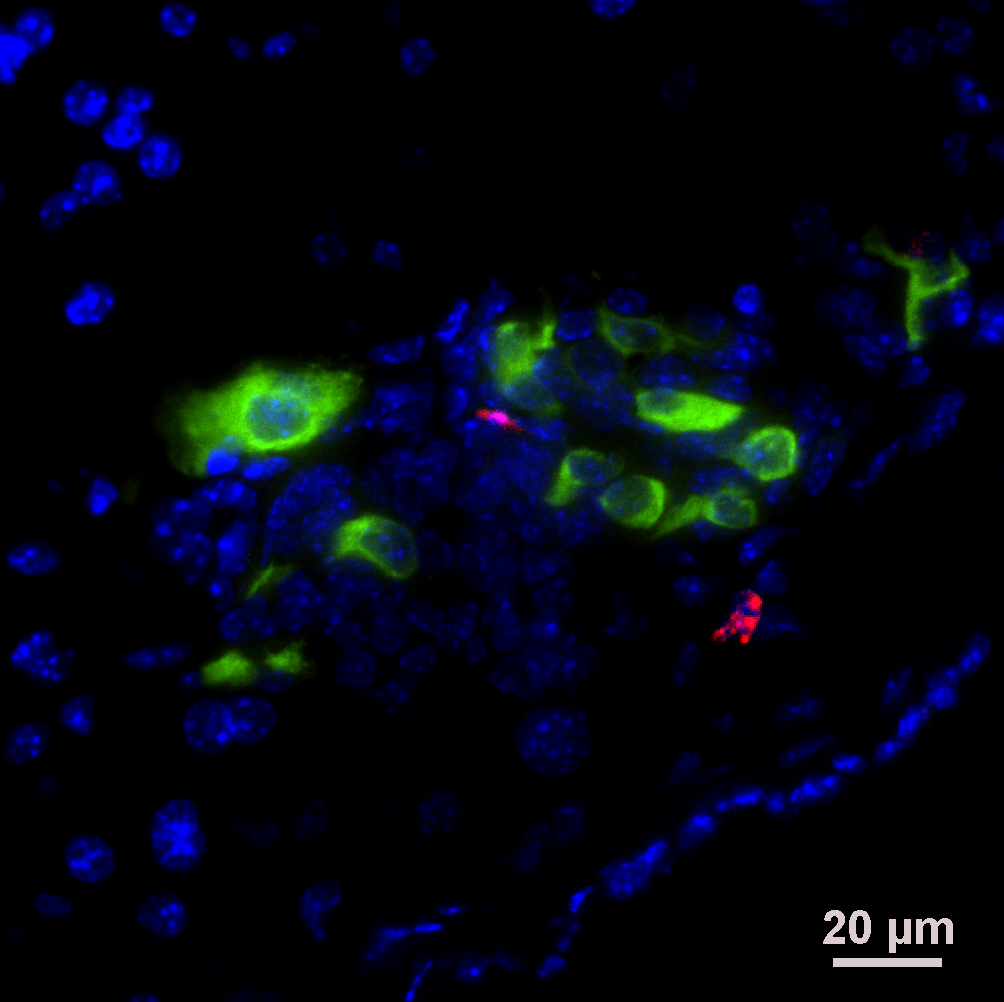

Supplement: Supplementary file 4 — Source Data for Figure 3 [file EMMM-15-e17928-s008.zip › Figure_3/3I_STZ+AAV-Ctrl_Merged.tif]

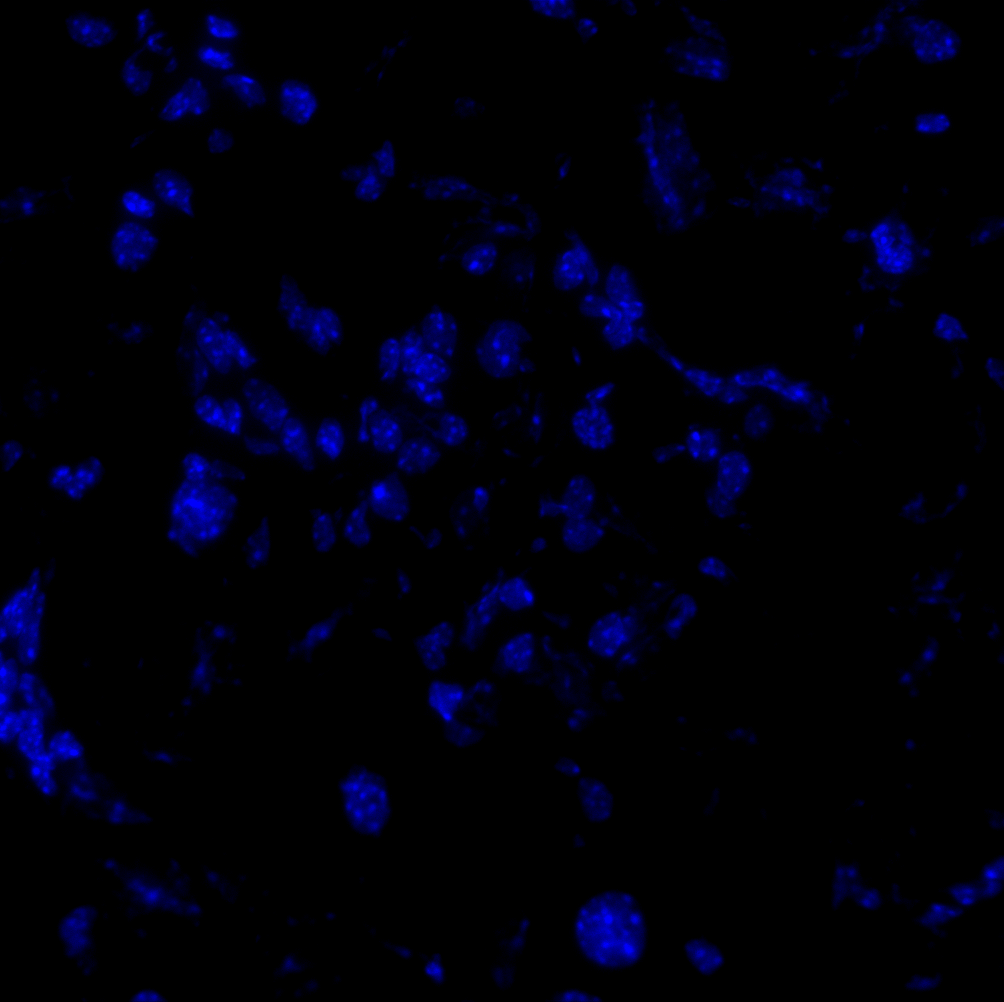

Supplement: Supplementary file 4 — Source Data for Figure 3 [file EMMM-15-e17928-s008.zip › Figure_3/3I_STZ+AAV-PAX6_DAPI.tif]

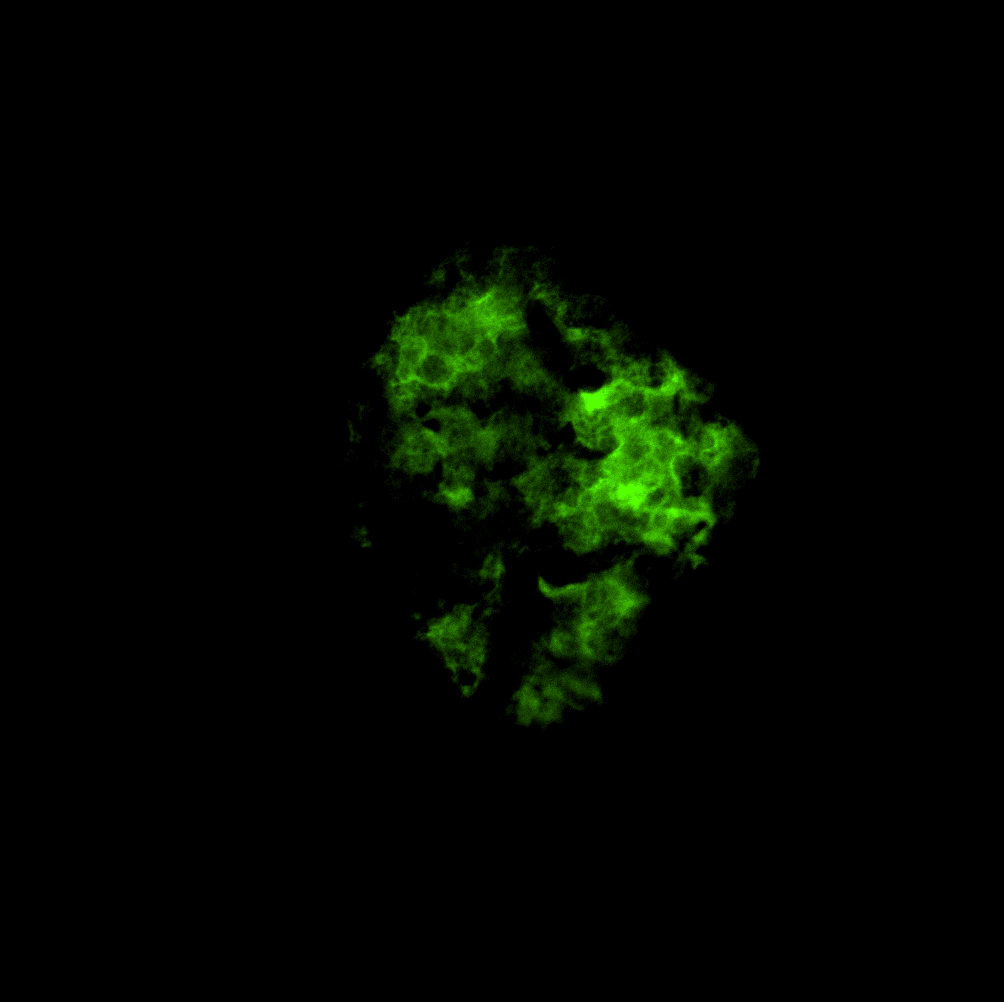

Supplement: Supplementary file 4 — Source Data for Figure 3 [file EMMM-15-e17928-s008.zip › Figure_3/3I_STZ+AAV-PAX6_Insulin.tif]

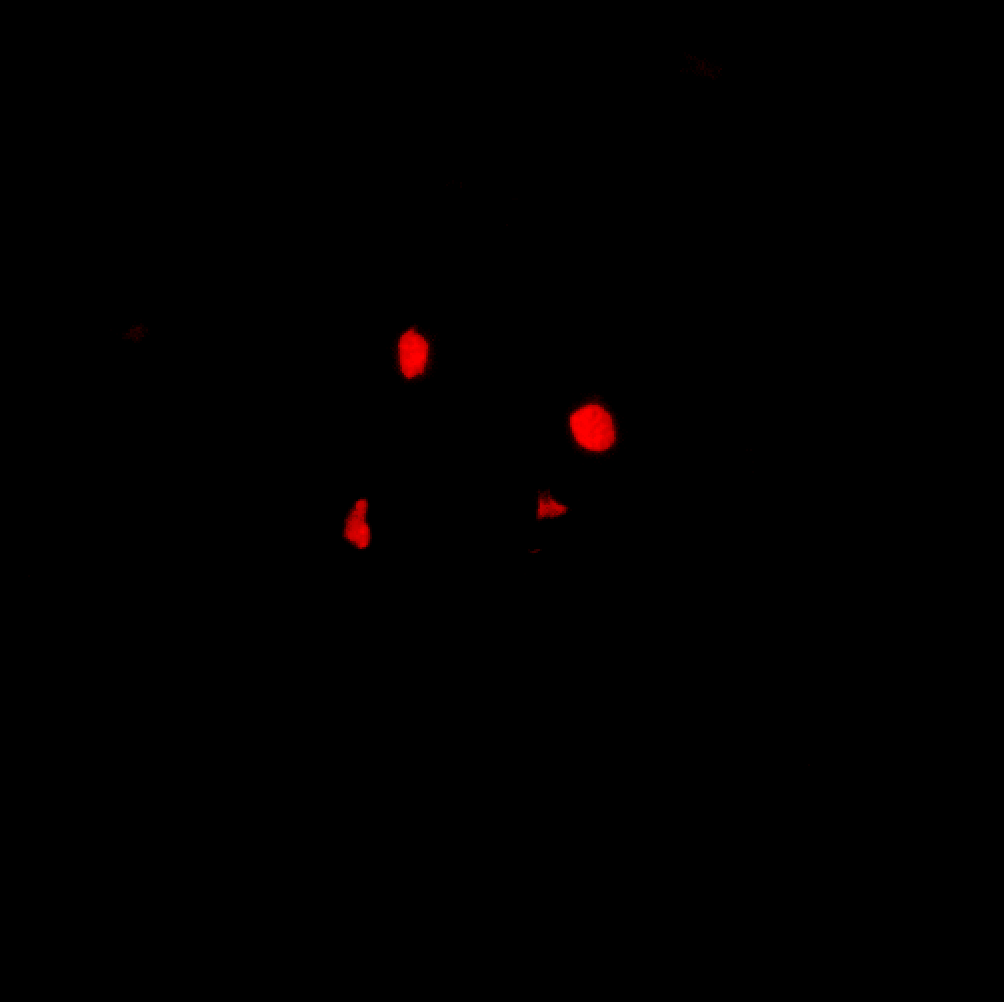

Supplement: Supplementary file 4 — Source Data for Figure 3 [file EMMM-15-e17928-s008.zip › Figure_3/3I_STZ+AAV-PAX6_Ki67.tif]

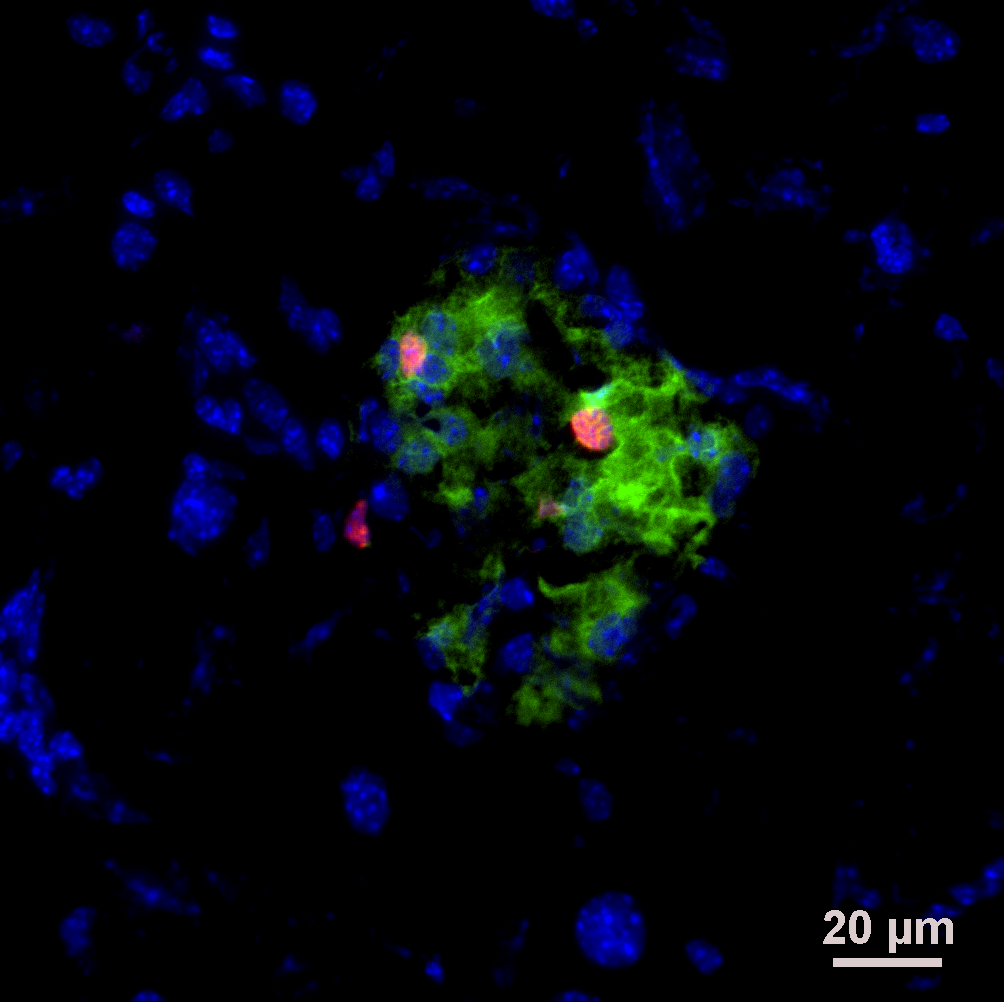

Supplement: Supplementary file 4 — Source Data for Figure 3 [file EMMM-15-e17928-s008.zip › Figure_3/3I_STZ+AAV-PAX6_Merged.tif]

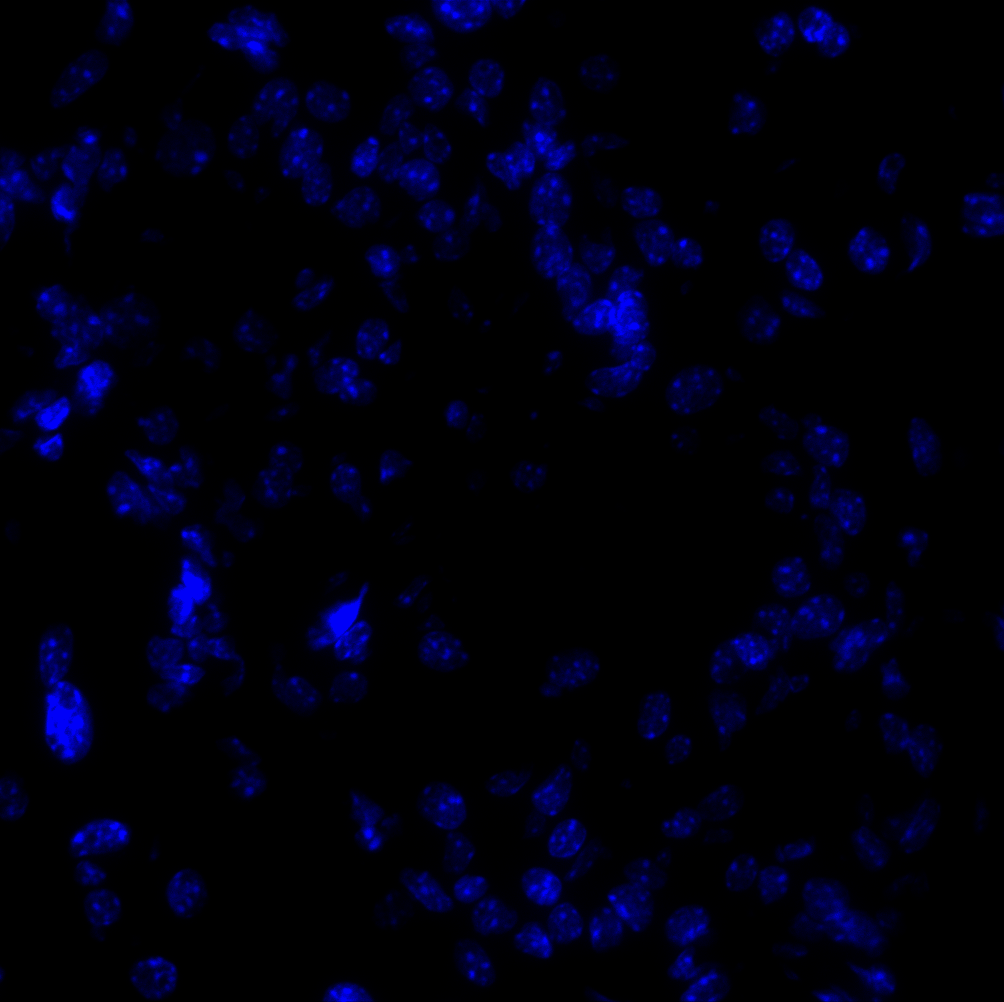

Supplement: Supplementary file 4 — Source Data for Figure 3 [file EMMM-15-e17928-s008.zip › Figure_3/3I_vehicle+AAV-Ctrl_DAPI.tif]

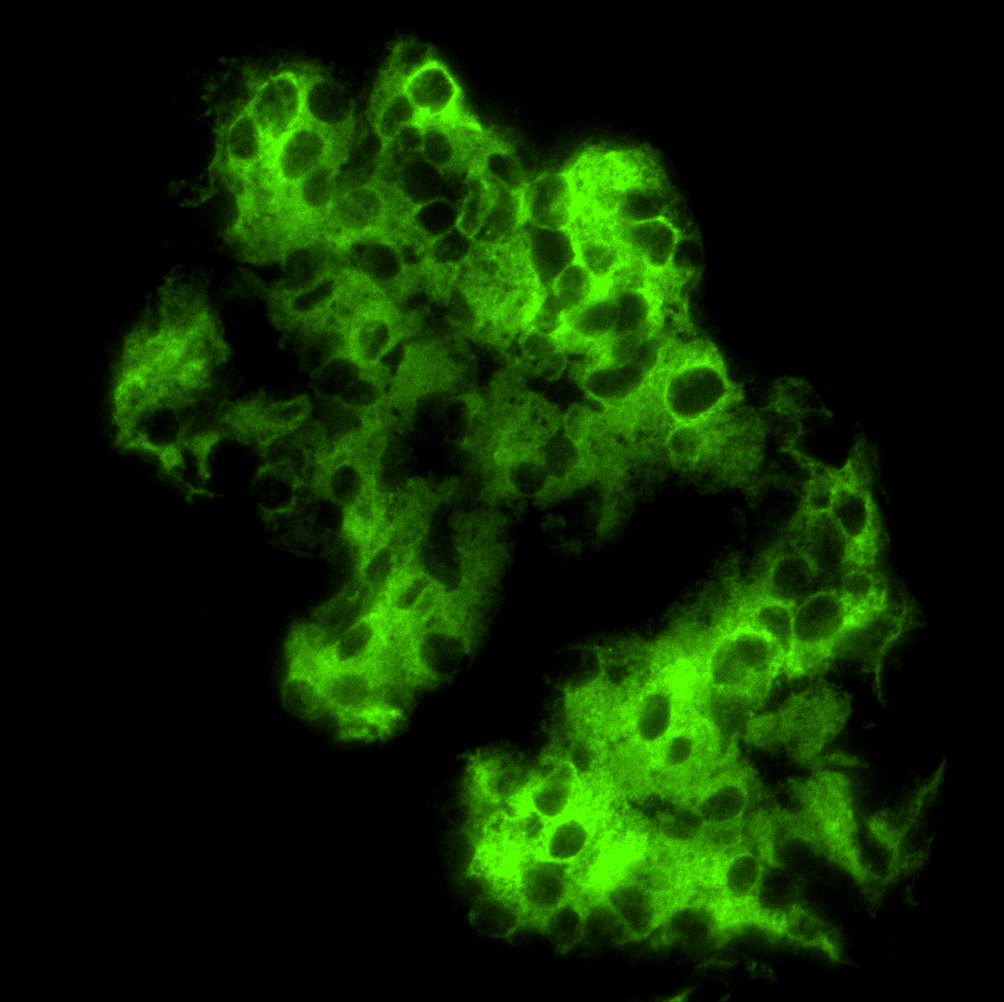

Supplement: Supplementary file 4 — Source Data for Figure 3 [file EMMM-15-e17928-s008.zip › Figure_3/3I_vehicle+AAV-Ctrl_Insulin.tif]

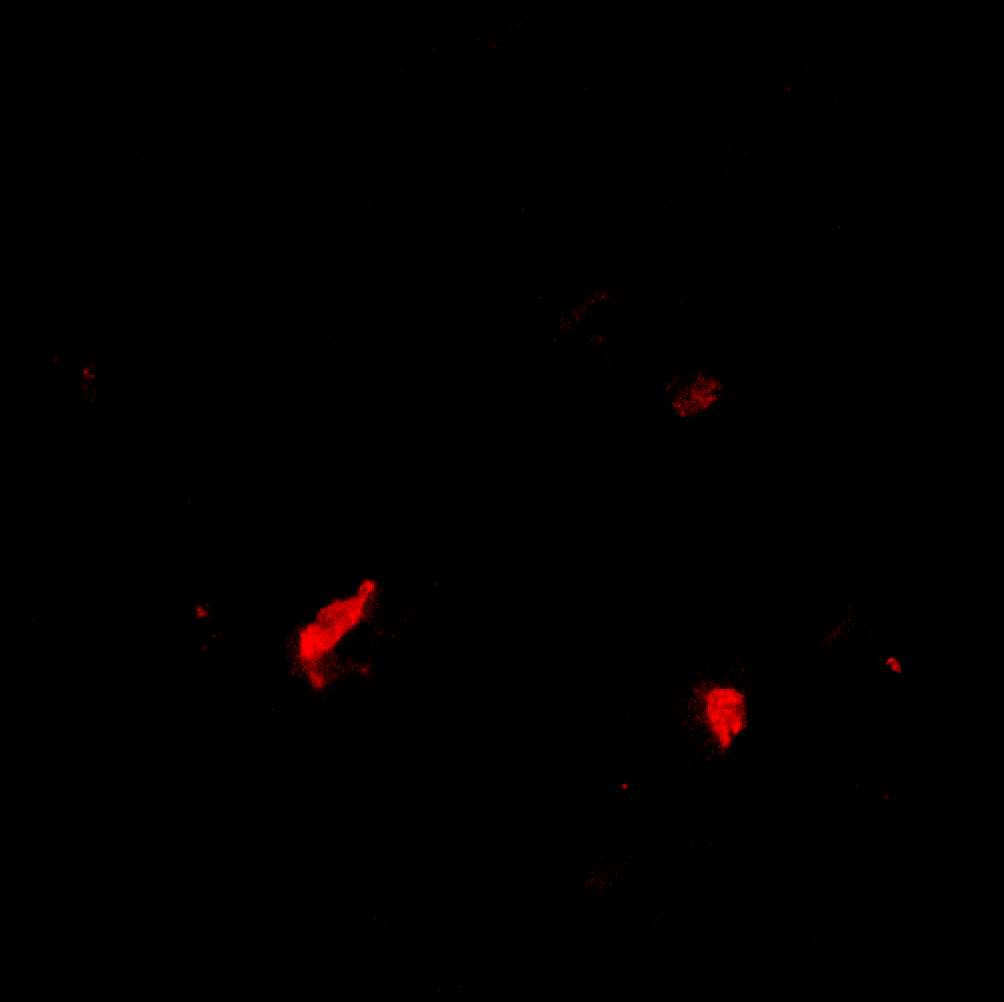

Supplement: Supplementary file 4 — Source Data for Figure 3 [file EMMM-15-e17928-s008.zip › Figure_3/3I_vehicle+AAV-Ctrl_Ki67.tif]

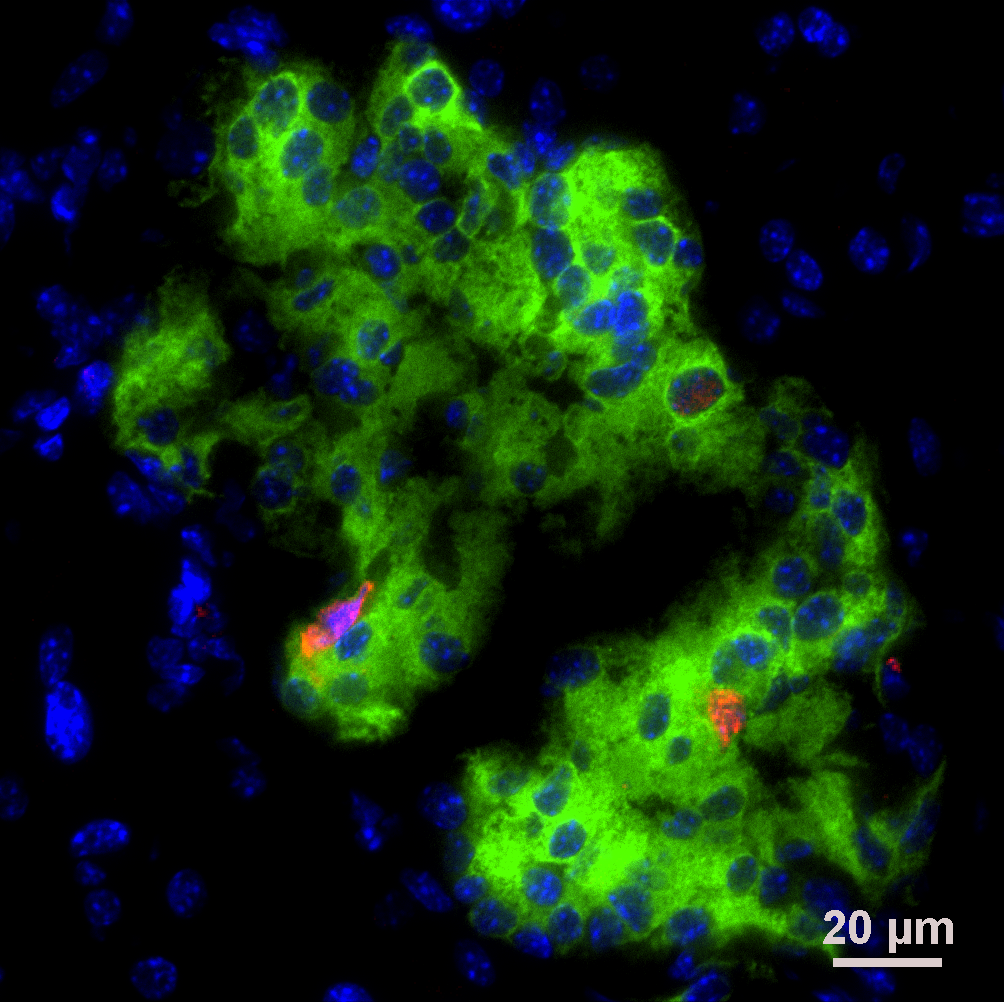

Supplement: Supplementary file 4 — Source Data for Figure 3 [file EMMM-15-e17928-s008.zip › Figure_3/3I_vehicle+AAV-Ctrl_Merged.tif]

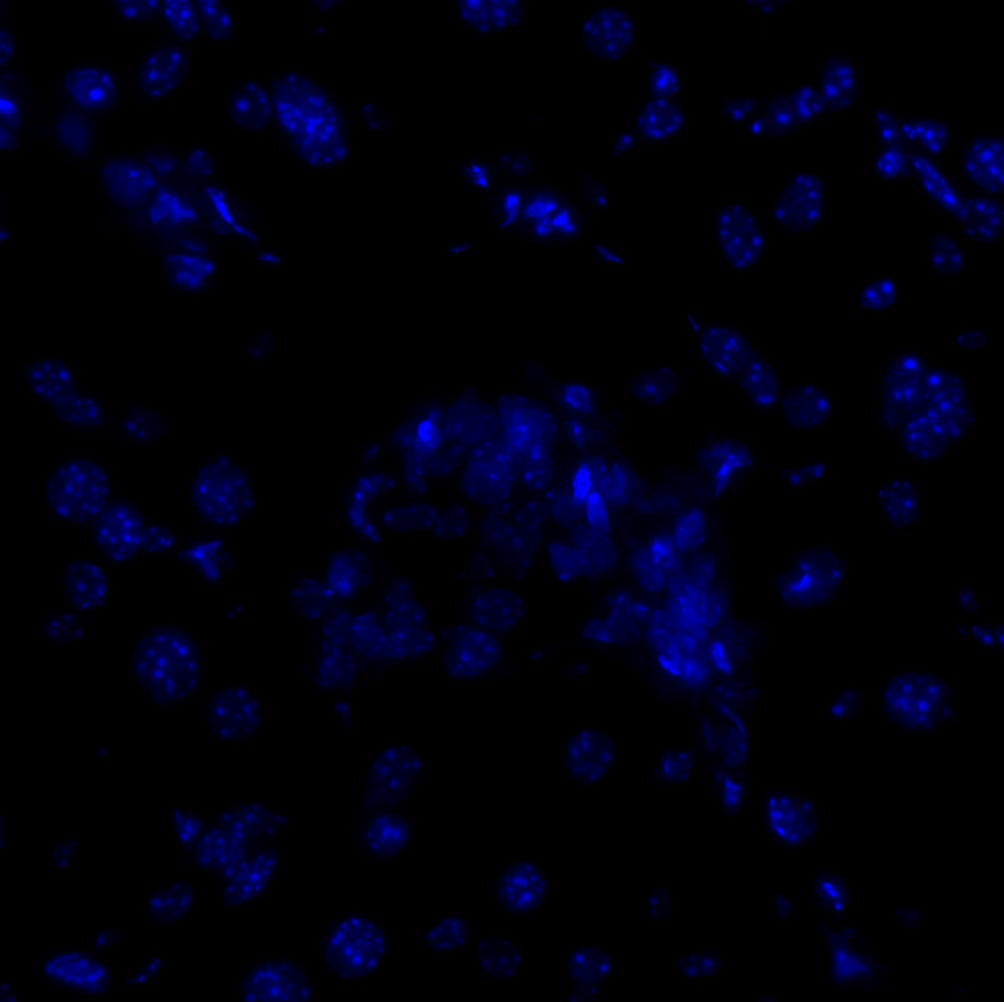

Supplement: Supplementary file 4 — Source Data for Figure 3 [file EMMM-15-e17928-s008.zip › Figure_3/3J_STZ+AAV-Ctrl_DAPI.tif]

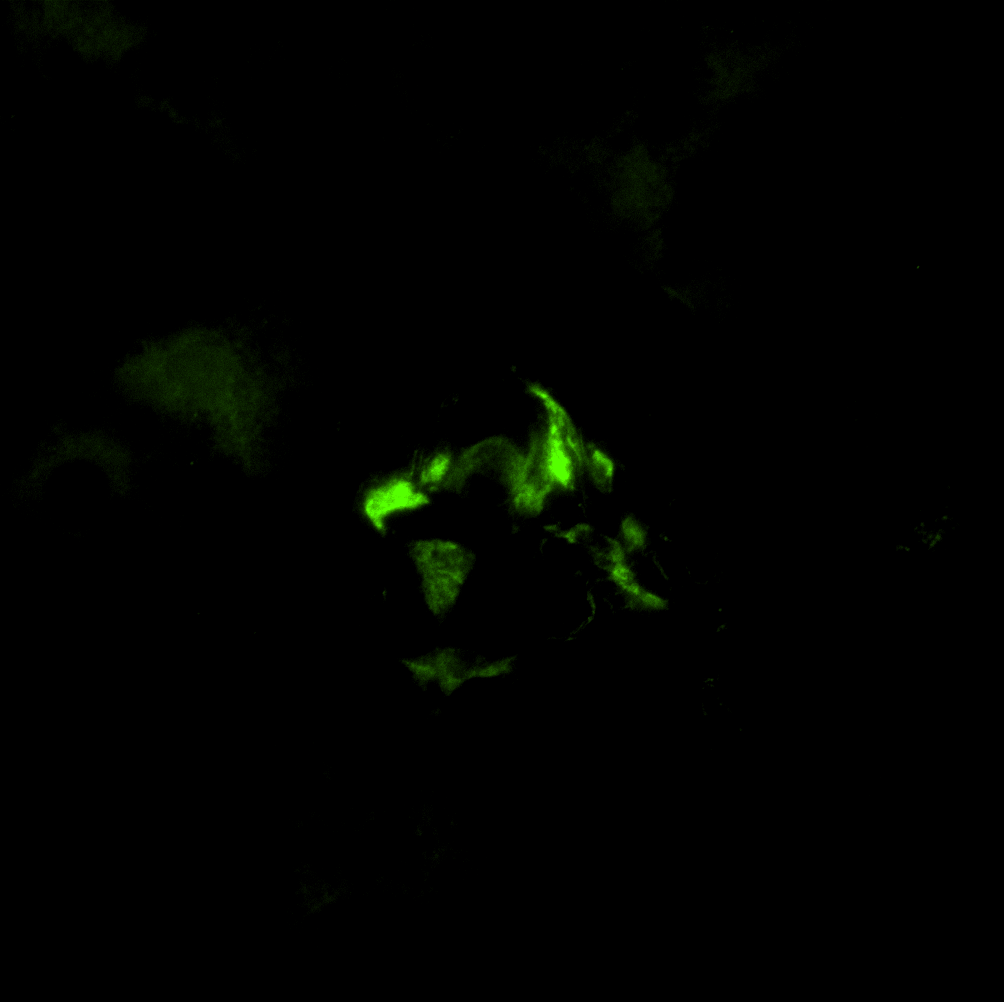

Supplement: Supplementary file 4 — Source Data for Figure 3 [file EMMM-15-e17928-s008.zip › Figure_3/3J_STZ+AAV-Ctrl_Insulin.tif]

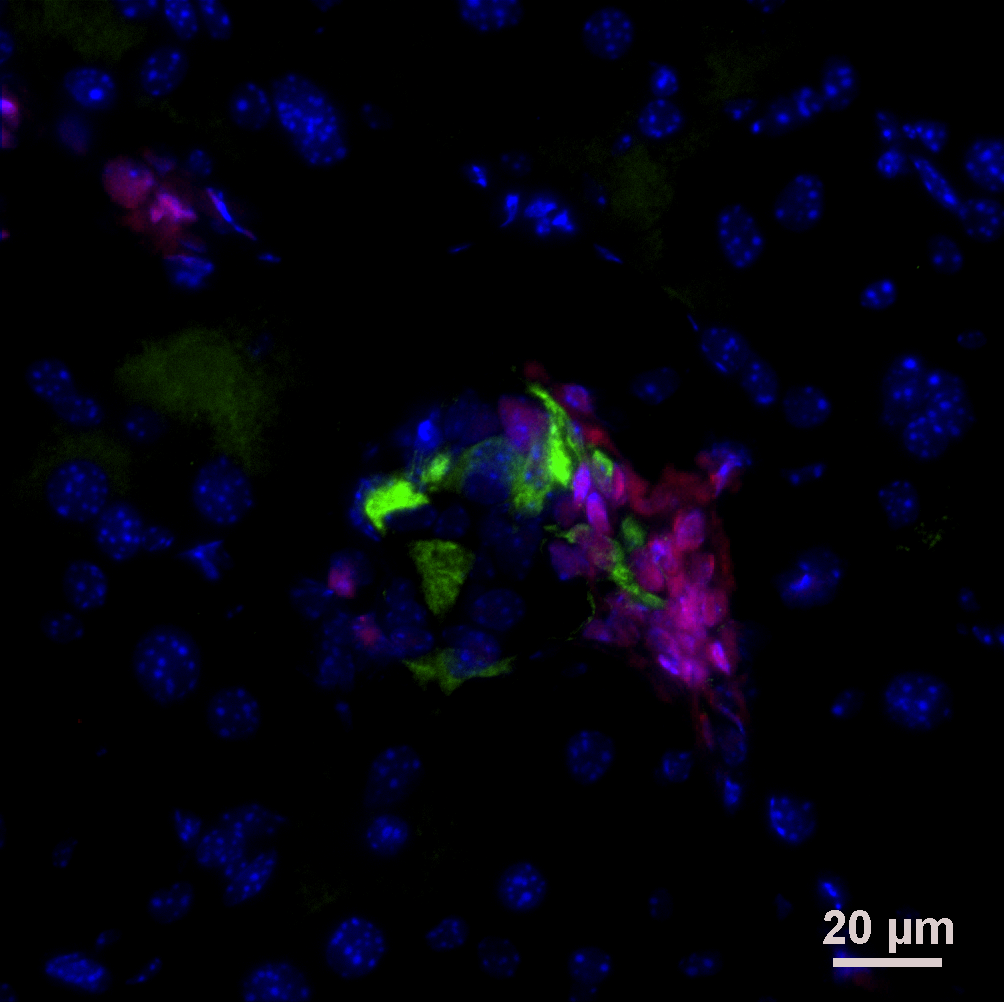

Supplement: Supplementary file 4 — Source Data for Figure 3 [file EMMM-15-e17928-s008.zip › Figure_3/3J_STZ+AAV-Ctrl_Merged.tif]

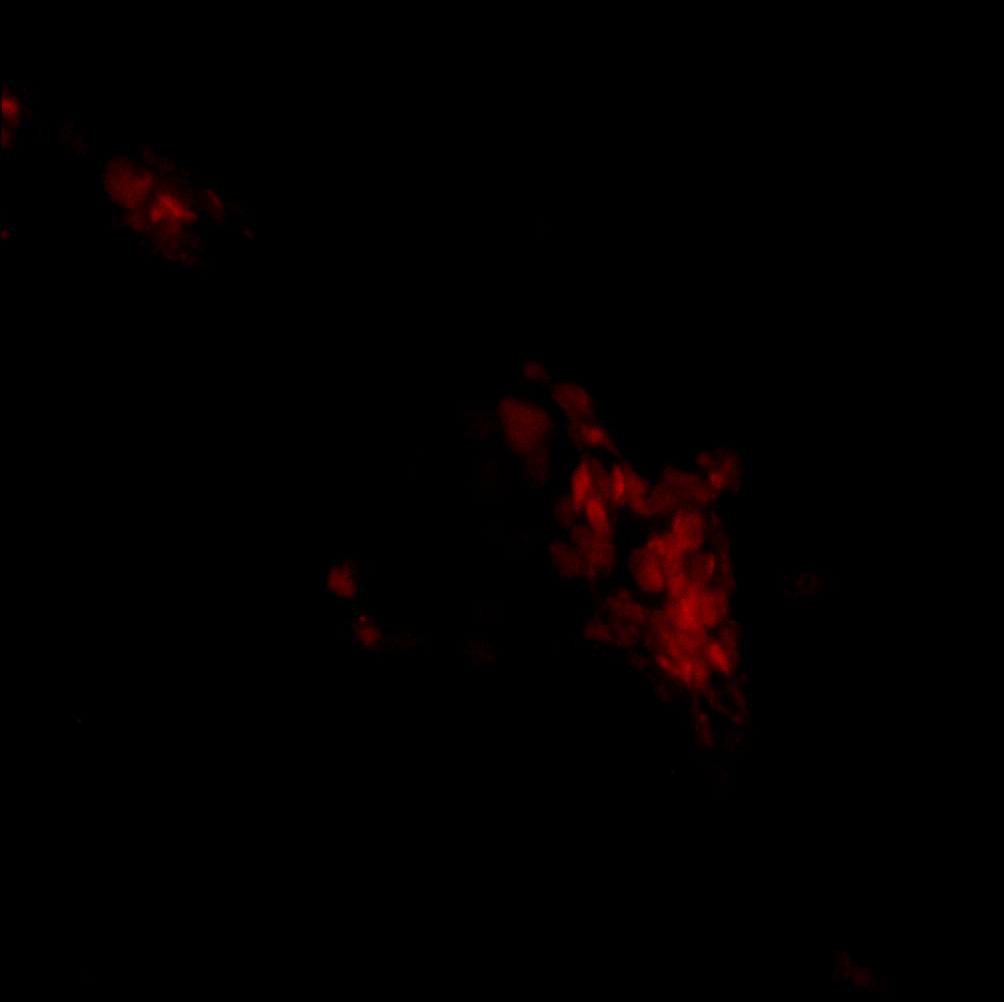

Supplement: Supplementary file 4 — Source Data for Figure 3 [file EMMM-15-e17928-s008.zip › Figure_3/3J_STZ+AAV-Ctrl_Tunel.tif]

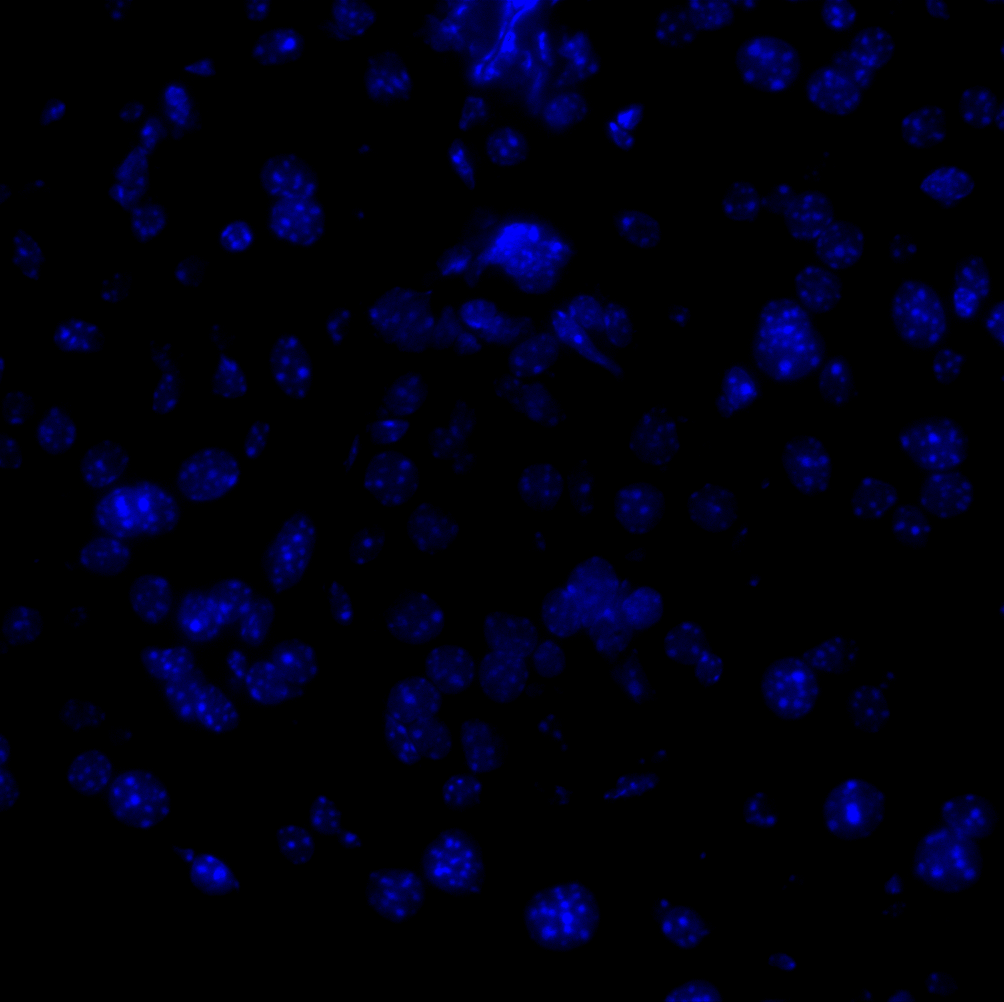

Supplement: Supplementary file 4 — Source Data for Figure 3 [file EMMM-15-e17928-s008.zip › Figure_3/3J_STZ+AAV-PAX6_DAPI.tif]

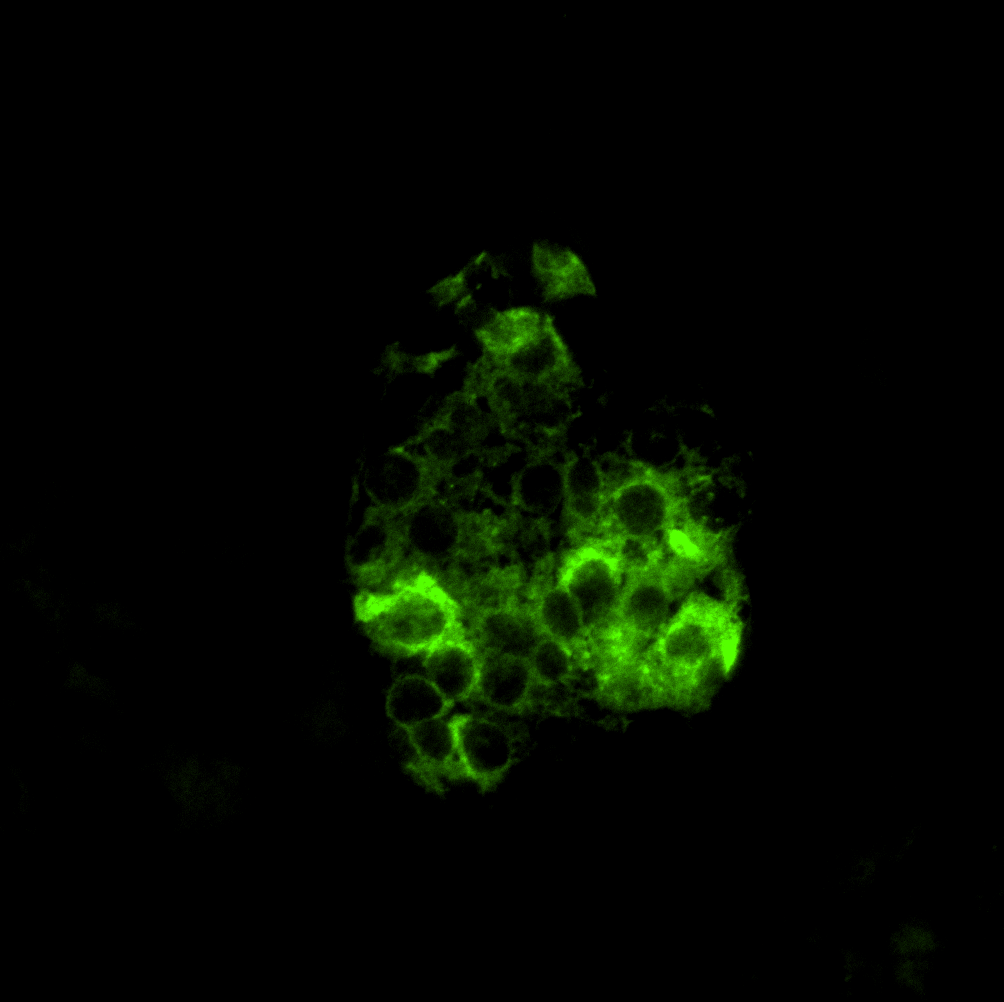

Supplement: Supplementary file 4 — Source Data for Figure 3 [file EMMM-15-e17928-s008.zip › Figure_3/3J_STZ+AAV-PAX6_Insulin.tif]

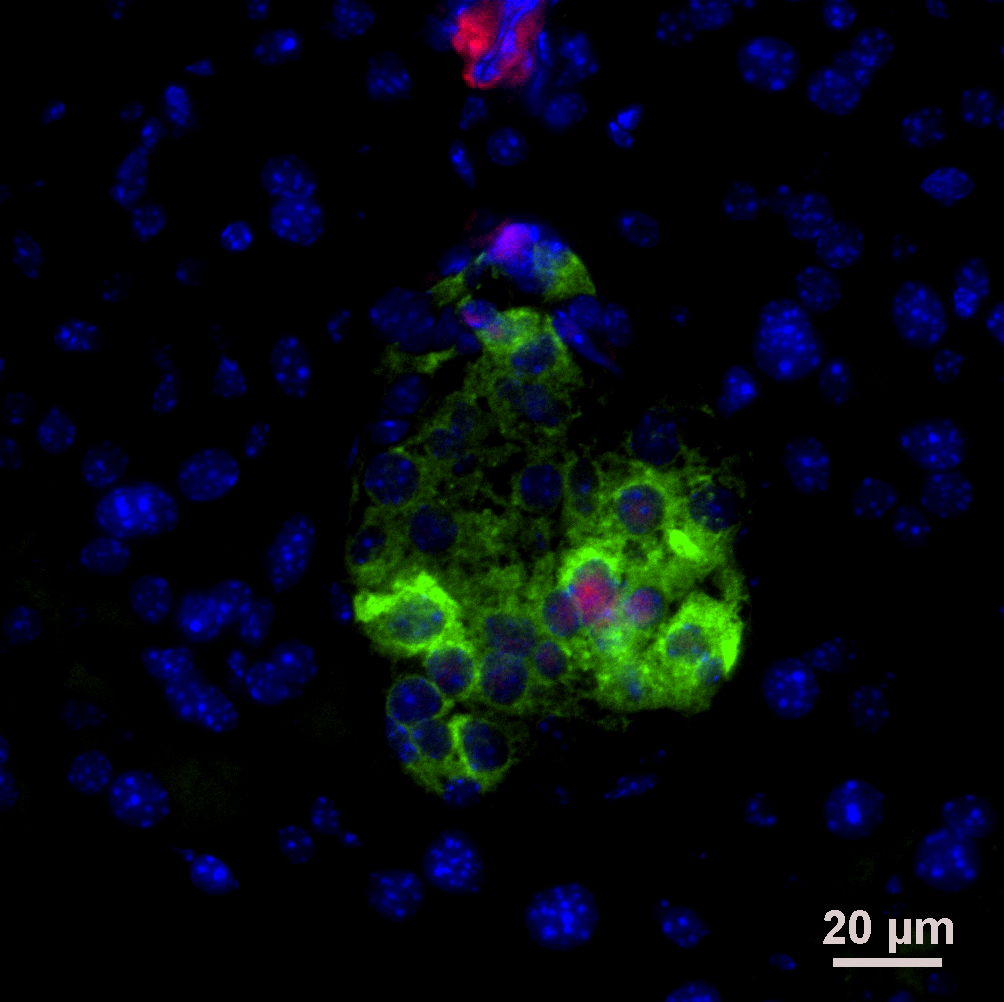

Supplement: Supplementary file 4 — Source Data for Figure 3 [file EMMM-15-e17928-s008.zip › Figure_3/3J_STZ+AAV-PAX6_Merged.tif]

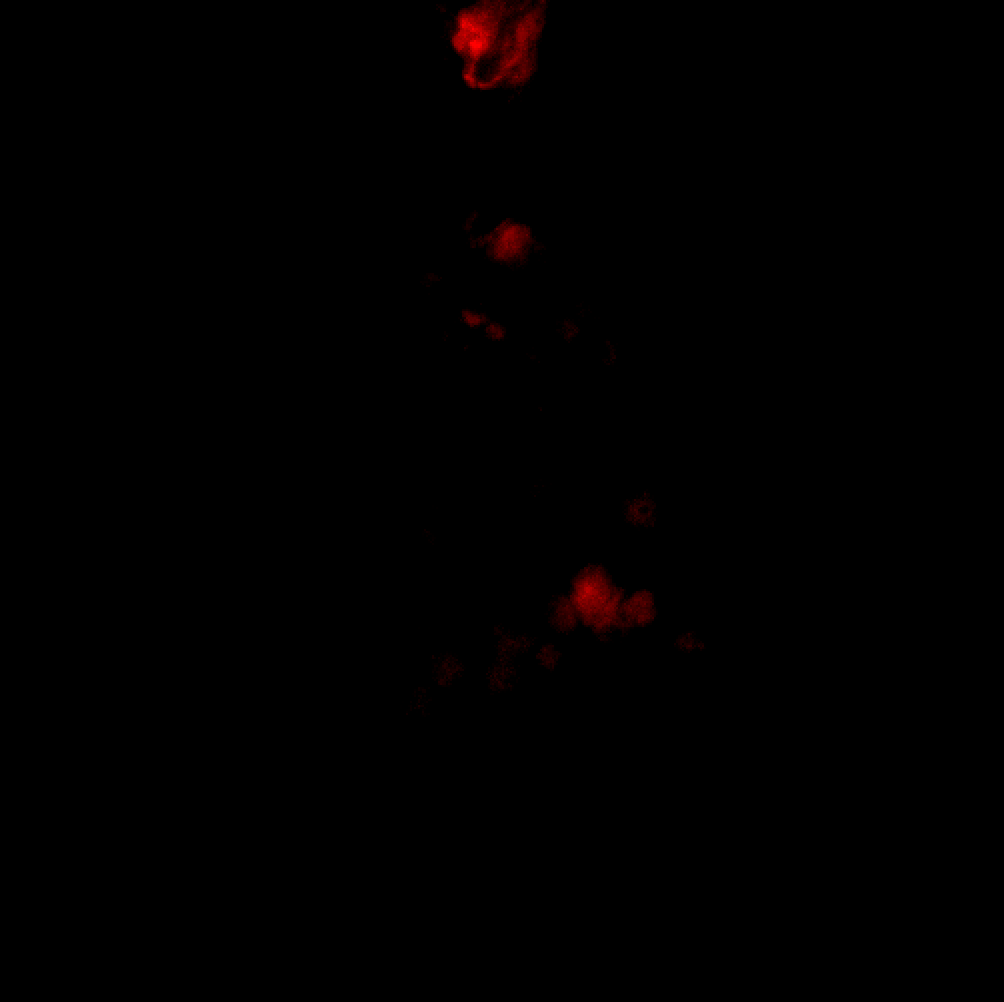

Supplement: Supplementary file 4 — Source Data for Figure 3 [file EMMM-15-e17928-s008.zip › Figure_3/3J_STZ+AAV-PAX6_Tunel.tif]

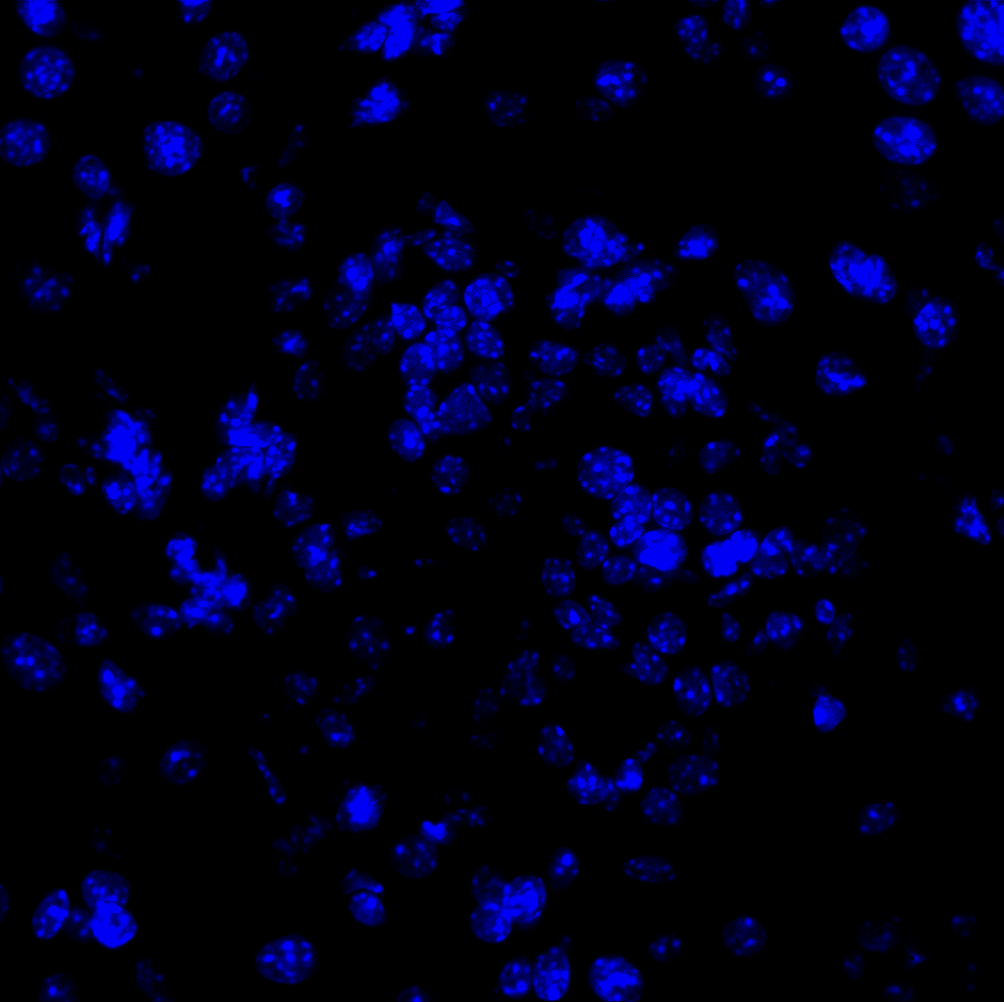

Supplement: Supplementary file 4 — Source Data for Figure 3 [file EMMM-15-e17928-s008.zip › Figure_3/3J_vehicle+AAV-Ctrl_DAPI.tif]

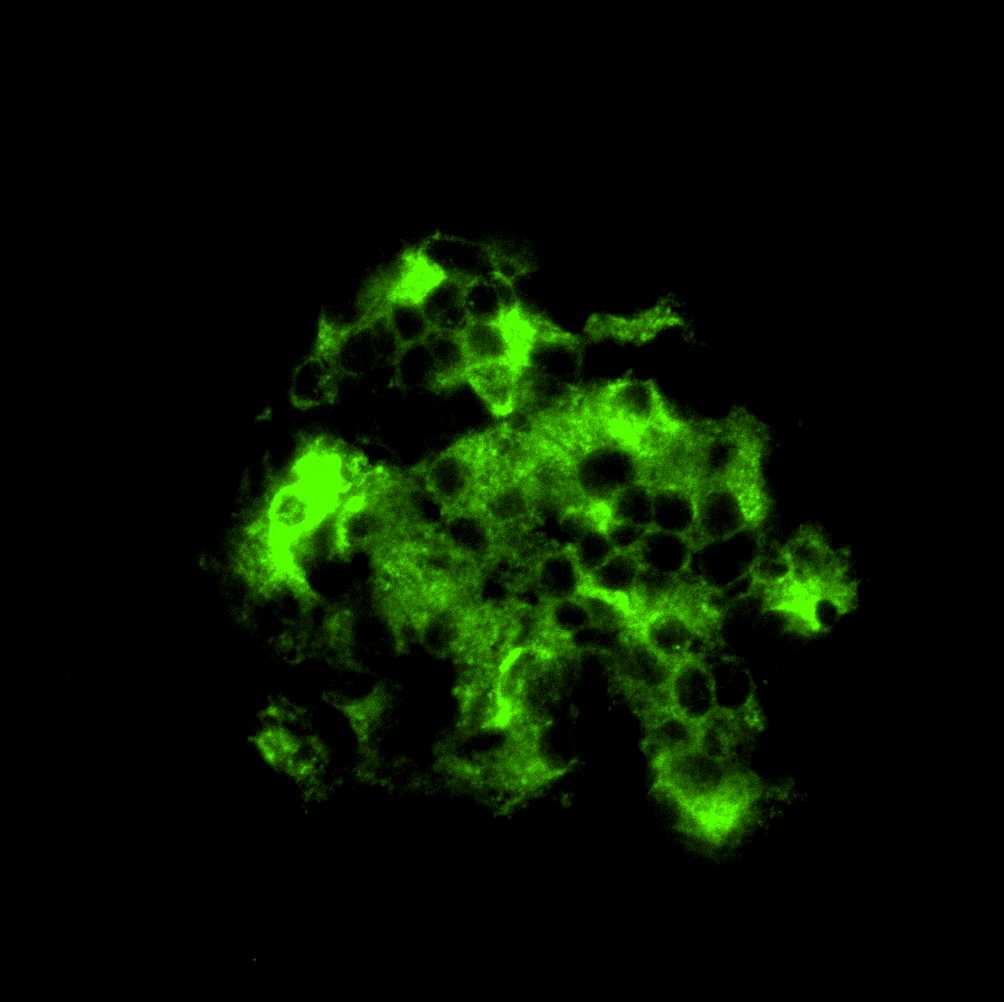

Supplement: Supplementary file 4 — Source Data for Figure 3 [file EMMM-15-e17928-s008.zip › Figure_3/3J_vehicle+AAV-Ctrl_Insulin.tif]

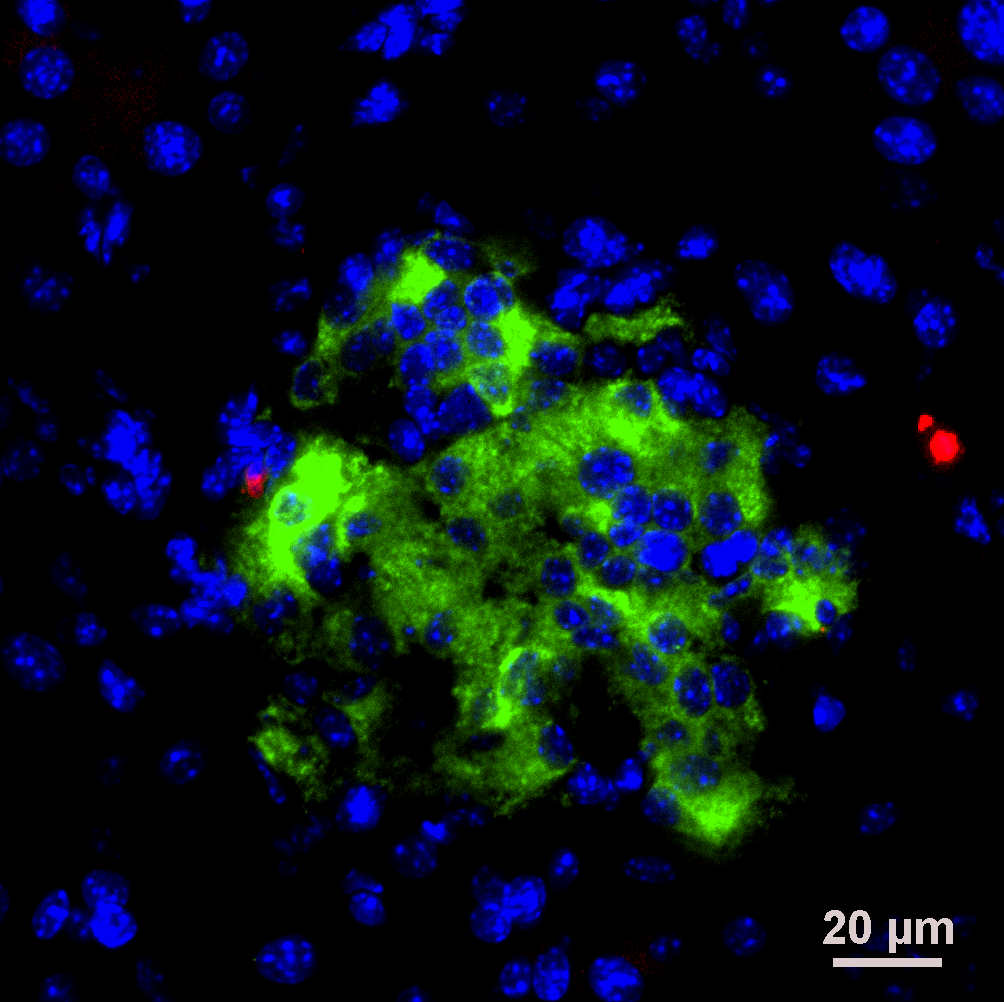

Supplement: Supplementary file 4 — Source Data for Figure 3 [file EMMM-15-e17928-s008.zip › Figure_3/3J_vehicle+AAV-Ctrl_Merged.tif]

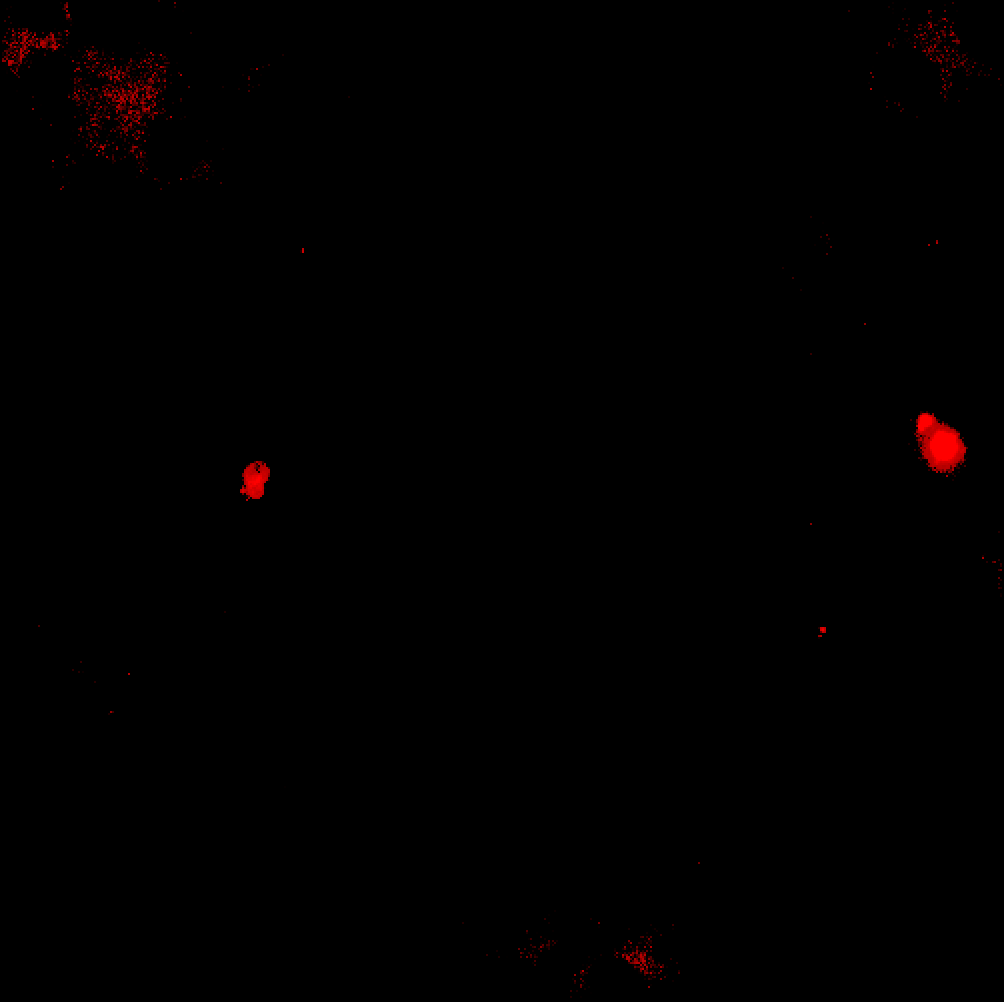

Supplement: Supplementary file 4 — Source Data for Figure 3 [file EMMM-15-e17928-s008.zip › Figure_3/3J_vehicle+AAV-Ctrl_Tunel.tif]

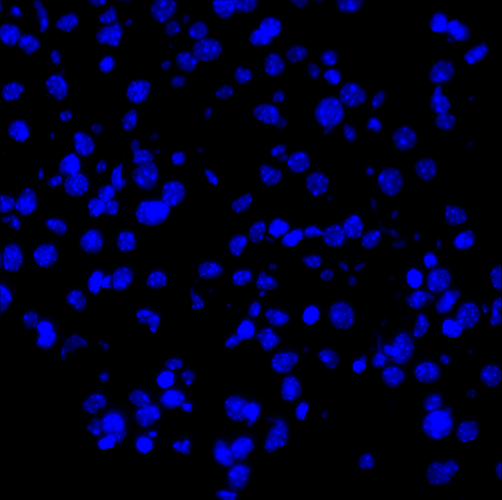

Supplement: Supplementary file 5 — Source Data for Figure 4 [file EMMM-15-e17928-s003.zip › Figure_4/4I_db++AAV-Ctrl_DAPI.tif]

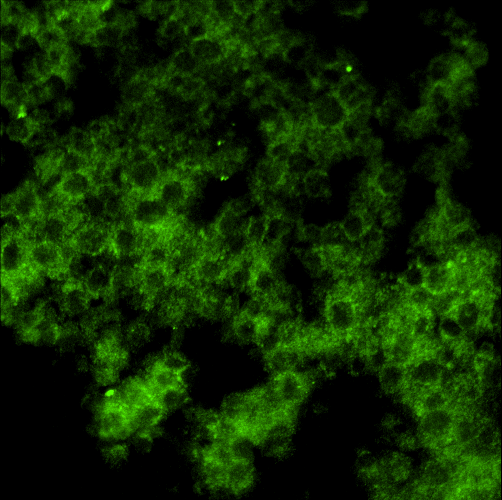

Supplement: Supplementary file 5 — Source Data for Figure 4 [file EMMM-15-e17928-s003.zip › Figure_4/4I_db++AAV-Ctrl_Insulin.tif]

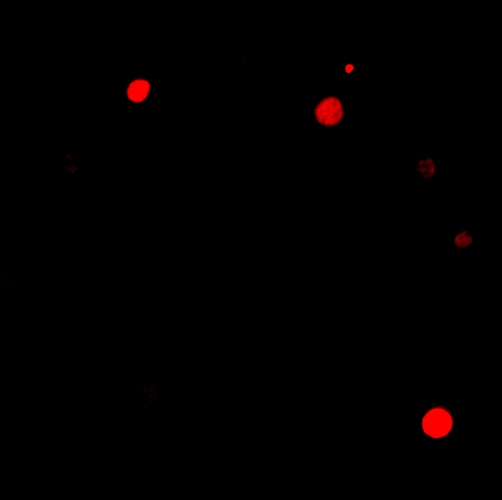

Supplement: Supplementary file 5 — Source Data for Figure 4 [file EMMM-15-e17928-s003.zip › Figure_4/4I_db++AAV-Ctrl_Ki67.tif]

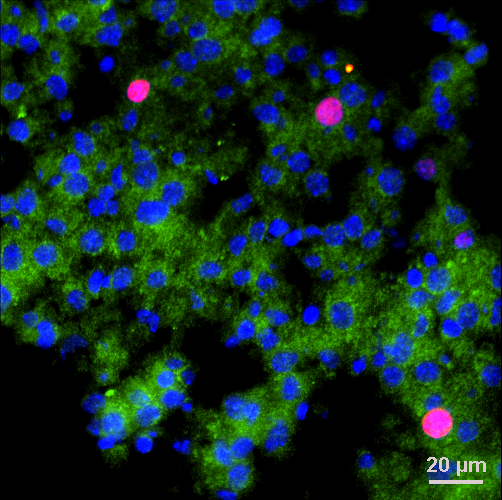

Supplement: Supplementary file 5 — Source Data for Figure 4 [file EMMM-15-e17928-s003.zip › Figure_4/4I_db++AAV-Ctrl_Merged.tif]

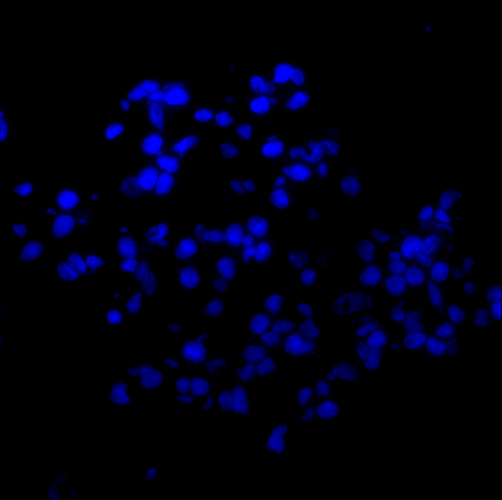

Supplement: Supplementary file 5 — Source Data for Figure 4 [file EMMM-15-e17928-s003.zip › Figure_4/4I_dbdb+AAV-Ctrl_DAPI.tif]

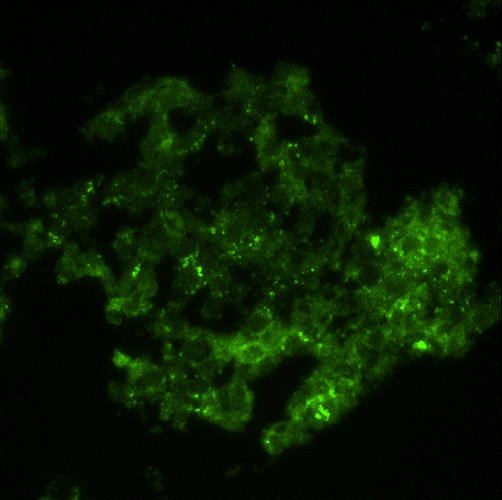

Supplement: Supplementary file 5 — Source Data for Figure 4 [file EMMM-15-e17928-s003.zip › Figure_4/4I_dbdb+AAV-Ctrl_Insulin.tif]

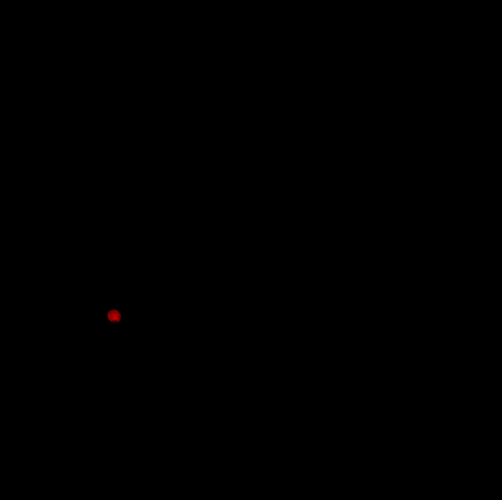

Supplement: Supplementary file 5 — Source Data for Figure 4 [file EMMM-15-e17928-s003.zip › Figure_4/4I_dbdb+AAV-Ctrl_Ki67.tif]

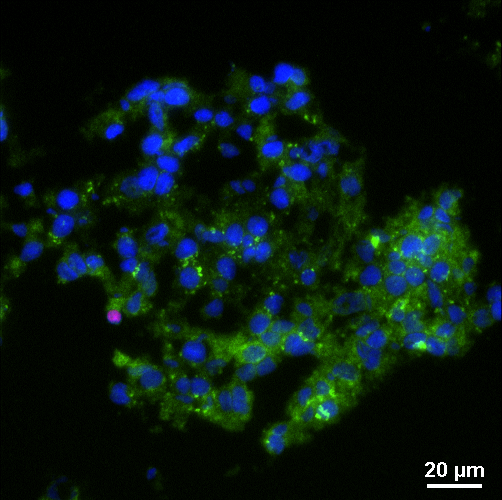

Supplement: Supplementary file 5 — Source Data for Figure 4 [file EMMM-15-e17928-s003.zip › Figure_4/4I_dbdb+AAV-Ctrl_Merged.tif]

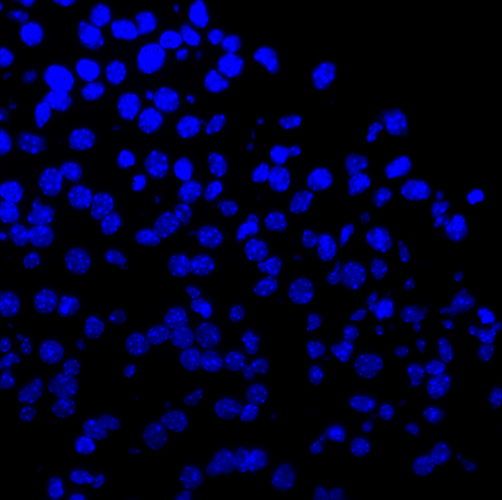

Supplement: Supplementary file 5 — Source Data for Figure 4 [file EMMM-15-e17928-s003.zip › Figure_4/4I_dbdb+AAV-PAX6_DAPI.tif]

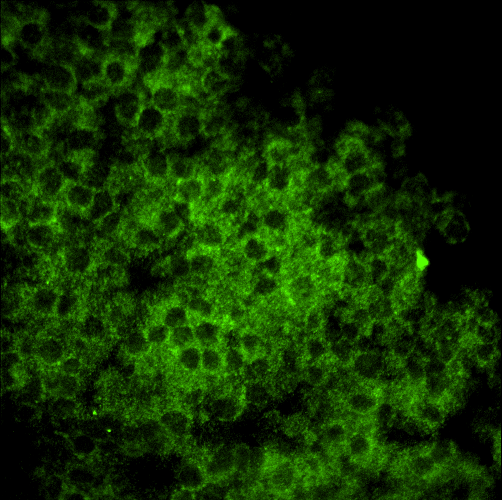

Supplement: Supplementary file 5 — Source Data for Figure 4 [file EMMM-15-e17928-s003.zip › Figure_4/4I_dbdb+AAV-PAX6_Insulin.tif]

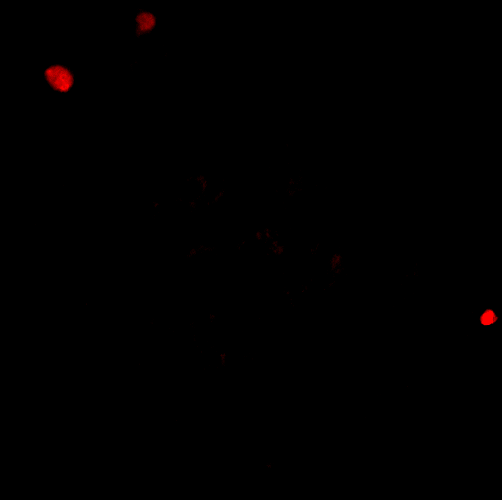

Supplement: Supplementary file 5 — Source Data for Figure 4 [file EMMM-15-e17928-s003.zip › Figure_4/4I_dbdb+AAV-PAX6_Ki67.tif]

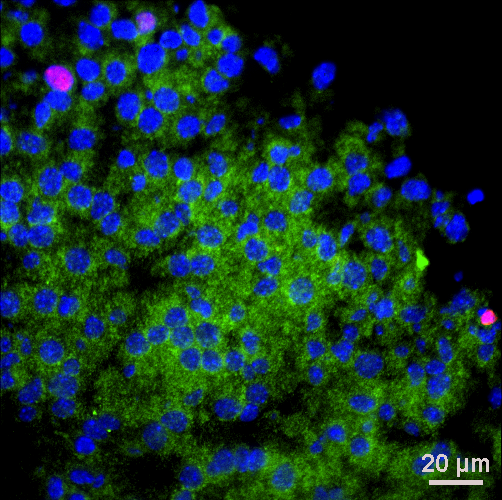

Supplement: Supplementary file 5 — Source Data for Figure 4 [file EMMM-15-e17928-s003.zip › Figure_4/4I_dbdb+AAV-PAX6_Merged.tif]

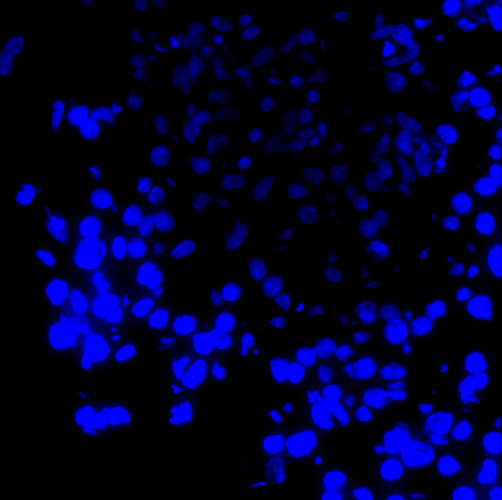

Supplement: Supplementary file 5 — Source Data for Figure 4 [file EMMM-15-e17928-s003.zip › Figure_4/4J_db++AAV-Ctrl_DAPI.tif]

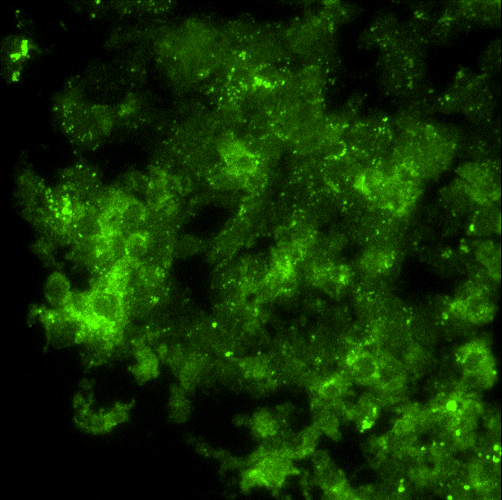

Supplement: Supplementary file 5 — Source Data for Figure 4 [file EMMM-15-e17928-s003.zip › Figure_4/4J_db++AAV-Ctrl_Insulin.tif]

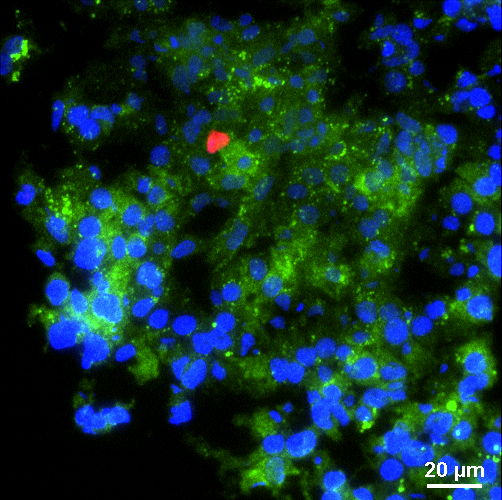

Supplement: Supplementary file 5 — Source Data for Figure 4 [file EMMM-15-e17928-s003.zip › Figure_4/4J_db++AAV-Ctrl_Merged.tif]

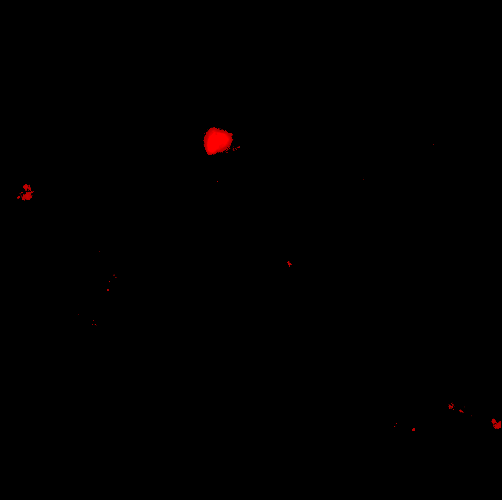

Supplement: Supplementary file 5 — Source Data for Figure 4 [file EMMM-15-e17928-s003.zip › Figure_4/4J_db++AAV-Ctrl_Tunel.tif]

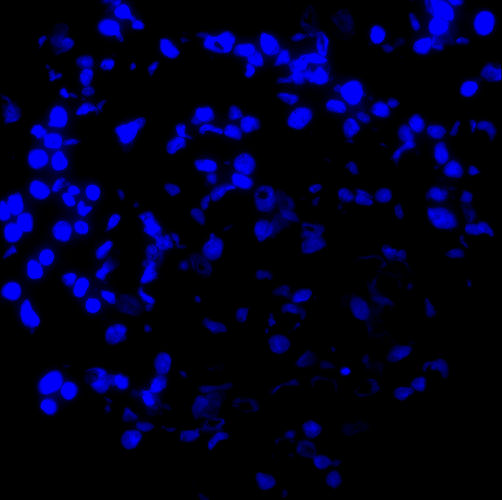

Supplement: Supplementary file 5 — Source Data for Figure 4 [file EMMM-15-e17928-s003.zip › Figure_4/4J_dbdb+AAV-Ctrl_DAPI.tif]

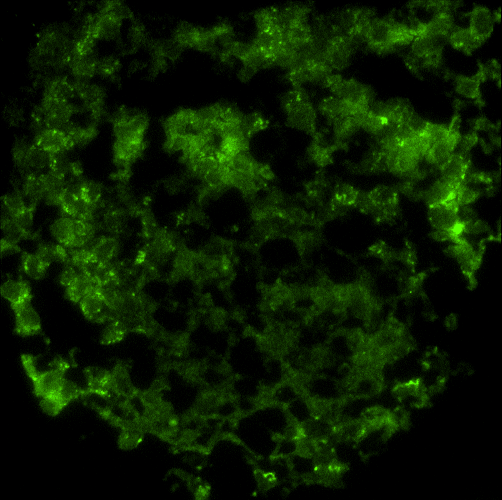

Supplement: Supplementary file 5 — Source Data for Figure 4 [file EMMM-15-e17928-s003.zip › Figure_4/4J_dbdb+AAV-Ctrl_Insulin.tif]

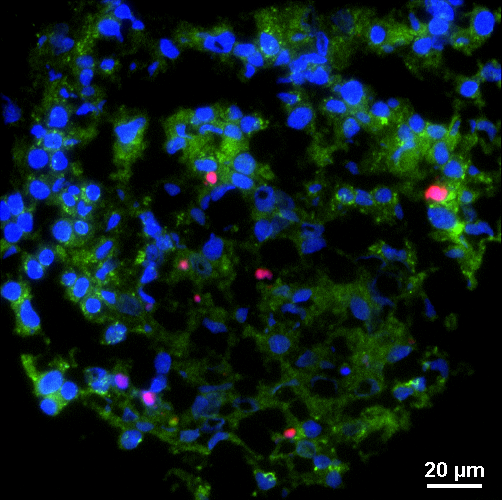

Supplement: Supplementary file 5 — Source Data for Figure 4 [file EMMM-15-e17928-s003.zip › Figure_4/4J_dbdb+AAV-Ctrl_Merged.tif]

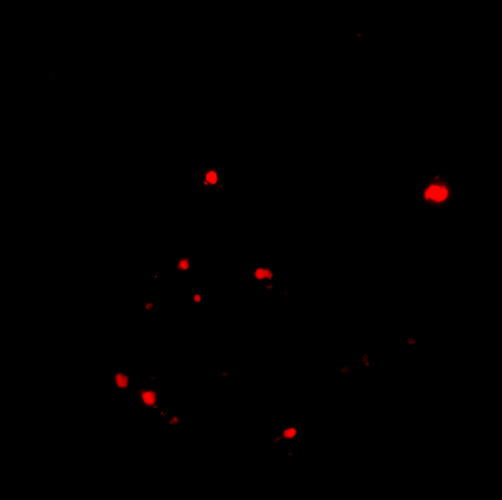

Supplement: Supplementary file 5 — Source Data for Figure 4 [file EMMM-15-e17928-s003.zip › Figure_4/4J_dbdb+AAV-Ctrl_Tunel.tif]

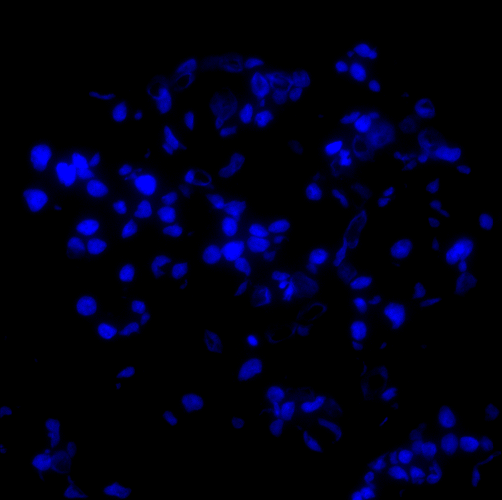

Supplement: Supplementary file 5 — Source Data for Figure 4 [file EMMM-15-e17928-s003.zip › Figure_4/4J_dbdb+AAV-PAX6_DAPI.tif]

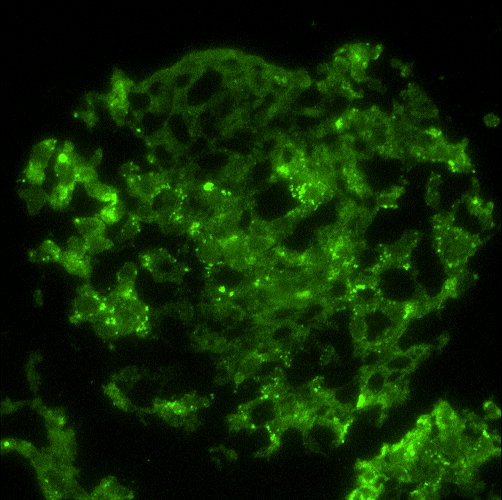

Supplement: Supplementary file 5 — Source Data for Figure 4 [file EMMM-15-e17928-s003.zip › Figure_4/4J_dbdb+AAV-PAX6_Insulin.tif]

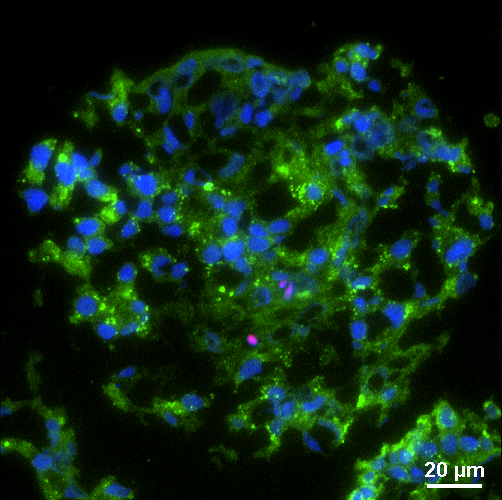

Supplement: Supplementary file 5 — Source Data for Figure 4 [file EMMM-15-e17928-s003.zip › Figure_4/4J_dbdb+AAV-PAX6_Merged.tif]

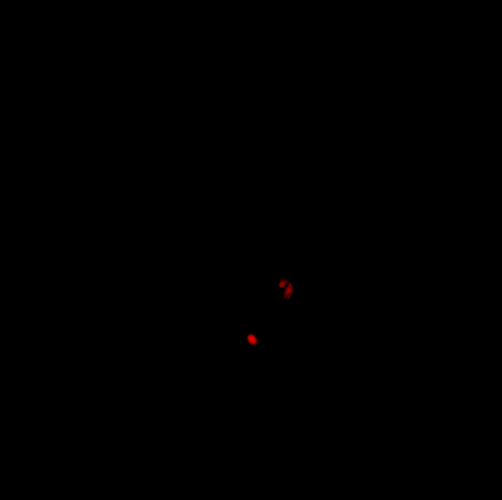

Supplement: Supplementary file 5 — Source Data for Figure 4 [file EMMM-15-e17928-s003.zip › Figure_4/4J_dbdb+AAV-PAX6_Tunel.tif]

**K**

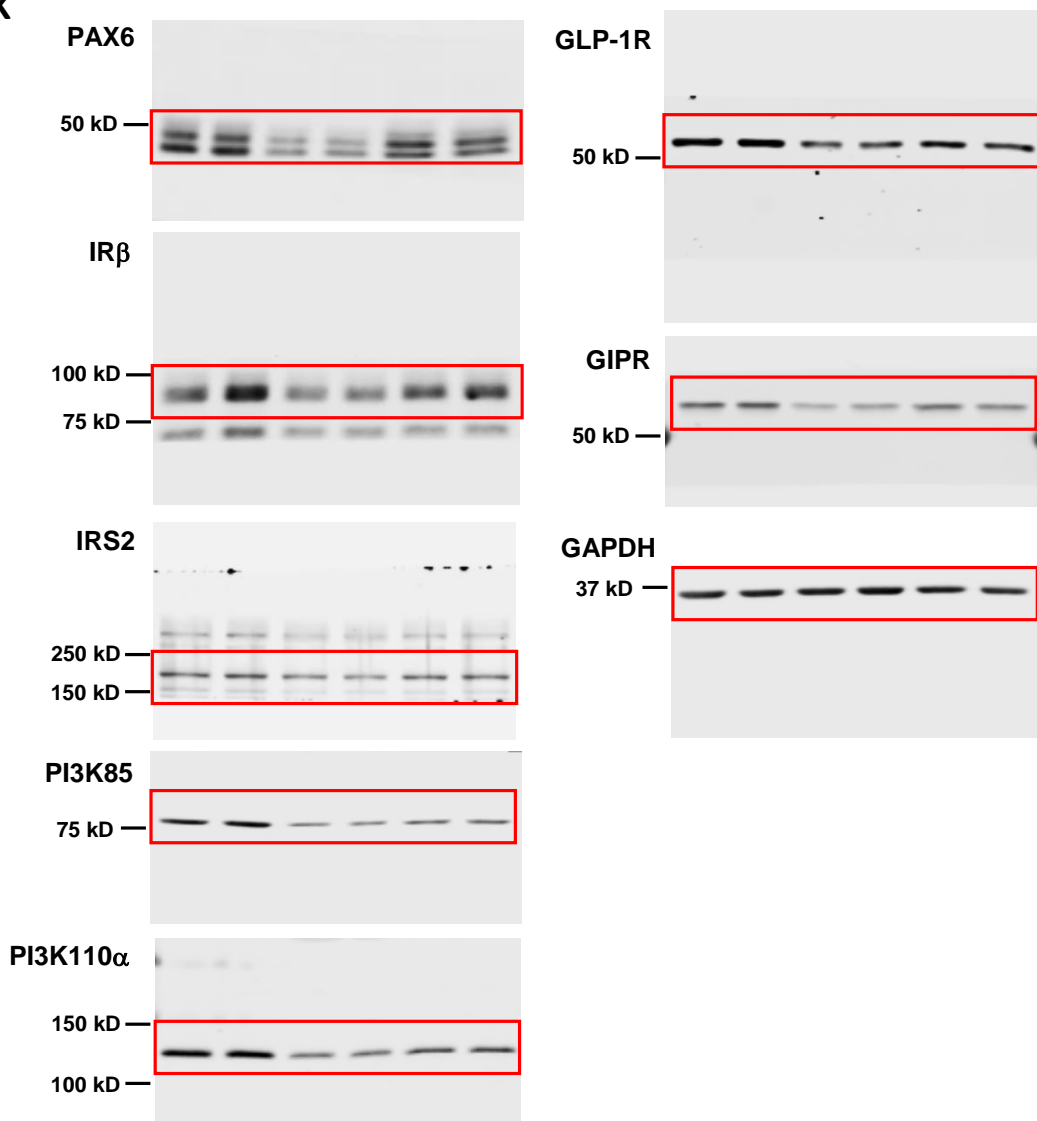

Supplement: Supplementary file 5 — Source Data for Figure 4 [file EMMM-15-e17928-s003.zip › Figure_4/Figure_4_western.pdf]

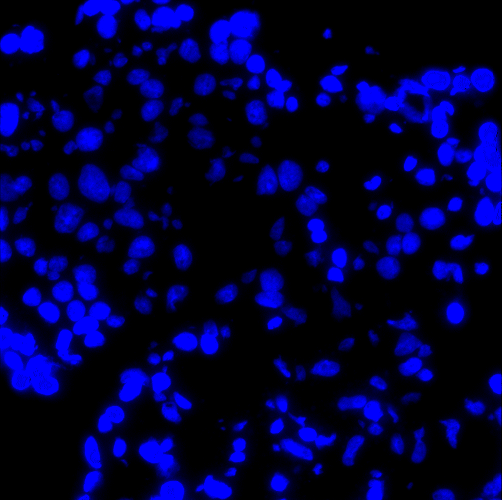

Supplement: Supplementary file 6 — Source Data for Figure 5 [file EMMM-15-e17928-s001.zip › Figure_5/5C_Normal+AAV-Ctrl_DAPI.tif]

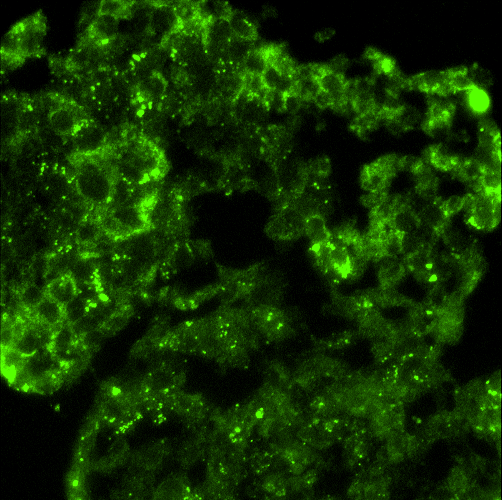

Supplement: Supplementary file 6 — Source Data for Figure 5 [file EMMM-15-e17928-s001.zip › Figure_5/5C_Normal+AAV-Ctrl_Insulin.tif]

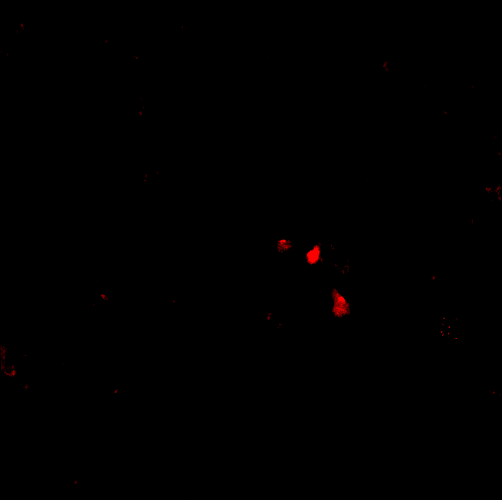

Supplement: Supplementary file 6 — Source Data for Figure 5 [file EMMM-15-e17928-s001.zip › Figure_5/5C_Normal+AAV-Ctrl_Ki67.tif]

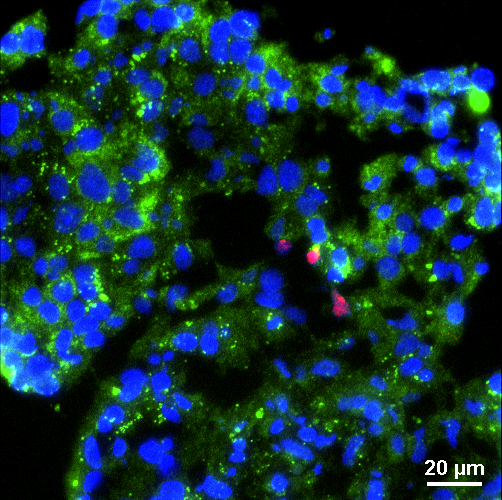

Supplement: Supplementary file 6 — Source Data for Figure 5 [file EMMM-15-e17928-s001.zip › Figure_5/5C_Normal+AAV-Ctrl_Merged.tif]

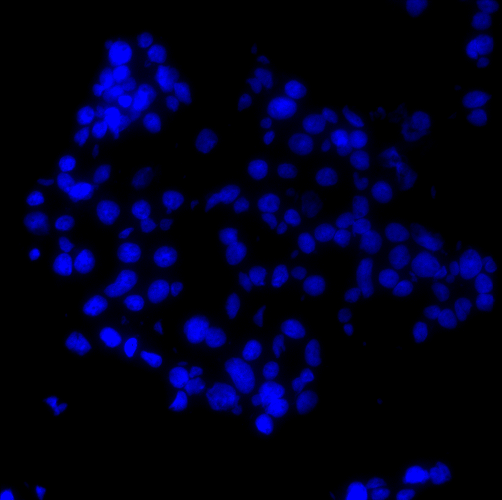

Supplement: Supplementary file 6 — Source Data for Figure 5 [file EMMM-15-e17928-s001.zip › Figure_5/5C_T2D+AAV-Ctrl_DAPI.tif]

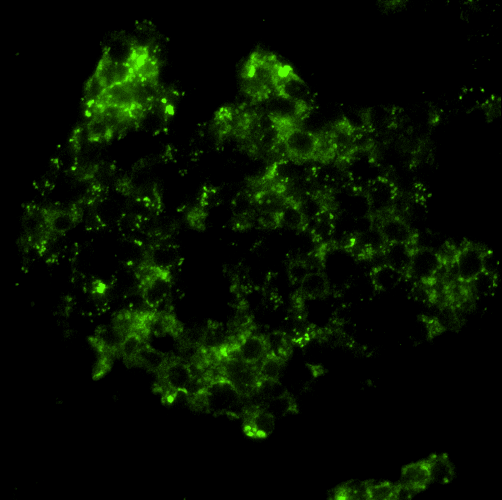

Supplement: Supplementary file 6 — Source Data for Figure 5 [file EMMM-15-e17928-s001.zip › Figure_5/5C_T2D+AAV-Ctrl_Insulin.tif]

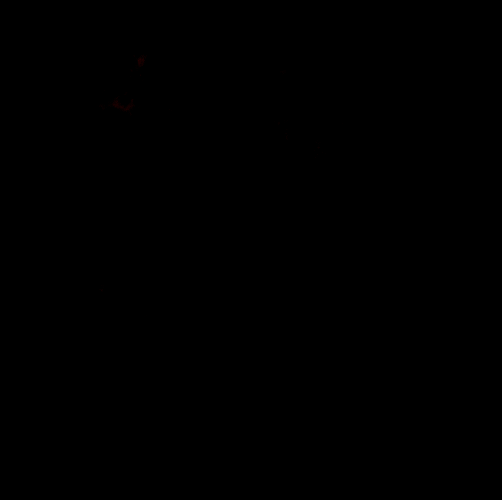

Supplement: Supplementary file 6 — Source Data for Figure 5 [file EMMM-15-e17928-s001.zip › Figure_5/5C_T2D+AAV-Ctrl_Ki67.tif]

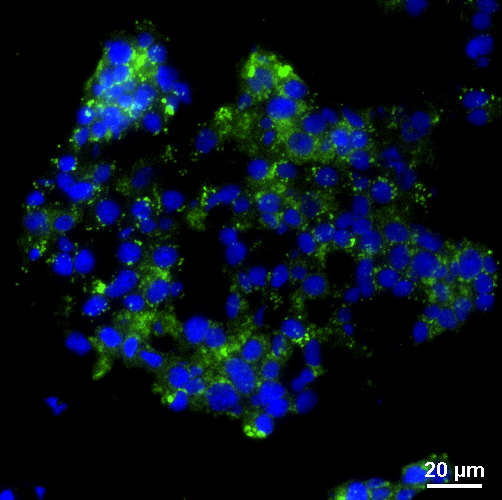

Supplement: Supplementary file 6 — Source Data for Figure 5 [file EMMM-15-e17928-s001.zip › Figure_5/5C_T2D+AAV-Ctrl_Merged.tif]

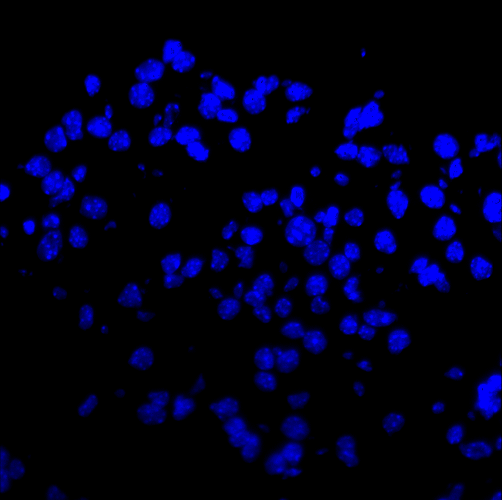

Supplement: Supplementary file 6 — Source Data for Figure 5 [file EMMM-15-e17928-s001.zip › Figure_5/5C_T2D+AAV-PAX6_DAPI.tif]

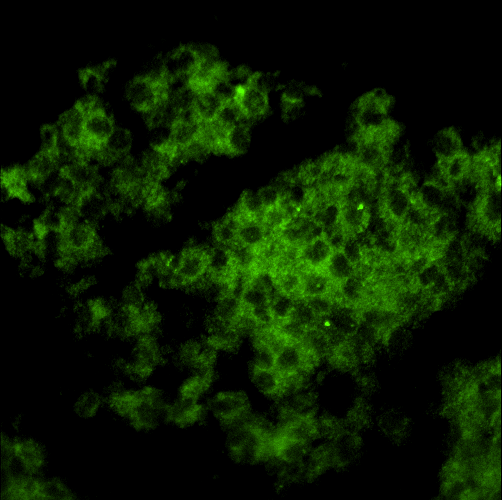

Supplement: Supplementary file 6 — Source Data for Figure 5 [file EMMM-15-e17928-s001.zip › Figure_5/5C_T2D+AAV-PAX6_Insulin.tif]

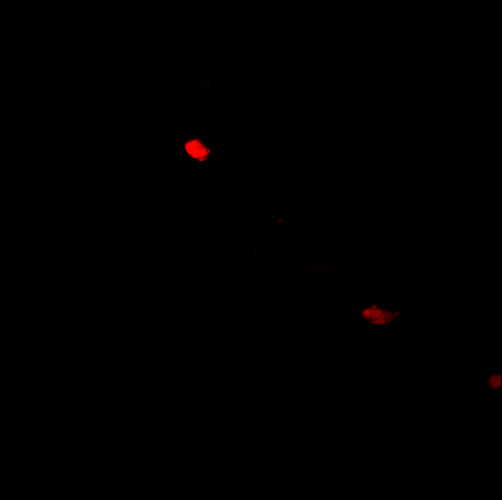

Supplement: Supplementary file 6 — Source Data for Figure 5 [file EMMM-15-e17928-s001.zip › Figure_5/5C_T2D+AAV-PAX6_Ki67.tif]

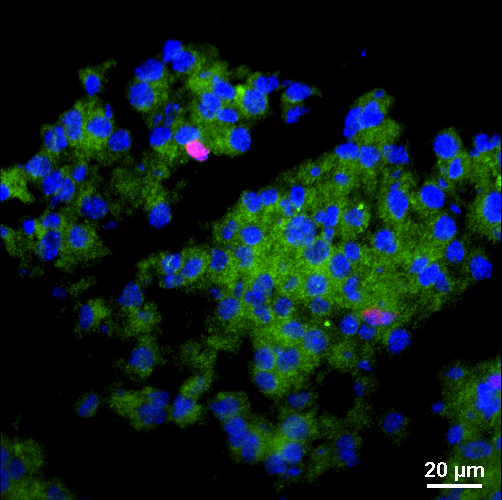

Supplement: Supplementary file 6 — Source Data for Figure 5 [file EMMM-15-e17928-s001.zip › Figure_5/5C_T2D+AAV-PAX6_Merged.tif]

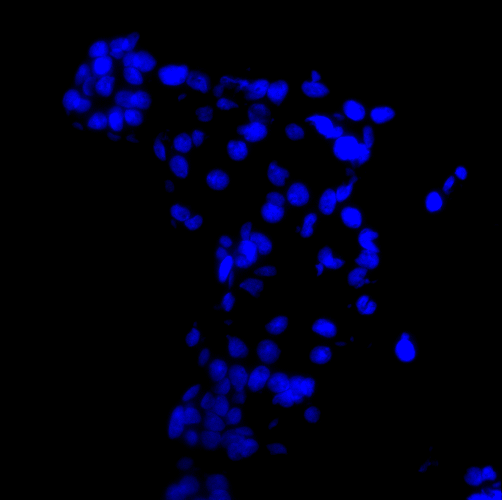

Supplement: Supplementary file 6 — Source Data for Figure 5 [file EMMM-15-e17928-s001.zip › Figure_5/5D_Normal+AAV-Ctrl_DAPI.tif]

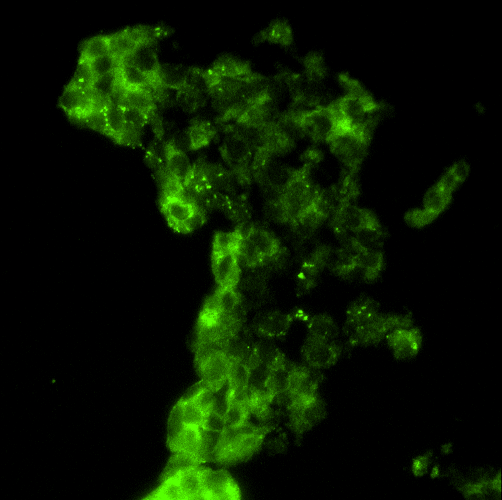

Supplement: Supplementary file 6 — Source Data for Figure 5 [file EMMM-15-e17928-s001.zip › Figure_5/5D_Normal+AAV-Ctrl_Insulin.tif]

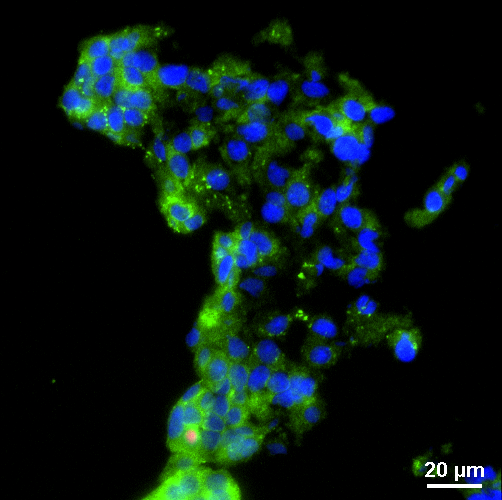

Supplement: Supplementary file 6 — Source Data for Figure 5 [file EMMM-15-e17928-s001.zip › Figure_5/5D_Normal+AAV-Ctrl_Merged.tif]

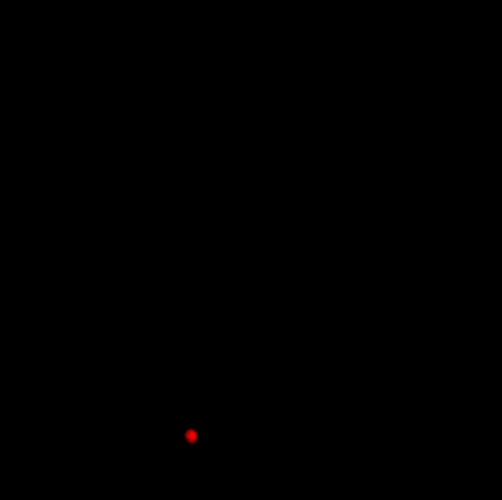

Supplement: Supplementary file 6 — Source Data for Figure 5 [file EMMM-15-e17928-s001.zip › Figure_5/5D_Normal+AAV-Ctrl_Tunel.tif]

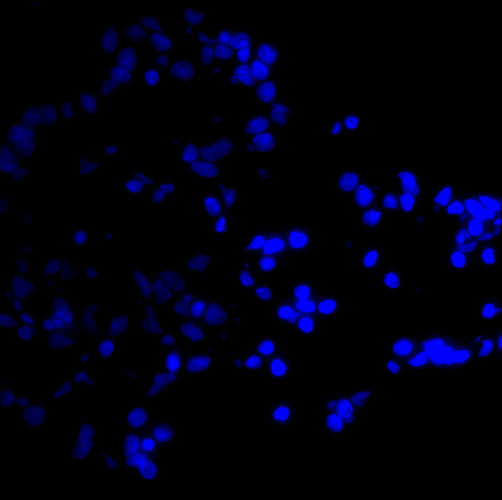

Supplement: Supplementary file 6 — Source Data for Figure 5 [file EMMM-15-e17928-s001.zip › Figure_5/5D_T2D+AAV-Ctrl_DAPI.tif]

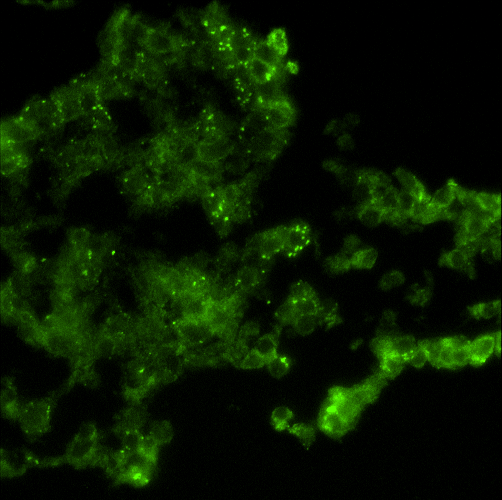

Supplement: Supplementary file 6 — Source Data for Figure 5 [file EMMM-15-e17928-s001.zip › Figure_5/5D_T2D+AAV-Ctrl_Insulin.tif]

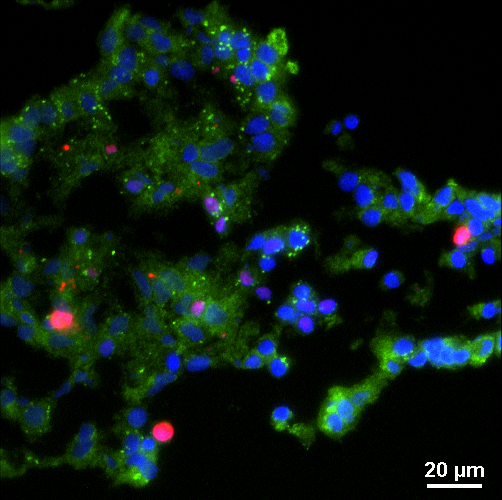

Supplement: Supplementary file 6 — Source Data for Figure 5 [file EMMM-15-e17928-s001.zip › Figure_5/5D_T2D+AAV-Ctrl_Merged.tif]

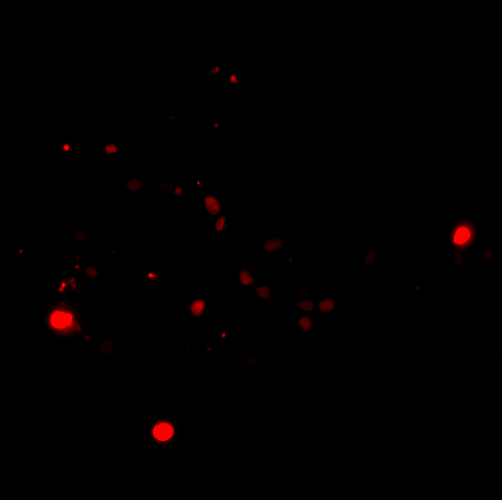

Supplement: Supplementary file 6 — Source Data for Figure 5 [file EMMM-15-e17928-s001.zip › Figure_5/5D_T2D+AAV-Ctrl_Tunel.tif]

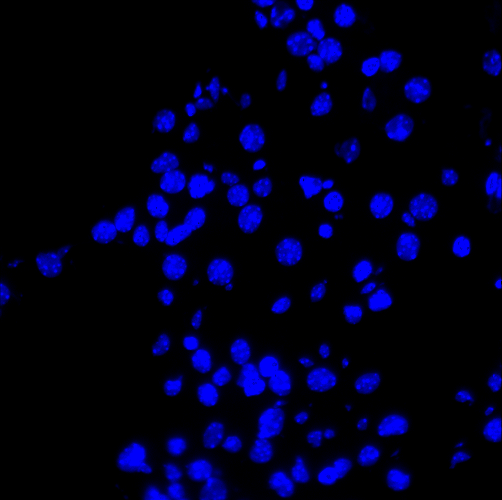

Supplement: Supplementary file 6 — Source Data for Figure 5 [file EMMM-15-e17928-s001.zip › Figure_5/5D_T2D+AAV-PAX6_DAPI.tif]

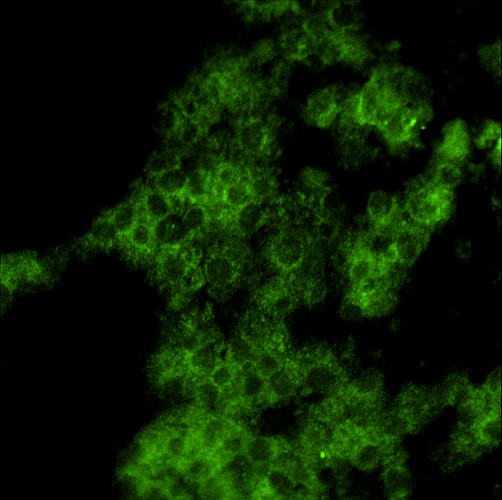

Supplement: Supplementary file 6 — Source Data for Figure 5 [file EMMM-15-e17928-s001.zip › Figure_5/5D_T2D+AAV-PAX6_Insulin.tif]

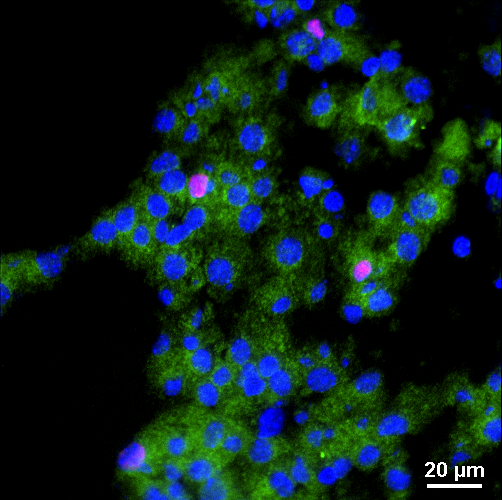

Supplement: Supplementary file 6 — Source Data for Figure 5 [file EMMM-15-e17928-s001.zip › Figure_5/5D_T2D+AAV-PAX6_Merged.tif]

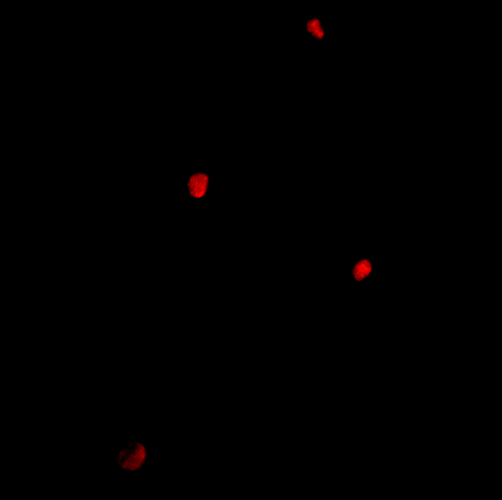

Supplement: Supplementary file 6 — Source Data for Figure 5 [file EMMM-15-e17928-s001.zip › Figure_5/5D_T2D+AAV-PAX6_Tunel.tif]

E

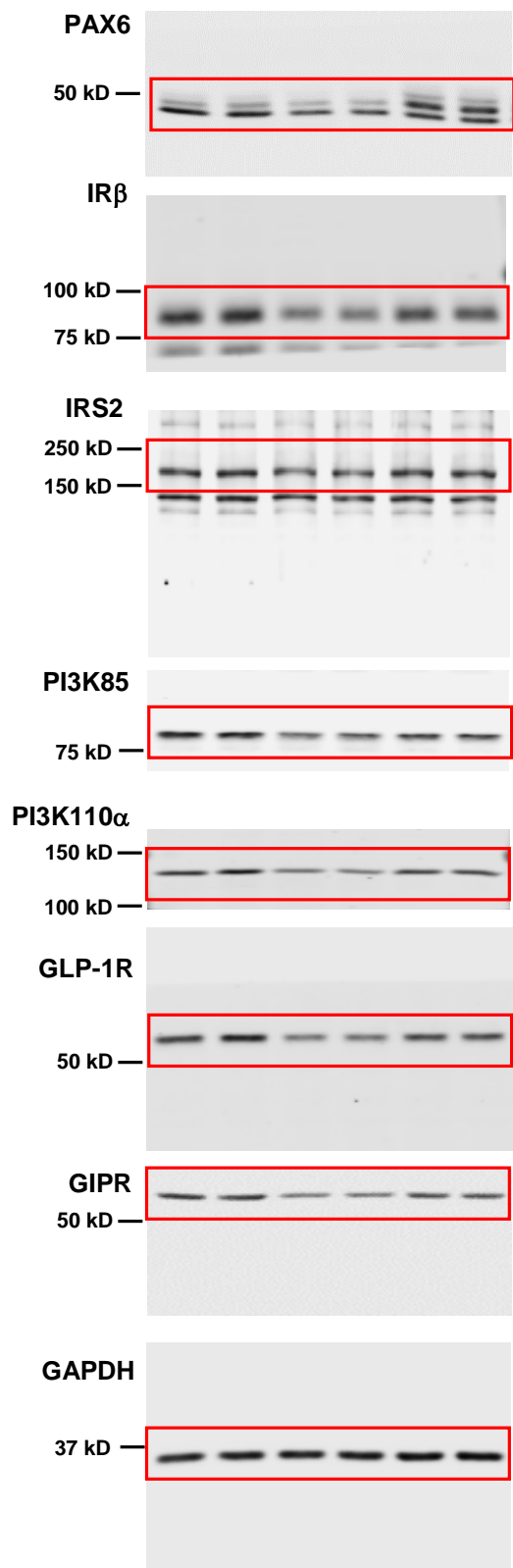

F

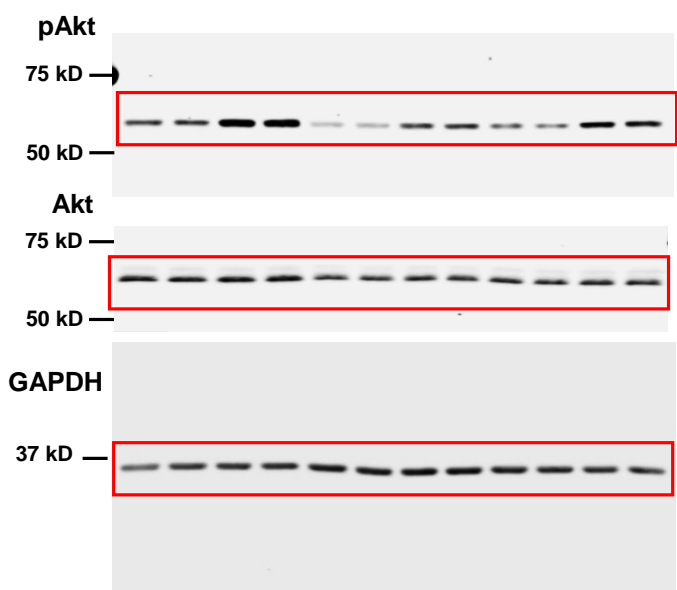

G

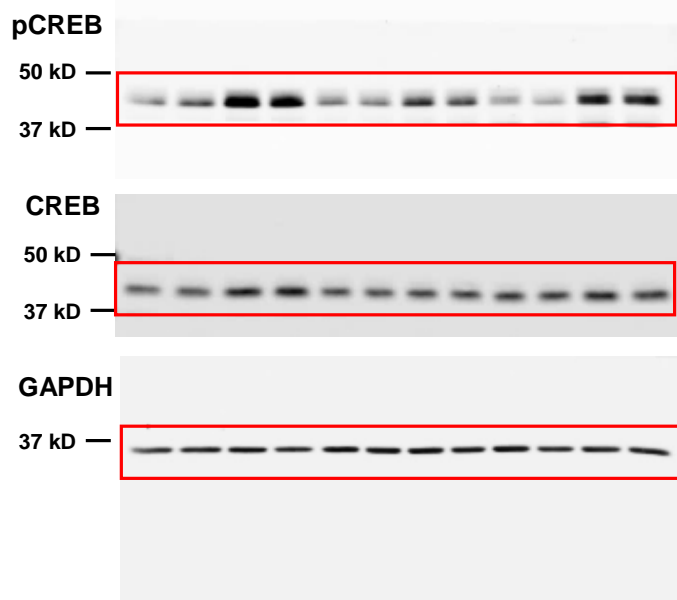

H

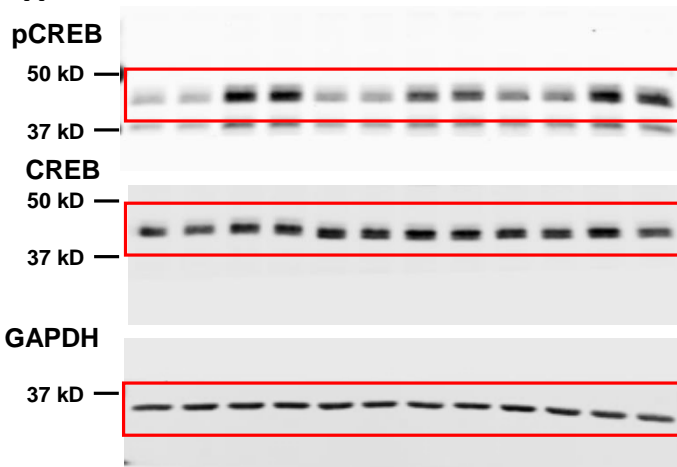

Supplement: Supplementary file 6 — Source Data for Figure 5 [file EMMM-15-e17928-s001.zip › Figure_5/Figure_5_western.pdf]

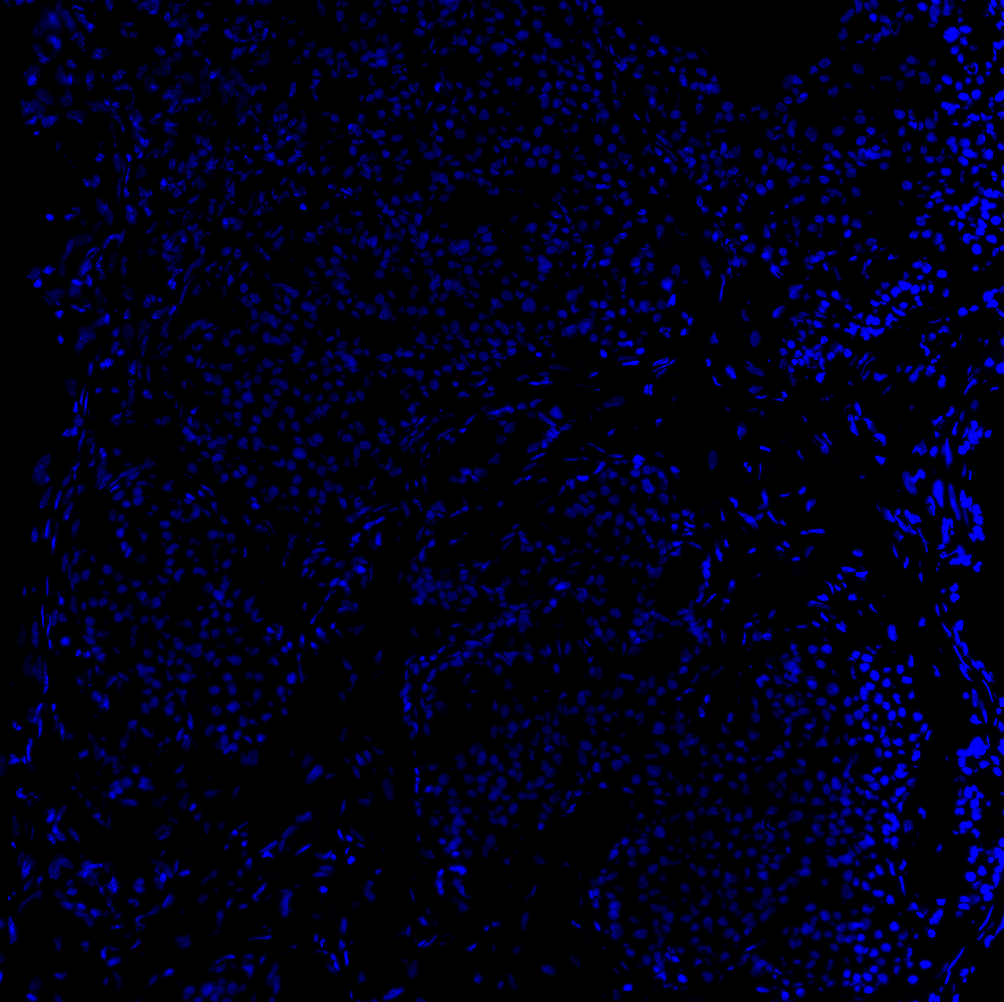

Supplement: Supplementary file 7 — Source Data for Figure 6 [file EMMM-15-e17928-s007.zip › Figure_6/6E_STZ_Nor-Ctrl_DAPI.tif]

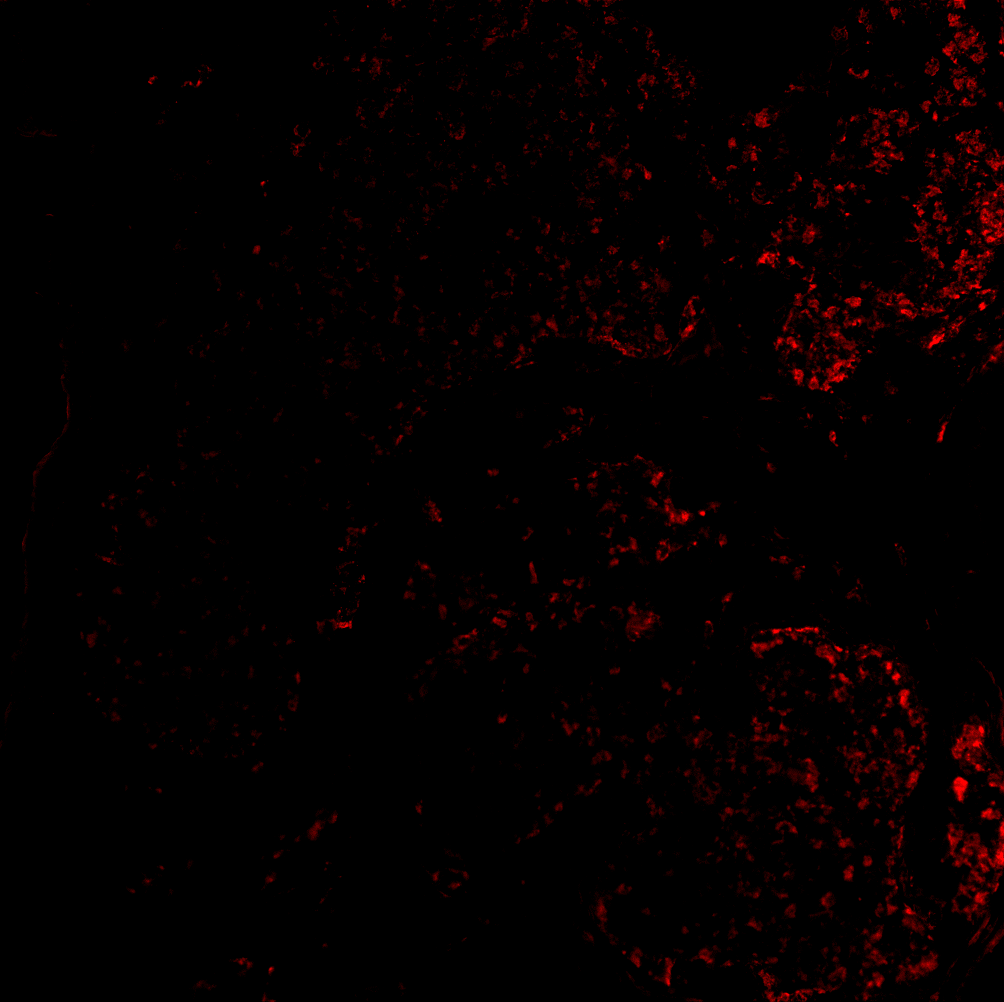

Supplement: Supplementary file 7 — Source Data for Figure 6 [file EMMM-15-e17928-s007.zip › Figure_6/6E_STZ_Nor-Ctrl_Glucagon.tif]

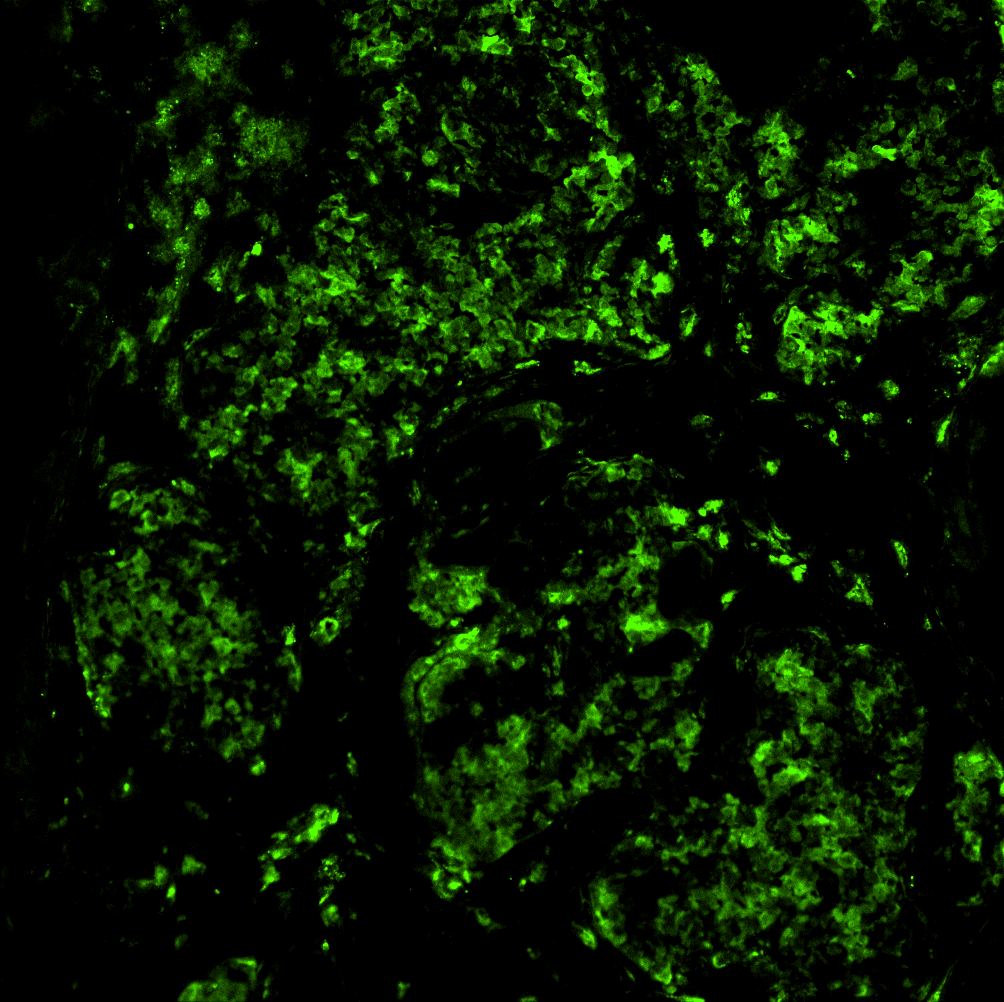

Supplement: Supplementary file 7 — Source Data for Figure 6 [file EMMM-15-e17928-s007.zip › Figure_6/6E_STZ_Nor-Ctrl_Insulin.tif]

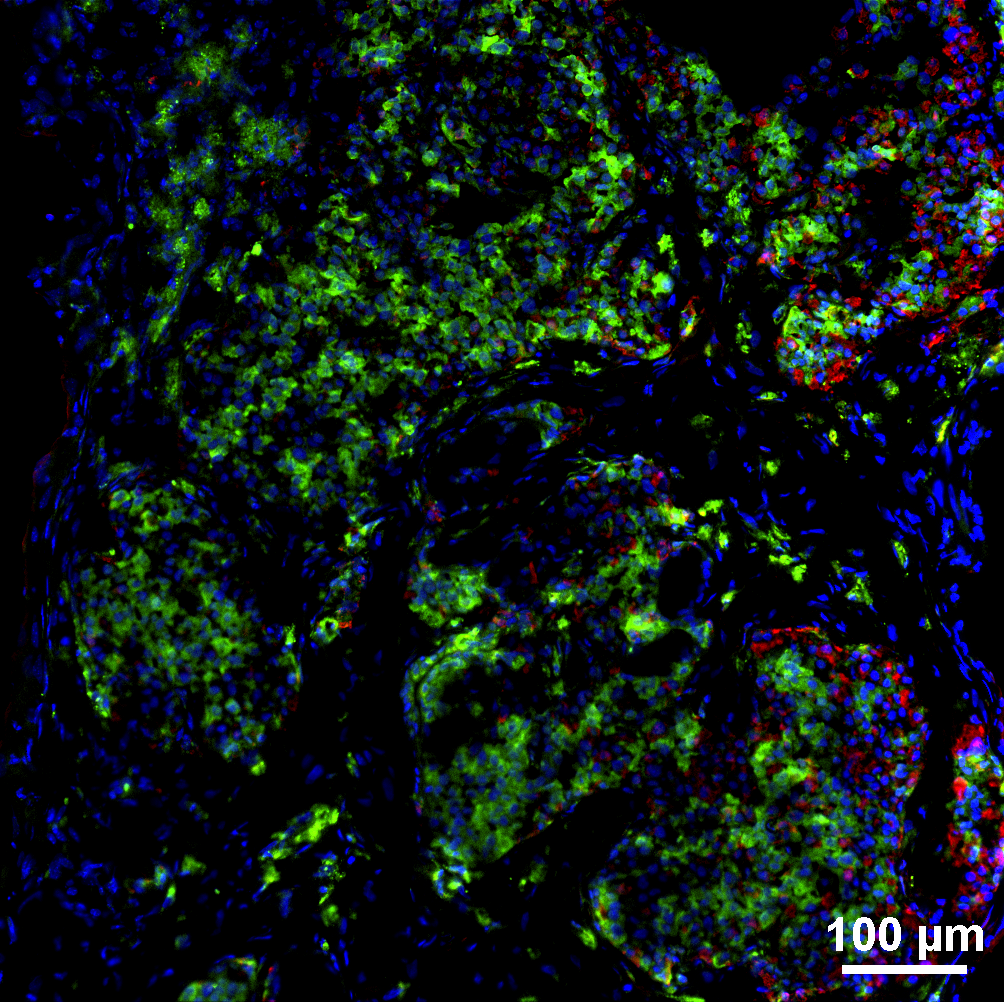

Supplement: Supplementary file 7 — Source Data for Figure 6 [file EMMM-15-e17928-s007.zip › Figure_6/6E_STZ_Nor-Ctrl_Merged.tif]

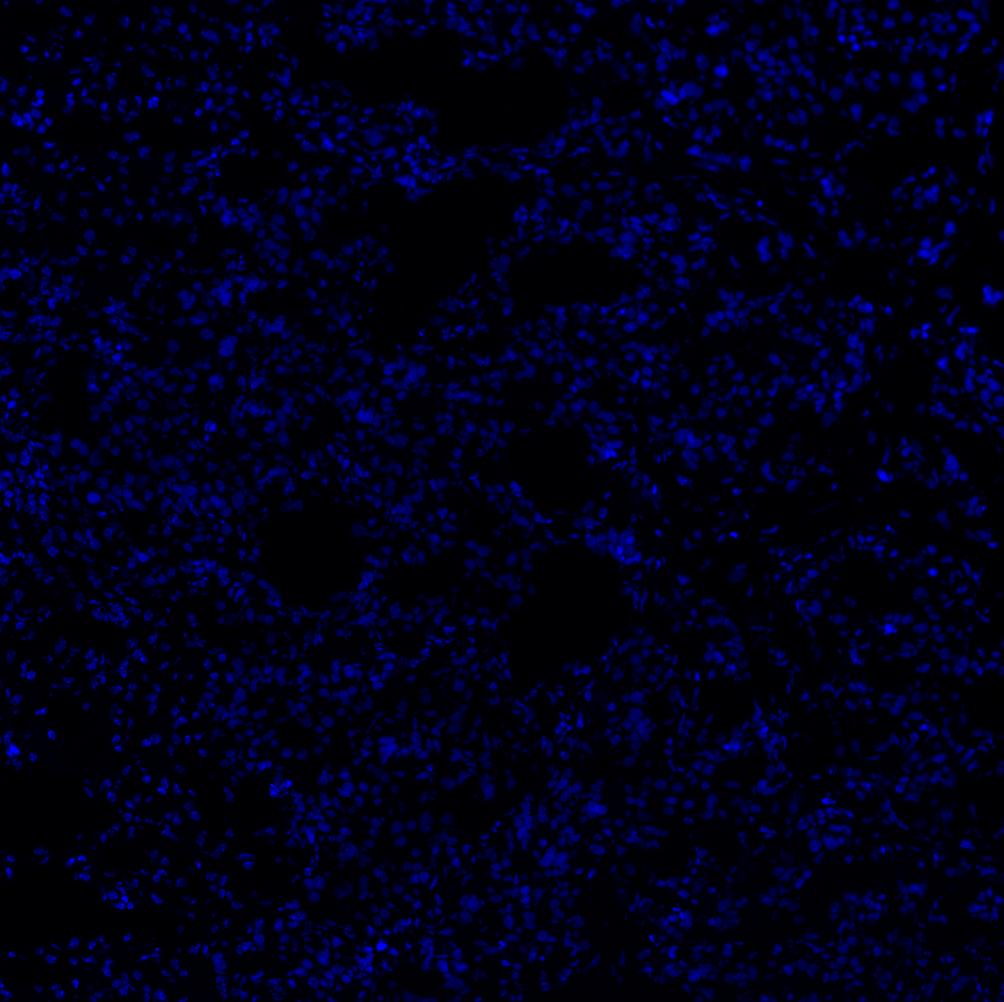

Supplement: Supplementary file 7 — Source Data for Figure 6 [file EMMM-15-e17928-s007.zip › Figure_6/6E_STZ_T2D-Ctrl_DAPI.tif]

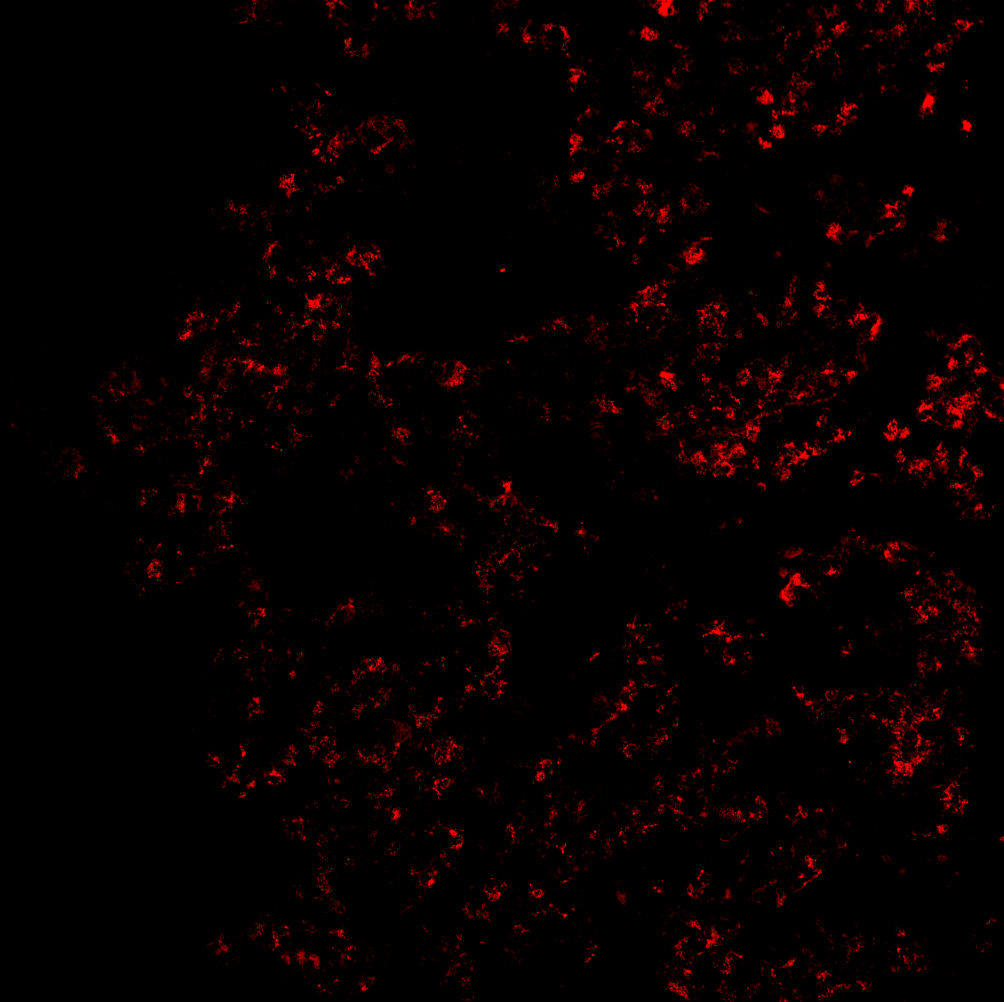

Supplement: Supplementary file 7 — Source Data for Figure 6 [file EMMM-15-e17928-s007.zip › Figure_6/6E_STZ_T2D-Ctrl_Glucagon.tif]

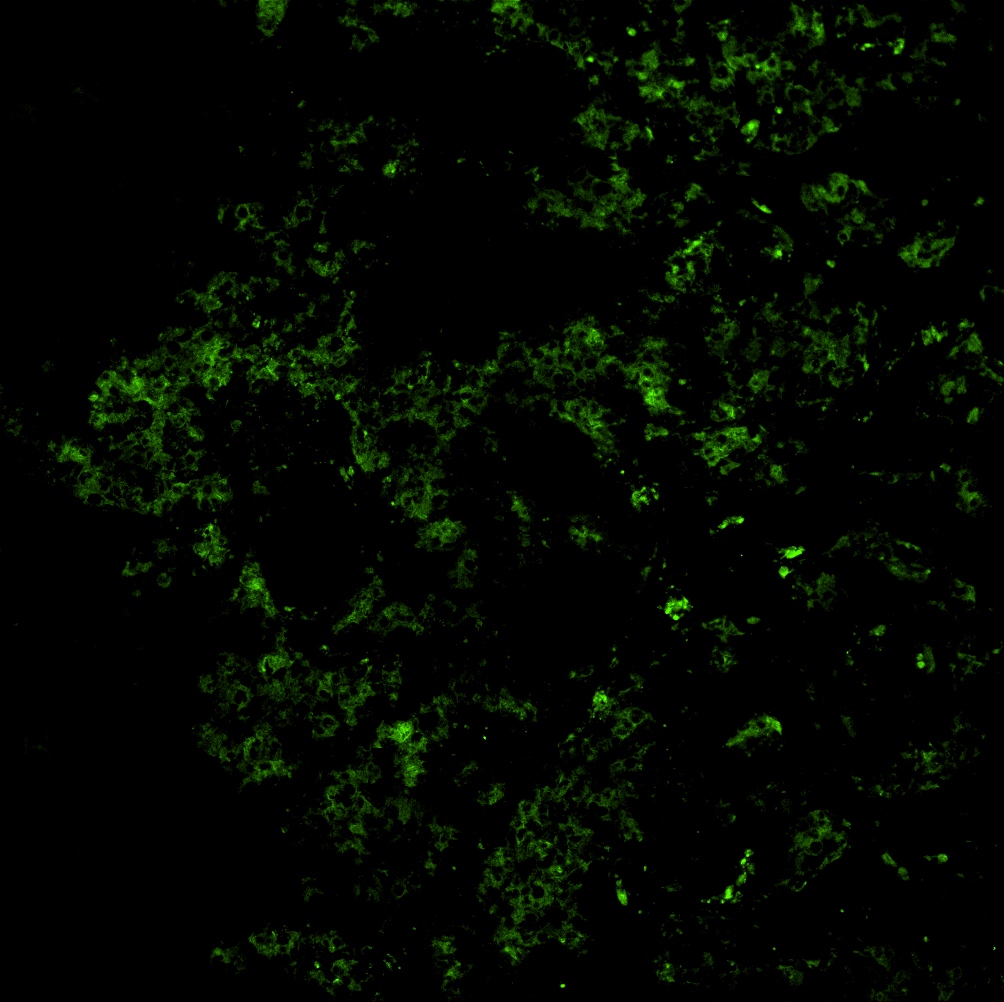

Supplement: Supplementary file 7 — Source Data for Figure 6 [file EMMM-15-e17928-s007.zip › Figure_6/6E_STZ_T2D-Ctrl_Insulin.tif]

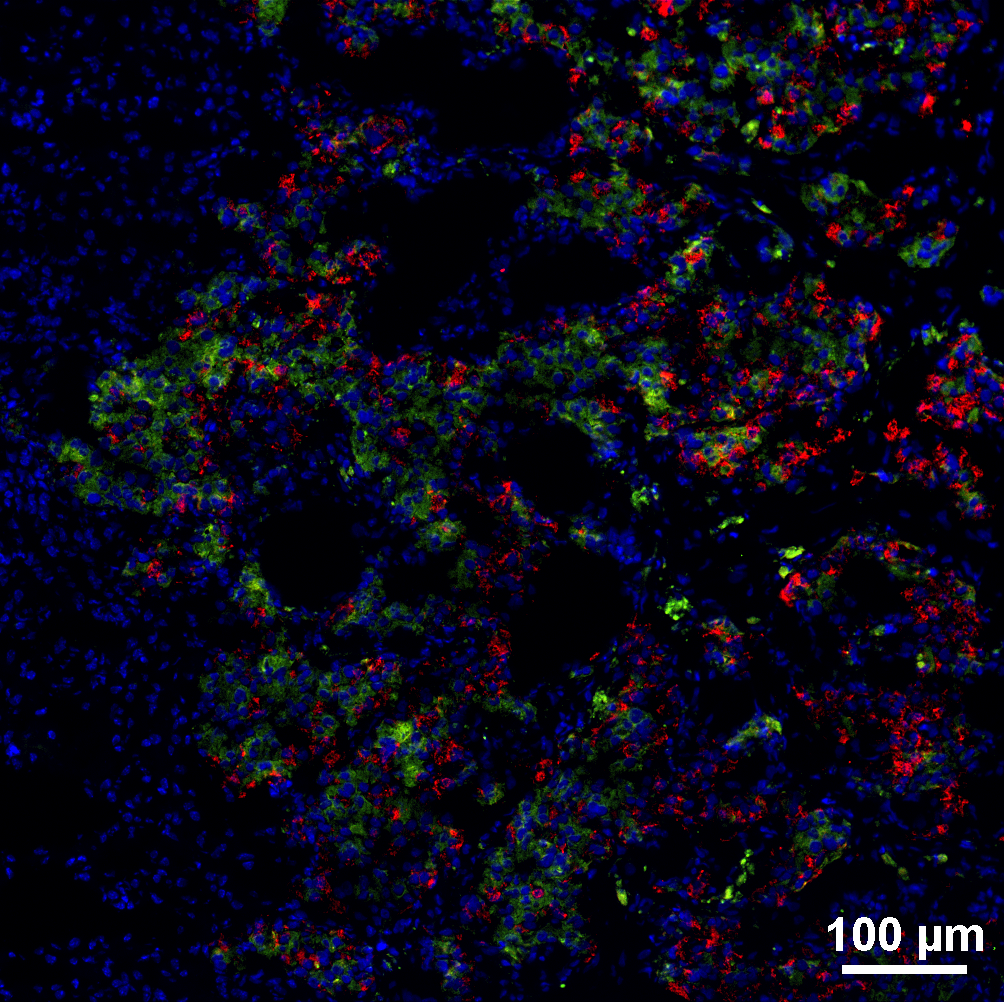

Supplement: Supplementary file 7 — Source Data for Figure 6 [file EMMM-15-e17928-s007.zip › Figure_6/6E_STZ_T2D-Ctrl_Merged.tif]

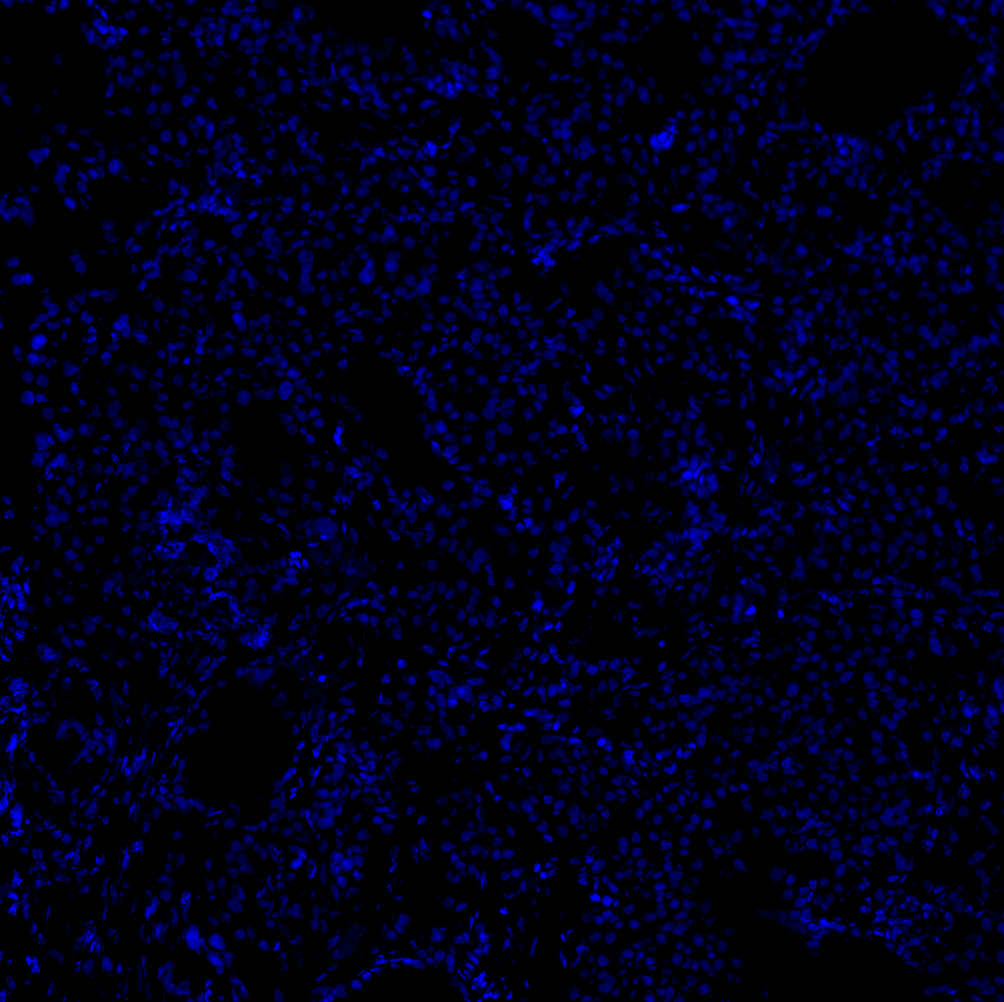

Supplement: Supplementary file 7 — Source Data for Figure 6 [file EMMM-15-e17928-s007.zip › Figure_6/6E_STZ_T2D-PAX6_DAPI.tif]

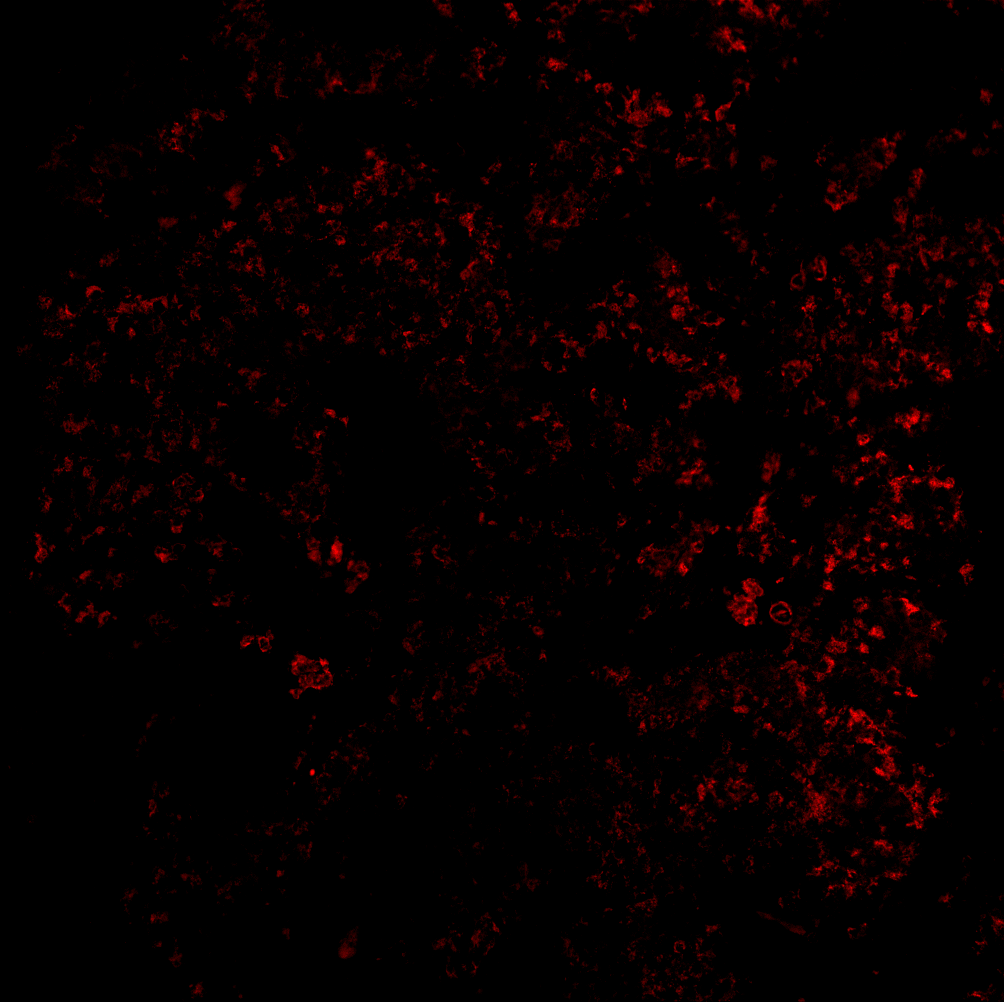

Supplement: Supplementary file 7 — Source Data for Figure 6 [file EMMM-15-e17928-s007.zip › Figure_6/6E_STZ_T2D-PAX6_Glucagon.tif]

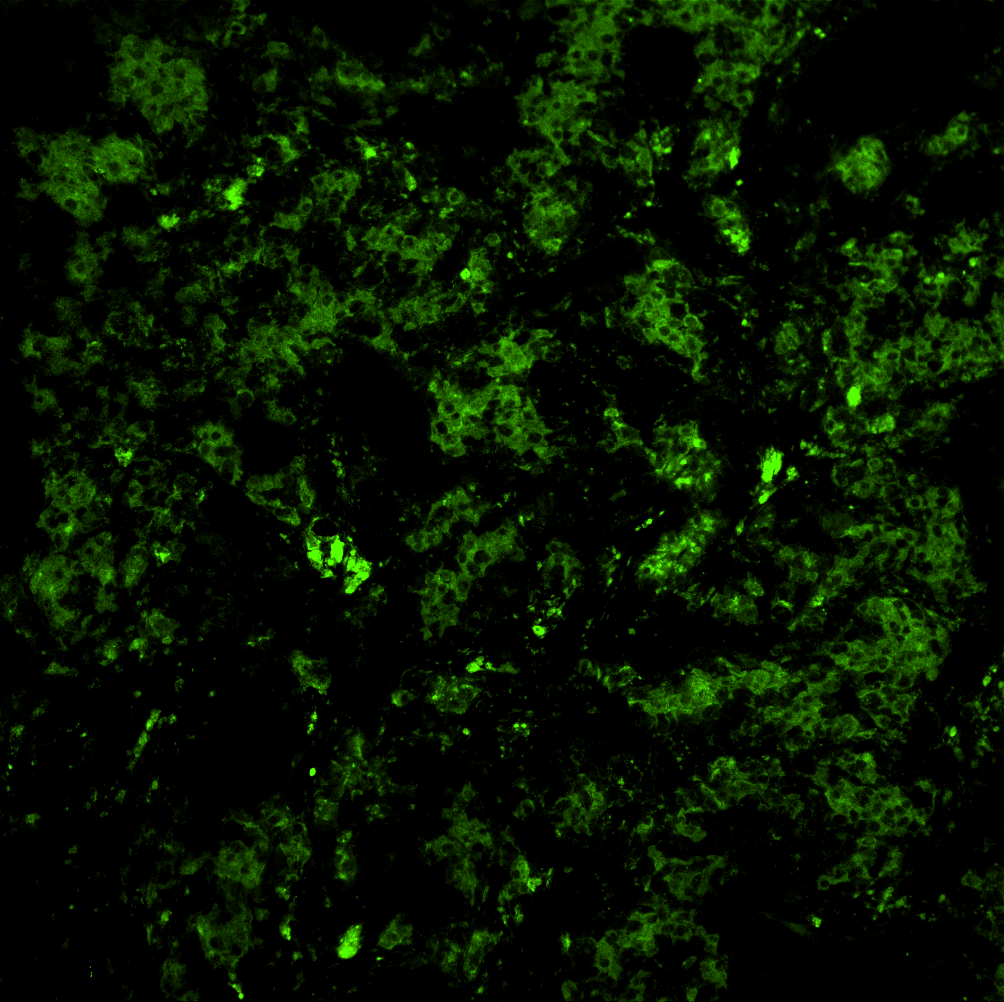

Supplement: Supplementary file 7 — Source Data for Figure 6 [file EMMM-15-e17928-s007.zip › Figure_6/6E_STZ_T2D-PAX6_Insulin.tif]

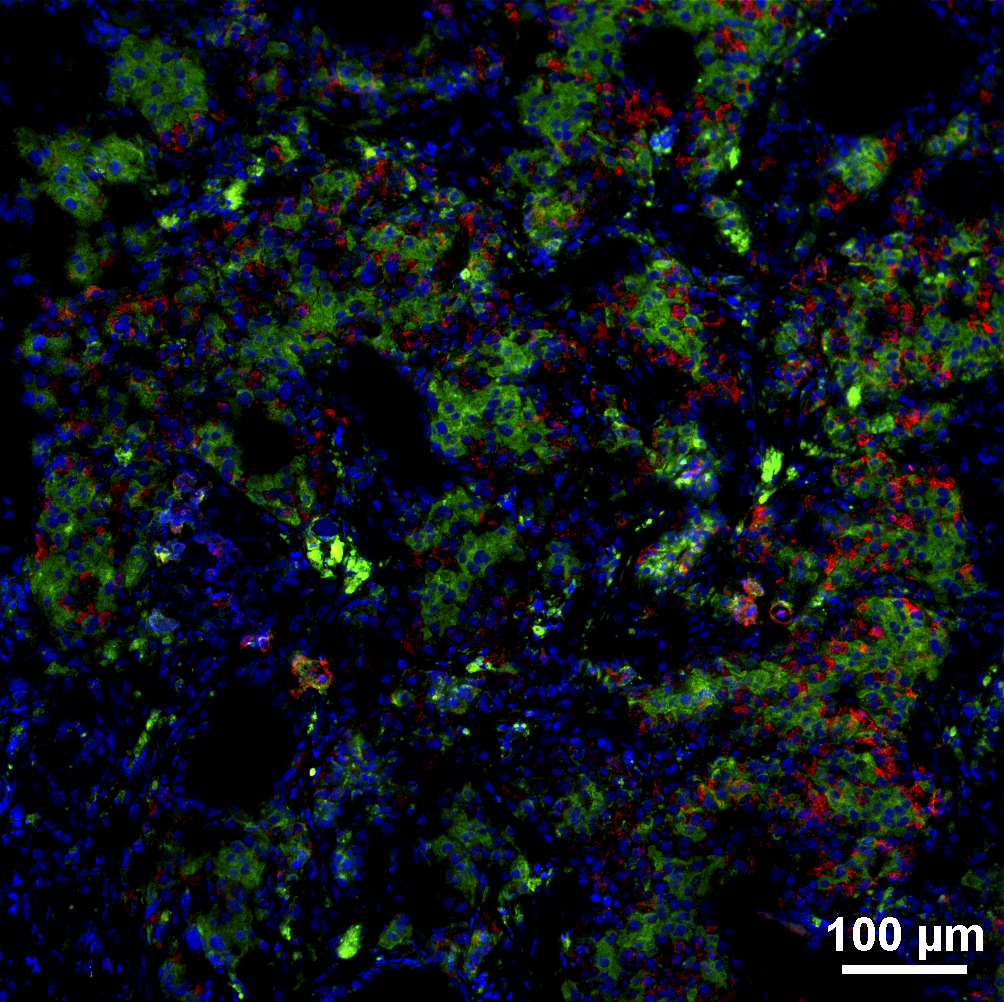

Supplement: Supplementary file 7 — Source Data for Figure 6 [file EMMM-15-e17928-s007.zip › Figure_6/6E_STZ_T2D-PAX6_Merged.tif]
