# Supplementary figures and images for: Paired box 6 gene delivery preserves beta cells and improves islet transplantation efficacy (part 2 of 2)
Source: EMBO Mol Med. 2023 Nov 7;15(12):e17928. doi: 10.15252/emmm.202317928 (PMC10701606; doi:10.15252/emmm.202317928)

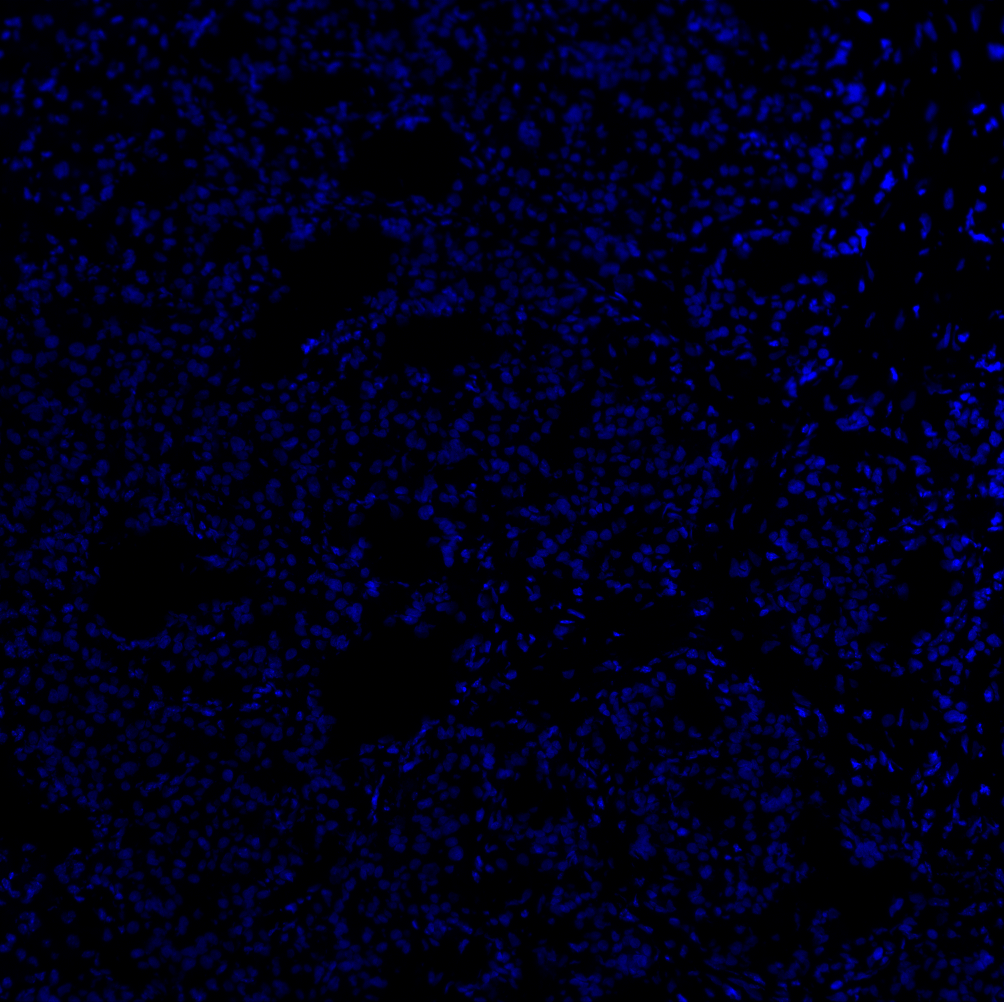

Supplement: Supplementary file 7 — Source Data for Figure 6 [file EMMM-15-e17928-s007.zip › Figure_6/6F_STZ_Nor-Ctrl_DAPI.tif]

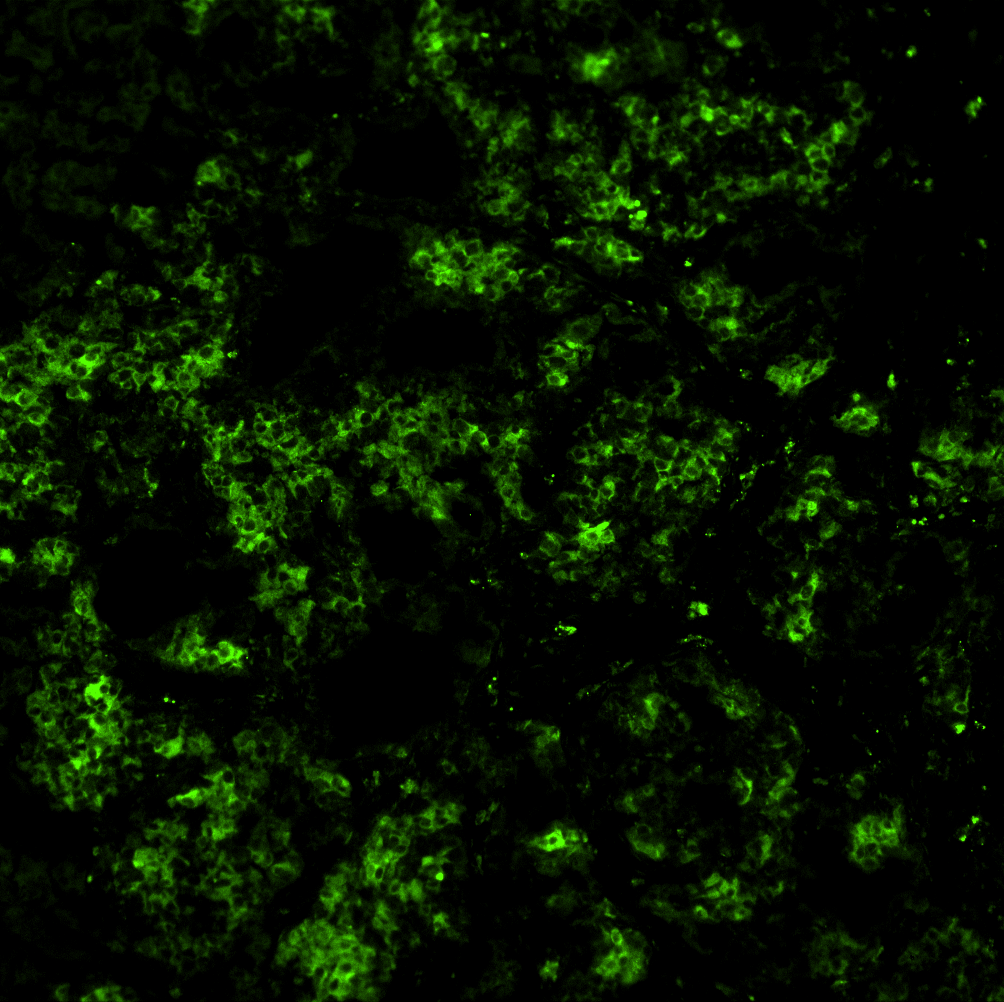

Supplement: Supplementary file 7 — Source Data for Figure 6 [file EMMM-15-e17928-s007.zip › Figure_6/6F_STZ_Nor-Ctrl_Insulin.tif]

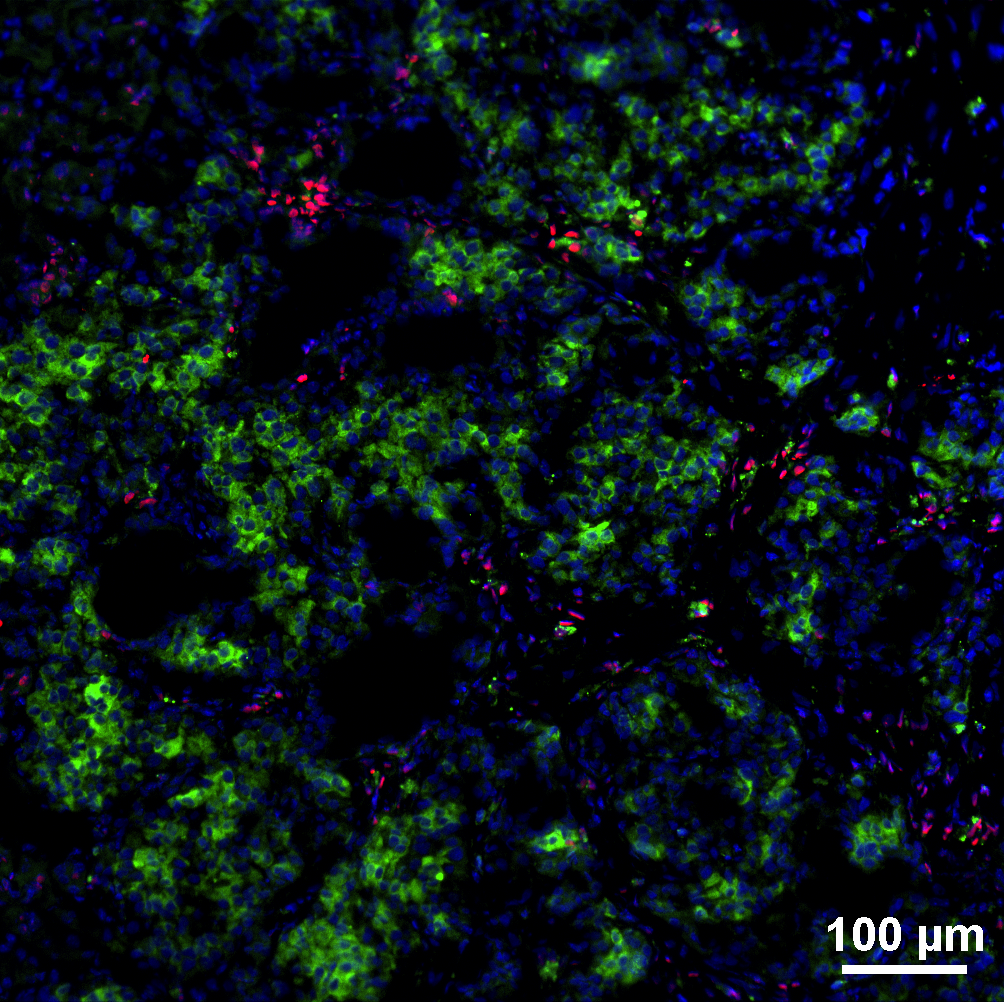

Supplement: Supplementary file 7 — Source Data for Figure 6 [file EMMM-15-e17928-s007.zip › Figure_6/6F_STZ_Nor-Ctrl_Merged.tif]

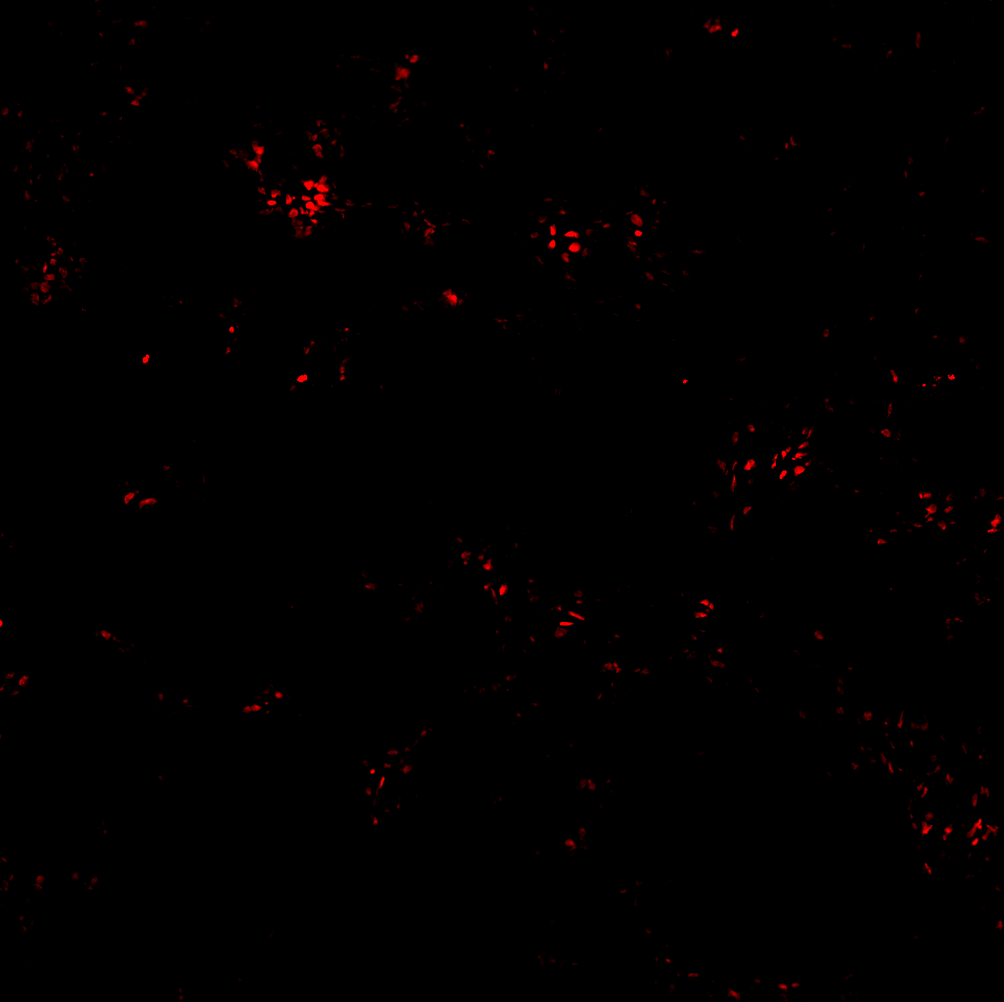

Supplement: Supplementary file 7 — Source Data for Figure 6 [file EMMM-15-e17928-s007.zip › Figure_6/6F_STZ_Nor-Ctrl_Tunel.tif]

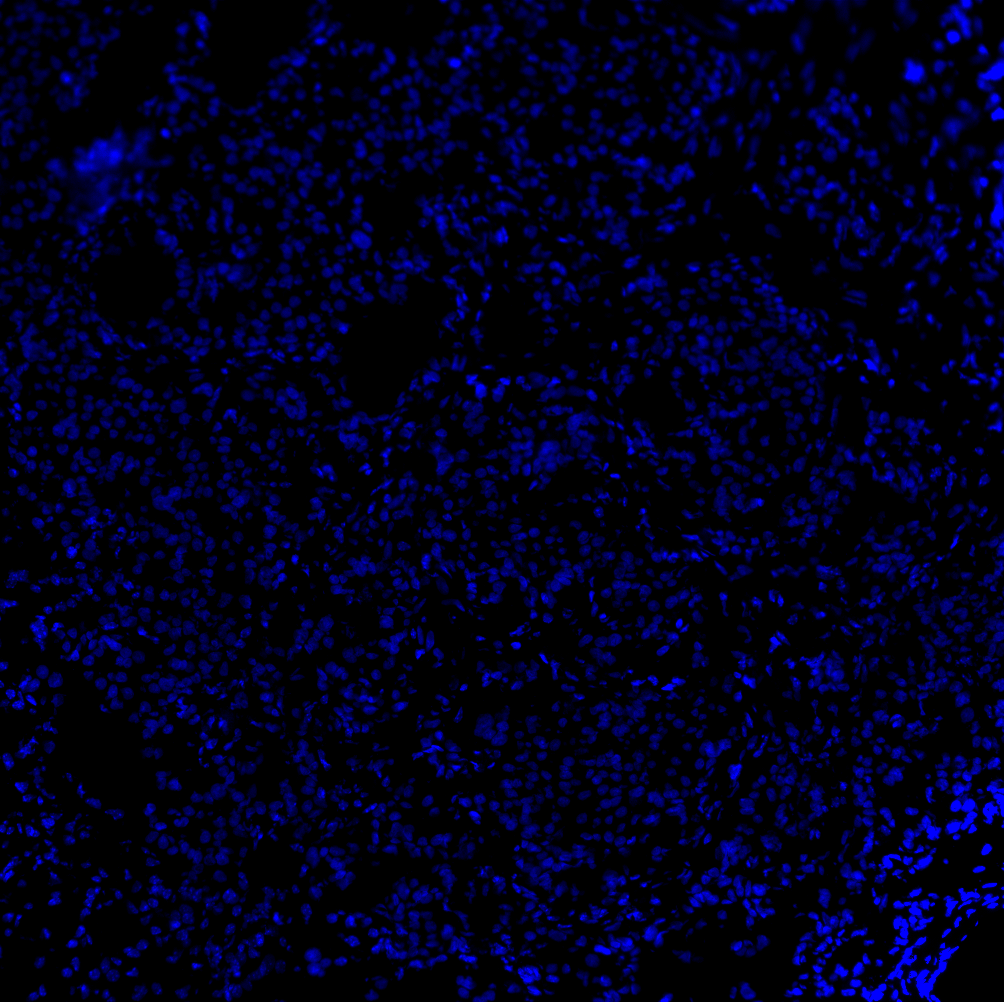

Supplement: Supplementary file 7 — Source Data for Figure 6 [file EMMM-15-e17928-s007.zip › Figure_6/6F_STZ_T2D-Ctrl_DAPI.tif]

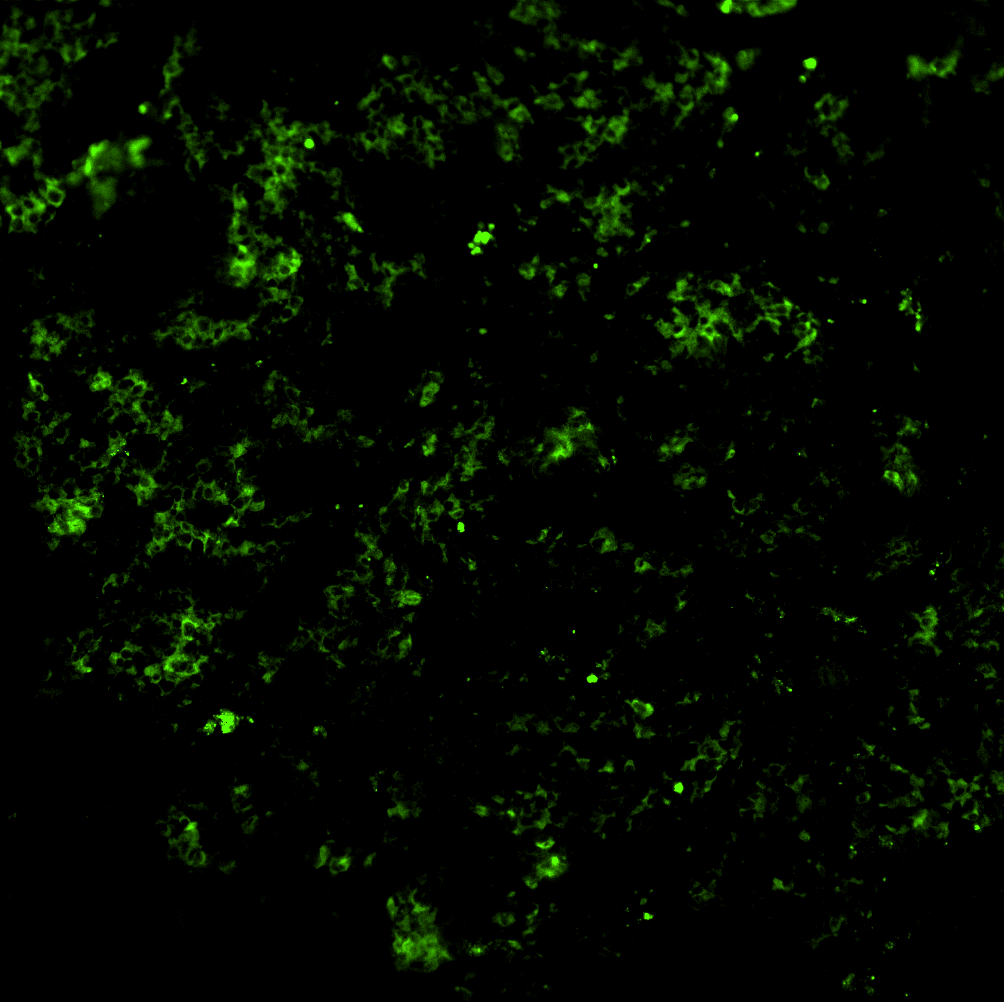

Supplement: Supplementary file 7 — Source Data for Figure 6 [file EMMM-15-e17928-s007.zip › Figure_6/6F_STZ_T2D-Ctrl_Insulin.tif]

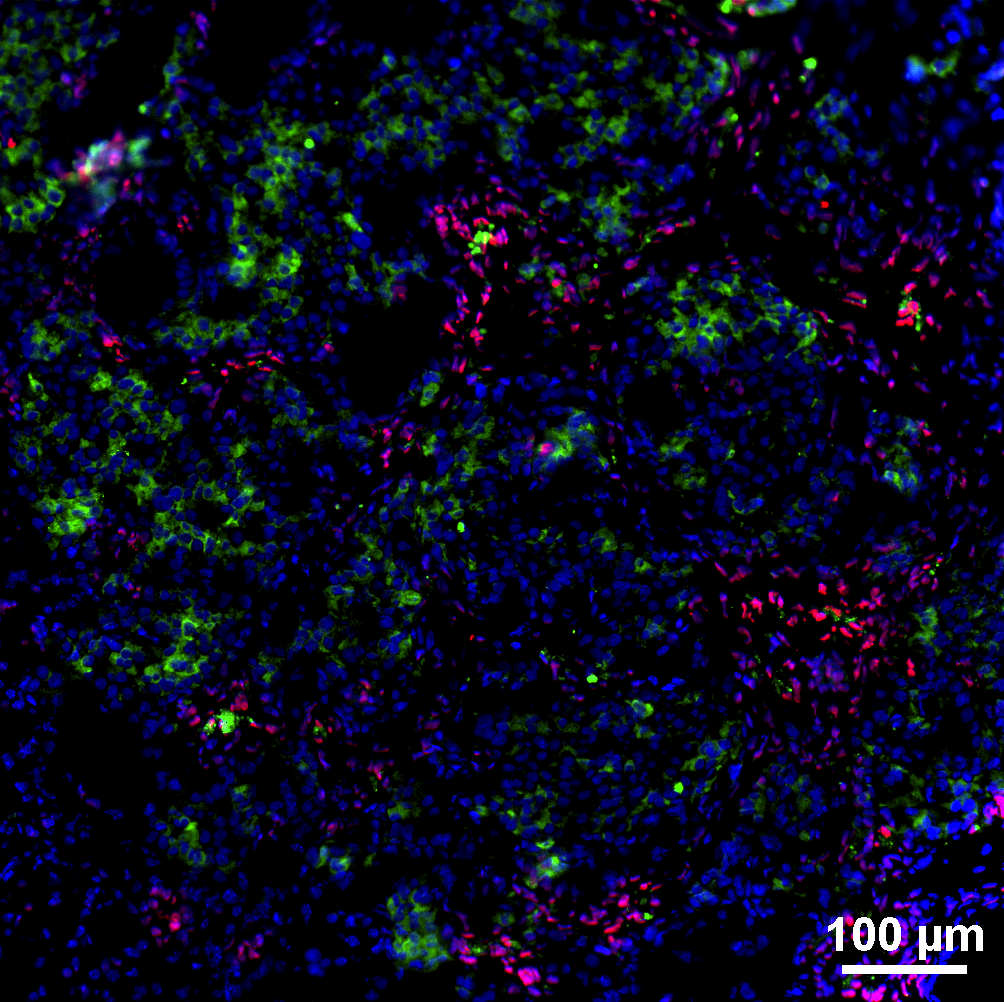

Supplement: Supplementary file 7 — Source Data for Figure 6 [file EMMM-15-e17928-s007.zip › Figure_6/6F_STZ_T2D-Ctrl_Merged.tif]

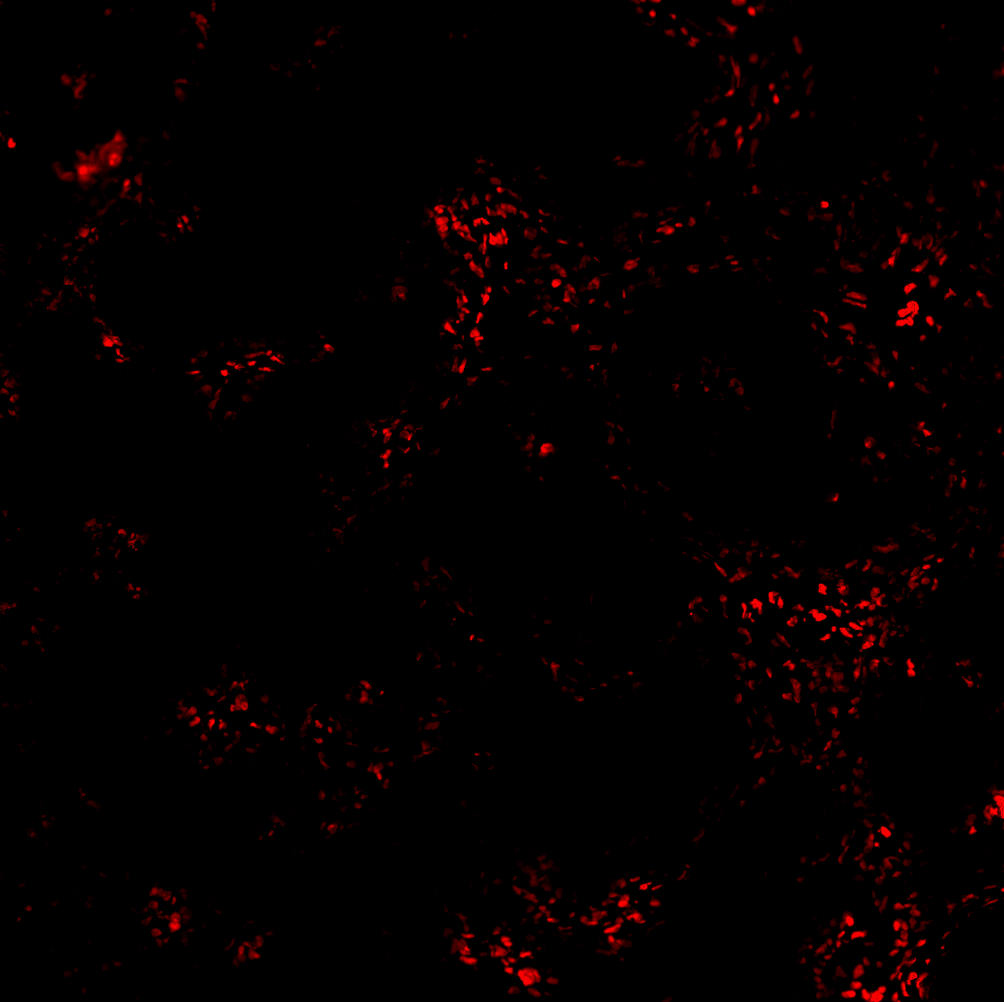

Supplement: Supplementary file 7 — Source Data for Figure 6 [file EMMM-15-e17928-s007.zip › Figure_6/6F_STZ_T2D-Ctrl_Tunel.tif]

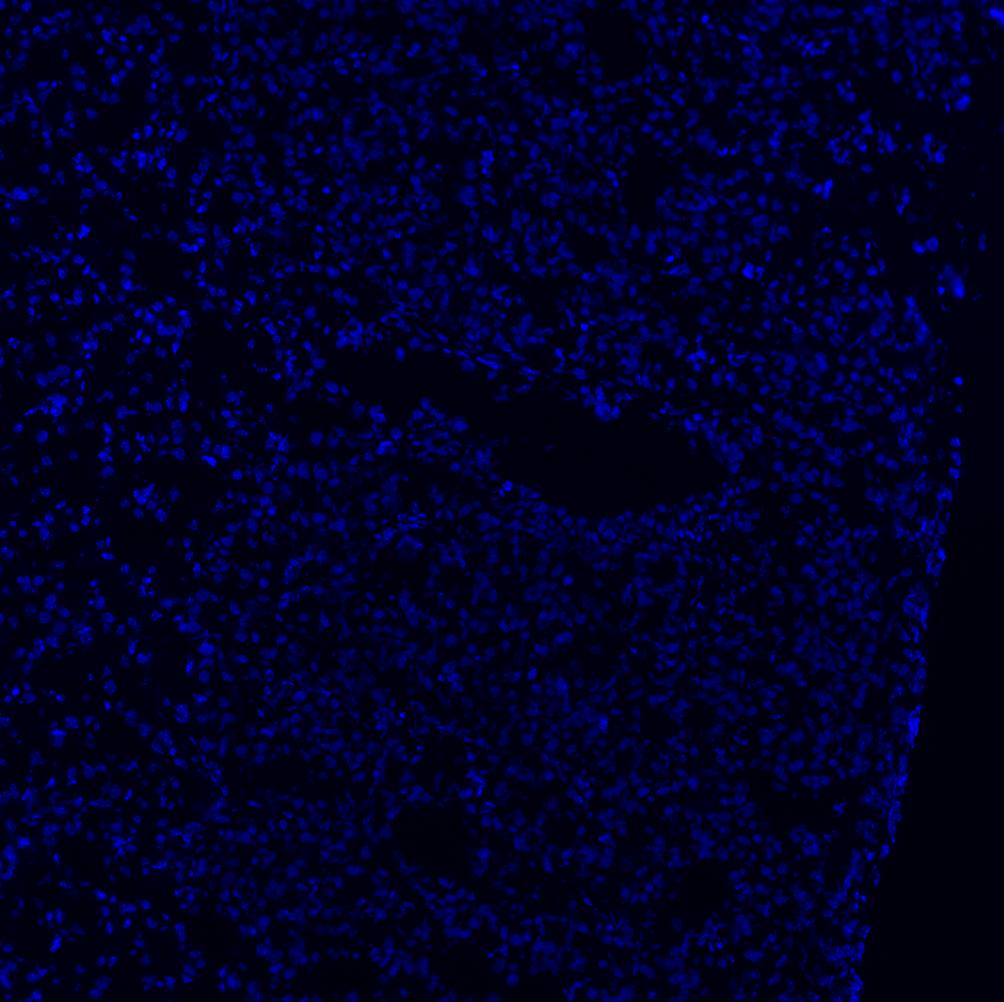

Supplement: Supplementary file 7 — Source Data for Figure 6 [file EMMM-15-e17928-s007.zip › Figure_6/6F_STZ_T2D-PAX6_DAPI.tif]

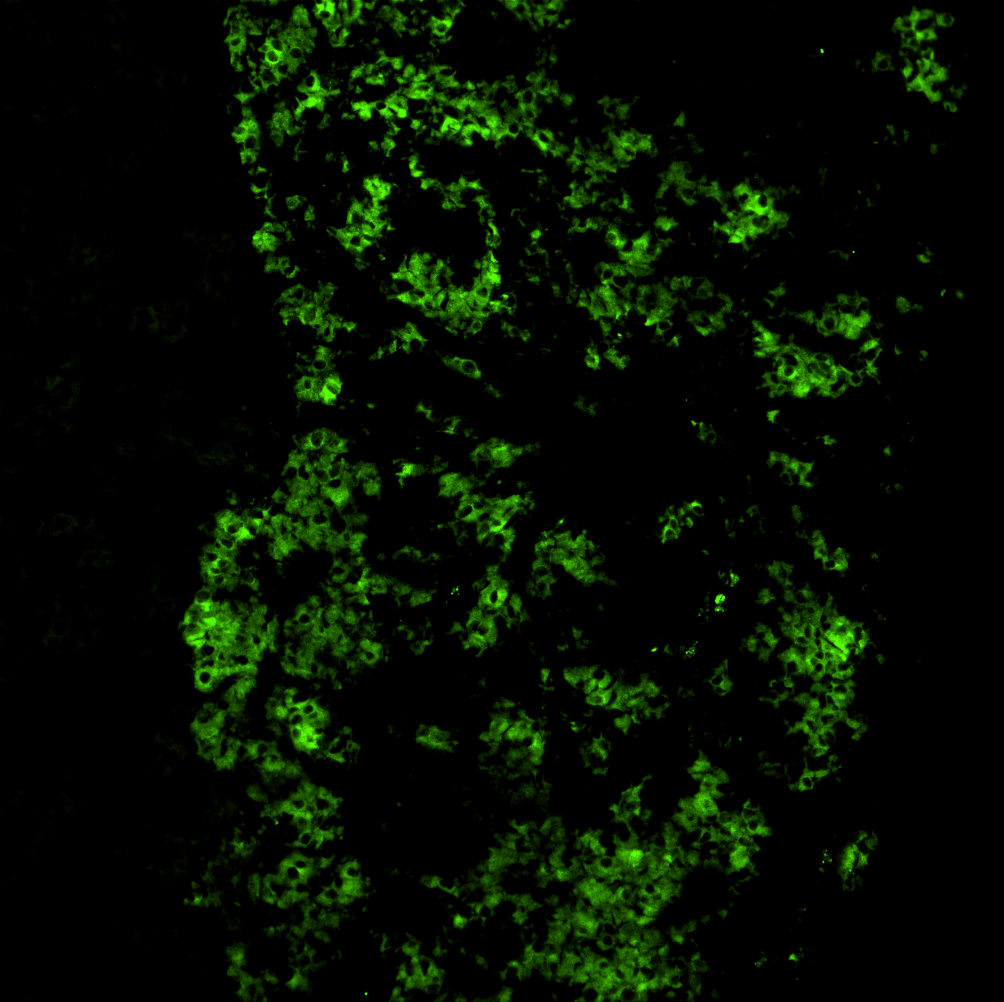

Supplement: Supplementary file 7 — Source Data for Figure 6 [file EMMM-15-e17928-s007.zip › Figure_6/6F_STZ_T2D-PAX6_Insulin.tif]

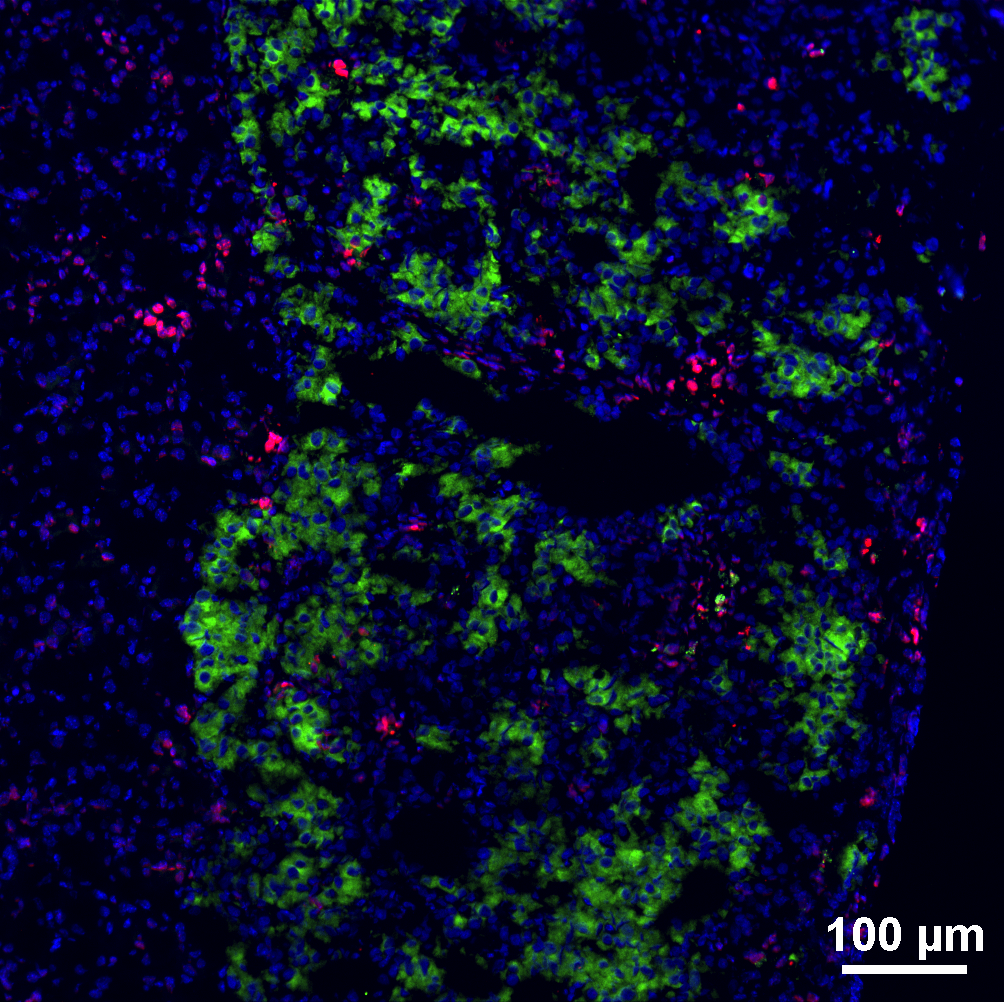

Supplement: Supplementary file 7 — Source Data for Figure 6 [file EMMM-15-e17928-s007.zip › Figure_6/6F_STZ_T2D-PAX6_Merged.tif]

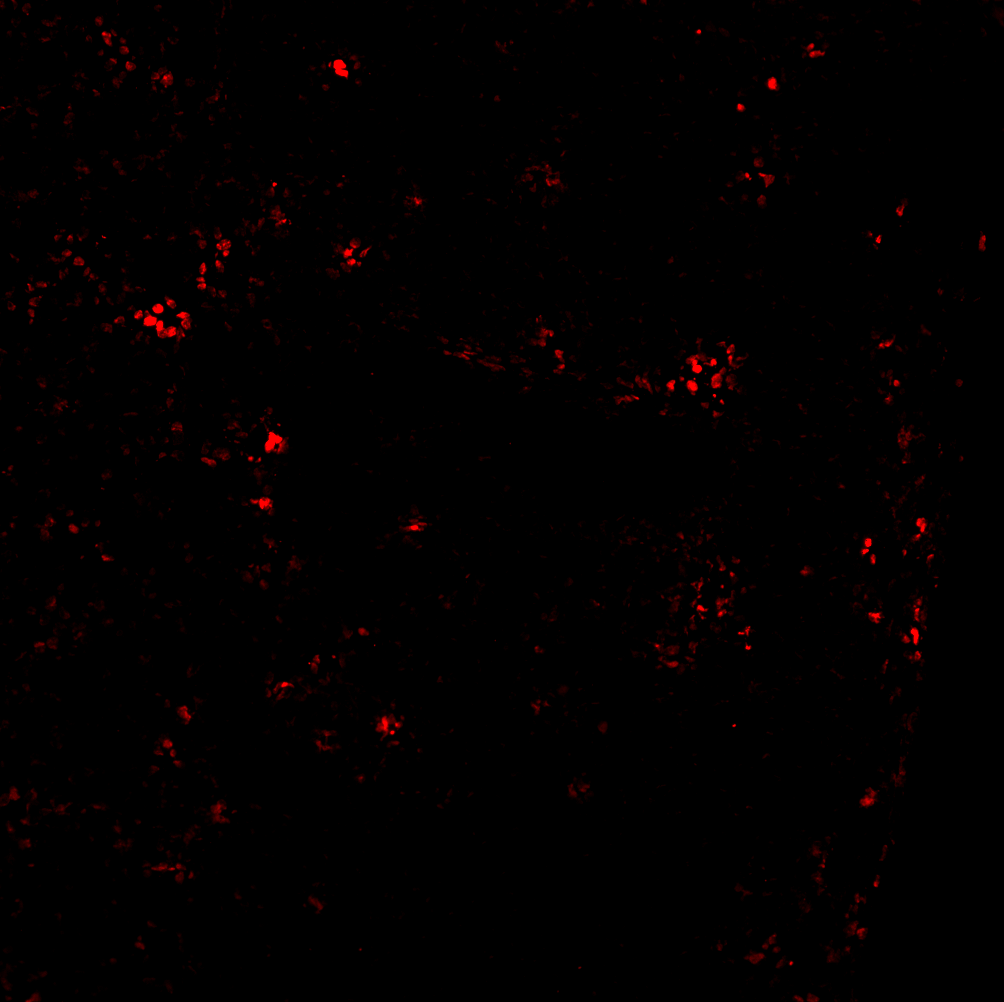

Supplement: Supplementary file 7 — Source Data for Figure 6 [file EMMM-15-e17928-s007.zip › Figure_6/6F_STZ_T2D-PAX6_Tunel.tif]

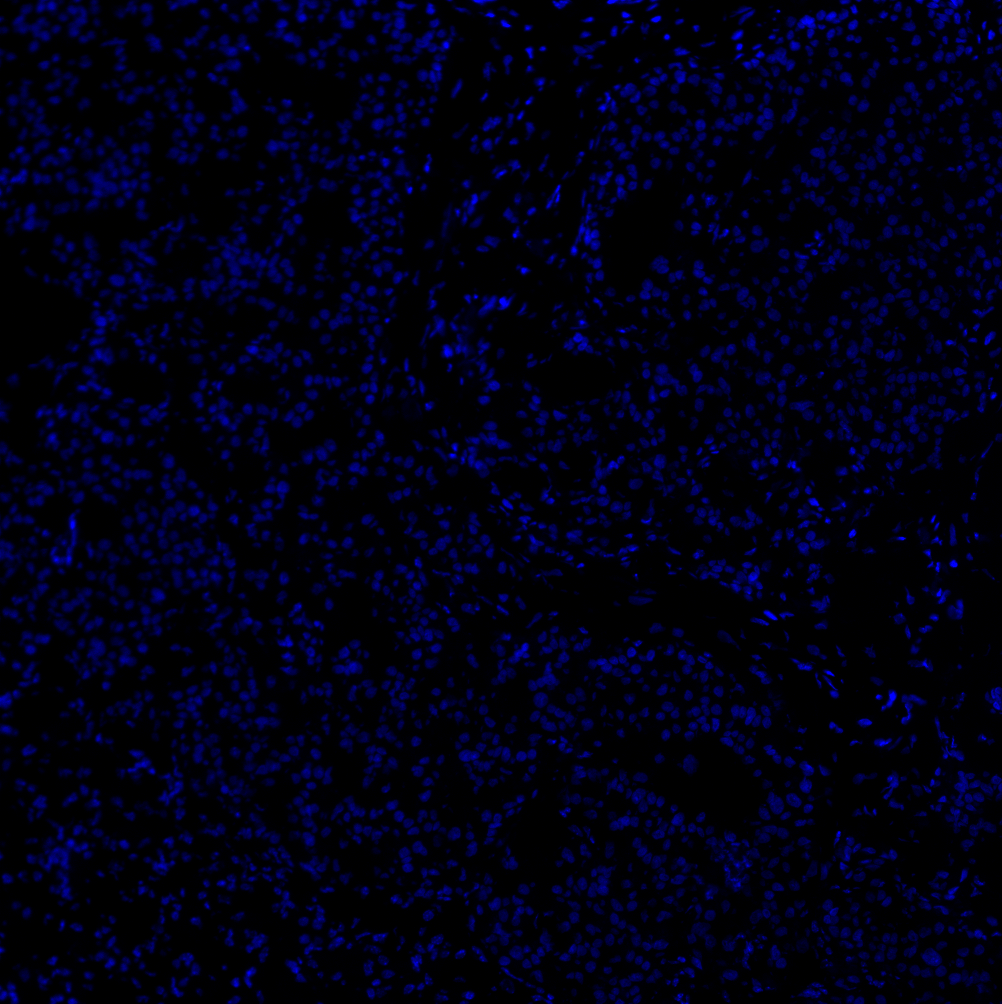

Supplement: Supplementary file 8 — Source Data for Figure 7 [file EMMM-15-e17928-s004.zip › Figure_7/7L_STZ_Ctrl-H_DAPI.tif]

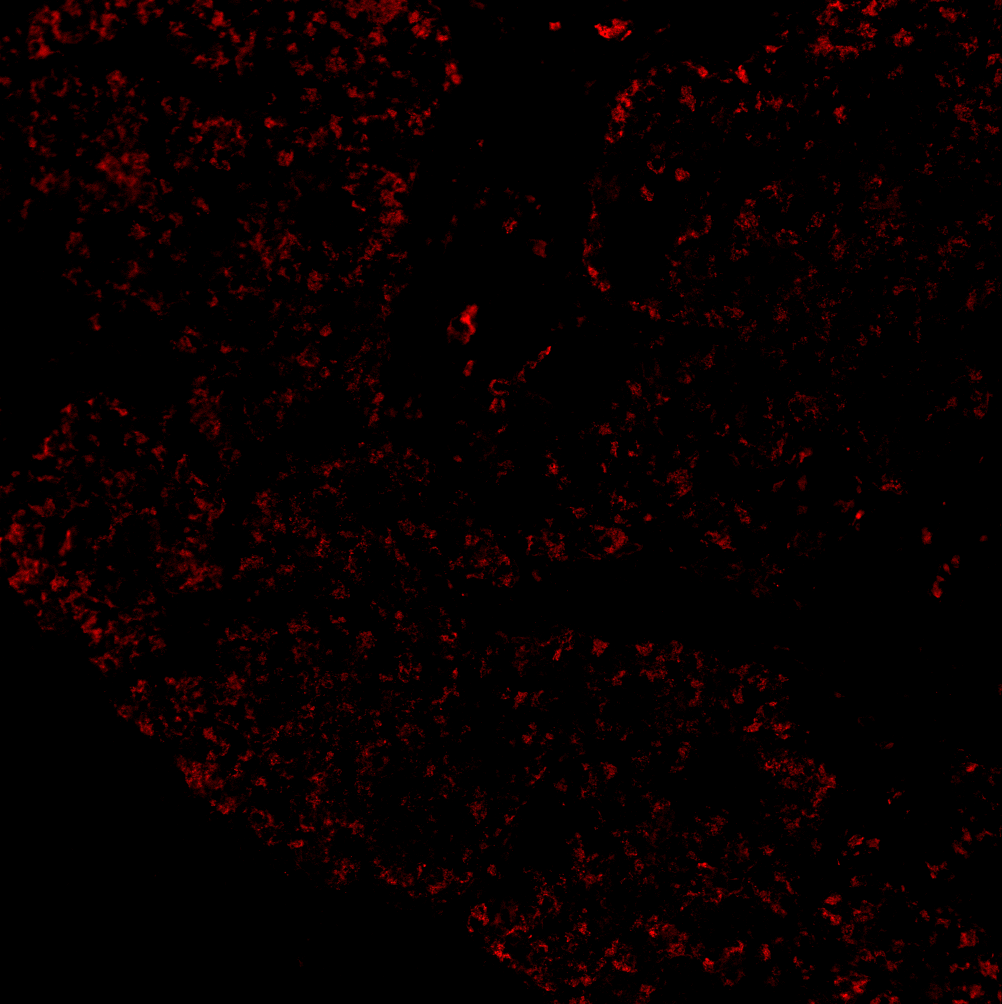

Supplement: Supplementary file 8 — Source Data for Figure 7 [file EMMM-15-e17928-s004.zip › Figure_7/7L_STZ_Ctrl-H_Glucagon.tif]

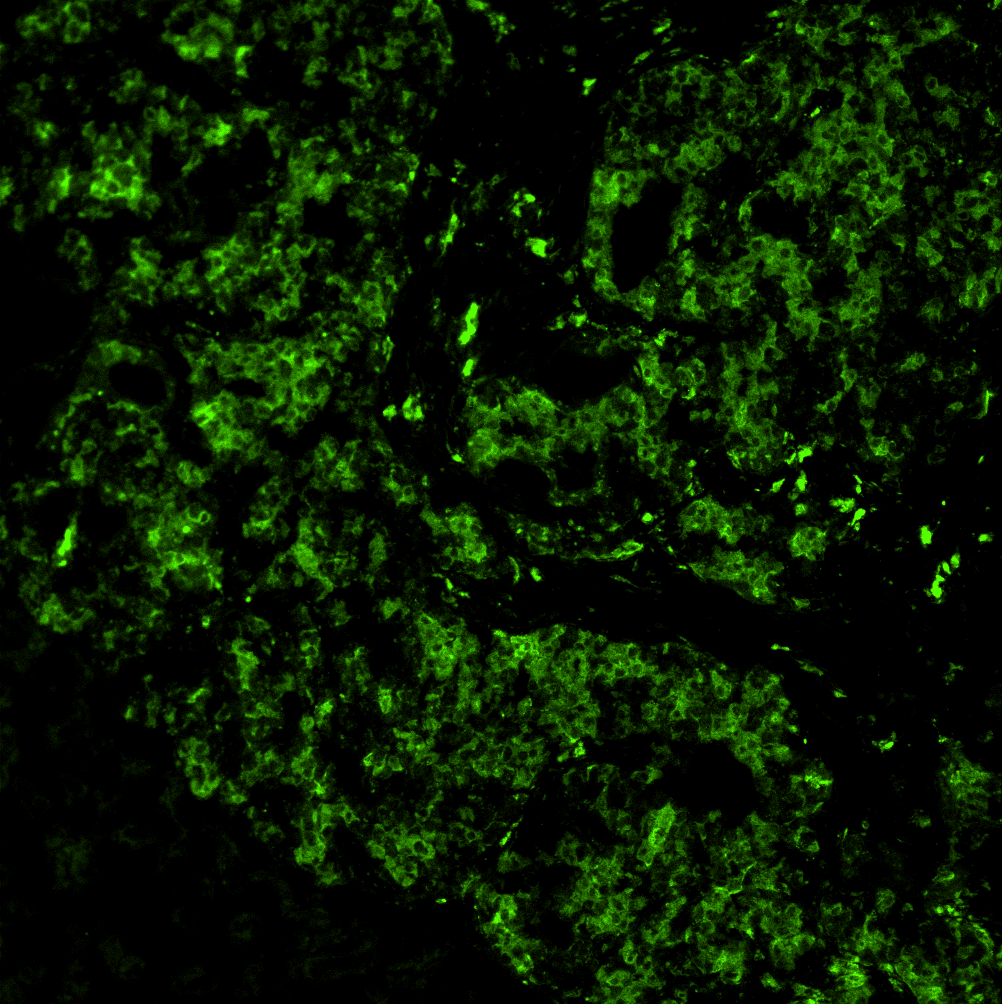

Supplement: Supplementary file 8 — Source Data for Figure 7 [file EMMM-15-e17928-s004.zip › Figure_7/7L_STZ_Ctrl-H_Insulin.tif]

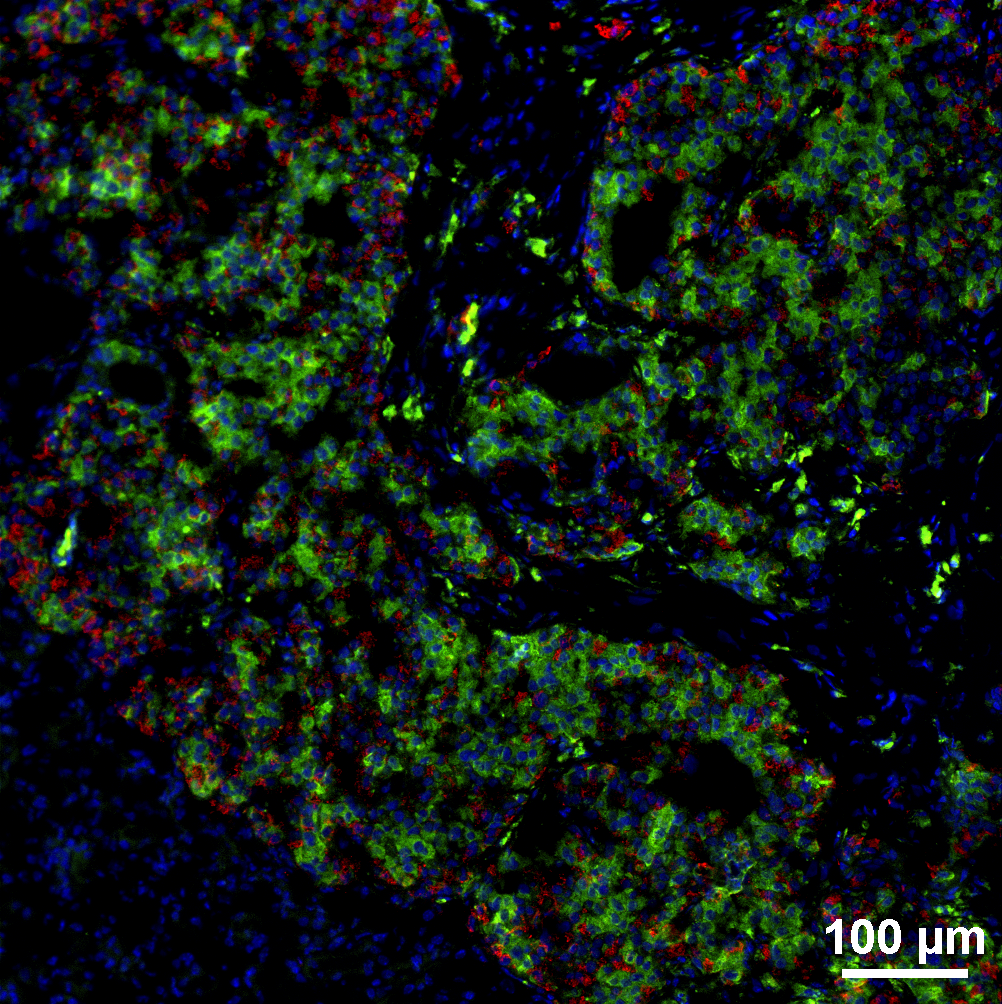

Supplement: Supplementary file 8 — Source Data for Figure 7 [file EMMM-15-e17928-s004.zip › Figure_7/7L_STZ_Ctrl-H_Merged.tif]

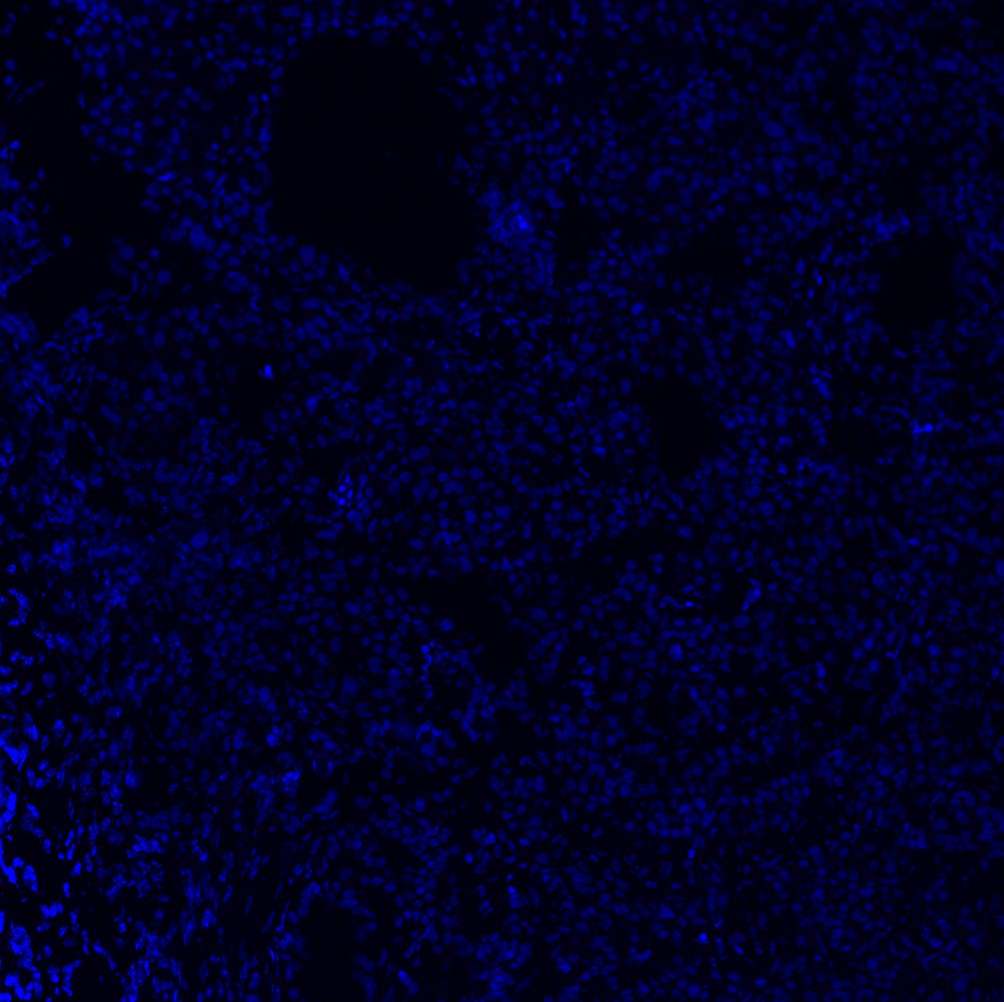

Supplement: Supplementary file 8 — Source Data for Figure 7 [file EMMM-15-e17928-s004.zip › Figure_7/7L_STZ_Ctrl-L_DAPI.tif]

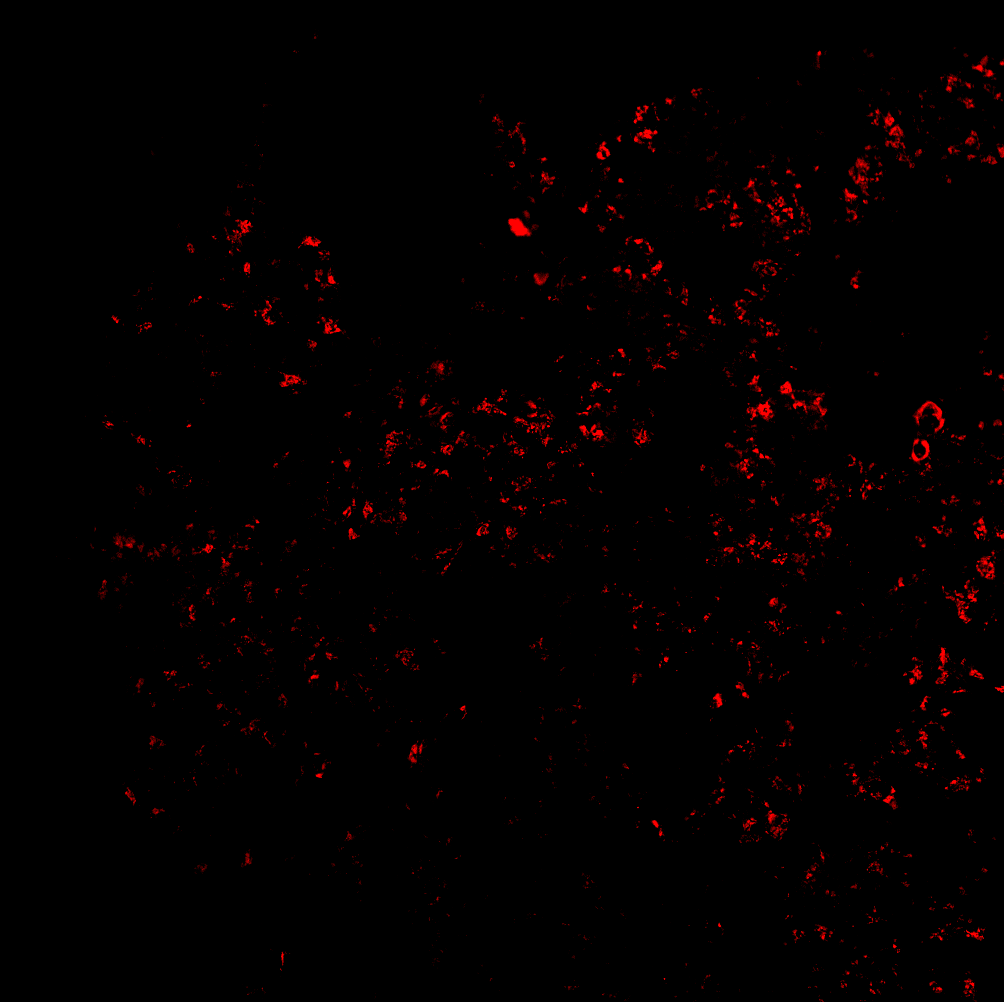

Supplement: Supplementary file 8 — Source Data for Figure 7 [file EMMM-15-e17928-s004.zip › Figure_7/7L_STZ_Ctrl-L_Glucagon.tif]

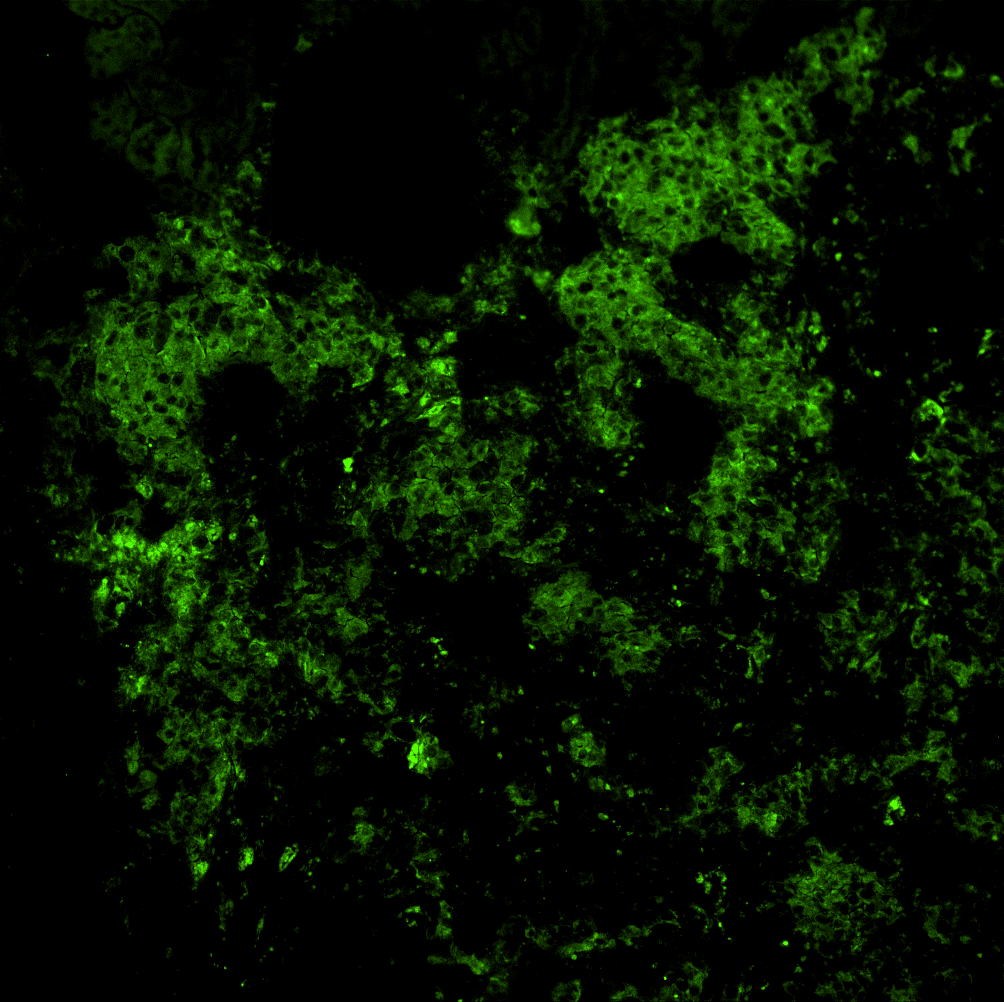

Supplement: Supplementary file 8 — Source Data for Figure 7 [file EMMM-15-e17928-s004.zip › Figure_7/7L_STZ_Ctrl-L_Insulin.tif]

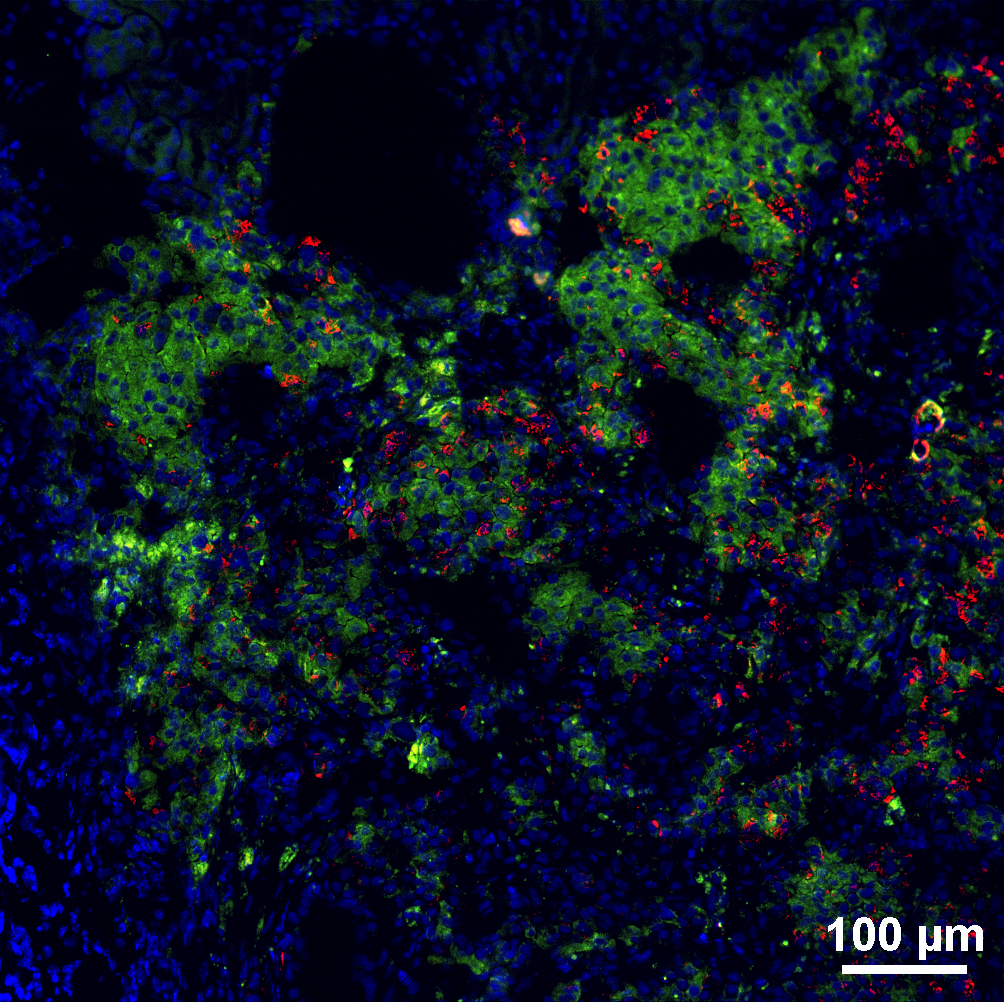

Supplement: Supplementary file 8 — Source Data for Figure 7 [file EMMM-15-e17928-s004.zip › Figure_7/7L_STZ_Ctrl-L_Merged.tif]

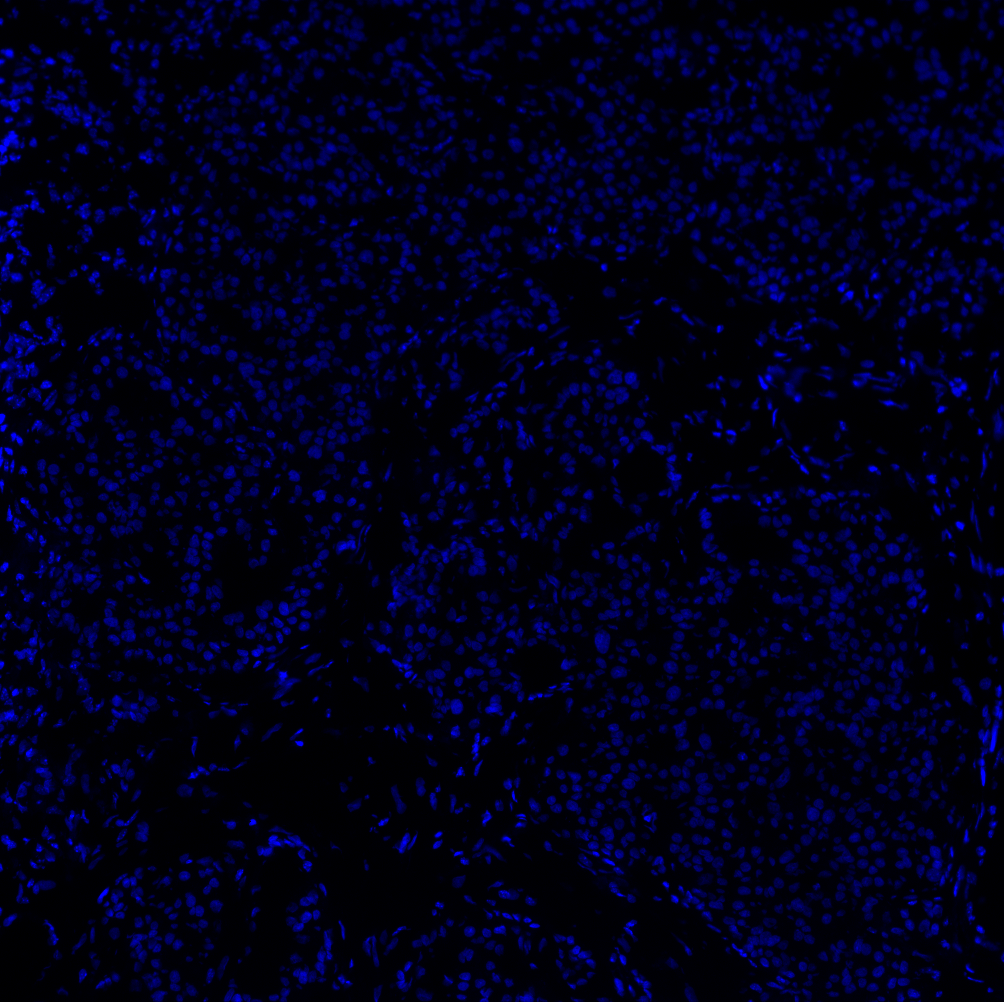

Supplement: Supplementary file 8 — Source Data for Figure 7 [file EMMM-15-e17928-s004.zip › Figure_7/7L_STZ_PAX6-L_DAPI.tif]

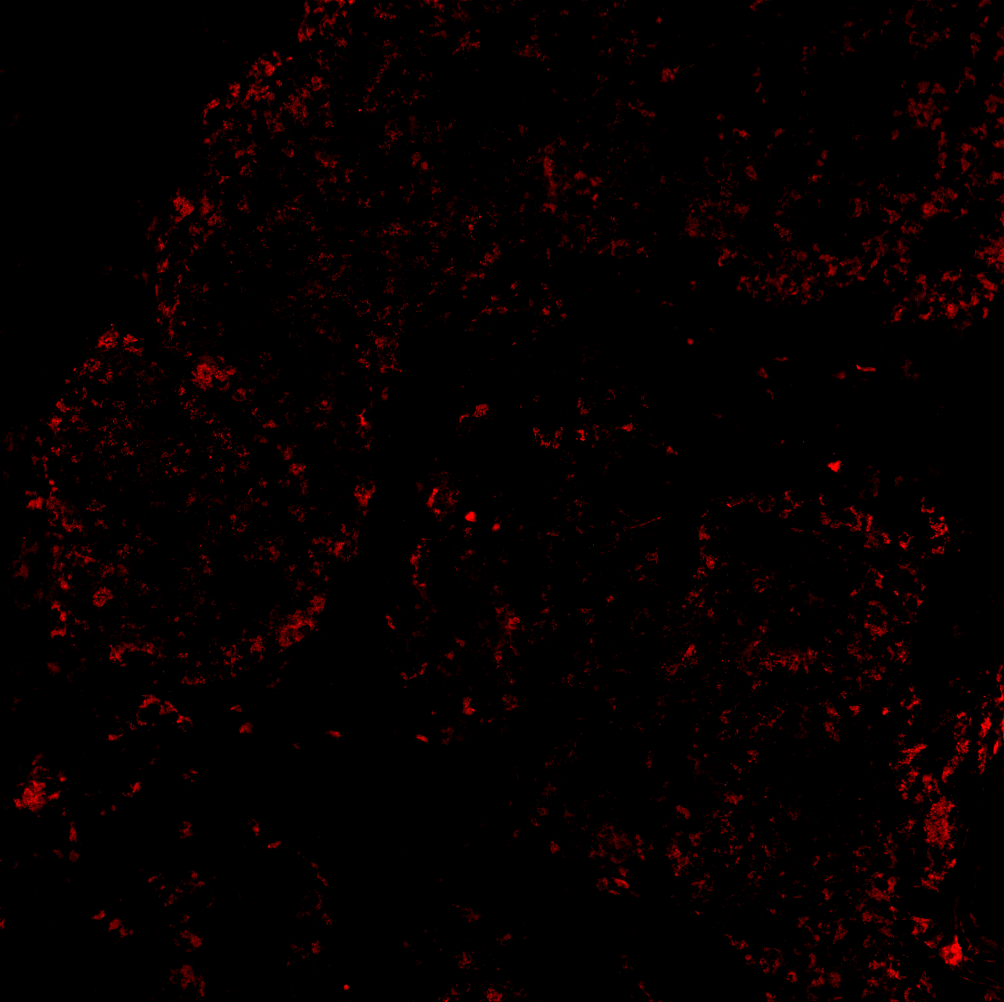

Supplement: Supplementary file 8 — Source Data for Figure 7 [file EMMM-15-e17928-s004.zip › Figure_7/7L_STZ_PAX6-L_Glucagon.tif]

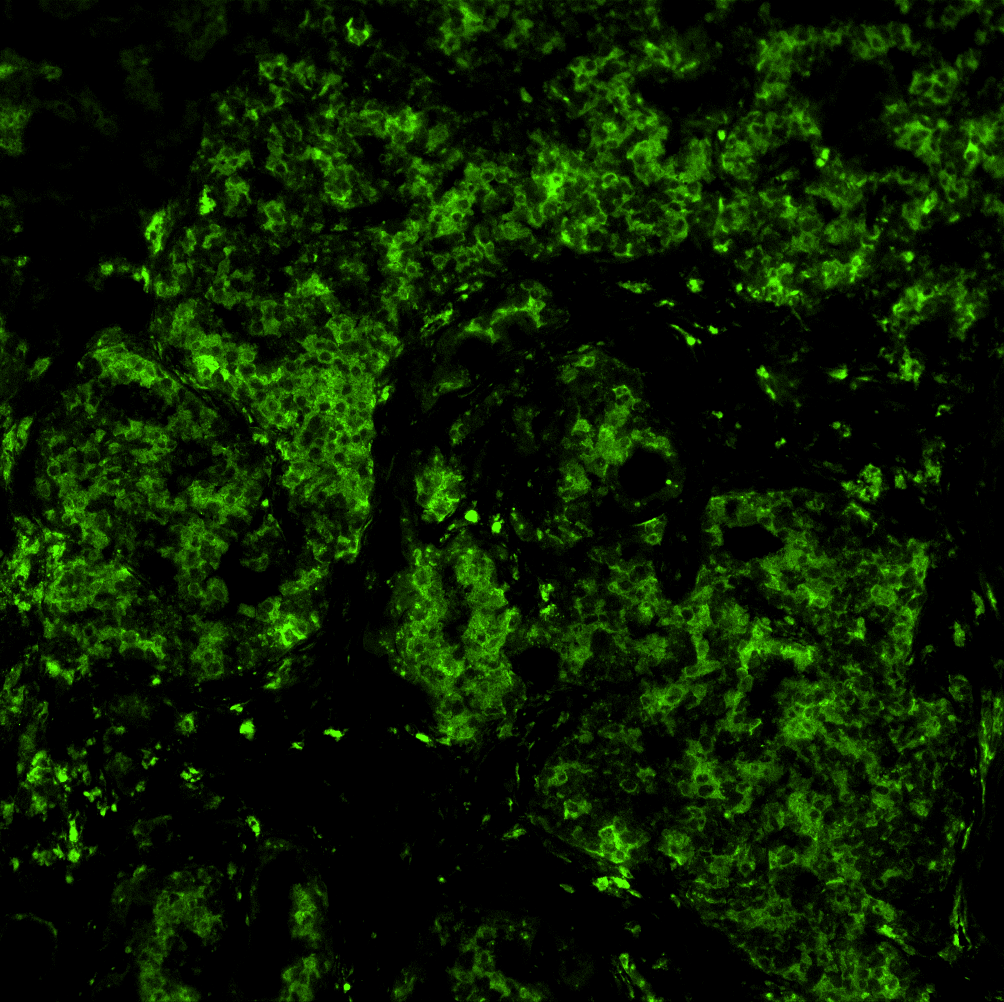

Supplement: Supplementary file 8 — Source Data for Figure 7 [file EMMM-15-e17928-s004.zip › Figure_7/7L_STZ_PAX6-L_Insulin.tif]

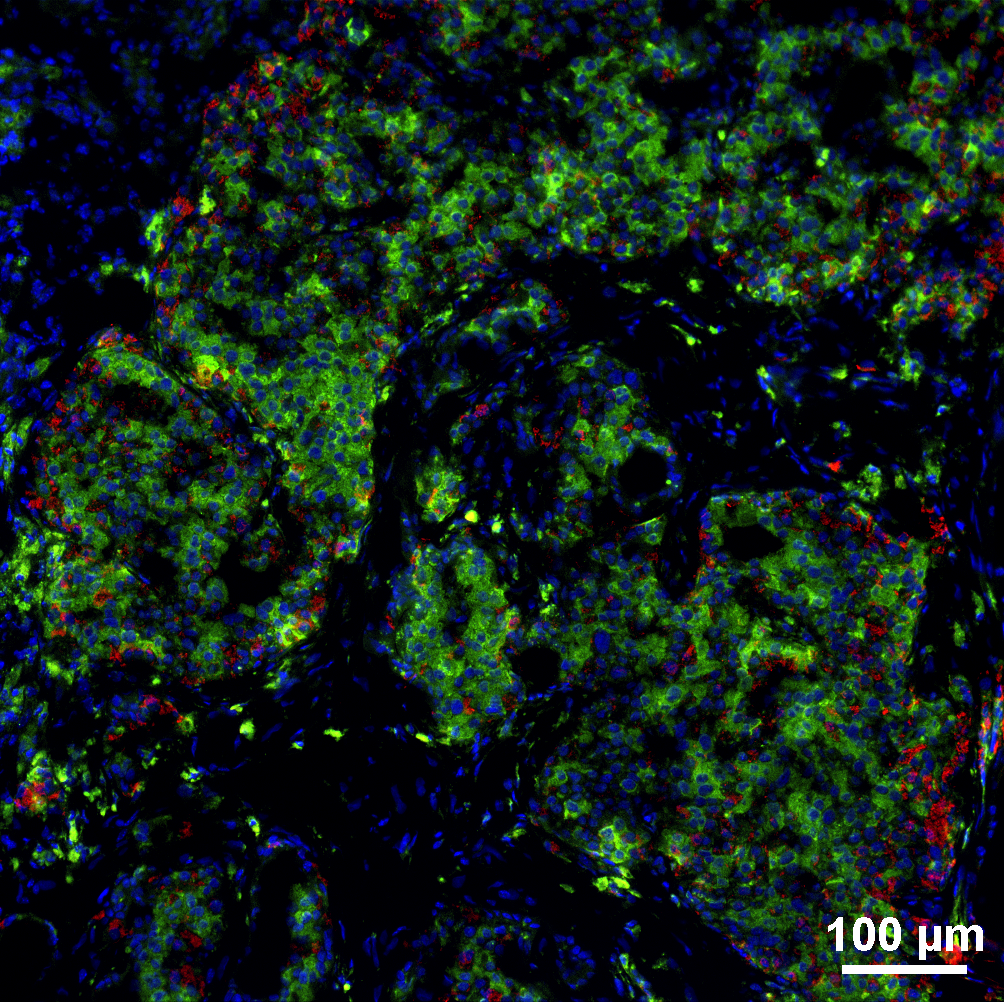

Supplement: Supplementary file 8 — Source Data for Figure 7 [file EMMM-15-e17928-s004.zip › Figure_7/7L_STZ_PAX6-L_Merged.tif]

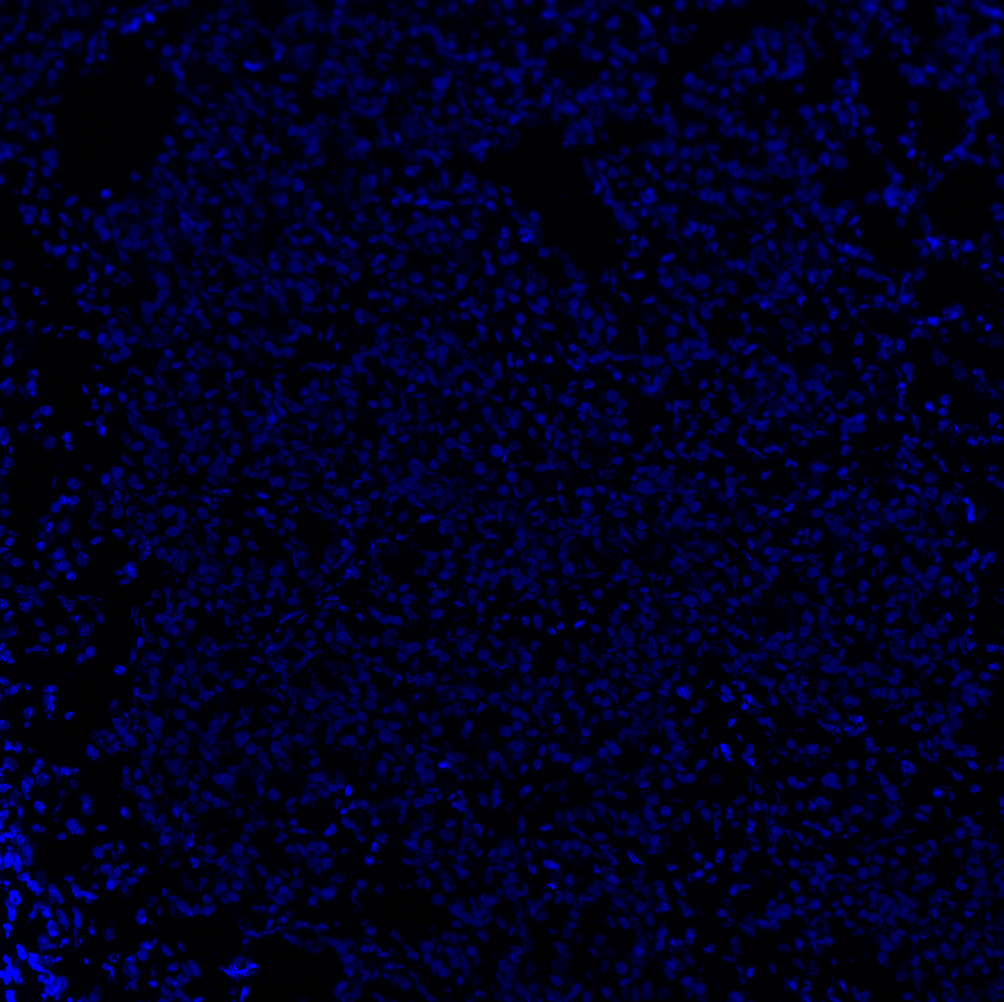

Supplement: Supplementary file 8 — Source Data for Figure 7 [file EMMM-15-e17928-s004.zip › Figure_7/7M_STZ_Ctrl-L_DAPI.tif]

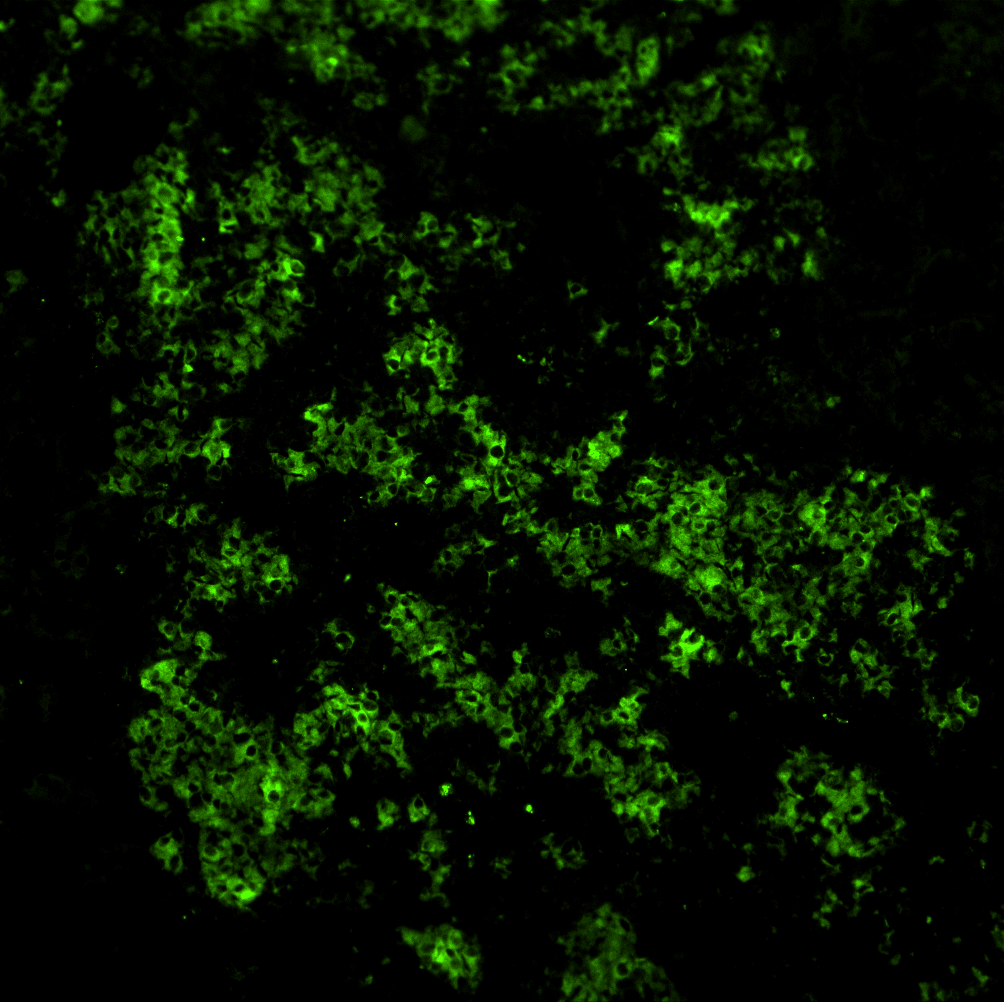

Supplement: Supplementary file 8 — Source Data for Figure 7 [file EMMM-15-e17928-s004.zip › Figure_7/7M_STZ_Ctrl-L_Insulin.tif]

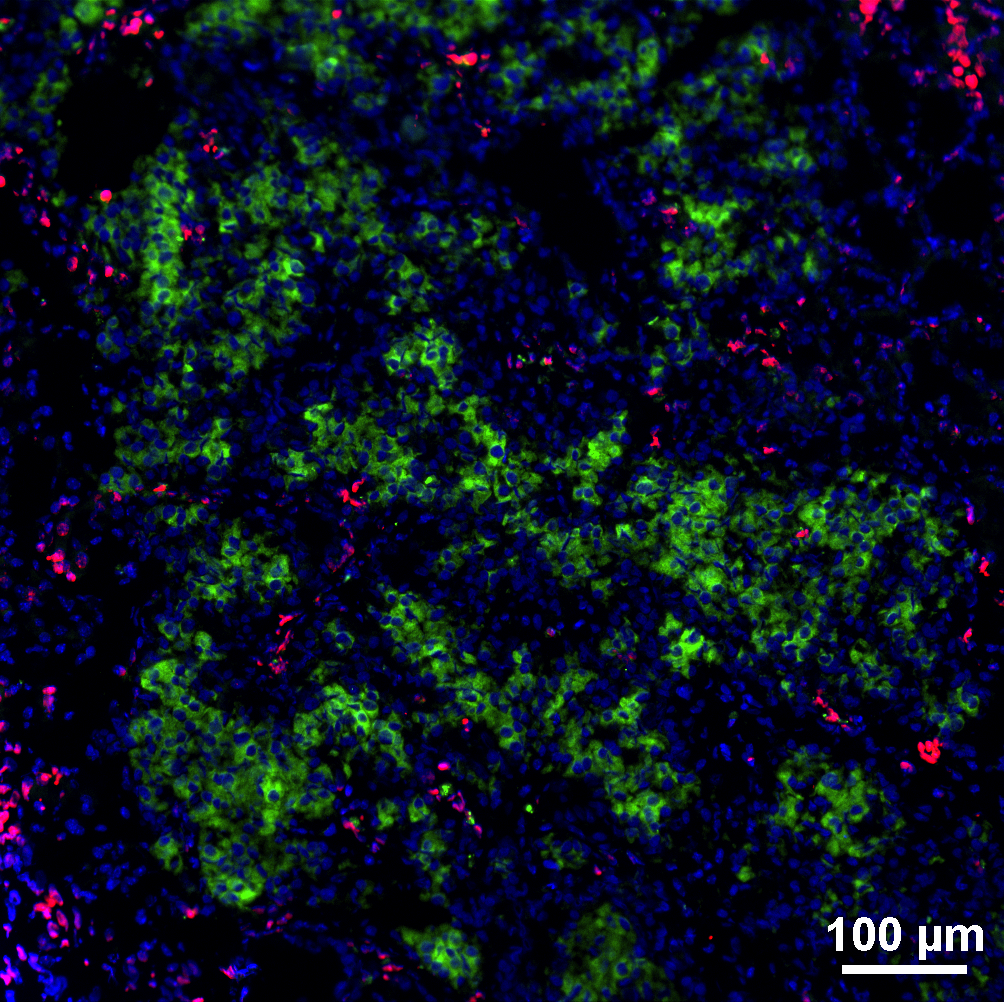

Supplement: Supplementary file 8 — Source Data for Figure 7 [file EMMM-15-e17928-s004.zip › Figure_7/7M_STZ_Ctrl-L_Merged.tif]

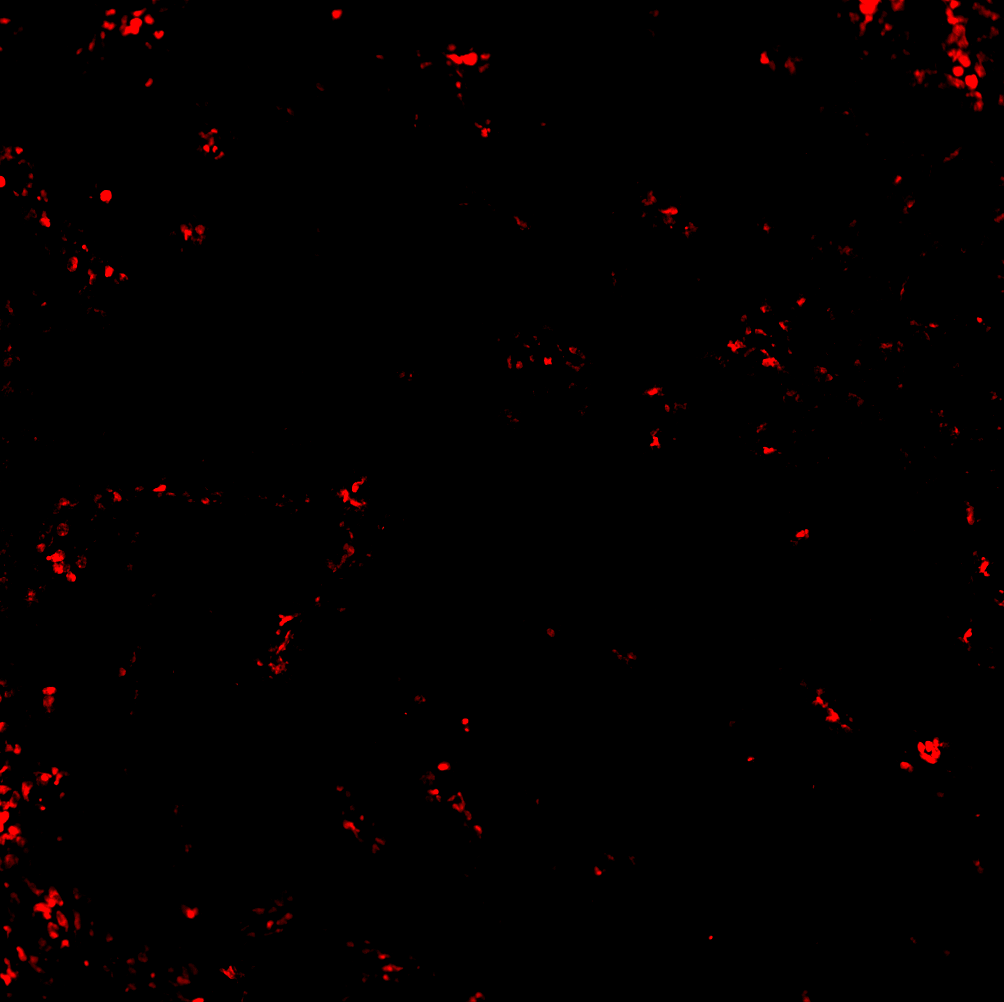

Supplement: Supplementary file 8 — Source Data for Figure 7 [file EMMM-15-e17928-s004.zip › Figure_7/7M_STZ_Ctrl-L_Tunel.tif]

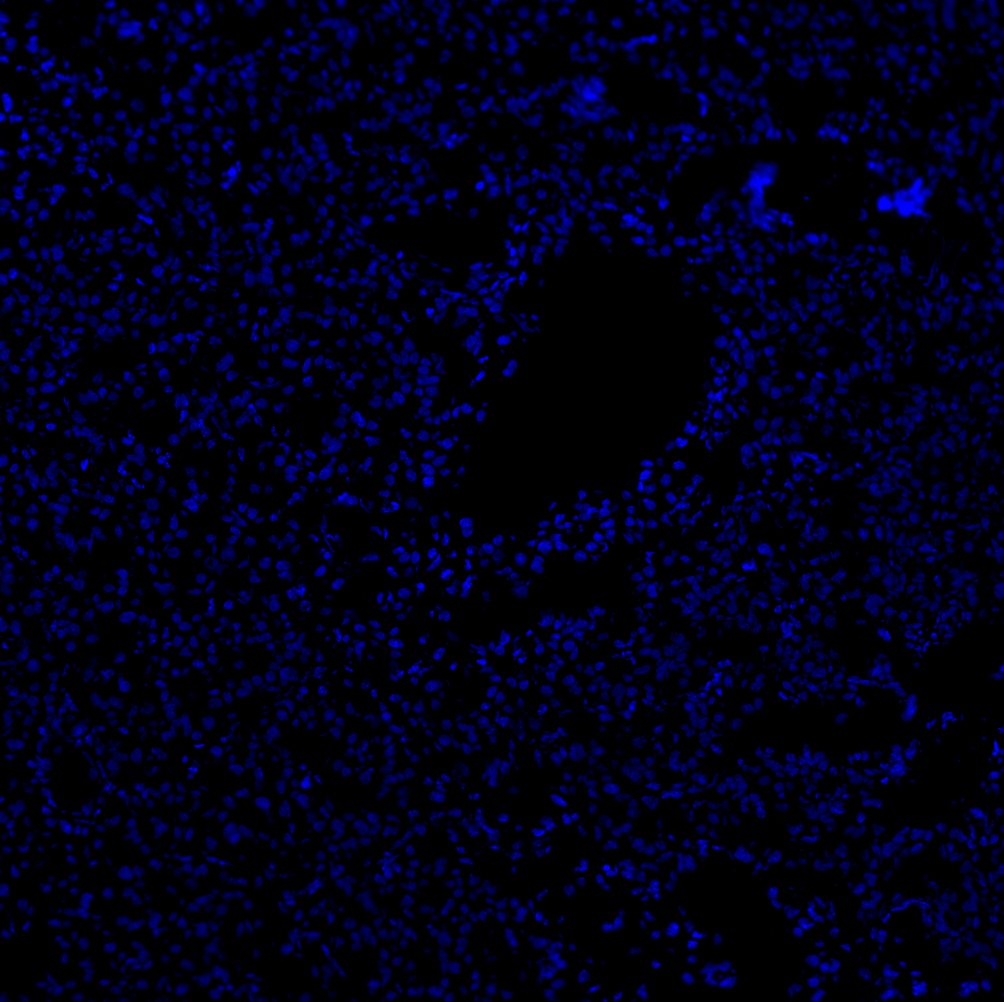

Supplement: Supplementary file 8 — Source Data for Figure 7 [file EMMM-15-e17928-s004.zip › Figure_7/7M_STZ_PAX6-L_DAPI.tif]

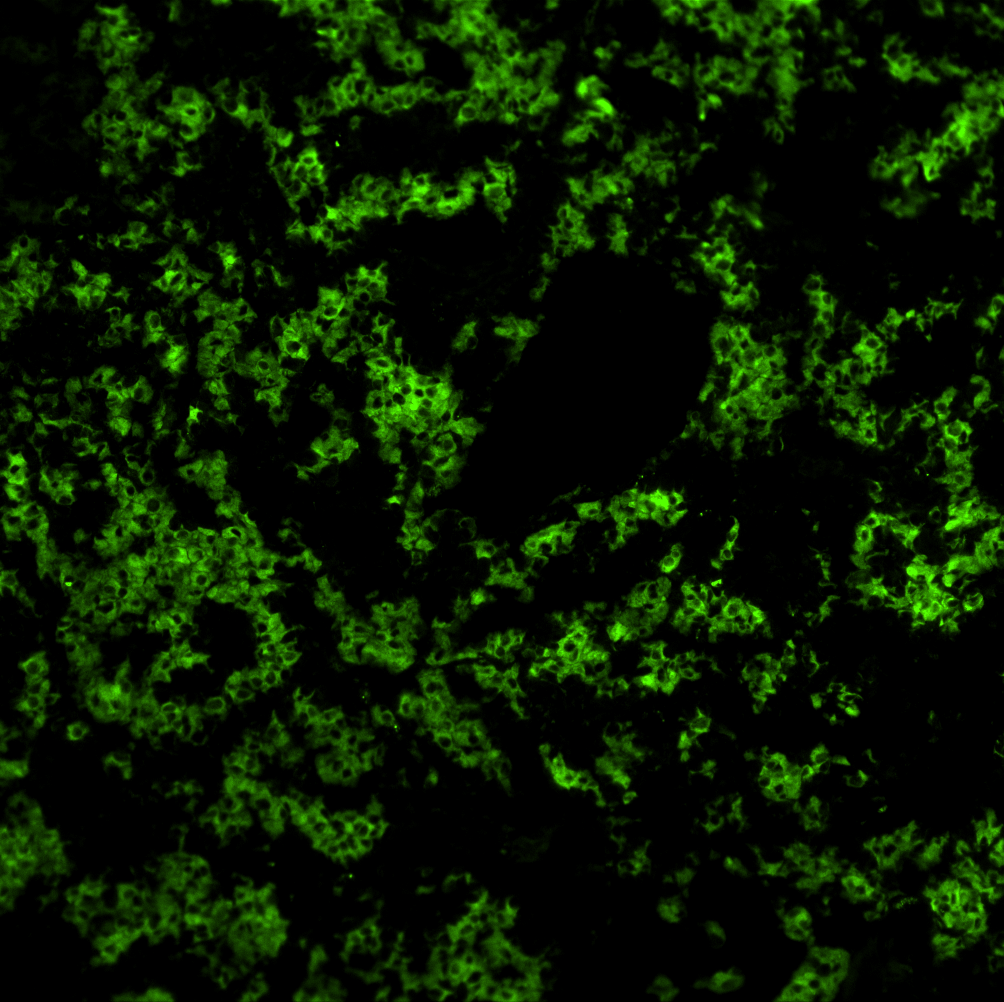

Supplement: Supplementary file 8 — Source Data for Figure 7 [file EMMM-15-e17928-s004.zip › Figure_7/7M_STZ_PAX6-L_Insulin.tif]

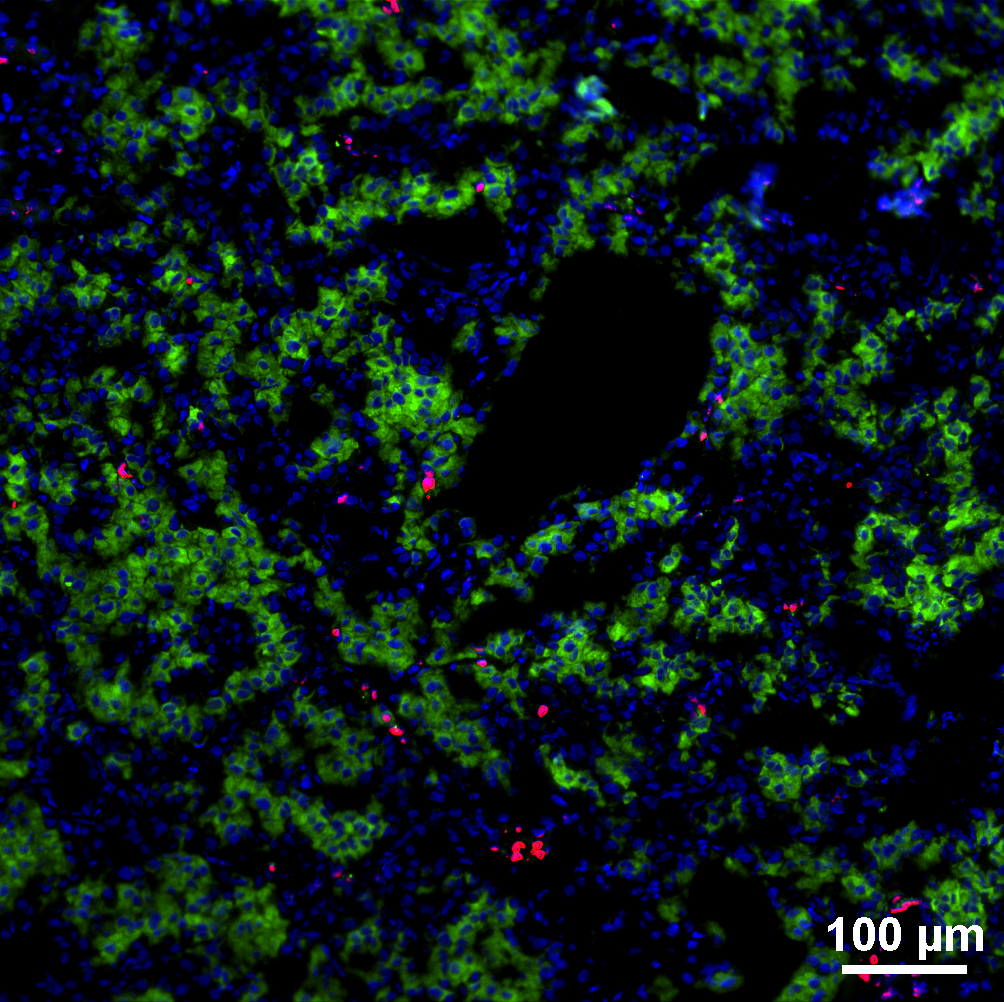

Supplement: Supplementary file 8 — Source Data for Figure 7 [file EMMM-15-e17928-s004.zip › Figure_7/7M_STZ_PAX6-L_Merged.tif]

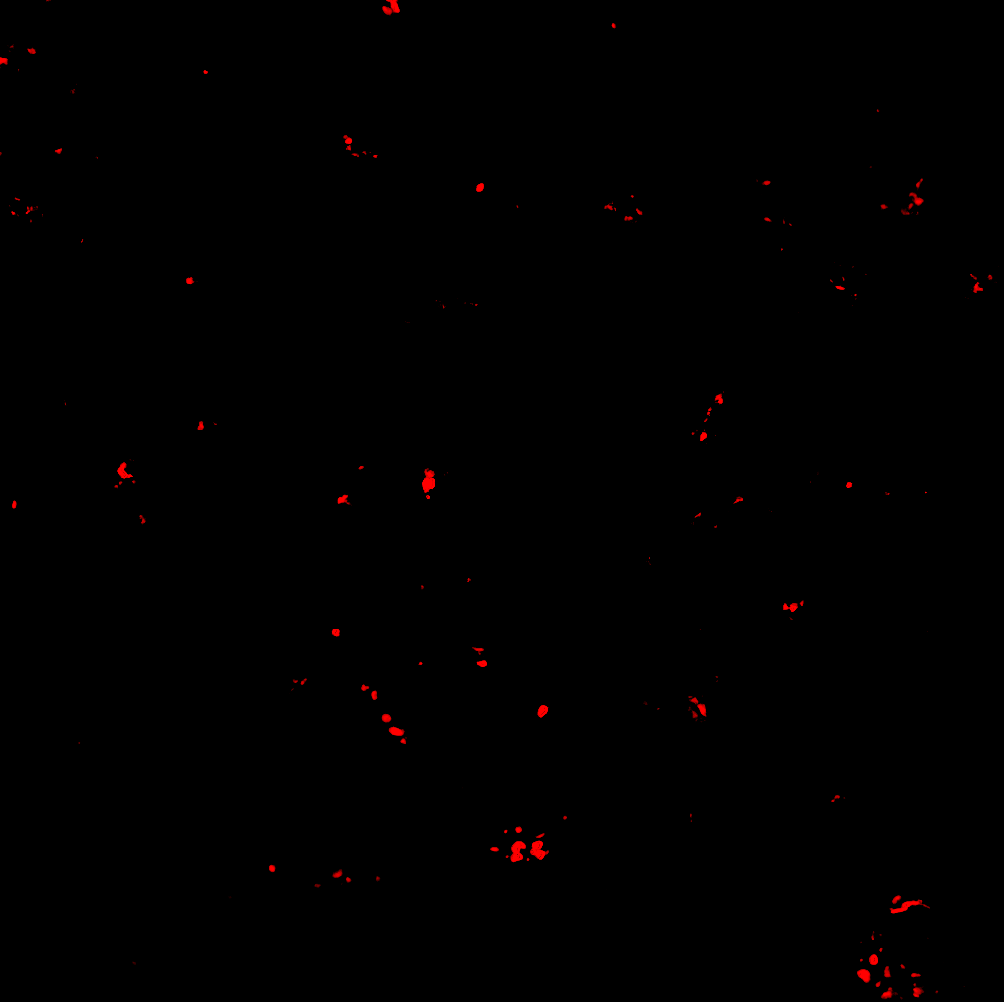

Supplement: Supplementary file 8 — Source Data for Figure 7 [file EMMM-15-e17928-s004.zip › Figure_7/7M_STZ_PAX6-L_Tunel.tif]
