# Supplementary material for: Paired box 6 gene delivery preserves beta cells and improves islet transplantation efficacy
Source: EMBO Mol Med. 2023 Nov 7;15(12):e17928. doi: 10.15252/emmm.202317928 (PMC10701606; doi:10.15252/emmm.202317928)
Supplement: Supplementary file 9 [file EMMM-15-e17928-s009.pdf]

EMBO Molecular Medicine

Paired box 6 gene delivery preserves beta cells and improves islet transplantation efficacy

Wing Yan SO, Yilie Liao, Wai Nam Liu, Guy Rutter, and Weiping Han
DOI: 10.15252/emmm.202317928

Corresponding author: Weiping Han (wh10@cornell.edu) , Weiping Han (wh10@cornell.edu), Wing Yan SO (so_wing_yan@imcb.a-star.edu.sg)

Review Timeline:

Submission Date:	28th Apr 23
Editorial Decision:	22nd May 23
Authors' correspondence:	26th May 23
Editor's correspondence:	26th May 23
Appeal:	8th Jun 23
Editorial Decision:	12th Jun 23
Revision Received:	12th Sep 23
Editorial Decision:	4th Oct 23
Revision Received:	9th Oct 23
Accepted:	13th Oct 23

Editor: Lise Roth

Transaction Report:

(Note: With the exception of the correction of typographical or spelling errors that could be a source of ambiguity, letters and reports are not edited. Depending on transfer agreements, referee reports obtained elsewhere may or may not be included in this compilation. Referee reports are anonymous unless the Referee chooses to sign their reports.)

22nd May 2023

Decision on your manuscript EMM-2023-17928

Dear Prof. Han,

Thank you for the submission of your manuscript to EMBO Molecular Medicine. We have now received feedback from the three referees who agreed to evaluate your work.

As you will see from the enclosed reports, the referees acknowledge the potential interest of the study, however, referees #1 and #2 raise several major concerns such as the incremental novelty, the inadequate T1D model, and missing controls (in particular regarding the AAV strategy). Referee #3 is more positive, but also requires clarifications on the AAV approach.

Given the nature of the referees' concerns and the amount of time and work that would be required to address them, and considering that at EMBO Press we encourage one round of revisions only in a reasonable time frame, I am afraid I see little choice but to return the manuscript to you at this point with the decision that we cannot offer to publish it.

I am very sorry to disappoint you in this occasion, and hope that the referees' comments are helpful in your continued work in this area.

With kind regards,

Lise Roth

Lise Roth
Senior Editor
EMBO Molecular Medicine

***** Reviewer's comments *****

Referee #1 (Comments on Novelty/Model System for Author):

The novelty of the studies is questionable. There is extensive literature on Pax6 and its role in beta cell formation and function, so I'm not sure how much this study adds. Delivery of a transcription factor as a therapeutic approach has been mentioned in the literature for years, but few approaches if any have materialized. Again, the novelty there is also marginal.

Referee #1 (Remarks for Author):

Summary: In this study, the authors argue that Pax6 is a critical regulator of beta cell proliferation and cell death based on data using lentivirus and insulin promoted AAV to either knockdown or overexpress Pax6. Through numerous in vitro and in vivo model systems focusing on islets, the authors show that Pax6 ameliorates T1D (via single, high dose STZ although not adequately explained in methods) and T2D (via db/db or high glucose media, HGPA) disease systems. Moreover, they transplant T2D human islets treated with AAV-Pax6 into STZ treated NSG mice to show functional glycemic control via increased insulin secretion. Bulk transcriptional analysis on control islets (unclear whether KO or WT Pax6 islets) versus AAV-Pax6 overexpressed islets show an increase in genes related to beta cell identity and pancreatic secretion. Islet transplantations in STZ treated NSG mice using islets of lower mass that have been exposed with AAV-Pax6 show similar improvement to the higher mass islets. Overall, the multiple experiments highlight that overexpression of Pax6 results in improved islet function.

Impact: Marginal, owing to the myriad other papers documenting the well-known role of Pax6. Moreover, this study does not account for AAV or lentiviral transfection efficiency issues that have been seen with other pancreatic genes. There may be marginal increase in knowledge depicting Pax6 as beneficial for low mass islets.

Strengths: This paper has multiple in vitro and in vivo methods of testing knockout and overexpression capabilities for AAV mediated constructs. The authors effectively utilize these repetitive techniques throughout the paper to support their hypothesis. The paper is well written with easy flow and the figures as well as figure legends are easy to follow. For the most part, they cite papers that have addressed Pax6 within the field to a reasonable extent.

Criticisms: Although this paper has copious data, the implications are not entirely novel. The introduction is fairly generic,

focusing more on T1D and T2D disease pathogenesis, rather than the background that led to the study of Pax6 vs. other transcription factors (e.g. Pax4). Moreover, the discussion does not include other potential therapeutic methods under development such as stem cell derived islet organoids, CRISPR corrected, or nanoparticle delivery systems. The authors do not discuss how Pax6, itself, enables improved beta cell function (as opposed to increased beta cell replication-which itself may not improve function).

Additional Suggestions:
Figure 1:
• The data from in vivo overexpression would be strengthened if the authors show similar effects of overexpression of Pax6 in EndoC cells
• Explain rationale for why incretin hormones are used
o Is proliferation being used as a measure of beta cell vitality? Why? A bit confusing given apparent lack of beta cell proliferation in adults
o Insulin secretion data would strengthen the studies
Figure 3:
• T1D modeling via STZ usually requires multiple low doses; as presented the authors are using high dose to ablate β cells. This is not really a T1D model (more a model of insulin deficiency), and to present it as such is questionable. A NOD mouse model with Pax6 overexpression would strengthen this claim.
• The increase in beta cell proliferation and reduction in death should result in an increase in beta cell mass, but these data are not presented.
• Panel I quantifies Ins+ Ki67+ cells to measure beta cell proliferation; should panel J quantify cells that are both TUNEL+ and Ins+ to measure beta cell apoptosis?
Figure 4:
• The striking differences observed in the overexpression (A-H) does not appear to be reflected by the small differences observed in proliferation and death (I-J); again, presentation of beta cell mass would be helpful.
Figure 5:
• Glucose stimulated insulin secretion (GSIS) to show effect of overexpression would strengthen the study.
Figure 7:
• Please clarify control group being used for RNA sequencing on the figure itself, and in Results section (listed as just Ctrl- are these AAV-Ctrl?)
• Panel A-D: p-value adjustment for multiple hypothesis testing?/FDR?
• Panel G: are the listed pathways significantly enriched/overrepresented? Suggest showing GO analysis, statistics
• Any cell cycle genes that are altered?
• Please show PCA plot to show variance between samples
• Discuss why only 20% of genes are similar between CHIP and OE; what are the other genes doing in relation to beta cells

Referee #2 (Remarks for Author):

The manuscript by So et al describes how Pax6 is important for beta-cell biology proliferation and survival. They present data that show that Pax6 overexpression may help treat diabetes both in mice models mimicking type 1 or type 2 diabetes. Moreover, they present data showing that Pax6 overexpression in human islets improve their function when transplanted in diabetic mice. The results are interesting and suggest that Pax6 overexpression may be part of new therapeutic strategies, at least in islet graft. Yet, they are several concerns about controls and experiments that are not presented. Thus, serious modifications of the present manuscript are required to improve the quality of the manuscript.

A main issue on the use of the AAV to overexpress Pax6 is to show that it actually targets beta cells and no other tissues. Controls of Pax6 expression in different tissues need to be shown. In addition, Pax6 overexpression in control normoglycemic mice, not just in diabetic mic, needs to be shown. In control mice, does Pax6 overexpression lead to a better glucose homeostasis?

Another main issue is the quantification of rare events. Proliferation and apoptosis rates were measured in alpha and beta cells in vivo, in STZ mice and Db mice. These events are rare, thus the positive cells (for example ki67+glucagon or TUNEL+insulin) are scarce. To obtain a precise measurement, it is required to count a minimum of these scarce events. For example, a bias can be easily made if a too low number of events is counted: 1 cells out of 1000 would suggest a rate of 0.1% while 2 cells out of 1000 would suggest a rate of 2% and a doubling of the rate with just one extra positive cell. The authors do not mention how many events where counted in each measurement and that is absolutely required.

On figure 1, it would be very logical to show Pax6 KD before showing the effects on the cells. It is a required control that have to be shown first to prove the efficiency of the KD.

Again, figure 1 needs to be re-organized: quantifications of WB need to be called as panels. Analysis of pAkt needs to display whether basal pAkt levels (not just insulin-stimulated) are different when Pax6 is KD.

Figure S1 may include the expression levels of more transcription factors, including Pax4, Tcfl2, hnf6 and hnf4alpha isl1 and neurod
The experience with HGPA needs to be more documented: what are the glucose levels normally required to grow the cells? What are the glucose levels for HGPA? Glucose-stimulated insulin secretion should be measured on endobetaH1 cells in the different conditions.
There is a need fora statistical analysis in between ctrl and HGPA cells. It is absolutely mandatory to describe properly the effect of HGPA. During HGPA, culture conditions may force cells to secrete more insulin thus the decreased ins content may reflect improved secretion. Therefore, insulin content must be related to GSIS.

Similarly to figure 1, the panels of figure 2 need to be re-organized. The control of Pax6 overexpression need to be shown first before the panels on proliferation and apoptosis. Also, the condition ctrl+pax6 overexpression is interesting and needs to be shown on figures.
Please comment on how calculations for ratios of pAkt were done. Is there a normalization to total akt and to GAPDH and to control condition ? Please explain.
It seems that pa6 overexpression reverts the effects of HGPA. Is it also true with Pax4 or pdx1 overexpression ? The question about the specificity of action of pax6 is central.
STZ mice are not a T1D like phenotype. STZ mice are a model of diabetes to a lack of insulin (after chemical destruction of beta cells) but without auto-immune attack. Please correct.

The expression of pax 6 was measured on whole pancreas. Since stz kills beta cells, the number of beta cells is decreased in the stz+aav-ctrl group.thus number of pax6 expressing cells decreases, hence loss of pax6. where is the ctrl + pax6 aav group ?

In STZ mice and in Db/db mice, AAVPax6 treatment may modify beta-cell mass. Assessment of beta-cell mass needs to be performed in these mice.

A more precise description of the STZ model is needed. What is the dose of STZ ? Is it a one shot of STZ or many shots ? Is there a total or a partial destruction of beta cells ?

In STZ mice, pax6 is overexpressed in beta cells and stimulates proliferation but the stimulation of proliferation is not impressive but seems to be sufficient to restore beta cells and produce insulin. Please comment on this result.

TUNEL levels are very high in all groups while apoptosis in beta cells is supposed to be a very low level. Also, pictures of IF for TUNEL suggest that apoptotic cells are not beta cells but other cells. How was the co staining for TUNEL and insulin quantified ?

Was alpha cell mass measured in groups of mice with modified blood glucagon levels ?

MafA IF should be nuclear but the picture show a weird staining in some cells. Please improve IF.

What is the phenotype of human normal islets + AAVPax6 ? Again, this group is missing and could provide interesting informations.

Line 262, the authors claim that "transplantation of T2D islets transduced with AAV-Ctrl (STZ/T2D-Ctrl) modestly normalized the blood glucose level". If glycose returns to a normal value, then it is called normalized, otherwise, it is decreased comparerd to control group. Modestly normalized is a non sense.

Insulin contents are compared in between STZ/Ctrl-L and STZ/L+Pax6 but it should also be compared to STZ/Ctrl-H.

Referee #3 (Remarks for Author):

The manuscript entitled "Pax6 gene delivery preserves beta cells and improves islets transplantation efficacy" by Wing Yan So, et al., describes the role of modulating Pax6 expression in pancreatic islets to preserve/restore beta cell function in different models of T2D, including preclinical (Db/Db, STZ treated mice) and human control and T2D islets, engrafted in STZ mice. This is an important study unravelling that modulation of Pax6 expression (i.e. increasing its levels) improves beta cell function. This is a well controlled and designed study, using pre-clinical relevant models, where functional tests are associated to molecular characterization of Pax6 over expression. The in vivo preclinical studies are clearly demonstrating that maintaining Pax6 levels may contribute to improve beta cell function/dedifferentiation during T2D.

I have some comments concerning the study.

Concerning the AAV8 approach, the authors do not describe why they have used this serotype, and whether the AAVs infect both human and mouse pancreatic islets. Is the infection specific to pancreatic islets or are other tissues also affected after

AAVs injection? How efficient is the transaction of AAVs in the middle of pancreatic islets?

It is interesting to observe that Pax6 over expression modulates proliferation and/or apoptosis, in response (or not) to Insulin or Glp1. In the RNA-seq/ChIP-seq integrated analysis, none of the proliferative nor apoptosis pathways were found to be regulated by Pax6, although this seems to be the major functional effect (and, consequently, subsequent insulin secretion effect). The authors should, at least, discuss this point.

As a service to authors, EMBO provides authors with the possibility to transfer a manuscript that one journal cannot offer to publish to another EMBO publication. The full manuscript and if applicable, reviewers reports are automatically sent to the receiving journal to allow for fast handling and a prompt decision on your manuscript. For more details of this service, and to transfer your manuscript to another EMBO title please click on Link Not Available

Please do not share this URL as it will give anyone who clicks it access to your account.

Dear Dr. Lise Roth,

Thanks for the message and the decision along with the comments from the reviewers. We have studied these comments and would like to discuss with you on the possibility of a reconsideration of the decision.

First, regarding the comments from the reviewers. We greatly appreciate the time and insights from all the reviewers, and would like to summarise the main points and our response/plans below:

The main criticism of Reviewer #1 is on the novelty of the study due to the extensive literature on PAX6 and its role in beta cell formation and function.

Those extensive studies on PAX6 are mainly in mouse beta cells using mutant mice (PMID 15110714, 9224716, 26658466, 27941241, 23326594, 28377501). These studies show that PAX6 is physiologically essential for normal mouse islet development and function using a loss-of-function approach. It is well documented that species differences do exist between human and rodent islets in terms of metabolism, cytoarchitecture and functional implications (PMID 21454710, 28422162, 16461897), which may influence the translatability of mouse studies to the human context. On top of using mouse models, we use human islets in our study to demonstrate the human relevance. Moreover, it is generally recognized that the capability of adult human beta cell replication or regeneration is very limited when compared to rodent beta cells (PMID 22751699), and there is currently no clinical therapy proven to stimulate beta cell proliferation or preserve beta cell mass once beta cell failure has ensued (PMID 27189025). Hence, strategies that can preserve beta cells, even in a modest manner, are valuable. With the use of cell line, mouse models and human islets, our results highlight previously unappreciated roles for PAX6 in maintaining human islet survival and the contribution to diabetes management. Although essential, understanding the physiological role of the critical beta cell regulators is just the first step. The ultimate goal is to develop therapeutic strategies by targeting those regulators, which is not addressed by any of the aforementioned studies. In our study, we apply various in vitro and in vivo models to demonstrate the therapeutic effects of AAV-mediated PAX6 gene delivery on beta cell preservation and diabetes management. In particular, we show that PAX6 gene delivery enhances both normal and T2D human islet potency, and thus might overcome two of the major unresolved barriers of clinical islet transplantation: post-transplant islet graft loss and scarcity of islet donors. By providing scientific basis to support targeting PAX6 to overcome beta cell failure, our study hence shows clinical relevance which has not been demonstrated before.

For Reviewer #2, the first main issue is to include a group of AAV-PAX6 overexpression in normal mice and on the other hand show that the expression does not occur in other tissues. We did not include the Ctrl + AAV-PAX6 group in our first draft as demonstrating the therapeutic potential of AAV-PAX6 in diseased mice would be more meaningful and in real practice, none of

the medications will be prescribed to normal subjects. We can provide those data in the revised version as requested. The second main issue is the quantification of Ki67 and TUNEL signals. For STZ mouse pancreas sections, we quantify 8-10 pancreas sections (each contains at least 3 islets) for each mouse and 6 mice for each group. For db/db mouse and human islet sections, we quantify 4-5 sections (each section contains at least 10 islets) for each batch of islets and 4-6 batches of islets for each group. For islet graft-bearing kidneys, we quantify 4-5 sections for each mouse and 4-6 mice for each group. The number of mice/ batches of islets is indicated in the figure legend. The details can be added in the method section in the revised version.

For the additional experiments suggested, we believe that they can be performed within a reasonable period of time.

We hope that these responses provide additional evidence that our study indeed offers significant insights, and the requested experiments could be completed within a reasonable time period. Could we request that you reconsider your decision and allow a revised version to be submitted for consideration of publication?

Best wishes,

Weiping

Dear Prof. Han,

Thank you for your email and request to reconsider our decision on your manuscript.
In order to make a formal appeal, I would suggest that you provide a detailed provisional point-by-point rebuttal letter in response to all referees' concerns.
This would allow us to contact the original referees or an advisor and to make an informed decision.

Let me know if this is agreeable to you.

With kind regards,

Lise Roth

Dear Dr. Roth,

As promised, please find below our point-to-point response to the reviewers' comments.

Referee #1 (Comments on Novelty/Model System for Author):

The novelty of the studies is questionable. There is extensive literature on Pax6 and its role in beta cell formation and function, so I'm not sure how much this study adds. Delivery of a transcription factor as a therapeutic approach has been mentioned in the literature for years, but few approaches if any have materialized. Again, the novelty there is also marginal.

A: The referenced studies on PAX6 are mainly in mouse beta cells using mutant mice (PMID 15110714, 9224716, 26658466, 27941241, 23326594, 28377501). These studies show that PAX6 is physiologically essential for normal mouse islet development and function using a loss-of-function approach. It is well documented that species differences exist between human and rodent islets in terms of metabolism, cytoarchitecture and functional implications (PMID 21454710, 28422162, 16461897), which may influence the translatability of mouse studies to the human context. In contrast, we use human islets in our study to demonstrate the human relevance besides the experiments with mice. Moreover, it is generally recognized that the capability of adult human beta cell replication or regeneration is very limited when compared to rodent beta cells (PMID 22751699), and there is currently no clinical therapy proven to stimulate beta cell proliferation or preserve beta cell mass once beta cell failure has ensued (PMID 27189025). Hence, strategies that can preserve beta cells, even in a modest manner, are valuable. With the use of cell line, mouse models and human islets, our results highlight previously unappreciated roles for PAX6 in maintaining human islet survival and the contribution to diabetes management. While it is important to understand the physiological role of the critical beta cell regulators, the ultimate goal is to develop therapeutic strategies by targeting those regulators, which is not addressed by any of the aforementioned studies. In our study, we apply various *in vitro* and *in vivo* models to demonstrate the therapeutic effects of AAV-mediated PAX6 gene delivery on beta cell preservation and diabetes management. In particular, we show that PAX6 gene delivery enhances both normal and T2D human islet potency, and thus might overcome two of the major unresolved barriers of clinical islet transplantation: post-transplant islet graft loss and scarcity of islet donors. By providing scientific basis to support targeting PAX6 to overcome beta cell failure, our study hence shows clinical relevance which has not been demonstrated before.

Referee #1 (Remarks for Author):

Summary: In this study, the authors argue that Pax6 is a critical regulator of beta cell proliferation and cell death based on data using lentivirus and insulin promoted AAV to either knockdown or overexpress Pax6. Through numerous in vitro and in vivo model systems focusing on islets, the authors show that Pax6 ameliorates T1D (via single, high dose STZ although not adequately explained in methods) and T2D (via db/db or high glucose media, HGPA) disease systems. Moreover, they transplant T2D human islets treated with AAV-Pax6

into STZ treated NSG mice to show functional glycemic control via increased insulin secretion. Bulk transcriptional analysis on control islets (unclear whether KO or WT Pax6 islets) versus AAV-Pax6 overexpressed islets show an increase in genes related to beta cell identity and pancreatic secretion. Islet transplantations in STZ treated NSG mice using islets of lower mass that have been exposed with AAV-Pax6 show similar improvement to the higher mass islets. Overall, the multiple experiments highlight that overexpression of Pax6 results in improved islet function.

Impact: Marginal, owing to the myriad other papers documenting the well-known role of Pax6. Moreover, this study does not account for AAV or lentiviral transfection efficiency issues that have been seen with other pancreatic genes. There may be marginal increase in knowledge depicting Pax6 as beneficial for low mass islets.

A: Our results have shown that the lentivirus and AAV approaches efficiently deliver PAX6 into cell line (fig. 2D), mouse beta cells/ islets (fig. S2A, S2B, 4K) and human islets (fig. S5E) as shown by the significant increases in PAX6 protein levels.

Strengths: This paper has multiple in vitro and in vivo methods of testing knockout and overexpression capabilities for AAV mediated constructs. The authors effectively utilize these repetitive techniques throughout the paper to support their hypothesis. The paper is well written with easy flow and the figures as well as figure legends are easy to follow. For the most part, they cite papers that have addressed Pax6 within the field to a reasonable extent.

Criticisms: Although this paper has copious data, the implications are not entirely novel. The introduction is fairly generic, focusing more on T1D and T2D disease pathogenesis, rather than the background that led to the study of Pax6 vs. other transcription factors (e.g. Pax4). Moreover, the discussion does not include other potential therapeutic methods under development such as stem cell derived islet organoids, CRISPR corrected, or nanoparticle delivery systems. The authors do not discuss how Pax6, itself, enables improved beta cell function (as opposed to increased beta cell replication-which itself may not improve function).

A: PAX4 is a key regulator for beta cell development and maturation during embryogenesis. Although still detectable, PAX4 expression is low in adult beta cells (PMID 18234907). Acute overexpression of PAX4 has been found to protect beta cells against stress-induced apoptosis and stimulates proliferation. However, sustained PAX4 expression results in beta cell dedifferentiation (repression of MAFA and insulin), blunted glucose-stimulated insulin secretion and glucose intolerance (PMID 21521872). Besides, transcript levels of PAX4 are increased in human T2D islets (PMID 17989064), which also suggests that PAX4 upregulation triggers beta cell dedifferentiation in T2D and explains why PAX4 expression is normally suppressed in adult beta cells. These observations make PAX4 not a suitable therapeutic target.

PAX6 is found to be down-regulated in human T2D islets (PMID 22768844, 34193609) while PAX6 gene mutations are associated with diminished insulin secretion and glycemic

perturbations in human (PMID 30151985, 15842522, 11756345). These observations suggest a strong correlation between PAX6 activity/ levels and human diabetes. Mechanistic studies are mainly done on mice, those studies suggest PAX6 critically regulates mouse beta cell function but its roles in human beta cells remain ambiguous. These justify the study of PAX6 in human islets.

Induced pluripotent stem cells (iPSC) are not yet suitable for large-scale production of patient-specific iPSC-beta cells for transplantation since individuals require several hundred thousand individual pancreatic islets. In addition, unlike whole pancreas or islet transplantation, iPSC-derived beta cells do not replace the lost function of alpha cells. Deficiency in glucagon production precipitates hypoglycemia unawareness. Until these challenges are addressed, transplantation of islets from cadaveric donors remains the preferred approach worldwide. Hence, strategies that enhance islet graft survival and reduce the islet mass for transplantation show clinical relevance and are valuable.

Both CRISPR and nanoparticle are other tools for gene delivery. CRISPR system is usually applied in stem-cell derived beta cells in which gene editing is first performed in stem cells followed by the differentiation into beta cells. There is one study demonstrating the use of CRISPR in human islet (PMID 33893274) and there is less study using nanoparticle to deliver genes into islets. Further studies and investigation are warranted to demonstrate their efficiency and safety in human islets. In contrast, AAV is a well-established gene delivery tool adopted in both mouse and human islets (PMID 29304344, 15858610, 31355778, 25676679, 25338552, 16567506, 18560422, 30400037), while multiple clinical studies have provided promising evidence of AAV-mediated long-term expression of therapeutic proteins without causing substantial adverse effects (PMID 23474247, 25409372, 25938638). Since our study did not aim to develop new gene delivery tool, but to demonstrate the therapeutic effects of PAX6 in diabetes management and islet transplant outcome, hence we chose AAV, the more well-established system as the gene delivery approach. These points can be discussed in the revised version.

Additional Suggestions:

Figure 1:

• The data from in vivo overexpression would be strengthened if the authors show similar effects of overexpression of Pax6 in EndoC cells

A: Overexpression of PAX6 in EndoC cells and the measurement of cell survival and function will be included in the revised version.

• Explain rationale for why incretin hormones are used

A: It is because previous studies have shown that PAX6 controls the GLP-1R and GIPR expression levels in rodent beta cells (PMID 22403172, 28377501, 27941241) and incretin signaling is known to critically regulate beta cell function and survival (PMID 16517403). These points will be emphasized in the revised version.

o Is proliferation being used as a measure of beta cell vitality? Why? A bit confusing given apparent lack of beta cell proliferation in adults

A: We measure beta cell proliferation, cell death and insulin content, which are the indicators of beta cell survival and mass. The capability of cell proliferation in human beta cell is very limited. However, previous studies have shown that this can be induced (PMID 32051230, 30059217, 30581122).

o Insulin secretion data would strengthen the studies

A: Glucose-stimulated insulin secretion will be included in the revised version.

Figure 3:

• T1D modeling via STZ usually requires multiple low doses; as presented the authors are using high dose to ablate β cells. This is not really a T1D model (more a model of insulin deficiency), and to present it as such is questionable. A NOD mouse model with Pax6 overexpression would strengthen this claim.

A: Both multiple low dose and single high dose of STZ are widely used in different mouse strains in different studies (PMID 21478271, 16755002) in order to ablate beta cells.

It is true that the STZ mouse model is not a T1D model. T1D is caused by the autoimmune destruction of beta cells while the STZ model is just to mimic the severe beta cell loss seen in T1D. Since we are not investigating the immunomodulation, we focus on the direct effect of PAX6 gene delivery on beta cell regeneration, the STZ model serves our purpose and hence is adopted in this study.

• The increase in beta cell proliferation and reduction in death should result in an increase in beta cell mass, but these data are not presented.

A: Measurement of beta cell mass will be included in the revised version.

• Panel I quantifies Ins+ Ki67+ cells to measure beta cell proliferation; should panel J quantify cells that are both TUNEL+ and Ins+ to measure beta cell apoptosis?

A: Figure 3J will be modified accordingly in the revised version.

Figure 4:

• The striking differences observed in the overexpression (A-H) does not appear to be reflected by the small differences observed in proliferation and death (I-J); again, presentation of beta cell mass would be helpful.

A: Measurement of beta cell mass will be included in the revised version.

Figure 5:

• Glucose stimulated insulin secretion (GSIS) to show effect of overexpression would strengthen the study.

A: Results of GSIS on human islets will be included in the revised version.

Figure 7:

• Please clarify control group being used for RNA sequencing on the figure itself, and in Results section (listed as just Ctrl- are these AAV-Ctrl?)

A: The control group is the AAV-Ctrl. The figure label will be modified in the revised version.

• Panel A-D: p-value adjustment for multiple hypothesis testing?/FDR?

A: This is Benjamini-Hochberg adjusted p-value.

• Panel G: are the listed pathways significantly enriched/overrepresented? Suggest showing GO analysis, statistics

A: Yes, the listed pathways are the top KEGG pathways enriched by the 843 CHIP-OE overlapping genes.

• Any cell cycle genes that are altered?

A: Yes, there are some genes involved in cell cycle altered which will be included in the revised version.

• Please show PCA plot to show variance between samples

A: PCA plot will be included in the revised version.

• Discuss why only 20% of genes are similar between CHIP and OE; what are the other genes doing in relation to beta cells

A: Genes bound by PAX6 with their expression unaltered by PAX6 overexpression suggest that PAX6 binds to these genes' promoters but do not regulate their expression. It is recognized that chromatin immunoprecipitation (ChIP) reveals the binding of transcription factors to thousands of genomic locations in the vicinity of both active and inactive regions. A number of weakly bound sites or the sites bound at low occupancy detected this way actually fail to drive gene transcription (PMID 24888900, 18271625, 23236164).

On the other hand, genes altered by PAX6 overexpression but not bound by PAX6 suggest that PAX6 indirectly regulates the expression of these genes without direct binding. This can be explained by the regulation of the expression/ activity of other transcription factors or master regulators such as MAFA, PDX1, NKX6.1, CREB and Akt by PAX6. These transcription factors/ regulators in turn regulate the expression of a bundle of genes.

These explain why only a portion of genes is shared by the ChIP and OE results.

Referee #2 (Remarks for Author):

The manuscript by So et al describes how Pax6 is important for beta-cell biology proliferation and survival. They present data that show that Pax6 overexpression may help treat diabetes both in mice models mimicking type 1 or type 2 diabetes. Moreover, they present data showing that Pax6 overexpression in human islets improve their function when transplanted in diabetic mice. The results are interesting and suggest that Pax6 overexpression may be part of new therapeutic strategies, at least in islet graft. Yet, they are several concerns about controls and experiments that are not presented. Thus, serious modifications of the present manuscript are required to improve the quality of the manuscript.

A main issue on the use of the AAV to overexpress Pax6 is to show that it actually targets beta cells and no other tissues. Controls of Pax6 expression in different tissues need to be shown. In addition, Pax6 overexpression in control normoglycemic mice, not just in diabetic mic, needs to be shown. In control mice, does Pax6 overexpression lead to a better glucose homeostasis?

A: AAV transduction is observed in beta cells (insulin positive cells) as shown in figure S2B. AAV transduction is minimally detected in other tissues (liver, kidney, muscle, spleen). The results will be included in the revised version.

We did not include the Ctrl + AAV-PAX6 group in our first draft as demonstrating the therapeutic potential of AAV-PAX6 in diseased mice would be more meaningful and in real practice, none of the medications will be prescribed to normal subjects. We can provide the data on normal mice in the revised version.

Another main issue is the quantification of rare events. Proliferation and apoptosis rates were measured in alpha and beta cells in vivo, in STZ mice and Db mice. These events are rare, thus the positive cells (for example ki67+glucagon or TUNEL+insulin) are scarce. To obtain a precise measurement, it is required to count a minimum of these scarce events. For example, a bias can be easily made if a too low number of events is counted: 1 cells out of 1000 would suggest a rate of 0.1% while 2 cells out of 1000 would suggest a rate of 2% and a doubling of the rate with just one extra positive cell. The authors do not mention how many events where counted in each measurement and that is absolutely required.

A: For STZ mouse pancreas sections, we quantify more than 10 pancreas sections (around 50 islets) for each mouse, 6 mice were included for each group. For db/db mouse and human islet sections, we quantify around 5 sections (over 50 islets) for each batch of islets and 4-6 batches of islets were included for each group. For islet graft-bearing kidneys, around 5 sections were quantified for each mouse and 4-6 mice were included for each group. The number of mice/ batches of islets is indicated in the figure legend. The details will be added in the method section in the revised version.

On figure 1, it would be very logical to show Pax6 KD before showing the effects on the cells. It is a required control that have to be shown first to prove the efficiency of the KD.

A: The graphs will be re-organized in the revised version.

Again, figure 1 needs to be re-organized: quantifications of WB need to be called as panels. Analysis of pAkt needs to display whether basal pAkt levels (not just insulin-stimulated) are different when Pax6 is KD.

A: Comparison of basal pAkt between Ctrl and PAX6 KD groups will be displayed in the revised version.

Figure S1 may include the expression levels of more transcription factors, including Pax4, Tcfl2, hnf6 and hnf4alpha isl1 and neurod

A: The measurement will be included in the revised version.

The experience with HGPA needs to be more documented: what are the glucose levels normally required to grow the cells? What are the glucose levels for HGPA? Glucose-stimulated insulin secretion should be measured on endobetaH1 cells in the different conditions.

There is a need fora statistical analysis in between ctrl and HGPA cells. It is absolutely mandatory to describe properly the effect of HGPA. During HGPA, culture conditions may force cells to secrete more insulin thus the decreased ins content may reflect improved secretion. Therefore, insulin content must be related to GSIS.

A: Cells are maintained under normal condition (5.6 mmol/l glucose) or treated with HGPA (25 mmol/l D-glucose and 0.3 mmol/l palmitic acid) which has been stated in the "cell culture" section in materials and methods. Insulin content is significantly reduced under HGPA condition compared to normal condition as shown in figure 2C. Data on GSIS will be included in the revised version.

Similarly to figure 1, the panels of figure 2 need to be re-organized. The control of Pax6 overexpression need to be shown first before the panels on proliferation and apoptosis. Also, the condition ctrl+pax6 overexpression is interesting and needs to be shown on figures.

A: Overexpression of PAX6 in EndoC cells under normal condition and the measurement of cell survival and function will be included in the revised version.

Please comment on how calculations for ratios of pAkt were done. Is there a normalization to total akt and to GAPDH and to control condition ? Please explain.

A: As indicated in the quantification graph (figure 1E, right panel), pAkt is first normalized to total Akt. Then fold change is calculated by comparing all groups to the Ctrl+ vehicle group.

It seems that pa6 overexpression reverts the effects of HGPA. Is it also true with Pax4 or pdx1 overexpression ? The question about the specificity of action of pax6 is central.

A: PAX4 is a key regulator for beta cell development and maturation during embryogenesis. Although still detectable, PAX4 expression is low in adult beta cells (PMID 18234907). Acute overexpression of PAX4 has been found to protect beta cells against stress-induced apoptosis and stimulates proliferation. However, sustained PAX4 expression results in beta cell dedifferentiation (repression of MAFA and insulin), blunted glucose-stimulated insulin secretion and glucose intolerance (PMID 21521872). Besides, transcript levels of PAX4 are increased in human T2D islets (PMID 17989064), which also suggests that PAX4

upregulation triggers beta cell dedifferentiation in T2D and explains why PAX4 expression is normally suppressed in adult beta cells. These observations make PAX4 not a suitable therapeutic target.

It has been demonstrated that PAX6 regulates PDX1 expression in mouse beta cells (PMID 27941241) and our results show that PAX6 overexpression would enhance PDX1 expression in diabetic mouse and human islets (figure S4 and S5). In addition to PDX1, PAX6 also regulates the expression/ activity of other key regulators including MAFA and NKX6.1, suggesting PAX6 as a more comprehensive target.

A strong correlation between PAX6 activity/ levels and human diabetes is seen. While mechanistic studies are mainly done on mice, those studies suggest PAX6 critically regulates mouse beta cell function but its roles in human beta cells remain ambiguous. These also justify the study of PAX6 in human islets.

STZ mice are not a T1D like phenotype. STZ mice are a model of diabetes to a lack of insulin (after chemical destruction of beta cells) but without auto-immune attack. Please correct.

A: The sentence will be corrected accordingly.

The expression of pax 6 was measured on whole pancreas. Since stz kills beta cells, the number of beta cells is decreased in the stz+aav-ctrl group.thus number of pax6 expressing cells decreases, hence loss of pax6. where is the ctrl + pax6 aav group ?

A: Data on normal mice with AAV-PAX6 will be included in the revised version.

In STZ mice and in Db/db mice, AAVPax6 treatment may modify beta-cell mass. Assessment of beta-cell mass needs to be performed in these mice.

A: Measurement of beta cell mass will be included in the revised version.

A more precise description of the STZ model is needed. What is the dose of STZ ? Is it a one shot of STZ or many shots ? Is there a total or a partial destruction of beta cells ?

A: One single high dose of STZ is used in both C57BL/6J and NSG mice. For C57BL/6J (150mg/kg), mice with blood glucose levels exceeding 14 mmol/l for 2 consecutive times were used as recipients of AAV. Partial destruction of beta cells is required to allow AAV transduction in the remaining beta cells. For NSG (120mg/kg), mice with blood glucose levels exceeding 20 mmol/l for 2 consecutive times were used as recipients of islet transplant to mimic human context, as islet transplant is usually performed in patients with severe hyperglycemia. The details will be included in the revised version.

In STZ mice, pax6 is overexpressed in beta cells and stimulates proliferation but the stimulation of proliferation is not impressive but seems to be sufficient to restore beta cells and produce insulin. Please comment on this result.

A: Both stimulation of beta cell proliferation and inhibition of beta cell death account for the amelioration of hyperglycemia. Besides, relief on hyperglucagonemia also helps.

TUNEL levels are very high in all groups while apoptosis in beta cells is supposed to be a very low level. Also, pictures of IF for TUNEL suggest that apoptotic cells are not beta cells but other cells. How was the co staining for TUNEL and insulin quantified ?

A: TUNEL level is low in normal islets but high in diabetic subjects (fig. 3J, 4J, 5D). STZ targets beta cells and causes beta cell death and more apoptotic cells are detected in STZ mice. Co-staining is quantified by counting the TUNEL and insulin double positive cells and divided by the total number of insulin positive cells.

Was alpha cell mass measured in groups of mice with modified blood glucagon levels ?

A: Alpha cell mass was not quantified, the pancreatic alpha-to-beta cell ratio was quantified instead.

MafA IF should be nuclear but the picture show a weird staining in some cells. Please improve IF.

A: The image will be improved accordingly.

What is the phenotype of human normal islets + AAVPax6 ? Again, this group is missing and could provide interesting informations.

A: Figure 7, S6C to S6F demonstrates the effects of AAV-PAX6 on normal human islets with the post-transplant islet graft function and survival evaluated.

Line 262, the authors claim that "transplantation of T2D islets transduced with AAV-Ctrl (STZ/T2D-Ctrl) modestly normalized the blood glucose level". If glycose returns to a normal value, then it is called normalized, otherwise, it is decreased comparerd to control group. Modestly normalized is a non sense.

A: The sentence will be corrected accordingly.

Insulin contents are compared in between STZ/Ctrl-L and STZ/L+Pax6 but it should also be compared to STZ/Ctrl-H.

A: Comparison between STZ/Ctrl-H and STZ/Ctrl-L will be included in the revised version.

Referee #3 (Remarks for Author):

The manuscript entitled "Pax6 gene delivery preserves beta cells and improves islets transplantation efficacy" by Wing Yan So, et al., describes the role of modulating Pax6 expression in pancreatic islets to preserve/restore beta cell function in different models of T2D, including preclinical (Db/Db, STZ treated mice) and human control and T2D islets, engrafted in STZ mice. This is an important study unravelling that modulation of Pax6 expression (i.e. increasing its levels) improves beta cell function. This is a well controlled and designed study, using pre-clinical relevant models, where functional tests are associated to molecular characterization of Pax6 over expression. The in vivo preclinical studies are clearly demonstrating that maintaining Pax6 levels may contribute to improve beta cell function/dedifferentiation during T2D.

I have some comments concerning the study.

Concerning the AAV8 approach, the authors do not describe why they have used this serotype, and whether the AAVs infect both human and mouse pancreatic islets. Is the infection specific to pancreatic islets or are other tissues also affected after AAVs injection? How efficient is the transaction of AAVs in the middle of pancreatic islets?

A: AAV8 has been demonstrated to stably transduce the pancreas, including beta cell in islets. The tissue specificity would be greatly enhanced when combining with a tissue specific promoter. Insulin promoter is used in the present study and other studies for beta cell transduction (PMID 31355778, 25676679, 25338552, 16567506, 18560422, 30400037). AAV8 is also able to efficiently transduce human islets as shown in this study (figure 5E) and other studies (PMID 29304344, 15858610). Details will be added in the revised version.

AAV transduction is minimally detected in other tissues (liver, kidney, muscle, spleen). The results will be included in the revised version. Transduction in the middle of islets is observed as shown in figure S2B; while quantification of protein expression (figure S2A, 4K and 5E) also shows the significant increase in PAX6 expression.

It is interesting to observe that Pax6 over expression modulates proliferation and/or apoptosis, in response (or not) to Insulin or Glp1. In the RNA-seq/ChIP-seq integrated analysis, none of the proliferative nor apoptosis pathways were found to be regulated by Pax6, although this seems to be the major functional effect (and, consequently, subsequent insulin secretion effect). The authors should, at least, discuss this point.

A: The RNA-seq/ChIP-seq integrated analysis only displays the pathways/ genes that are directly regulated by PAX6 (genes bound by PAX6 and are also altered by PAX6 overexpression). However, PAX6 also regulates the expression/ activity of other transcription factors or master regulators such as MAFA, PDX1, NKX6.1, CREB and Akt. These transcription factors/ regulators in turn regulate the expression of a bundle of genes. These genes/ pathways are not directly regulated by PAX6 (no direct binding) and hence are not displayed in the ChIP/ OE overlapping result. This point can be discussed in the revised version.

12th Jun 2023

Dear Prof. Han,

Thank you for your e-mail asking us to reconsider our decision on your manuscript and for providing a detailed provisional point-by-point letter. I have now carefully read it and after discussion with my colleagues, we would like to invite you to submit your revised work to EMBO Molecular Medicine.

Please note that based on the initial reports and on the amount of work needed to address the referees' concerns, we cannot guarantee at this stage that the eventual outcome will be favorable. Addressing the reviewers' concerns in full will be necessary for further considering the manuscript in our journal, and a revised manuscript will once again be subject to review.

EMBO Molecular Medicine encourages a single round of revision only and therefore, acceptance or rejection of the manuscript will depend on the completeness of your responses included in the next, final version of the manuscript. For this reason, and to save you from any frustrations in the end, I would strongly advise against returning an incomplete revision. Should you find that the requested revisions are not feasible within the constraints outlined here and prefer, therefore, to submit your paper elsewhere, we would welcome a message to this effect.

Revised manuscripts should be submitted within three months of a request for revision; they will otherwise be treated as new submissions, except under exceptional circumstances in which a short extension is obtained from the editor.

When submitting your revised manuscript, please carefully review the instructions that follow below. We perform an initial quality control of all revised manuscripts before re-review; failure to include requested items will delay the evaluation of your revision.

We require:

1) A .docx formatted version of the manuscript text (including legends for main figures, EV figures and tables). Please make sure that the changes are highlighted to be clearly visible.

2) Individual production quality figure files as .eps, .tif, .jpg (one file per figure). For guidance, download the 'Figure Guide PDF' (https://www.embopress.org/page/journal/17574684/authorguide#figureformat).

3) At EMBO Press we ask authors to provide source data for the main figures. Our source data coordinator will contact you to discuss which figure panels we would need source data for and will also provide you with helpful tips on how to upload and organize the files.

4) A .docx formatted letter INCLUDING the reviewers' reports and your detailed point-by-point responses to their comments. As part of the EMBO Press transparent editorial process, the point-by-point response is part of the Review Process File (RPF), which will be published alongside your paper.

5) A complete author checklist, which you can download from our author guidelines (https://www.embopress.org/page/journal/17574684/authorguide#submissionofrevisions). Please insert information in the checklist that is also reflected in the manuscript. The completed author checklist will also be part of the RPF.

6) Please note that all corresponding authors are required to supply an ORCID ID for their name upon submission of a revised manuscript.

7) It is mandatory to include a 'Data Availability' section after the Materials and Methods. Before submitting your revision, primary datasets produced in this study need to be deposited in an appropriate public database, and the accession numbers and database listed under 'Data Availability'. Please remember to provide a reviewer password if the datasets are not yet public (see https://www.embopress.org/page/journal/17574684/authorguide#dataavailability).

In case you have no data that requires deposition in a public database, please state so in this section. Note that the Data Availability Section is restricted to new primary data that are part of this study.

8) For data quantification: please specify the name of the statistical test used to generate error bars and P values, the number (n) of independent experiments (specify technical or biological replicates) underlying each data point and the test used to calculate p-values in each figure legend. The figure legends should contain a basic description of n, P and the test applied. Graphs must include a description of the bars and the error bars (s.d., s.e.m.). Please provide exact p values.

9) Our journal encourages inclusion of *data citations in the reference list* to directly cite datasets that were re-used and obtained from public databases. Data citations in the article text are distinct from normal bibliographical citations and should

directly link to the database records from which the data can be accessed. In the main text, data citations are formatted as follows: "Data ref: Smith et al, 2001" or "Data ref: NCBI Sequence Read Archive PRJNA342805, 2017". In the Reference list, data citations must be labeled with "[DATASET]". A data reference must provide the database name, accession number/identifiers and a resolvable link to the landing page from which the data can be accessed at the end of the reference. Further instructions are available at .

10) We replaced Supplementary Information with Expanded View (EV) Figures and Tables that are collapsible/expandable online. A maximum of 5 EV Figures can be typeset. EV Figures should be cited as 'Figure EV1, Figure EV2" etc... in the text and their respective legends should be included in the main text after the legends of regular figures.
- For the figures that you do NOT wish to display as Expanded View figures, they should be bundled together with their legends in a single PDF file called *Appendix*, which should start with a short Table of Content. Appendix figures should be referred to in the main text as: "Appendix Figure S1, Appendix Figure S2" etc.
- Additional Tables/Datasets should be labeled and referred to as Table EV1, Dataset EV1, etc. Legends have to be provided in a separate tab in case of .xls files. Alternatively, the legend can be supplied as a separate text file (README) and zipped together with the Table/Dataset file.
See detailed instructions here:

11) The paper explained: EMBO Molecular Medicine articles are accompanied by a summary of the articles to emphasize the major findings in the paper and their medical implications for the non-specialist reader. Please provide a draft summary of your article highlighting
- the medical issue you are addressing,
- the results obtained and
- their clinical impact.
This may be edited to ensure that readers understand the significance and context of the research. Please refer to any of our published articles for an example.

12) For more information: There is space at the end of each article to list relevant web links for further consultation by our readers. Could you identify some relevant ones and provide such information as well? Some examples are patient associations, relevant databases, OMIM/proteins/genes links, author's websites, etc...

13) Author contributions: CRediT has replaced the traditional author contributions section because it offers a systematic machine readable author contributions format that allows for more effective research assessment. Please remove the Authors Contributions from the manuscript and use the free text boxes beneath each contributing author's name in our system to add specific details on the author's contribution. More information is available in our guide to authors.

14) Disclosure statement and competing interests: We updated our journal's competing interests policy in January 2022 and request authors to consider both actual and perceived competing interests. Please review the policy https://www.embopress.org/competing-interests and update your competing interests if necessary.

15) Every published paper now includes a 'Synopsis' to further enhance discoverability. Synopses are displayed on the journal webpage and are freely accessible to all readers. They include a short stand first (maximum of 300 characters, including space) as well as 2-5 one-sentences bullet points that summarizes the paper. Please write the bullet points to summarize the key NEW findings. They should be designed to be complementary to the abstract - i.e. not repeat the same text. We encourage inclusion of key acronyms and quantitative information (maximum of 30 words / bullet point). Please use the passive voice. Please attach these in a separate file or send them by email, we will incorporate them accordingly.

Please also suggest a striking image or visual abstract to illustrate your article as a PNG file 550 px wide x 300-600 px high.

16) As part of the EMBO Publications transparent editorial process initiative (see our Editorial at http://embomolmed.embopress.org/content/2/9/329), EMBO Molecular Medicine will publish online a Review Process File (RPF) to accompany accepted manuscripts.
In the event of acceptance, this file will be published in conjunction with your paper and will include the anonymous referee reports, your point-by-point response and all pertinent correspondence relating to the manuscript. Let us know whether you agree with the publication of the RPF and as here, if you want to remove or not any figures from it prior to publication.
Please note that the Authors checklist will be published at the end of the RPF.

EMBO Molecular Medicine has a "scooping protection" policy, whereby similar findings that are published by others during review or revision are not a criterion for rejection. Should you decide to submit a revised version, I do ask that you get in touch after three months if you have not completed it, to update us on the status.

I look forward to receiving your revised manuscript.

Yours sincerely,

Lise Roth

Lise Roth, PhD
Senior Editor
EMBO Molecular Medicine

Dear Dr. Roth,
Thank you for your decision letter on 12 June 2023. We appreciate the insightful comments from all 3 reviewers and have performed a number of new experiments based on their suggestions to strengthen the study and address their concerns. We have also made changes to the text in the current revised version as highlighted for your kind consideration of publication in *EMBO Molecular Medicine*.
Please find below our point-to-point response to the reviewers' comments.

Referee #1 (Comments on Novelty/Model System for Author):

The novelty of the studies is questionable. There is extensive literature on Pax6 and its role in beta cell formation and function, so I'm not sure how much this study adds. Delivery of a transcription factor as a therapeutic approach has been mentioned in the literature for years, but few approaches if any have materialized. Again, the novelty there is also marginal.

A: The referenced studies on PAX6 are mainly in mouse beta cells using mutant mice (PMID 15110714, 9224716, 26658466, 27941241, 23326594, 28377501). These studies show that PAX6 is physiologically essential for normal mouse islet development and function using a loss-of-function approach. It is well documented that species differences exist between human and rodent islets in terms of metabolism, cytoarchitecture and functional implications (PMID 21454710, 28422162, 16461897), which may influence the translatability of mouse studies to the human context. In contrast, we use human islets in our study to demonstrate the human relevance besides the experiments with mice. Moreover, it is generally recognized that the capability of adult human beta cell replication or regeneration is very limited when compared to rodent beta cells (PMID 22751699), and there is currently no clinical therapy proven to stimulate beta cell proliferation or preserve beta cell mass once beta cell failure has started (PMID 27189025). Hence, strategies that can preserve beta cells, even in a modest manner, are valuable. With the use of cell line, mouse models and human islets, our results highlight previously unappreciated roles for PAX6 in maintaining human islet survival and the potential contribution to diabetes management. While it is important to understand the physiological role of the critical beta cell regulators, the ultimate goal is to develop therapeutic strategies by targeting those regulators, which is not addressed by any of the aforementioned studies. In our study, we apply various *in vitro* and *in vivo* models to demonstrate the therapeutic effects of AAV-mediated PAX6 gene delivery on beta cell preservation and diabetes management. In particular, we show that PAX6 gene delivery enhances both normal and T2D human islet potency, and thus might overcome two of the major unresolved barriers of clinical islet transplantation: post-transplant islet graft loss and scarcity of healthy islets from donors. By investigating scientific basis to support targeting PAX6 to overcome beta cell failure, our study hence provides the first evidence for the potential clinical relevance of PAX6 in diabetes management.

Referee #1 (Remarks for Author):

Summary: In this study, the authors argue that Pax6 is a critical regulator of beta cell proliferation and cell death based on data using lentivirus and insulin promoted AAV to

either knockdown or overexpress Pax6. Through numerous in vitro and in vivo model systems focusing on islets, the authors show that Pax6 ameliorates T1D (via single, high dose STZ although not adequately explained in methods) and T2D (via db/db or high glucose media, HGPA) disease systems. Moreover, they transplant T2D human islets treated with AAV-Pax6 into STZ treated NSG mice to show functional glycemic control via increased insulin secretion. Bulk transcriptional analysis on control islets (unclear whether KO or WT Pax6 islets) versus AAV-Pax6 overexpressed islets show an increase in genes related to beta cell identity and pancreatic secretion. Islet transplantations in STZ treated NSG mice using islets of lower mass that have been exposed with AAV-Pax6 show similar improvement to the higher mass islets. Overall, the multiple experiments highlight that overexpression of Pax6 results in improved islet function.

A: A single high dose of STZ was intraperitoneally injected into C57BL/6J (150 mg/kg) or NSG (120 mg/kg) mice to render hyperglycemia. The details have been added in the Materials and Methods section (lines 571-573).

Impact: Marginal, owing to the myriad other papers documenting the well-known role of Pax6. Moreover, this study does not account for AAV or lentiviral transfection efficiency issues that have been seen with other pancreatic genes. There may be marginal increase in knowledge depicting Pax6 as beneficial for low mass islets.

A: With all due respect, we disagree on the reviewer's suggestion that our study provides only marginal increase in knowledge. The novelty and the significance of the current study have been elaborated above in response to the first question. Regarding the transfection efficiency, our results have shown that the lentivirus efficiently causes PAX6 knockdown in the EndoC-bH1 cell line (Appendix fig. S1A). Moreover, we also show that our lentivirus and AAV approaches efficiently deliver PAX6 into cell line (Appendix fig. S1C and S1D), mouse beta cells/ islets (Appendix fig. S2A, S2B, S3A, S3B and fig. 4K) and human islets (fig. 5E) as shown by the significant increases in PAX6 protein levels.

Strengths: This paper has multiple in vitro and in vivo methods of testing knockout and overexpression capabilities for AAV mediated constructs. The authors effectively utilize these repetitive techniques throughout the paper to support their hypothesis. The paper is well written with easy flow and the figures as well as figure legends are easy to follow. For the most part, they cite papers that have addressed Pax6 within the field to a reasonable extent.

Criticisms: Although this paper has copious data, the implications are not entirely novel. The introduction is fairly generic, focusing more on T1D and T2D disease pathogenesis, rather than the background that led to the study of Pax6 vs. other transcription factors (e.g. Pax4). Moreover, the discussion does not include other potential therapeutic methods under development such as stem cell derived islet organoids, CRISPR corrected, or nanoparticle

delivery systems. The authors do not discuss how Pax6, itself, enables improved beta cell function (as opposed to increased beta cell replication-which itself may not improve function).

A: PAX4 is a key regulator for beta cell development and maturation during embryogenesis. Although still detectable, PAX4 expression is low in adult beta cells (PMID 18234907). Acute overexpression of PAX4 has been found to protect beta cells against stress-induced apoptosis and stimulates proliferation. However, sustained PAX4 expression results in beta cell dedifferentiation (repression of MAFA and insulin), blunted glucose-stimulated insulin secretion and glucose intolerance (PMID 21521872). Besides, transcript levels of PAX4 are increased in human T2D islets (PMID 17989064), which also suggests that PAX4 upregulation triggers beta cell dedifferentiation in T2D and explains why PAX4 expression is normally suppressed in adult beta cells. These observations strongly argue against PAX4 as a suitable therapeutic target.

PAX6 is found to be down-regulated in human T2D islets (PMID 22768844, 34193609) while PAX6 gene mutations are associated with diminished insulin secretion and glycemic perturbations in human (PMID 30151985, 15842522, 11756345). These observations suggest a strong correlation between PAX6 activity / levels and human diabetes. While mechanistic studies are mainly done on mice, those studies suggest PAX6 critically regulates mouse beta cell function but its roles in human beta cells remain ambiguous. These justify the study of PAX6 in human islets. The background of PAX4 and PAX6 has been included in the Introduction section (lines 99-112).

Induced pluripotent stem cells (iPSC) are not yet suitable for large-scale production of patient-specific iPSC-beta cells for transplantation since individuals require a large number of individual pancreatic islets. In addition, unlike whole pancreas or islet transplantation, iPSC-derived beta cells do not replace the lost function of alpha cells. Deficiency in glucagon production precipitates hypoglycemia unawareness. Until these challenges are addressed, transplantation of islets from cadaveric donors remains the preferred approach worldwide. Hence, it is clinically relevant to develop strategies that enhance islet graft survival and reduce the islet mass for transplantation. These points have been discussed in the Discussion section (lines 517-525).

CRISPR and nanoparticle are additional tools for gene delivery. The CRISPR system is usually applied in stem-cell derived beta cells. There is only one study demonstrating the use of CRISPR in human islets, which involves extensive manipulations including islet dispersion and reaggregation into pseudoislets (PMID 33893274). There are also limited number of studies using nanoparticles to deliver genes into islets (PMID 36706177, 26530912). Further investigation is warranted to demonstrate their efficiency and effects on islet physiology of these approaches. In contrast, AAV is the leading platform for gene delivery for the treatment of various human diseases as it provides long-term gene modification without causing substantial adverse effects (PMID 30710128). AAV is also widely adopted in both mouse and human islets (PMID 29304344, 15858610, 31355778, 25676679, 25338552, 16567506, 18560422, 30400037). Since our study was not aimed at developing a new gene delivery tool, but demonstrating the therapeutic effects of PAX6

delivery in diabetes management and islet transplant outcome, hence we chose AAV, the well-established system as the gene delivery approach. These points have been discussed in the Discussion section (lines 415-427).

The effects of PAX6 manipulations on glucose-stimulated insulin secretion (GSIS) have been examined as suggested and shown in fig. 1C, 2C, S1G, S6A and S8B. Our study demonstrated PAX6 as a critical regulator of beta cell function, survival and identity, which orchestrate to enhance the potency of human beta cells and islets.

Additional Suggestions:

Figure 1:

• The data from in vivo overexpression would be strengthened if the authors show similar effects of overexpression of Pax6 in EndoC cells

A: Overexpression of PAX6 in EndoC-bH1 cells has been performed as suggested (Appendix fig. S1D to S1L and lines 180-185). It was found that PAX6 overexpression (Appendix fig. S1D) increased cell proliferation stimulated by insulin, exendin-4, GIP and IGF1 (Appendix fig. S1E). PAX6 overexpression also inhibited cell apoptosis (Appendix fig. S1F), increased GSIS (Appendix fig. S1G) and insulin content (Appendix fig. S1H). Moreover, PAX6 overexpression also enhanced insulin and incretin signalings (Appendix fig. S1I to S1L).

• Explain rationale for why incretin hormones are used

A: Hormones or factors that are well-known to stimulate beta cell proliferation including insulin, exendin-4 (GLP-1 analog), glucose-dependent insulinotropic polypeptide (GIP) and insulin-like growth factor 1 (IGF1) were used to demonstrate the effects of PAX6 manipulations on beta cell proliferation. Besides, previous ChIP-sequencing data reveal that PAX6 binds to genes related to the insulin signaling (PMID 27941241) and on the other hand PAX6 was found to control the GLP-1R and GIPR expression levels in rodent beta cells (PMID 22403172, 28377501, 27941241). Both insulin and incretin signalings are known to critically regulate beta cell function and survival (PMID 18481923, 16517403) but whether PAX6 modulates these signaling events in human beta cells is yet to be confirmed. These points have been included in the Result and Discussion sections (lines 143-146 and lines 445-450).

o Is proliferation being used as a measure of beta cell vitality? Why? A bit confusing given apparent lack of beta cell proliferation in adults

A: We measure beta cell proliferation, cell death and insulin content, which are the indicators of beta cell survival and mass. EndoC-bH1 cell line is an immortalized cell line capable of

proliferation as shown in our data and previous publication (PMID 21865645). On the other hand, the capability of cell proliferation in primary human beta cell is very limited. However, previous studies have shown that this can be induced (PMID 32051230, 30059217, 30581122).

o Insulin secretion data would strengthen the studies

A: The effects of PAX6 manipulations on GSIS in EndoC-bH1 cells have been examined as suggested. As shown in fig. 1C, PAX6 knockdown significantly reduces GSIS while in fig. 2C and S1G, PAX6 overexpression enhances GSIS in both normal and HGPA conditions.

Figure 3:

• T1D modeling via STZ usually requires multiple low doses; as presented the authors are using high dose to ablate β cells. This is not really a T1D model (more a model of insulin deficiency), and to present it as such is questionable. A NOD mouse model with Pax6 overexpression would strengthen this claim.

A: Both multiple low dose and single high dose STZ are widely used in different mouse strains in different studies (PMID 21478271, 16755002) in order to ablate beta cells.

We agree that the STZ mouse model is not ideal as a T1D model. T1D is caused by the autoimmune destruction of beta cells while the STZ model is just to mimic the severe beta cell loss seen in T1D. Since we are not investigating the immunomodulation, we focus on the direct effect of PAX6 gene delivery on beta cell regeneration, the beta cell deficit feature in the STZ model serves our purpose and hence is adopted in this study.

• The increase in beta cell proliferation and reduction in death should result in an increase in beta cell mass, but these data are not presented.

A: Measurement of beta cell mass in STZ mice has been performed as suggested and shown in Appendix fig. S2D. It was found that beta cell mass was significantly reduced by STZ, but increased by PAX6 delivery.

• Panel I quantifies Ins+ Ki67+ cells to measure beta cell proliferation; should panel J quantify cells that are both TUNEL+ and Ins+ to measure beta cell apoptosis?

A: Figure 3J has been modified accordingly.

Figure 4:

• The striking differences observed in the overexpression (A-H) does not appear to be reflected by the small differences observed in proliferation and death (I-J); again, presentation of beta cell mass would be helpful.

A: Measurement of beta cell mass in *db/db* mice has been performed as suggested and shown in Appendix fig. S4A. It was found that PAX6 gene delivery increases beta cell mass in *db/db* mice. On top of promoting beta cell survival, our results demonstrate that PAX6 gene delivery also increases insulin content and insulin secretion, maintains beta cell identity while causing a complementary reduction in glucagon levels. These modulations orchestrate to relieve glycemic perturbation.

Figure 5:

• Glucose stimulated insulin secretion (GSIS) to show effect of overexpression would strengthen the study.

A: GSIS in human islets has been performed as suggested. As shown in Appendix fig. S6A and S8B, AAV-PAX6 transduction promotes GSIS in both normal and T2D human islets.

Figure 7:

• Please clarify control group being used for RNA sequencing on the figure itself, and in Results section (listed as just Ctrl- are these AAV-Ctrl?)

A: The control group is the AAV-Ctrl. The figure labels and Result section have been modified accordingly (lines 343-345).

• Panel A-D: p-value adjustment for multiple hypothesis testing?/FDR?

A: Panel A is Benjamini-Hochberg adjusted p-value while panels B-D are FDR adjusted p-values. These are stated in the figure legend (lines 1125 and 1127).

• Panel G: are the listed pathways significantly enriched/overrepresented? Suggest showing GO analysis, statistics

A: Yes, the listed pathways are the top and significantly enriched KEGG pathways enriched by the 843 CHIP-OE overlapping genes adjusted p-value < 0.05; cutoff $\log_2 FC \geq 0.5$). This is stated in the figure legend (lines 1129-1132). The GO pathways were not included because there are many redundant terms, but relevant pathways such as hormone/insulin secretion or

transport, glucose homeostasis, positive regulation of MAPK cascade, are consistently overrepresented.

• Any cell cycle genes that are altered?

A: Yes, there is a list of genes involved in the regulation of DNA replication and cell proliferation upregulated in PAX6-overexpressing islets, which has been included in Appendix fig. S8E.

• Please show PCA plot to show variance between samples

A: The PCA plot has been included in Appendix fig. S8D as suggested.

• Discuss why only 20% of genes are similar between CHIP and OE; what are the other genes doing in relation to beta cells

A: That expression of genes bound by PAX6 was unaltered by PAX6 overexpression suggests that PAX6 binds to these genes' promoters but do not regulate their expression. It is recognized that chromatin immunoprecipitation (ChIP) reveals the binding of transcription factors to thousands of genomic locations in the vicinity of both active and inactive regions. A number of weakly bound sites or the sites bound at low occupancy detected this way actually fail to drive gene transcription (PMID 24888900, 18271625, 23236164).

On the other hand, that expression of genes that were not bound by PAX6 was altered by PAX6 overexpression suggests that PAX6 indirectly regulates the expression of these genes. This can be explained by the regulation of the expression / activity of other transcription factors or master regulators such as MAFA, PDX1, NKX6.1, CREB and Akt by PAX6. These factors in turn regulate the expression of a bundle of effector genes. These points have been highlighted in the Result section (lines 364-373).

Referee #2 (Remarks for Author):

The manuscript by So et al describes how Pax6 is important for beta-cell biology proliferation and survival. They present data that show that Pax6 overexpression may help treat diabetes both in mice models mimicking type 1 or type 2 diabetes. Moreover, they present data showing that Pax6 overexpression in human islets improve their function when transplanted in diabetic mice. The results are interesting and suggest that Pax6 overexpression may be part of new therapeutic strategies, at least in islet graft. Yet, they are

several concerns about controls and experiments that are not presented. Thus, serious modifications of the present manuscript are required to improve the quality of the manuscript.

A main issue on the use of the AAV to overexpress Pax6 is to show that it actually targets beta cells and no other tissues. Controls of Pax6 expression in different tissues need to be shown. In addition, Pax6 overexpression in control normoglycemic mice, not just in diabetic mic, needs to be shown. In control mice, does Pax6 overexpression lead to a better glucose homeostasis?

A: AAV-mediated exogenous PAX6 expression is observed in beta cells (insulin positive cells) as shown in Appendix fig. S2B and S3B, while exogenous PAX6 is minimally detected in other tissues (liver, kidney, muscle, spleen) as shown in Appendix fig. S2C.

The effects of AAV-PAX6 in normal mice have been investigated and included in Appendix fig. S3 and the Results section (lines 222-235). Unlike the marked effects in STZ mice, PAX6 gene delivery in normal mouse beta cells (Appendix fig. S3A and S3B) resulted in a mild reduction in blood glucose (Appendix fig. S3C) and slight improvement in glucose tolerance (Appendix fig. S3D and S3E). Serum insulin level and pancreatic insulin content were modestly increased by AAV-PAX6 administration (Appendix fig. S3F and S3G) while serum and pancreatic glucagon levels were not significantly altered (Appendix fig. S3H and S3I). Immunofluorescence staining showed that the islet alpha-to-beta cell ratio was not altered by PAX6 overexpression (Appendix fig. S3J). Moreover, the anti-apoptotic effect of AAV-PAX6 was not evident in normal mouse islets as both AAV-Ctrl- and AAV-PAX6-treated mice displayed a low amount of TUNEL-positive apoptotic cells in islets (Appendix fig. S3K). Notably, similar to the effects in STZ-treated mice, AAV-PAX6 increased beta cell proliferation (Appendix fig. S3L) and beta cell mass (Appendix fig. S3M) in normal mice whereas alpha cell mass was not significantly altered (Appendix fig. S3M). Taken together, AAV-PAX6 stimulates beta cell proliferation but improves glucose homeostasis only mildly in normal mice.

Another main issue is the quantification of rare events. Proliferation and apoptosis rates were measured in alpha and beta cells in vivo, in STZ mice and Db mice. These events are rare, thus the positive cells (for example ki67+glucagon or TUNEL+insulin) are scarce. To obtain a precise measurement, it is required to count a minimum of these scarce events. For example, a bias can be easily made if a too low number of events is counted: 1 cells out of 1000 would suggest a rate of 0.1% while 2 cells out of 1000 would suggest a rate of 2% and a doubling of the rate with just one extra positive cell. The authors do not mention how many events where counted in each measurement and that is absolutely required.

A: For normal and STZ-treated mouse pancreas sections, more than 10 pancreas sections containing over 50 islets were quantified for each mouse and 5 to 6 mice were included for each group. For *db/db* mouse and human islet sections, more than 5 sections containing over 50 islets were quantified for each batch of islets and 5 to 6 batches of islets were included for each group. More than 4000 beta cells per mouse or batch of islets were counted. For islet

graft-bearing kidney sections, more than 5 sections were quantified for each mouse and 4 to 6 mice were included for each group. The number of mice or batches of islets is indicated in the figure legend. The details have been added in the Materials and Methods section (lines 683-690).

On figure 1, it would be very logical to show Pax6 KD before showing the effects on the cells. It is a required control that have to be shown first to prove the efficiency of the KD.

A: Figure 1 has been re-organized with the quantification of PAX6 protein level shown first (Appendix fig. S1A).

Again, figure 1 needs to be re-organized: quantifications of WB need to be called as panels. Analysis of pAkt needs to display whether basal pAkt levels (not just insulin-stimulated) are different when Pax6 is KD.

A: Statistical comparison of basal pAkt between Ctrl and PAX6 KD groups has been displayed in the graphs (fig. 1F to 1H).

Figure S1 may include the expression levels of more transcription factors, including Pax4, Tcfl2, hnf6 and hnf4alpha isl1 and neurod

A: The measurement has been included as suggested (Appendix fig. S1B). Among those suggested transcription factors, only gene expression of *PAX4* and *NEUROD1* is reduced in cells with PAX6 KD.

The experience with HGPA needs to be more documented: what are the glucose levels normally required to grow the cells? What are the glucose levels for HGPA? Glucose-stimulated insulin secretion should be measured on endobetaH1 cells in the different conditions.

There is a need fora statistical analysis in between ctrl and HGPA cells. It is absolutely mandatory to describe properly the effect of HGPA. During HGPA, culture conditions may force cells to secrete more insulin thus the decreased ins content may reflect improved secretion. Therefore, insulin content must be related to GSIS.

A: The EndoC-bH1 cells are normally maintained in medium containing 5.6 mmol/l glucose, according to the standard protocol (PMID 21865645). HGPA medium contains 25 mmol/l D-glucose and 0.3 mmol/l palmitic acid. These are stated in the Materials and Methods section (lines 551-554).

GSIS in EndoC-bH1 cells under HGPA condition with PAX6 overexpression has been included as suggested (fig. 2C) and it was found that HGPA significantly suppressed GSIS but PAX6 overexpression could rescue the impaired GSIS to some extent.

Statistical comparison between Ctrl and HGPA has been displayed in the relevant graphs (fig. 2C and 2D).

It is recognized that only a few percent of insulin within a beta cell is released during a 1 hour glucose stimulation (PMID 17919179, 21865645), so the subtle reduction in insulin content caused by insulin secretion is not evident and might not be reflected. Conversely, it has been revealed that insulin content positively correlated with the pulse mass of insulin secretion and first and second-phase insulin secretory response in human islets (PMID 12574208, 14764798, 12865318) while reduction of insulin content in T2D patients (PMID3538738, 28887444, 21104225) associates with a concomitant decline in insulin secretion (PMID 10950818, 21104225). In line with these observations, our results demonstrated that HGPA significantly reduced insulin secretion and insulin content while PAX6 overexpression reversed these (fig. 2C and 2D).

Similarly to figure 1, the panels of figure 2 need to be re-organized. The control of Pax6 overexpression need to be shown first before the panels on proliferation and apoptosis. Also, the condition ctrl+pax6 overexpression is interesting and needs to be shown on figures.

A: Figure 2 has been re-organized with the quantification of PAX6 protein level shown first (Appendix fig. S1C).

Overexpression of PAX6 in EndoC-bH1 cells under normal condition has been performed as suggested (Appendix fig. S1D to S1L and lines 180-185). Similar to the effects under HGPA condition, PAX6 overexpression (Appendix fig. S1D) increased cell proliferation stimulated by insulin, exendin-4, GIP and IGF1 (Appendix fig. S1E). On top of that, PAX6 overexpression inhibited cell apoptosis (Appendix fig. S1F), increased GSIS (Appendix fig. S1G) and insulin content (Appendix fig. S1H). On top of that, PAX6 overexpression also enhanced insulin and incretin signalings (Appendix fig. S1I to S1L).

Please comment on how calculations for ratios of pAkt were done. Is there a normalization to total akt and to GAPDH and to control condition ? Please explain.

A: As indicated in the quantification panel (fig. 1F and 2F, right panels), pAkt is normalized to total Akt and hence pAkt/ Akt is displayed. Then the pAkt/Akt ratio of all groups is compared to the pAkt/Akt ratio of the Ctrl + vehicle group and thus the pAkt/Akt ratio of the Ctrl + vehicle group is set as 1.

It seems that pa6 overexpression reverts the effects of HGPA. Is it also true with Pax4 or pdx1 overexpression ? The question about the specificity of action of pax6 is central.

A: PAX4 is a key regulator for beta cell development and maturation during embryogenesis. Although still detectable, PAX4 expression is low in adult beta cells (PMID 18234907). Acute overexpression of PAX4 has been found to protect beta cells against stress-induced apoptosis and stimulates proliferation. However, sustained PAX4 expression results in beta cell dedifferentiation (repression of MAFA and insulin), blunted glucose-stimulated insulin secretion and glucose intolerance (PMID 21521872). Besides, transcript levels of PAX4 are increased in human T2D islets (PMID 17989064), which also suggests that PAX4 upregulation triggers beta cell dedifferentiation in T2D and explains why PAX4 expression is normally suppressed in adult beta cells. These observations argue against PAX4 as a suitable therapeutic target.

PAX6 is found to be down-regulated in human T2D islets (PMID 22768844, 34193609) while PAX6 gene mutations are associated with diminished insulin secretion and glycemic perturbations in human (PMID 30151985, 15842522, 11756345). These observations suggest a strong correlation between PAX6 activity / levels and human diabetes. While mechanistic studies are mainly done on mice, those studies suggest PAX6 critically regulates mouse beta cell function but its roles in human beta cells remain ambiguous. These justify the study of PAX6 in human islets. The background of PAX4 and PAX6 has been included in the Introduction section (lines 99-112).

It has been demonstrated that PAX6 regulates PDX1 expression in mouse beta cells (PMID 27941241) and our results show that PAX6 overexpression would enhance PDX1 expression in diabetic mouse and human islets (figure S5A, S5B, S6B and S6C). In addition to PDX1, PAX6 also regulates the expression / activity of other key regulators including MAFA and NKX6.1, suggesting PAX6 as a more suitable target.

STZ mice are not a T1D like phenotype. STZ mice are a model of diabetes to a lack of insulin (after chemical destruction of beta cells) but without auto-immune attack. Please correct.

A: The sentence has been corrected accordingly (lines 190-192).

The expression of pax 6 was measured on whole pancreas. Since stz kills beta cells, the number of beta cells is decreased in the stz+aav-ctrl group.thus number of pax6 expressing cells decreases, hence loss of pax6. where is the ctrl + pax6 aav group ?

A: The effects of AAV-PAX6 in normal mice have been investigated and included in Appendix fig. S3 and lines 222-235.

In STZ mice and in Db/db mice, AAVPax6 treatment may modify beta-cell mass. Assessment of beta-cell mass needs to be performed in these mice.

A: Measurement of beta cell mass in STZ mice and *db/db* has been included as suggested and shown in Appendix fig. S2D and S4A. Results show that AAV-PAX6 increased beta cell mass in both STZ-treated and *db/db* mice.

A more precise description of the STZ model is needed. What is the dose of STZ ? Is it a one shot of STZ or many shots ? Is there a total or a partial destruction of beta cells ?

A: One single high dose of STZ is used in both C57BL/6J and NSG mice. For C57BL/6J mice (150 mg/kg), mice with blood glucose levels exceeding 14 mmol/l for 2 consecutive times were used as recipients of AAV. Partial destruction of beta cells is required to allow AAV transduction in the remaining beta cells. For NSG (120 mg/kg), mice with blood glucose levels exceeding 20 mmol/l for 2 consecutive times were used as recipients of islet transplant to mimic human context, as islet transplant is usually performed in patients with severe hyperglycemia. These details have been included in the Materials and Methods section (lines 571-573, 578-579, 633-634).

In STZ mice, pax6 is overexpressed in beta cells and stimulates proliferation but the stimulation of proliferation is not impressive but seems to be sufficient to restore beta cells and produce insulin. Please comment on this result.

A: Apart from stimulation of beta cell proliferation, inhibition of beta cell death and increase in insulin content account for the increase in insulin levels and amelioration of hyperglycemia. Besides, relief on hyperglucagonemia also helps relieve glycemic disturbance.

TUNEL levels are very high in all groups while apoptosis in beta cells is supposed to be a very low level. Also, pictures of IF for TUNEL suggest that apoptotic cells are not beta cells but other cells. How was the co staining for TUNEL and insulin quantified ?

A: TUNEL level is low in normal islets but high in diabetic subjects (fig. 3J, 4J, 5D). Co-staining is quantified by dividing the TUNEL and insulin double positive cell number by the total insulin positive cell number.

Was alpha cell mass measured in groups of mice with modified blood glucagon levels ?

A: Alpha cell mass has been quantified. For STZ-treated mice, STZ caused a dramatic increase in alpha cell mass which was reduced by PAX6 overexpression (Appendix fig. S2G).

On the other hand, *db/db* mice also exhibited increased alpha cell mass which was decreased by PAX6 replenishment (Appendix fig. S4B).

MafA IF should be nuclear but the picture show a weird staining in some cells. Please improve IF.

A: The images have been improved accordingly (Appendix fig. S5B and S6C).

What is the phenotype of human normal islets + AAVPax6 ? Again, this group is missing and could provide interesting informations.

A: Figures 7 and S8 demonstrate the effects of AAV-PAX6 on normal human islets. Our results showed that PAX6 overexpression in normal islets stimulated beta cell proliferation, increased GSIS and insulin content *ex vivo* (Appendix fig. S8A to S8C). RNA sequencing reveals down-regulation of genes involved in T1D and inflammation and up-regulation of genes involved in MAPK signaling (fig. 7A to 7C) in PAX6-overexpressing islets. Besides, a number of genes involved in the regulation of DNA replication and cell proliferation were up-regulated (Appendix fig. S8E) while a list of disallowed genes was found to be down-regulated in PAX6-overexpressing islets (fig. 7F), these contributed to the enhanced potency of islets which in turn enabled a reduction in transplanted islet mass (fig. 7H to 7M, Appendix fig. S8F and S8G).

Line 262, the authors claim that "transplantation of T2D islets transduced with AAV-Ctrl (STZ/T2D-Ctrl) modestly normalized the blood glucose level". If glycose returns to a normal value, then it is called normalized, otherwise, it is decreased comparerd to control group. Modestly normalized is a non sense.

A: The sentence has been corrected accordingly (lines 310-311).

Insulin contents are compared in between STZ/Ctrl-L and STZ/L+Pax6 but it should also be compared to STZ/Ctrl-H.

A: Comparison of insulin levels between STZ/Ctrl-H and STZ/Ctrl-L has been included (fig. 7K and 7L). Lower transplanted islet mass (STZ/Ctrl-L) rendered lower serum human insulin graft human insulin levels compared to higher transplanted islet mass (STZ/Ctrl-H), which were increased by *PAX6* gene delivery (STZ/PAX6-L).

Referee #3 (Remarks for Author):

The manuscript entitled "Pax6 gene delivery preserves beta cells and improves islets transplantation efficacy" by Wing Yan So, et al., describes the role of modulating Pax6 expression in pancreatic islets to preserve/restore beta cell function in different models of T2D, including preclinical (Db/Db, STZ treated mice) and human control and T2D islets, engrafted in STZ mice. This is an important study unravelling that modulation of Pax6 expression (i.e. increasing its levels) improves beta cell function. This is a well controlled and designed study, using pre-clinical relevant models, where functional tests are associated to molecular characterization of Pax6 over expression. The in vivo preclinical studies are clearly demonstrating that maintaining Pax6 levels may contribute to improve beta cell function/dedifferentiation during T2D.

I have some comments concerning the study.

Concerning the AAV8 approach, the authors do not describe why they have used this serotype, and whether the AAVs infect both human and mouse pancreatic islets. Is the infection specific to pancreatic islets or are other tissues also affected after AAVs injection? How efficient is the transaction of AAVs in the middle of pancreatic islets?

A: AAV8 has been demonstrated to stably transduce the pancreas, including beta cells in islets. The tissue specificity would be greatly enhanced when combining with a tissue specific promoter. The combination of AAV8 and insulin promoter has been widely used to effectively transduce beta cells *in vivo* in a number of studies (PMID 31355778, 25676679, 25338552, 16567506, 18560422, 30400037). AAV8 is also able to efficiently transduce human islets as shown in this study (figure 5E) and other studies (PMID 29304344, 15858610). The use of AAV8 has been highlighted in the Discussion section (lines 422-427).

AAV-mediated exogenous PAX6 expression is observed in the middle of islets as shown in Appendix fig. S2B and S3B, while exogenous PAX6 is minimally detected in other tissues (liver, kidney, muscle, spleen) as shown in Appendix fig. S2C.

It is interesting to observe that Pax6 over expression modulates proliferation and/or apoptosis, in response (or not) to Insulin or Glp1. In the RNA-seq/ChIP-seq integrated analysis, none of the proliferative nor apoptosis pathways were found to be regulated by Pax6, although this seems to be the major functional effect (and, consequently, subsequent insulin secretion effect). The authors should, at least, discuss this point.

A: The RNA-seq/ChIP-seq integrated analysis only displays the pathways / genes that are directly regulated by PAX6 (genes bound by PAX6 and are also altered by PAX6 overexpression). However, PAX6 also regulates the expression / activity of other transcription factors or master regulators such as MAFA, PDX1, NKX6.1, CREB and Akt. These regulators which in turn regulate the expression of a bundle of effector genes, are well-

known to regulate beta cell function and survival. Those effector genes might not be directly bound by PAX6 and hence they are not displayed in the ChIP/ OE overlapping result. These points have been highlighted in the Result section (lines 369-373).

4th Oct 2023

Dear Prof. Han,

Thank you for the submission of your revised manuscript to EMBO Molecular Medicine. We have now received the reports from the 3 referees who re-reviewed your manuscript. As you will see, they are supportive of publication pending minor revisions, and we will therefore be able to accept your manuscript once the following editorial points will be addressed:

1/ Referees' comments:
Please address the remaining concerns (text editing) raised by referee #1.

2/ Main manuscript file:
- Please remove the blue text and only keep in track changes mode any new modification.
- Materials and methods:
o Mice: please indicate the gender of the mice and their age at time of experiments.
o Human islets: please include a statement that informed consent was obtained from each patient and that the experiments conformed to the principles set out in the WMA Declaration of Helsinki and the Department of Health and Human Services Belmont Report.
o Please provide antibody dilutions/concentrations
o Statistics: please include a statement about blinding and correct the checklist accordingly.
- Thank you for providing a Data Availability section. We kindly remind you that datasets must be available to the public before publication of your manuscript.
- Acknowledgements: All funding sources should be added to the submission system, and match the information provided in the manuscript.
- Author contributions: CRediT has replaced the traditional author contributions section because it offers a systematic machine-readable author contributions format that allows for more effective research assessment. Please remove the Authors Contributions from the manuscript and use the free text boxes beneath each contributing author's name in our system to add specific details on the author's contribution. More information is available in our guide to authors.
- Please rename the "conflict of interest" section to "Disclosure statement and competing interests". We updated our journal's competing interests policy in January 2022 and request authors to consider both actual and perceived competing interests. Please review the policy https://www.embopress.org/competing-interests and update your competing interests if necessary.

3/ Figures and Appendix:
- Please address the queries from our data editors in the figure legends:
• Please note that the figure legend style does not comply with the journal guidelines i.e. all the figre legends are in a run-on style.
• Please note that a separate 'Data Information' section is required in the legends of figures 1-7.
• Please note that quantification graphs for figures 1f, g are not placed properly in the figure.
• Please indicate the statistical test used for data analysis in the legends of figures 4a-b; 6a, c; 7a-d
• Although 'n' is provided, please describe the nature of entity for 'n' in the legends of figures 1a-h; 2a-h; 5a-h

- Please note that you have the possibility to have up to 5 Expanded View (EV) Figures that are collapsible/expandable online. EV Figures should be cited as 'Figure EV1, Figure EV2" etc... in the text and their respective legends should be included in the main text after the legends of regular figures. For the figures that you do NOT wish to display as Expanded View figures, they should be bundled together with their legends in a single PDF file called *Appendix*, which should start with a short Table of Content. Appendix figures should be referred to in the main text as: "Appendix Figure S1, Appendix Figure S2" etc. (Appendix legends should be removed from the main manuscript text).

4/ Thank you for providing 'The paper explained'. I included minor modifications, please let me know if you agree or amend as you see fit:
Problem
Loss of pancreatic beta cells is the central feature of all forms of diabetes. Current therapies fail to halt the declining beta cell mass. Islet transplantation is a beta cell replacement therapy adopted in clinical practice. However, post-transplant islet graft loss and insufficient supply of cadaveric islets limit the success and utility of this modality of treatment.

Results
This work identified PAX6 as a critical regulator of beta cell function and survival. PAX6 expression was reduced in beta cells or islets under diabetic conditions, associated with loss of functional beta cells. Replenishment of PAX6 preserved beta cells and led to a sustained maintenance of glucose homeostasis in diabetic mice. Moreover, PAX6 overexpression in human islets prior to transplantation promoted islet potency, preserved post-transplant islet graft and enhanced glycemic control in diabetic mice.

Impact
Through identification of PAX6 as a critical regulator of beta cell function, survival and identity, this work demonstrates that PAX6 gene transfer might overcome two of the major barriers of islet transplantation: post-transplant islet graft loss and scarcity of islet donors. These findings thus provide grounds to support future clinical translation of PAX6 gene delivery to overcome beta cell failure in diabetes.

5/ I slightly edited your synopsis to match our style and format. Please let me know if you agree with the following or amend as you see fit:
PAX6 was identified as a critical regulator of beta cell function, survival and identity. Furthermore, PAX6 gene delivery in beta cells promoted beta cell potency, preserved beta cells, and enhanced the capability of glycemic control.

- PAX6 was down-regulated in beta cells under diabetic conditions.
- PAX6 regulated insulin and incretin signalings in beta cells.
- AAV-PAX6 administration in STZ-induced diabetic mice and db/db mice preserved beta cells and ameliorated glycemic disturbance.
- AAV-PAX6 transduction in human islets prior to transplantation preserved post-transplant islet graft and improved glycemic control in diabetic mice.

6/ As part of the EMBO Publications transparent editorial process initiative (see our Editorial at http://embomolmed.embopress.org/content/2/9/329), EMBO Molecular Medicine will publish online a Review Process File (RPF) to accompany accepted manuscripts.
This file will be published in conjunction with your paper and will include the anonymous referee reports, your point-by-point response and all pertinent correspondence relating to the manuscript. Let us know whether you agree with the publication of the RPF and as here, if you want to remove or not any figures from it prior to publication.
Please note that the Authors checklist will be published at the end of the RPF.

I look forward to receiving your revised manuscript.

Yours sincerely,

Lise Roth

Lise Roth, PhD
Senior Editor
EMBO Molecular Medicine

***** Reviewer's comments *****

Referee #1 (Comments on Novelty/Model System for Author):

As noted, the model systems are good--mostly human cells, which is an important feature of this study. However, the "novelty" of the work remains questionable, notwithstanding its importance. I recognize that the authors were very defensive of the comments under "novelty and advance", both of which I still feel are low. That said, the study is of importance, which is a different issue (and that I did not dispute).

Referee #1 (Remarks for Author):

I think the introduction is a bit "over the top" in terms of length (4 pages) and the overall need for justification. The authors did add more insight into Pax4/Pax6 in the intro, as I requested, but did not edit the rest of it to highlight the key gaps in knowledge and the need for a study of this nature. I still think the introduction needs better focus and should set the stage for the rationale and importance of the work--which it fails to do.

Referee #2 (Remarks for Author):

All the issues that had been raised before have been adressed. The present version of the manuscript is clearly improved and I find it suitable for publication.

Referee #3 (Comments on Novelty/Model System for Author):

The manuscript has been improved, in terme of quality controls and new data that further strengthen the study. It is an important field that is addressed here, i.e. how to increase the quality/survival of human islets for transplantation. The multiple yet complementary approaches used in this study are convincing and should provide new insights for diabetes research/therapy.

Referee #3 (Remarks for Author):

The authors have replied to my concerns and have added several key experiments and controls that improve the quality of the study.

Dear Dr. Roth,
Thank you for your decision letter on 4 Oct 2023. We greatly appreciate the favorable comments from all 3 reviewers and have made changes to the text in the current revised version based on their suggestions. We have also followed the instructions from data editors to revise the manuscript, the synopsis and “the paper explained”. We also agree with the publication of the Review Process File. We hope that our response has addressed the remaining concerns and that the revised version is now ready for publication in *EMBO Molecular Medicine*.

Please find below our point-to-point response to the reviewers’ comments/ suggestions.

Referee #1 (Comments on Novelty/Model System for Author):

As noted, the model systems are good--mostly human cells, which is an important feature of this study. However, the "novelty" of the work remains questionable, notwithstanding its importance. I recognize that the authors were very defensive of the comments under "novelty and advance", both of which I still feel are low. That said, the study is of importance, which is a different issue (and that I did not dispute).

Referee #1 (Remarks for Author):

I think the introduction is a bit "over the top" in terms of length (4 pages) and the overall need for justification. The authors did add more insight into Pax4/Pax6 in the intro, as I requested, but did not edit the rest of it to highlight the key gaps in knowledge and the need for a study of this nature. I still think the introduction needs better focus and should set the stage for the rationale and importance of the work--which it fails to do.

A: We appreciate the comments. The introduction has been trimmed with some general background information deleted. In the revised version, we first state that loss of functional beta cells is a central event in both T1D and T2D and failure to rescue beta cell loss affects the efficacy of their treatments, which highlights the urgent need for strategies to preserve beta cells. Then we illustrate the important roles of PAX6 in mouse beta cell function and the association between PAX6 expression/activity with human T2D. After that, we highlight the key gaps in knowledge: most of the mechanistic studies of PAX6 are mainly done in mouse beta cells, while species differences do exist between human and rodent islets and evidence regarding the role of PAX6 in human beta cell survival is still missing. More importantly, whether PAX6 can serve as a therapeutic target for beta cell preservation remains ambiguous. These justify the study of PAX6 in human islets and the introduction ends with the findings of this study.

Referee #2 (Remarks for Author):

All the issues that had been raised before have been adressed. The present version of the manuscript is clearly improved and I find it suitable for publication.

A: We greatly appreciate the favorable comment.

Referee #3 (Comments on Novelty/Model System for Author):

The manuscript has been improved, in terme of quality controls and new data that further strengthen the study. It is an important field that is addressed here, i.e. how to increase the quality/survival of human islets for transplantation. The multiple yet complementary approaches used in this study are convincing and should provide new insights for diabetes research/therapy.

Referee #3 (Remarks for Author):

The authors have replied to my concerns and have added several key experiments and controls that improve the quality of the study.

A: We greatly appreciate the favorable comment.

13th Oct 2023

Dear Prof. Han,

Thank you for providing the revised files. I am pleased to inform you that your manuscript is accepted for publication and is now being sent to our publisher to be included in the next available issue of EMBO Molecular Medicine!

Please be reminded that the dataset must be publicly available before online publication of your article.

Please read below for additional IMPORTANT information regarding your article, its publication and the production process.

Congratulations on your interesting work,

Sincerely,

Lise Roth

Lise Roth, Ph.D
Senior Editor
EMBO Molecular Medicine

Follow us on Twitter @EmboMolMed
Sign up for eTOCs at embopress.org/alertsfeeds

*** *** *** IMPORTANT INFORMATION *** *** ***

SPEED OF PUBLICATION
The journal aims for rapid publication of papers, using using the advance online publication "Early View" to expedite the process: A properly copy-edited and formatted version will be published as "Early View" after the proofs have been corrected. Please help the Editors and publisher avoid delays by providing e-mail address(es), telephone and fax numbers at which author(s) can be contacted.

Should you be planning a Press Release on your article, please get in contact with embomolmed@wiley.com as early as possible, in order to coordinate publication and release dates.

LICENSE AND PAYMENT:

All articles published in EMBO Molecular Medicine are fully open access: immediately and freely available to read, download and share.

EMBO Molecular Medicine charges an article processing charge (APC) to cover the publication costs. You, as the corresponding author for this manuscript, should have already received a quote with the article processing fee separately. Please let us know in case this quote has not been received.

Once your article is at Wiley for editorial production you will receive an email from Wiley's Author Services system, which will ask you to log in and will present you with the publication license form for completion. Within the same system the publication fee can be paid by credit card, an invoice, pro forma invoice or purchase order can be requested.

Payment of the publication charge and the signed Open Access Agreement form must be received before the article can be published online.

PROOFS

You will receive the proofs by e-mail approximately 2 weeks after all relevant files have been sent o our Production Office. Please return them within 48 hours and if there should be any problems, please contact the production office at embopressproduction@wiley.com.

Please inform us if there is likely to be any difficulty in reaching you at the above address at that time. Failure to meet our deadlines may result in a delay of publication.

All further communications concerning your paper proofs should quote reference number EMM-2023-17928-V4 and be directed to the production office at embopressproduction@wiley.com.

EMBO Press Author Checklist

Corresponding Author Name: SO Wing Yan and HAN Weiping	
Journal Submitted to: EMBO Molecular Medicine	
Manuscript Number: EMM-2023-17928-V3	

USEFUL LINKS FOR COMPLETING THIS FORM
The EMBO Journal - Author Guidelines
EMBO Reports - Author Guidelines
Molecular Systems Biology - Author Guidelines
EMBO Molecular Medicine - Author Guidelines

Reporting Checklist for Life Science Articles (updated January
This checklist is adapted from Materials Design Analysis Reporting (MDAR) Checklist for Authors. MDAR establishes a minimum set of requirements in transparent reporting in the life sciences (see Statement of Task: 10.31222/osf.io/9sm4x). Please follow the journal's guidelines in preparing your
Please note that a copy of this checklist will be published alongside your article.

Abridged guidelines for figures
1. Data
The data shown in figures should satisfy the following conditions:
- the data were obtained and processed according to the field's best practice and are presented to reflect the results of the experiments in an accurate and unbiased manner.
- ideally, figure panels should include only measurements that are directly comparable to each other and obtained with the same assay.
- plots include clearly labeled error bars for independent experiments and sample sizes. Unless justified, error bars should not be shown for technical
- if n<5, the individual data points from each experiment should be plotted. Any statistical test employed should be justified.
- Source Data should be included to report the data underlying figures according to the guidelines set out in the authorship guidelines on Data

2. Captions
Each figure caption should contain the following information, for each panel where they are relevant:
- a specification of the experimental system investigated (eg cell line, species name).
- the assay(s) and method(s) used to carry out the reported observations and measurements.
- an explicit mention of the biological and chemical entity(ies) that are being measured.
- an explicit mention of the biological and chemical entity(ies) that are altered/varied/perturbed in a controlled manner.
- the exact sample size (n) for each experimental group/condition, given as a number, not a range;
- a description of the sample collection allowing the reader to understand whether the samples represent technical or biological replicates (including how many animals, litters, cultures, etc.).
- a statement of how many times the experiment shown was independently replicated in the laboratory.
- definitions of statistical methods and measures:
  - common tests, such as t-test (please specify whether paired vs. unpaired), simple χ2 tests, Wilcoxon and Mann-Whitney tests, can be unambiguously identified by name only, but more complex techniques should be described in the methods section;
  - are tests one-sided or two-sided?
  - are there adjustments for multiple comparisons?
  - exact statistical test results, e.g., P values = x but not P values < x;
  - definition of 'center values' as median or average;
  - definition of error bars as s.d. or s.e.m.

Please complete ALL of the questions below.
Select "Not Applicable" only when the requested information is not relevant for your study.

Materials

Newly Created Materials	Information included in the manuscript?	In which section is the information available? (Reagents and Tools Table, Materials and Methods, Figures, Data Availability Section)
New materials and reagents need to be available; do any restrictions apply?	Not Applicable	

Antibodies	Information included in the manuscript?	In which section is the information available? (Reagents and Tools Table, Materials and Methods, Figures, Data Availability Section)
For **antibodies** provide the following information:<br>- Commercial antibodies: RRID (if possible) or supplier name, catalogue number and or/clone number<br>- Non-commercial: RRID or citation	Yes	Materials and Methods

DNA and RNA sequences	Information included in the manuscript?	In which section is the information available? (Reagents and Tools Table, Materials and Methods, Figures, Data Availability Section)
**Short novel DNA or RNA including primers, probes**: provide the sequences.	Yes	Appendix table S1

Cell materials	Information included in the manuscript?	In which section is the information available? (Reagents and Tools Table, Materials and Methods, Figures, Data Availability Section)
**Cell lines:** Provide species information, strain. Provide accession number in repository **OR** supplier name, catalog number, clone number, and/**OR** RRID.	Yes	Materials and Methods
**Primary cultures:** Provide species, strain, sex of origin, genetic modification status.	Yes	Materials and Methods, Appendix table S2
Report if the cell lines were recently **authenticated** (e.g., by STR profiling) and tested for mycoplasma contamination.	Yes	Materials and Methods

Experimental animals	Information included in the manuscript?	In which section is the information available? (Reagents and Tools Table, Materials and Methods, Figures, Data Availability Section)
**Laboratory animals or Model organisms:** Provide species, strain, sex, age, genetic modification status. Provide accession number in repository **OR** supplier name, catalog number, clone number, **OR** RRID.	Yes	Materials and Methods
**Animal observed in or captured from the field:** Provide species, sex, and age where possible**.**	Not Applicable	
Please detail **housing and husbandry conditions**.	Yes	Materials and Methods

Plants and microbes	Information included in the manuscript?	In which section is the information available? (Reagents and Tools Table, Materials and Methods, Figures, Data Availability Section)
**Plants:** provide species and strain, ecotype and cultivar where relevant, unique accession number if available, and source (including location for collected wild specimens).	Not Applicable	
**Microbes:** provide species and strain, unique accession number if available, and source.	Not Applicable	

Human research participants	Information included in the manuscript?	In which section is the information available? (Reagents and Tools Table, Materials and Methods, Figures, Data Availability Section)
If collected and within the bounds of privacy constraints report on age, sex and gender or ethnicity for all study participants.	Not Applicable	

Core facilities	Information included in the manuscript?	In which section is the information available? (Reagents and Tools Table, Materials and Methods, Figures, Data Availability Section)
If your work benefited from core facilities, was their service mentioned in the acknowledgments section?	Not Applicable	

Design

Study protocol	Information included in the manuscript?	In which section is the information available? (Reagents and Tools Table, Materials and Methods, Figures, Data Availability Section)
If study protocol has been **pre-registered, provide DOI in the manuscript**. For clinical trials, provide the trial registration number **OR** cite DOI.	Not Applicable	
Report the **clinical trial registration number** (at ClinicalTrials.gov or equivalent), where applicable.	Not Applicable	

Laboratory protocol	Information included in the manuscript?	In which section is the information available? (Reagents and Tools Table, Materials and Methods, Figures, Data Availability Section)
Provide DOI OR other citation details if **external detailed step-by-step protocols** are available.	Not Applicable	

Experimental study design and statistics	Information included in the manuscript?	In which section is the information available? (Reagents and Tools Table, Materials and Methods, Figures, Data Availability Section)
Include a statement about **sample size** estimate even if no statistical methods were used.	Not Applicable	
Were any steps taken to minimize the effects of subjective bias when allocating animals/samples to treatment (e.g. **randomization procedure**)? If yes, have they been described?	Yes	Materials and Methods
Include a statement about **blinding** even if no blinding was done.	Yes	Materials and Methods
Describe **inclusion/exclusion criteria** if samples or animals were excluded from the analysis. Were the criteria pre-established?<br><br>If sample or data points were omitted from analysis, report if this was due to attrition or intentional exclusion and provide justification.	Yes	Materials and Methods
For every figure, are **statistical tests** justified as appropriate? Do the data meet the assumptions of the tests (e.g., normal distribution)? Describe any methods used to assess it. Is there an estimate of variation within each group of data? Is the variance similar between the groups that are being statistically compared?	Yes	Materials and Methods, figure legends

Sample definition and in-laboratory replication	Information included in the manuscript?	In which section is the information available? (Reagents and Tools Table, Materials and Methods, Figures, Data Availability Section)
In the figure legends: state number of times the experiment was **replicated** in laboratory.	Yes	Materials and Methods, figure legends
In the figure legends: define whether data describe **technical or biological replicates**.	Yes	Materials and Methods, figure legends

Ethics

Ethics	Information included in the manuscript?	In which section is the information available? (Reagents and Tools Table, Materials and Methods, Figures, Data Availability Section)
Studies involving **human participants**: State details of **authority granting ethics approval** (IRB or equivalent committee(s), provide reference number for approval.	Not Applicable	
Studies involving **human participants**: Include a statement confirming that **informed consent** was obtained from all subjects and that the experiments conformed to the principles set out in the WMA Declaration of Helsinki and the Department of Health and Human Services Belmont Report.	Not Applicable	
Studies involving **human participants:** For publication of **patient photos**, include a statement confirming that consent to publish was obtained.	Not Applicable	
Studies involving experimental **animals**: State details of **authority granting ethics approval** (IRB or equivalent committee(s), provide reference number for approval. Include a statement of compliance with ethical regulations.	Yes	Materials and Methods
Studies involving **specimen and field samples:** State if relevant **permits** obtained, provide details of authority approving study; if none were required, explain why.	Not Applicable	

Dual Use Research of Concern (DURC)	Information included in the manuscript?	In which section is the information available? (Reagents and Tools Table, Materials and Methods, Figures, Data Availability Section)
Could your study fall under dual use research restrictions? Please check biosecurity documents and list of **select agents and toxins** (CDC): https://www.selectagents.gov/sat/list.htm	Not Applicable	
If you used a select agent, is the security level of the lab appropriate and reported in the manuscript?	Not Applicable	
If a study is subject to dual use research of concern regulations, is the name of the **authority granting approval and reference number** for the regulatory approval provided in the manuscript?	Not Applicable	

Reporting

The MDAR framework recommends adoption of discipline-specific guidelines, established and endorsed through community initiatives. Journals have their own policy about requiring specific guidelines and recommendations to complement MDAR.

Adherence to community standards	Information included in the manuscript?	In which section is the information available? (Reagents and Tools Table, Materials and Methods, Figures, Data Availability Section)
State if relevant guidelines or checklists (e.g., **ICMJE, MIBBI, ARRIVE, PRISMA**) have been followed or provided.	Not Applicable	
For **tumor marker prognostic studies,** we recommend that you follow the **REMARK** reporting guidelines (see link list at top right). See author guidelines, under 'Reporting Guidelines'. Please confirm you have followed these guidelines.	Not Applicable	
For **phase II and III randomized controlled trials**, please refer to the **CONSORT** flow diagram (see link list at top right) and submit the CONSORT checklist (see link list at top right) with your submission. See author guidelines, under 'Reporting Guidelines'. Please confirm you have submitted this list.	Not Applicable	

Data Availability

Data availability	Information included in the manuscript?	In which section is the information available? (Reagents and Tools Table, Materials and Methods, Figures, Data Availability Section)
Have **primary datasets** been deposited according to the journal's guidelines (see 'Data Deposition' section) and the respective accession numbers provided in the Data Availability Section?	Yes	Data availability section
Were **human clinical and genomic datasets** deposited in a public access-controlled repository in accordance to ethical obligations to the patients and to the applicable consent agreement?	Not Applicable	
Are **computational models** that are central and integral to a study available without restrictions in a machine-readable form? Were the relevant accession numbers or links provided?	Not Applicable	
If publicly available data were reused, provide the respective **data citations in the reference list**.	Yes	Results, figure legends
